# Photocatalyzed hydrogen atom transfer enables multicomponent olefin oxo-amidomethylation under aerobic conditions

**DOI:** 10.1039/d5sc06277b

**Published:** 2025-11-18

**Authors:** Mattia Lepori, Dimitris I. Ioannou, Joshua P. Barham, Timothy Noël

**Affiliations:** a Flow Chemistry Group, Van't Hoff Institute for Molecular Sciences (HIMS), University of Amsterdam Amsterdam 1098XH The Netherlands t.noel@uva.nl; b Fakultät für Chemie und Pharmazie, Universität Regensburg Universitätsstraße 31 93040 Regensburg Germany Joshua-Philip.Barham@chemie.uni-regensburg.de; c Department of Pure & Applied Chemistry, University of Strathclyde 295 Cathedral Street Glasgow G1 1XL UK joshua.p.barham@strath.ac.uk

## Abstract

The direct functionalization of abundant and readily available feedstock chemicals has emerged as a powerful strategy to rapidly increase molecular complexity and access valuable scaffolds. Herein, we report a novel photocatalyzed three-component oxo-amidomethylation of aromatic olefins under aerobic conditions, enabling the synthesis of *N*-(γ-oxopropyl)amides *via* simultaneous incorporation of two orthogonal functional groups across the alkene C=C bond in a single step. Mechanistic features include the photocatalyzed hydrogen atom transfer (HAT)-mediated engagement of the α-*N*-alkyl C(sp^3^)–H bond in inexpensive, unfunctionalized amide feedstocks and the trapping of a key olefin-derived carbon-centered radical intermediate by molecular oxygen to afford the oxo-functionalized homologated products. This cascade protocol demonstrates compatibility with a broad range of aryl olefins and amides, as well as efficient scalability. The method provides streamlined access to high-value molecular architectures of synthetic and pharmaceutical relevance without the need for pre-functionalized radical precursors.

## Abstract 2

A novel photocatalytic oxo-amidomethylation of olefins is presented, achieving the synthesis of *N*-(γ-oxopropyl)amides using commodity chemicals and O_2_ as an atom-economical oxidant.

## Graphical abstract

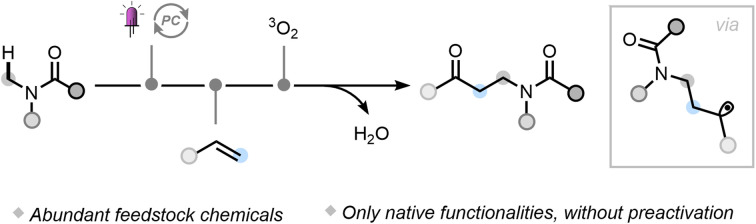

## Introduction

**Fig. 1:**
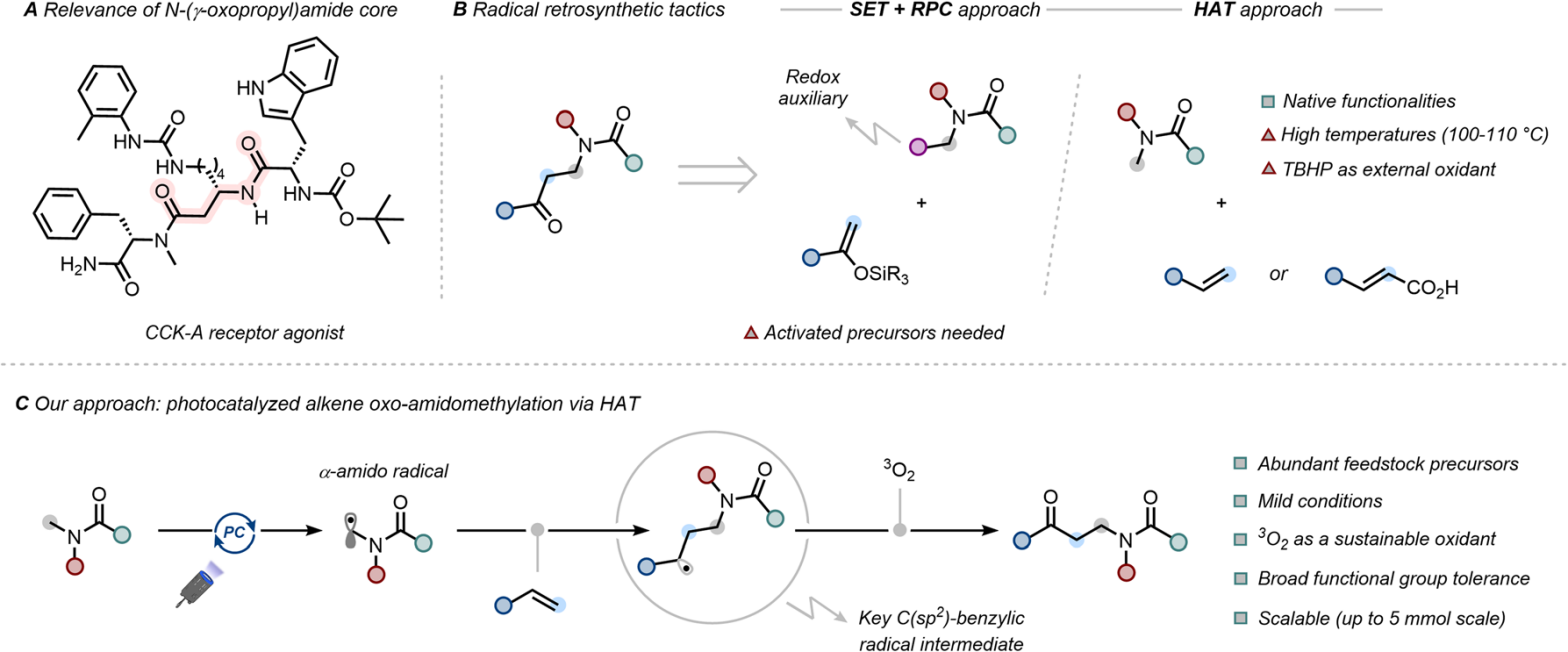
(A) Relevance of *N*-(γ-oxopropyl)amide core. (B) Retrosynthetic analyses and conventional radical tactics for alkene oxo-amidomethylation. (C) Our approach: photocatalyzed alkene oxo-amidomethylation *via* direct hydrogen atom transfer (HAT).

The *N*-(γ-oxopropyl)amide core constitutes a versatile platform for the development of pharmaceutically and biologically relevant compounds, exemplified by its incorporation into a Asp32-replacement peptide analogue that exhibits potent agonist activity at the pancreatic CCK-A receptor (Fig. 1A).^1^ The privileged character of the *N*-(γ-oxopropyl)amide core is underscored by its versatility as a precursor to (i) β-aminoketones, key intermediates in the synthesis of heterocycles and natural product frameworks;^2^ and (ii) γ-amino alcohols, widely used as ligands and as building blocks in drug development.^3–5^

Despite this synthetic potential, conventional polar chemistry provides limited access to the *N*-(γ-oxopropyl)amide space, with only a narrow scope of derivatives accessible *via* Mukaiyama-type reactions of 1,3,5-trialkylhexahydro-1,3,5-triazines with silyl enol ethers,^6^ or through oxidative ring-openings of tetrahydropyridines.^7,8^ In contrast, radical chemistry offers a compelling alternative, unlocking two key retrosynthetic disconnections (Fig. 1B).^9^ The first exploits the merger of reductive single electron transfer (SET)^10,11^ to tailored redox-active precursors (*e.g. N*-hydroxyphthalimide (NHPI)-based redox-active esters, Katritzky salts)^12,13^ with oxidative radical polar crossover (RPC)^14–16^ following Giese-type addition to a silyl enol ether. While effective, this method requires two pre-activated reaction partners, limiting its operational flexibility. Recently, and as a second retrosynthetic tactic, the rapid expansion of multicomponent reactions (MCRs),^17,18^ particularly three-component alkene difunctionalizations, has emerged as an attractive solution. The main reasons are: *(i)* olefins are inexpensive and readily available organic feedstocks;^19^*(ii)* the simultaneous introduction of two orthogonal functionalities across the C=C bond results in a rapid increase in molecular complexity;^20–24^ and *(iii)* the uninterrupted cascade nature of such transformations obviates the need for intermediate purifications.^25^ In the context of alkene oxo-functionalization,^26^ radical precursors such as redox-active esters^27,28^ or aryl sulfides can be proposed,^29^ with dimethyl sulfoxide (DMSO) serving as a ketone synthon *via* Kornblum oxidation.^30^ To date, however, these strategies have not been extended to the synthesis of *N*-(γ-oxopropyl)amides, likely due to the need for pre-installed *ad hoc* functionalities enabling SET-driven α-amido radical generation.^31^

We envisioned overcoming this challenge by exploiting the native, unfunctionalized amide moiety itself as a precursor to access α-amido radicals *via* hydrogen atom transfer (HAT).^32–34^ Within this area, a thermal strategy has been demonstrated using styrenes or cinnamic acids as radical acceptors.^35–37^ Amide *N*-methyl group functionalization was achieved *via* C(sp^3^)–H bond fragmentation promoted by the peroxyl radical derived from *tert*-butyl hydroperoxide (TBHP),^38^ furnishing the oxo-amidomethylated product upon Kornblum–DeLaMare rearrangement.^39^ However, the use of superstoichiometric amounts of oxidant and high temperatures (100–110 °C) limits its synthetic generality. Inspired by our efforts in multicomponent reactions,^40,41^ homologation of amides with styrenes,^42,43^ and use of gaseous reactants,^44–47^ we conceived a light-promoted catalytic HAT activation of amides for one-carbon alkene elongation, using molecular oxygen as a non-toxic, green and atom-economical oxidant for carbonyl installation.^48,49^ Herein, we report the realization of this design, enabling the three-component oxo-amidomethylation of a broad range of aromatic alkenes with different amides under aerobic conditions in a operationally simple, robust and scalable methodology (Fig. 1C).

## Results and discussion

Our experimental investigation commenced with the optimization of the photocatalyzed alkene oxo-amidomethylation. Extensive screening of all relevant reaction parameters (for further details, see Section 5, SI) revealed that the desired difunctionalized product 3 could be obtained in 75% yield when a solution of styrene 1a (0.2 mmol) as radical acceptor and *N*,*N*-dimethylacetamide (DMA, 2a) as C–H donor in the presence of tetra-*n*-butylammonium decatungstate (*n*-Bu_4_N)_4_W_10_O_32_ (TBADT, 3 mol%) as a HAT photocatalyst in a 1 : 0.4 v/v mixture of acetonitrile (CH_3_CN) and 1 M aqueous HCl was irradiated with violet LEDs (52 W, 390 nm Kessil lamp) for 16 h in a standardized UFO reactor (Table 1, Entry 1).^50^ Remarkably, no photocatalytic styrene oxidative cleavage to benzaldehyde was observed under optimized conditions.^51,52^ Among the catalysts tested, TBADT proved to be the most effective, outperforming commonly employed HAT organophotocatalysts such as anthraquinone-2,7-disulfonic acid disodium salt (AQDS)^53^ and 4,4′-dichlorobenzophenone (4Cl_2_-BP) (Entry 2).^54^ The role of the aqueous 1 M HCl additive was investigated through control experiments employing either water alone or tetra-*n*-butylammonium chloride (*n*-Bu_4_N·Cl) as a chloride ion source, which led to moderate yields or complete suppression of the reaction, respectively (Entry 3). These results strongly imply that the primary role of HCl is to establish acidic conditions in the reaction medium, which boosts the reactivity of decatungstate and preserves its catalytic integrity.^45^ The hypothesis was supported by a comparable yield obtained upon replacing HCl with trifluoroacetic acid (TFA) under otherwise identical conditions. In contrast, the use of basic additives such as Na_3_PO_4_ resulted in a diminished yield, likely due to the instability of the decatungstate anion under basic conditions (Entry 4).^55^ Removal of 1 M HCl led to a lower yield, underscoring the prominent impact of acidic conditions in accelerating the process (Entry 5).^45^ Our observation is consistent with the previously proposed role of controlled aqueous acidic medium in suppressing the formation of undesired tungsten oxide clusters.^55–62^ Halving the amount of the C–H donor 2a led to a reduced, yet synthetically useful yield (Entry 6). Furthermore, omitting the pre-irradiation sparging of the reaction mixture with oxygen led to a diminished yield, suggesting initial gas–liquid mass transfer is important to initiate the reaction effectively (Entry 7). The complete exclusion of the gaseous reagent entirely suppressed the process (Entry 8). Finally, no desired product was observed in the absence of the photocatalyst (Entry 9).

**Table 1:** Optimization of alkene oxo-amidomethylation conditions

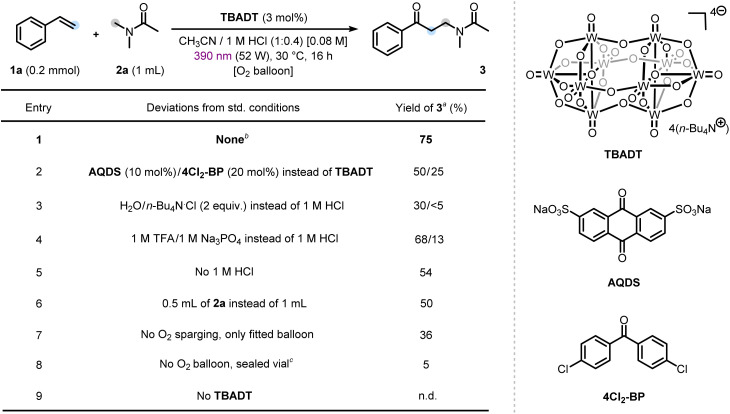

a Yields were determined by ^1^H NMR using 1,1,2-trichloroethylene as an internal standard. Reactions performed on a 0.2 mmol scale.

b Reaction mixture was sparged for 5 minutes with oxygen before fitting the vial with an oxygen balloon for the irradiation time. For further details, see the SI. n.d. = not detected.

c Reaction mixture prepared and sealed under air.

**Fig. 2:**
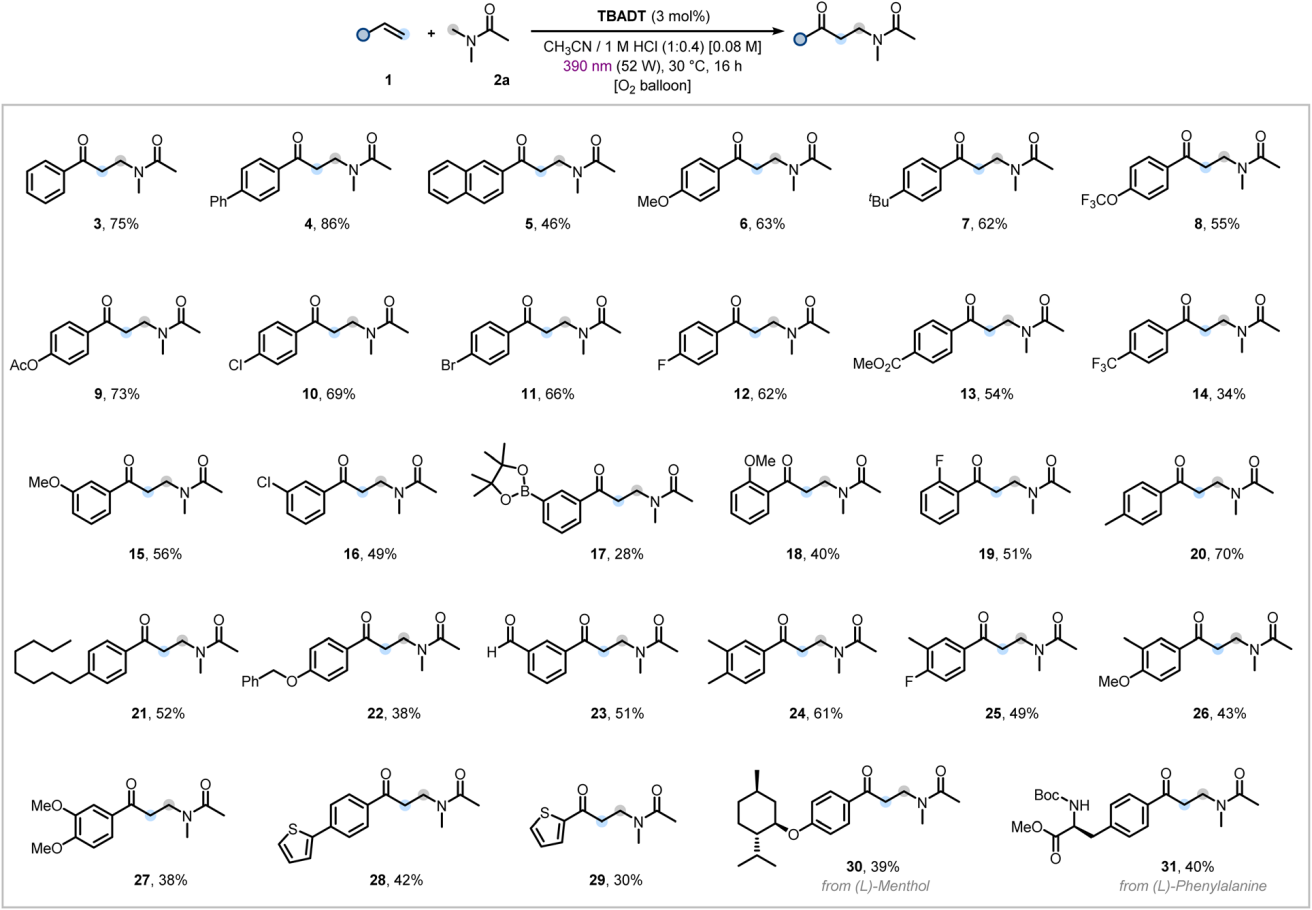
Scope of the alkene oxo-amidomethylation using *N*,*N*-dimethylacetamide (2a). Reaction conditions: styrene 1 (0.3 mmol, 1.0 equiv.), 2a (1.5 mL), TBADT (3 mol%), 1 M aqueous HCl (0.6 mL) in anhydrous CH_3_CN (1.5 mL). For further details, see the SI. Isolated yields are reported.

With the optimized conditions in hand, we next set about assessing the generality of our three-component protocol. Initially, *N*,*N*-dimethylacetamide (2a) was combined with a variety of aromatic olefins (Fig. 2). First efforts focused on variation of the aryl moiety, affording products (3–5) moderate to excellent yields from biphenyl- and naphthalene-derived alkenes. We then evaluated the effect of substituents at the *para*-position of styrene derivatives. Strongly (OMe) and moderately (*tert*-Bu) electron-donating groups delivered the corresponding difunctionalized products in good yields (6 and 7). Notably, analogues bearing trifluoromethoxy and acetoxy substituents were also well tolerated (8 and 9), with no evidence of ester hydrolysis under our mild acidic reaction conditions. For moderately electron-withdrawing groups, halogenated styrenes (Cl, Br, F) were successfully converted to the desired products (10–12), with the preserved C(sp^2^)–X bonds providing versatile handles for downstream functionalization *via* classical transition metal-catalyzed cross-coupling reactions.^63^ Pleasingly, styrenes containing stronger electron-withdrawing substituents (carboxylic methyl ester, trifluoromethyl) were reactive under the optimized conditions, affording products in modest to moderate yields (13 and 14). *meta*-Substituted styrenes were suitable radical acceptors (15 and 16), including the boronic ester-bearing olefin that was successfully engaged under our aerobic conditions, albeit with a lower yield (17).

**Fig. 3:**
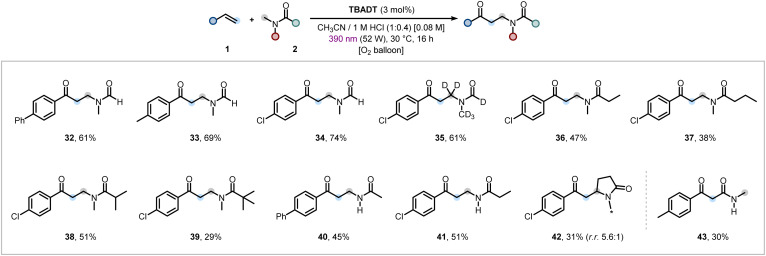
Scope of the alkene oxo-amidomethylation varying amide partner. Reaction conditions: styrene 1 (0.3 mmol, 1.0 equiv.), amide 2 (1.5 mL), TBADT (3 mol%), 1 M aqueous HCl (0.6 mL) in anhydrous CH_3_CN (1.5 mL). For further details, see the SI. Isolated yields are reported.

**Fig. 4:**
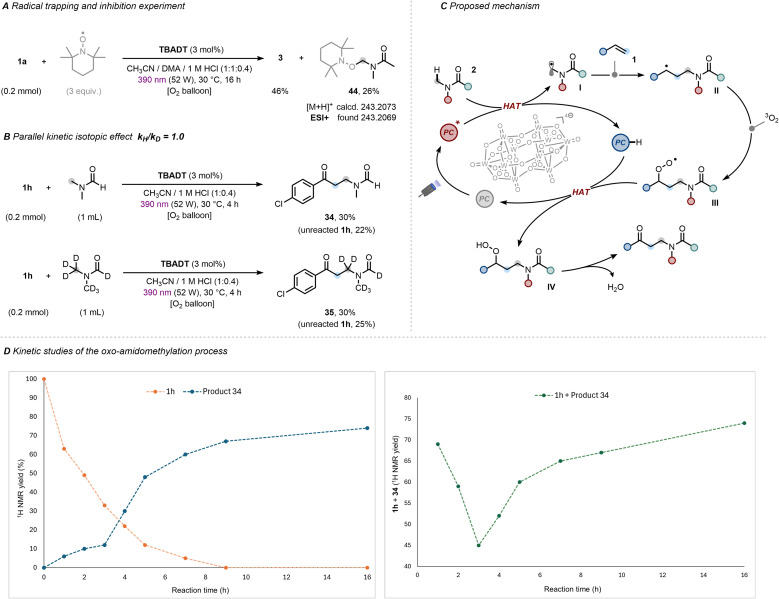
(A) Radical trapping and inhibition experiment. (B) Kinetic isotope effect (KIE). (C) Proposed mechanism. (D) *Left*: Temporal evolution of 4-chlorostyrene (1h, orange) and oxo-amidomethylated product 34 (blue); *Right*: Temporal evolution of combined starting material (1h) and product 34 (mass balance).

Finally, *ortho*-substituted styrenes bearing methoxy and fluoro groups furnished the oxo-amidomethylated products (18 and 19), though the yields were diminished likely due to increased steric hindrance and disordered conjugation between the arene and alkene moieties,^64^ impacting the Giese-type addition of the α-amido radical (*cf.*Fig. 4C). Next, the method was applied to styrenes bearing substituents with activated C–H bonds which could be engaged by the excited state of TBADT to give undesired products. These substituents included benzylic methyl, benzylic methylene, benzyloxy, and aldehydic groups (BDE ≃ 84–88 kcal mol^−1^).^32^ Gratifyingly, all were effectively tolerated under the reaction conditions, affording products in modest to good yields (20–23). *para*–*meta*-Disubstitution had little impact on the reaction outcome, enabling the desired functionalizations in synthetically useful yields (24–27). The heteroaromatic scaffold thiophene was also tolerated, both when conjugated to a classical styrene and when directly attached to the alkenyl moiety, affording the respective products in modest to moderate yields (28 and 29). To further demonstrate the synthetic utility of our method, bioactive and natural product containing olefins, such as (l)-menthol and (l)-phenylalanine, were successfully engaged as radical acceptors (30 and 31). Non-aromatic olefins were found to be unreactive under the optimized conditions. We subsequently examined the scope of C–H donors and demonstrated that a series of inexpensive and readily available amides were viable for our homologative oxo-functionalization reaction (Fig. 3). In particular, *N*,*N*-dimethylformamide (DMF) was found to react smoothly under aerobic conditions with both electron-rich and electron-poor styrenes, affording the desired products in good yields (32–34). Its deuterated analogue, DMF-*d*_7_, also underwent the transformation effectively (35). The yield only decreased by 1/5 compared to the product from DMF-*h*_7_ (34), suggesting the photocatalyst does not discriminate well between the difference in C–H and C–D bond strengths (*cf.*Fig. 4B for kinetic isotope effect discussion). Subsequently, various *N*,*N*-dimethylamides bearing different acyl group derivatives were subjected to reaction with 4-chlorostyrene (1h), delivering the corresponding products in moderate to good yields (36–39). Additionally, *N*-methyl-substituted alkyl amides and cyclic amides such as *N*-methylpyrrolidinone (NMP) were successfully engaged as reaction partners under the optimized conditions (40–42). Finally, when *N*-methylformamide was employed, a regiodivergent functionalization was observed: the formyl C(sp^2^)–H bond was selectively engaged over the *N*-methyl group,^65,66^ leading to the formation of an appealing 1,3-dicarbonyl scaffold in modest yield (43).^67^

Next, a series of additional experiments was conducted to gain mechanistic insights into the developed alkene difunctionalization process. First, involvement of the α-amido radical species was confirmed by the formation of adduct 44, detected in 26% yield upon addition of the radical scavenger 2,2,6,6-tetramethylpiperidine-1-oxyl (TEMPO) under standard reaction conditions (Fig. 4A).^68^

Kinetic isotope effect (KIE) experiments performed *via* a parallel reaction approach furnished a *k*_H_/*k*_D_ value of 1.0 (Fig. 4B). A competitive KIE experiment yielded the same result (for further details, see Section 7.5, SI). This indicated that HAT is either *(i)* not involved in the rate-determining step;^69^ or *(ii)* that direct HAT involving the excited state PC* is a process sufficiently high in energy that it does not recognize the difference in BDE between C–H and C–D bonds for a KIE to manifest. On the basis of our mechanistic findings and those of previous studies,^70–76^ a proposed mechanism is presented in Fig. 4C. Upon absorption of violet light, the excited state of TBADT triggers the hydrogen abstraction from the α-*N*-methyl C(sp^3^)–H bond of amide 2 (BDE ≃ 89 kcal mol^−1^)^77^*via* direct HAT, generating the nucleophilic α-amido radical I. The latter engages in a Giese-type addition to 1, yielding benzylic radical intermediate II. At this stage, II is trapped by triplet oxygen (^3^O_2_), forming peroxyl radical III. This subsequently reacts with the reduced catalyst species, TBADT–H, in a putative back-HAT step,^74,78^ yielding the hydroperoxide intermediate IV and closing the catalytic cycle. Upon elimination of water, IV releases the oxo-amidomethylated product.^79,80^ Further insight into the reaction mechanism was gained from kinetic experiments. The kinetic profile of the reaction between 4-chlorostyrene (1h) and DMF (2b) is shown in Fig. 4D, left and reveals two distinctive features: *(i)* a rapid consumption of 4-chlorostyrene (*orange trace*) during the first three hours of irradiation; and *(ii)* a comparatively slow formation of the oxo-amidomethylated product (*blue trace*) within this period (around 10% yield), followed by a pronounced increase after 3–5 hours (30% yield after 4 h and 48% after 5 h, respectively). Although complete conversion of the starting material is reached after 9 hours (67% yield), the product yield continues to increase until 16 hours (74% yield). While inspecting the sigmoidal-looking shaped product/time profile alone one would be tempted to assign autocatalytic behaviour that is being unearthed in photochemical reports in recent years.^81–83^ However, this would be a mis-assignment because the starting material/time curve does not reflect this profile. A further clue arose from considering the combined mole fractions of starting material and product over time (overall ‘mass balance’, *green trace*), which starts at ∼70%, drops to ∼45% after 3 hours, and gradually returns to ∼70% after 16 hours (Fig. 4D, right).^84^ This kinetic behaviour strongly points to the accumulation of a (meta)stable intermediate that slowly evolves into the final difunctionalized product under prolonged reaction time. Although this species could not be directly detected or isolated, we assign it as the benzylic hydroperoxide intermediate IV, whose involvement was further corroborated by quenching experiments with triethyl phosphite (for further details, see Section 7.1, SI).^45,56^ Overall, since the rapid consumption of 1h suggests that the Giese-type addition occurs readily and the subsequent trapping of II by molecular oxygen is known to be extremely fast,^85,86^ the breakdown of intermediate IV is proposed to constitute the rate-determining step of the oxo-amidomethylation reaction sequence. Since the C–H bond involved in the breakdown of IV involved derives from styrene 1h, this consists with the lack of observed KIE when DMF-*d*_7_ was used.

**Fig. 5:**
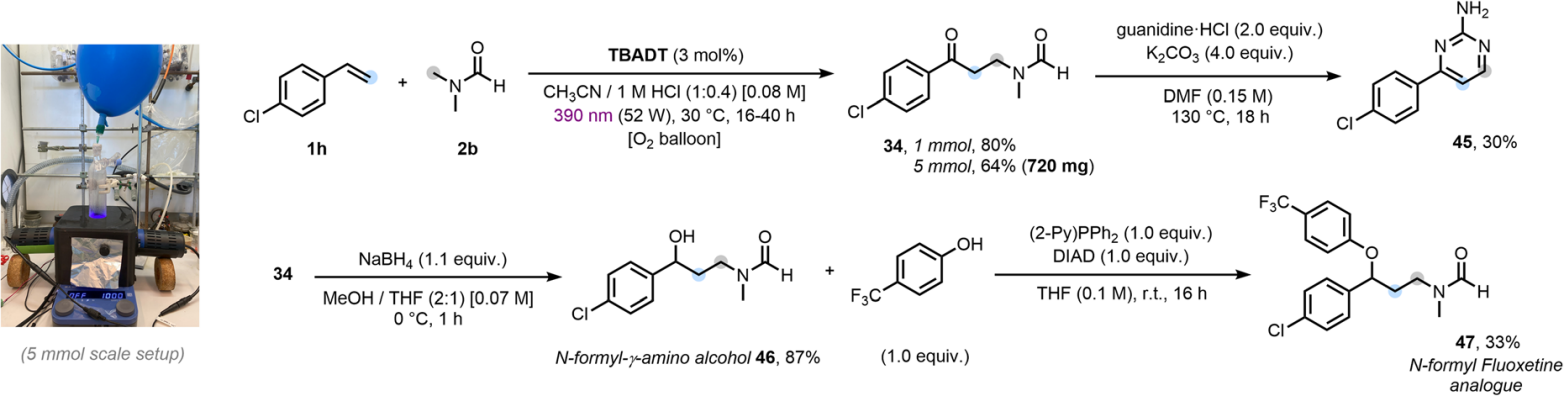
Scale up and product post-functionalizations. For further details, see the SI. Isolated yields are reported.

Finally, we demonstrated the scalability of our process and the synthetic versatility of the obtained *N*-(γ-oxopropyl)amide core (Fig. 5). The three-component protocol proved to be readily scalable up to a 5 mmol scale. While irradiation for 16 h afforded a good yield on a 1 mmol scale, scaling to 5 mmol required two violet lamps (52 W, 390 nm Kessil) and an extended irradiation time of 40 h, enabling the synthesis of over 0.7 g of 34 (for further details, see Section 8, SI). In light of the biological relevance of pyrimidine-based derivatives,^87^ the oxo-amidomethylated intermediate 34 was leveraged to access cyclized product 45 in a single step, offering a compelling alternative to the commonly employed β-amino enone precursors.^88^ Moreover, we showcased the potential of 34 as building block for the synthesis of a structural analogue of *N*-formyl fluoxetine^89^*via* a two-step synthetic route. Ketone reduction furnished the protected γ-amino alcohol scaffold 46, which, upon Mitsunobu etherification, afforded compound 47. Therefore, both an arylpyrimidine-2-amine motif and a structural analogue of *N*-formyl fluoxetine were accessed in 2–3 concise steps from cheap, commercial C/N/O-containing building blocks: styrene, DMF, O_2_, guanidine and a phenol.

## Conclusions

In summary, we have developed an operationally simple, modular and scalable multicomponent oxo-amidomethylation of alkenes, starting from abundant and cost-effective feedstock materials. This homologative cascade process involves photocatalyzed hydrogen atom transfer (HAT) engagement of amides, Giese-type radical addition of α-amido radicals to alkenes, and subsequent benzylic radical trapping by abundant molecular oxygen to provide streamlined access to synthetically valuable *N*-(γ-oxopropyl)amides. The method addresses key limitations of conventional approaches by *(i)* offering broad functional group tolerance across diverse aryl olefins and amides, *(ii)* operating under mild conditions, and *(iii)* circumventing the need for redox-auxiliary containing precursors or stoichiometric peroxides. Overall, this work offers practical advantages in terms of step economy, sustainability and modularity, enriching the current toolbox for multicomponent alkene difunctionalizations and underscoring its potential for immediate applications in drug discovery and development.

## Author contributions

M. L. developed the reaction conditions, conducted the majority of the scope and mechanistic experiments and wrote the first draft of the manuscript under the guidance of J. P. B. and T. N. D. I. I. contributed to the substrate scope and product derivatization. J. P. B. and T. N. conceptualized the project and co-supervised M. L. and D. I. I. in their contributions through monthly meetings. J. P. B. dealt with peer-review of the manuscript, directed planned revision experiments with input from all authors, and conceptualized the cover artwork. M. L. conducted all revision experiments. All authors have approved the final version of the manuscript.

## Conflicts of interest

There are no conflicts to declare.

## Supplementary Material

SC-016-D5SC06277B-s001

## Data Availability

All data supporting this article are available as part of the article and its supplementary information (SI). Supplementary information is available. See DOI: https://doi.org/10.1039/d5sc06277b.
