# Supplementary material for: Photocatalyzed hydrogen atom transfer enables multicomponent olefin oxo-amidomethylation under aerobic conditions
Source: Chem Sci. 2025 Nov 18;16(48):22944–51. doi: 10.1039/d5sc06277b (PMC12679465; doi:10.1039/d5sc06277b)
Supplement: SC-016-D5SC06277B-s001 [file SC-016-D5SC06277B-s001.pdf]

Supplementary Information (SI) for Chemical Science.
This journal is © The Royal Society of Chemistry 2025

Supplementary Information

Photocatalyzed hydrogen atom transfer enables multicomponent olefin oxo-amidomethylation under aerobic conditions

Mattia Lepori,[ab] Dimitris I. Ioannou,[a] Joshua P. Barham,*[bc] and Timothy Noël*[a]

[a] Flow Chemistry Group, Van't Hoff Institute for Molecular Sciences (HIMS), University of Amsterdam, Amsterdam 1098 XH, The Netherlands.

[b] Fakultät für Chemie und Pharmazie, Universität Regensburg, Universitätsstraße 31, 93040 Regensburg, Germany.

[c] Department of Pure & Applied Chemistry, University of Strathclyde, 295 Cathedral Street, Glasgow G1 1XL, U.K.

* Email(s): joshua.p.barham@strath.ac.uk; Joshua-Philip.Barham@chemie.uni-regensburg.de; t.noel@uva.nl

Table of Contents

1. General information 1

2. Chart of starting materials used in the scope 2

3. Synthesis of starting materials 3

3.1 General Procedure (**GP1**) for the synthesis of aromatic olefins 3

3.2 Synthesis of **1z** 3

3.3 Synthesis of **1ab** 4

3.4 Synthesis of **1ac** 4

3.5 Synthesis of **2g** 5

4. Photoreaction setup 6

5. Optimization of the photocatalyzed alkene oxo-amidomethylation 8

6. General Procedure (**GP2**): Photocatalyzed alkene oxo-amidomethylation 12

7. Mechanistic investigation 13

7.1 Quenching experiments 13

7.2 Radical trapping and inhibition experiment 14

7.3 Reactivity of *α*-methylstyrene 16

7.4 Reaction kinetics 19

7.5 Kinetic isotope effect (KIE) 20

8. Scale-up procedures 21

9. Post-functionalizations 23

9.1 Synthesis of **45** 23

9.2 Synthesis of **46** 23

9.3 Synthesis of **47** 24

10. Characterization data of aryl olefins 25

11. Characterization data of oxo-amidomethylation products 27

12. Limitations of the scope 41

13. NMR spectra of oxo-amidomethylation products 43

14. NMR spectra of post-functionalizations 91

15. References 95

1. General information

All reagents and solvents were used as received without further purification, unless stated otherwise. Reagents, reactants and solvents were purchased from Sigma Aldrich, TCI, FluoroChem, BLD-Pharm and Fisher Scientific. If applicable, reagents, reactants and solvents were kept under nitrogen (N_2) atmosphere. Apart from photocatalytic reactions, unless stated otherwise, reactions were carried out under an inert (N_2) atmosphere. Cryogenic conditions (−78 °C) were achieved using dry ice/acetone baths. Temperatures of 0 °C were obtained by means of an ice bath or ice/salt bath. 'Room temperature' (r.t.) indicates temperatures in the range of 20-25 °C.

The photocatalyst, tetra-*n*-butylammonium decatungstate ($(n\text{-}Bu_4N)_4W_{10}O_{32}$) (**TBADT**), was prepared according to a published procedure.[1] Disposable syringes were purchased from B. Braun. Product isolation was performed manually, using silica (P60, SILICYCLE) or automatically, using Biotage® Isolation Four. TLC analysis was performed using Silica on aluminum foil-backed TLC plates (Macherey Nagel: Alugram Xtra SIL G UV254 Nr. 818333, thickness 0.2 mm) and visualized under ultraviolet light (254 nm and 365 nm) or with an appropriate TLC stain (cerium ammonium molybdate or potassium permanganate).

^{1}H NMR spectra were recorded on Bruker AV300-I (300 MHz) or Bruker AV400 (400 MHz) spectrometers. Chemical shifts are reported in parts per million (ppm) using $CDCl_3$ (7.26 ppm) as an internal reference. The following abbreviations are adopted to describe the multiplicity: br. s (broad singlet), s (singlet), d (doublet), t (triplet), q (quartet), p (pentet), dd (doublet of doublets), td (triplet of doublets), tt (triplet of triplets), dq (doublet of quartets), ddd (doublet of doublets of doublets), ddt (doublet of doublets of triplets), dtd (doublet of triplets of doublets), m (multiplet). Coupling constants (J) are reported in hertz (Hz) and are uncorrected. ^{13}C NMR spectra were recorded on Bruker AV 300-I (75 MHz) spectrometer, run in ^{1}H-decoupled mode. Chemical shifts for ^{13}C NMR were reported in parts per million, using $CDCl_3$ (77.16 ppm, center line signal of the triplet) as an internal reference. Where appropriate, COSY, DEPT, HSQC and HMBC were carried out to aid assignment. ^{19}F NMR spectra were recorded on Bruker AV300-I (282 MHz) spectrometer. ^{11}B NMR spectra were recorded on Bruker AV300-I (96 MHz) spectrometer. All NMR data were processed using the MestReNova 12.0.2 software package. The names of all products were generated using the PerkinElmer ChemDraw v.23.1.2 software package. Known products were characterized by comparison to the corresponding ^{1}H NMR and ^{13}C NMR from the respective literature. The majority of the compounds described in this work were isolated as mixtures of rotamers. In some entries, not all rotameric signals could be detected in the ^{13}C NMR spectra.

High resolution mass spectra (HR-MS) were collected on an AccuTOF LC, JMS-T100LP Mass spectrometer (JEOL, Japan), where the UV-Vis spectra were recorded with a double beam spectrophotometer (Shimadzu UV2600) equipped with a deuterium lamp (190-350 nm), a halogen lamp (330-900 nm) and a photomultiplier (Hamamatsu R928) detector. GC-MS data were collected on an Agilent 5977C GC/MSD system.

For the photochemical small scale (0.2 mmol) and scale-up experiments (1.0 mmol), a 3D-printed (PLA) reactor was used, internally coated with aluminum foil and equipped with a specific 3D-printed (PLA) lid serving as vials holder and lamp holder (see **Section 4** for details).

2. Chart of starting materials used in the scope

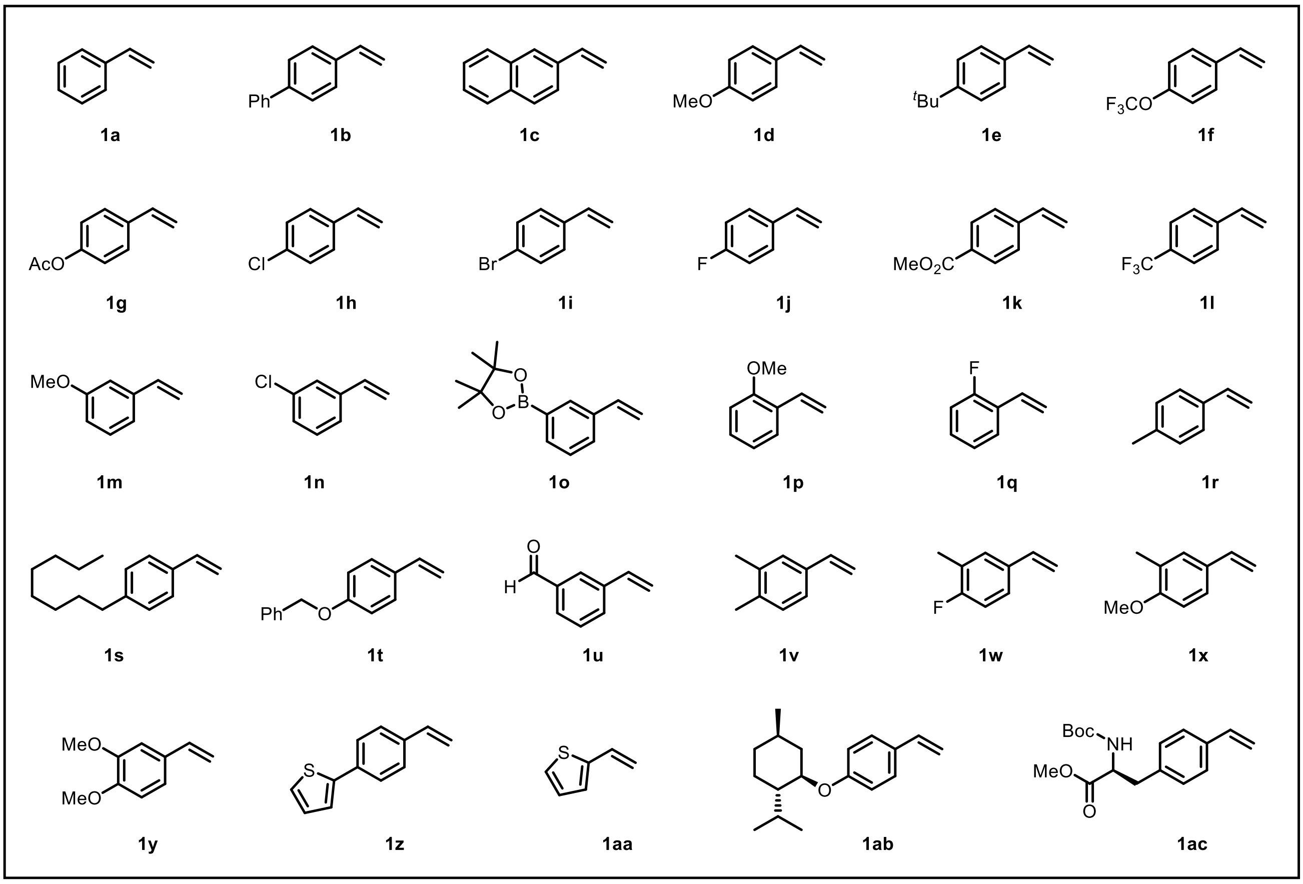

Figure S1: Structures of styrenes **1** used in the substrate scope and optimization studies.

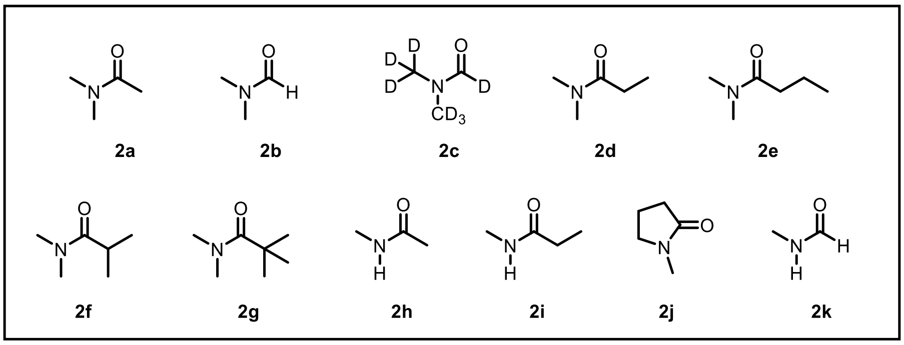

Figure S2: Structures of amides **2** used in the substrate scope and optimization studies.

3. Synthesis of starting materials

3.1 General Procedure (GP1) for the synthesis of aromatic olefins

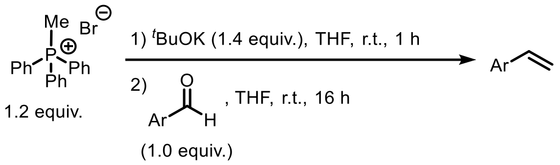

Aromatic olefins **1f**, **1q, 1t** and **1v-1x** were synthesized adapting a procedure reported in the literature.[2] To a suspension of methyltriphenylphosphonium bromide (1.2 equiv) in dry THF (5.0 mL per mmol of phosphonium salt) under N_2 atmosphere, a solution of ^{t}BuOK (1.4 equiv) in THF (1.0 mL/mmol) was added. The resulting bright yellow mixture was stirred at r.t. for 1 h. Then, the aldehyde (1.0 equiv.) was added portionwise and the resulting mixture was stirred at r.t. for 16 h. Upon completion, pentane (3.0 mL/mL of THF) was added and the precipitate was filtered over celite. The filtrate was concentrated *in vacuo* and the residue purified via flash column chromatography on silica gel. See **Section 10** for characterization data of **1f**, **1q, 1t** and **1v**-**1x**.

3.2 Synthesis of 1z

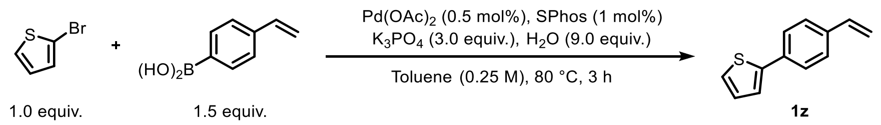

Compound **1z** was synthesized adapting a procedure reported in the literature.[3] An oven-dried pressure tube was charged with $Pd(OAc)_2$ (3.0 mg, 0.5 mol%), SPhos (10.0 mg, 1.0 mol%), (4-vinylphenyl)boronic acid (550.0 mg, 3.8 mmol, 1.5 equiv.) and K_3PO_4 (1.59 g, 7.5 mmol, 3.0 equiv.). After sealing the tube, it was evacuated and back-filled with N_2 three times. Toluene (10 mL), 2-bromothiophene (0.24 mL, 2.5 mmol, 1.0 equiv.) and H_2O (0.40 mL, 22.5 mmol, 9.0 equiv.) were added via syringe. The resulting mixture was stirred at 80 °C for 3 h. Upon completion, EtOAc (20 mL) and water (20 mL) were added. The aqueous layer was extracted with EtOAc (3 x 15 mL) and the combined organic phase was washed with sat. aq. NaCl (2 x 30 mL) and dried over anhydrous Na_2SO_4. The crude reaction mixture was then concentrated *in vacuo* and purified via flash column chromatography on silica gel (Pentane) to afford **1z** as a white solid (389.0 mg, 84% yield). Characterization data are in accordance with the literature.[4]

^{1}H NMR (300 MHz, $CDCl_3$) δ 7.58 (d, *J* = 8.4 Hz, 2H), 7.42 (d, *J* = 8.3 Hz, 2H), 7.32 (dd, *J* = 3.6, 1.2 Hz, 1H), 7.28 (dd, *J* = 5.1, 1.1 Hz, 1H), 7.08 (dd, *J* = 5.1, 3.6 Hz, 1H), 6.72 (dd, *J* = 17.6, 10.9 Hz, 1H), 5.77 (d, *J* = 17.6, 1H), 5.27 (d, *J* = 10.9, 1H).
^{13}C NMR (75 MHz, $CDCl_3$) δ 144.3, 136.9, 136.4, 134.0, 128.2, 126.9, 126.1, 125.0, 123.2, 114.0.

3.3 Synthesis of 1ab

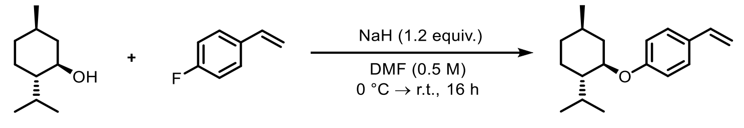

Compound **1ab** was synthesized adapting a procedure reported in the literature.[5] An oven-dried Schlenk tube was charged with sodium hydride (240.0 mg as 60% w/w dispersion in mineral oil, 6.0 mmol, 1.2 equiv.). After sealing the tube, it was evacuated and back-filled with N_2 three times. Then, a solution of (1*R*,2*S*,5*R*)-2-isopropyl-5-methylcyclohexan-1-ol (860.0 mg, 5.5 mmol, 1.1 equiv.) in dry DMF (10 mL) was added via syringe under N_2 atmosphere at 0 °C. The reaction was stirred at r.t. for 15 min. 1-Fluoro-4-vinylbenzene (0.55 mL, 5.0 mmol, 1.0 equiv.) was subsequently added, and the reaction mixture was stirred at r.t. for 16 hours. Upon completion, the reaction was quenched by the addition of sat. aq. NH_4Cl (10 mL). The aqueous layer was extracted with EtOAc (3 x 10 mL), the combined organic phase was washed with sat. aq. NaCl (10 mL) and dried over anhydrous Na_2SO_4. The crude reaction mixture was then concentrated *in vacuo* and purified via flash column chromatography on silica gel (Pentane) to afford **1ab** as a colourless oil (167.0 mg, 13% yield). Characterization data are in accordance with the literature.[5]

^{1}H NMR (300 MHz, $CDCl_3$) δ 7.33 (d, *J* = 8.7 Hz, 2H), 6.86 (d, *J* = 8.7 Hz, 2H), 6.66 (dd, *J* = 17.6, 10.9 Hz, 1H), 5.60 (dd, *J* = 17.6, 1.0 Hz, 1H), 5.11 (dd, *J* = 10.9, 1.0 Hz, 1H), 4.03 (td, *J* = 10.5, 4.1 Hz, 1H), 2.25 – 2.11 (m, 2H), 1.80 – 1.68 (m, 2H), 1.59 – 1.43 (m, 2H), 1.15 – 0.99 (m, 3H), 0.93 (dd, *J* = 6.8, 2.5 Hz, 6H), 0.77 (d, *J* = 7.0 Hz, 3H).
^{13}C NMR (75 MHz, $CDCl_3$) δ 158.4, 136.4, 130.3, 127.6, 115.9, 111.5, 77.7, 48.2, 40.5, 34.7, 31.6, 26.2, 23.9, 22.3, 20.9, 16.7.

3.4 Synthesis of 1ac

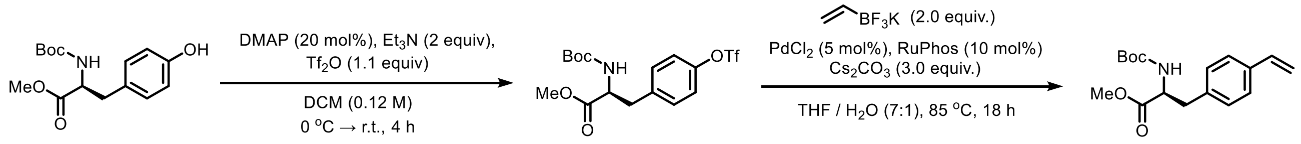

Compound **1ac** was synthesized adapting a procedure reported in the literature.[6] A pressure tube was charged with methyl (*tert*-butoxycarbonyl)-*L*-tyrosinate (738 mg, 2.5 mmol, 1.0 equiv.) and DMAP (61 mg, 0.5 mmol, 20 mol%). After sealing the tube, it was evacuated and back-filled with N_2 three times. Dry DCM (20 mL) was added and the mixture was cooled down to 0 ºC. Afterwards, triethylamine (0.70 mL, 5.0 mmol, 2.0 equiv.) and trifluoromethanesulfonic anhydride (0.46 mL, 2.8 mmol, 1.1 equiv.) were added dropwise. The resulting pink mixture was allowed to warm up to r.t. and further stirred for 4 h. Upon completion, sat. aq. $NaHCO_3$ (20 mL) was added, the aqueous phase was extracted with DCM (3 x 15 mL) and the combined organic phase was dried over anhydrous Na_2SO_4. Evaporation of the solvent afforded methyl (*S*)-2-((*tert*-butoxycarbonyl)amino)-3-(4-(((trifluoromethyl)sulfonyl)oxy)phenyl)propanoate, that was used without further purification. For the second step, an oven-dried pressure tube was charged with the crude product (855.0 mg, 2.0 mmol, 1.0 equiv.), potassium vinyltrifluoroborate (536.0 mg, 4.0 mmol, 2.0 equiv.), $PdCl_2$ (18.0 mg, 0.1 mmol, 5

mol%), RuPhos (93.0 mg, 0.2 mmol, 10 mol%) and Cs_2CO_3 (2.0 g, 6.0 mmol, 3 equiv.). After sealing the tube, it was evacuated and back-filled with N_2 three times. Dry THF (5 mL) and distilled water (0.75 mL) were added and the resulting mixture was stirred at 85 °C for 18 h. Upon completion, water (10 mL) was added, the aqueous phase was extracted with DCM (3 x 10 mL) and the combined organic phase was dried over anhydrous Na_2SO_4. The crude reaction mixture was then concentrated *in vacuo* and resulting residue purified via flash column chromatography on silica gel (Pentane:EtOAc 20:1) to afford **1ac** as a colorless oil (450.0 mg, 51% yield over 2 steps). Characterization data are in accordance with the literature.[6]

^{1}H NMR (300 MHz, $CDCl_3$) δ 7.33 (d, *J* = 8.1 Hz, 2H), 7.08 (d, *J* = 8.0 Hz, 2H), 6.68 (dd, *J* = 17.6, 10.9 Hz, 1H), 5.72 (dd, *J* = 17.6, 1.0 Hz, 1H), 5.22 (dd, *J* = 10.9, 1.0 Hz, 1H), 4.97 (d, *J* = 8.4 Hz, 1H), 4.58 (q, *J* = 6.5 Hz, 1H), 3.71 (s, 3H), 3.17 – 2.97 (m, 2H), 1.42 (s, 9H).
^{13}C NMR (75 MHz, $CDCl_3$) δ 172.4, 155.2, 136.6, 136.5, 135.7, 129.6, 126.5, 113.8, 80.1, 54.5, 52.4, 38.2, 28.4.

3.5 Synthesis of 2g

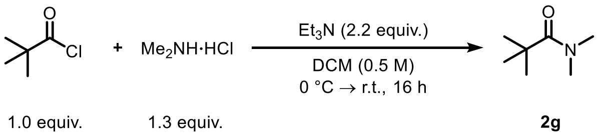

Compound **2g** was synthesized adapting a procedure reported in the literature.[7] To a solution of dimethylamine hydrochloride (1.9 g, 23 mmol, 1.3 equiv.) and triethylamine (5.5 mL, 40 mmol, 2.2 equiv.) in dichloromethane (36 mL), pivaloyl chloride (2.2 mL, 18 mmol, 1.0 equiv.) was added in one portion at 0 °C. The resulting mixture was stirred at r.t. for 16 h. Upon completion, pentane / EtOAc 1:1 (50 mL) was added and the precipitate was filtered over silica. The filtrate was concentrated *in vacuo* to afford **2g** as a pale yellow oil (1.8 g, 78% yield). Characterization data are in accordance with the literature.[8]

^{1}H NMR (400 MHz, $CDCl_3$) δ 2.99 (s, 6H), 1.24 (s, 9H).
^{13}C NMR (75 MHz, $CDCl_3$) δ 177.6, 38.7, 38.3, 28.3.

4. Photoreaction setup

4-8 vials photoreactor (UFO reactor)

Four reactions were irradiated simultaneously using the photoreactor shown below (**Figure S3**). A 52 W Kessil PR160L-390 nm or 40 W Kessil PR160L-456 nm was used as LED lamp, while the temperature was maintained around 30–35 °C via a fan positioned underneath the reactor.[9] The assembled setup was placed behind a UV-light shielding amber acrylic panel for the entire duration of the reaction.

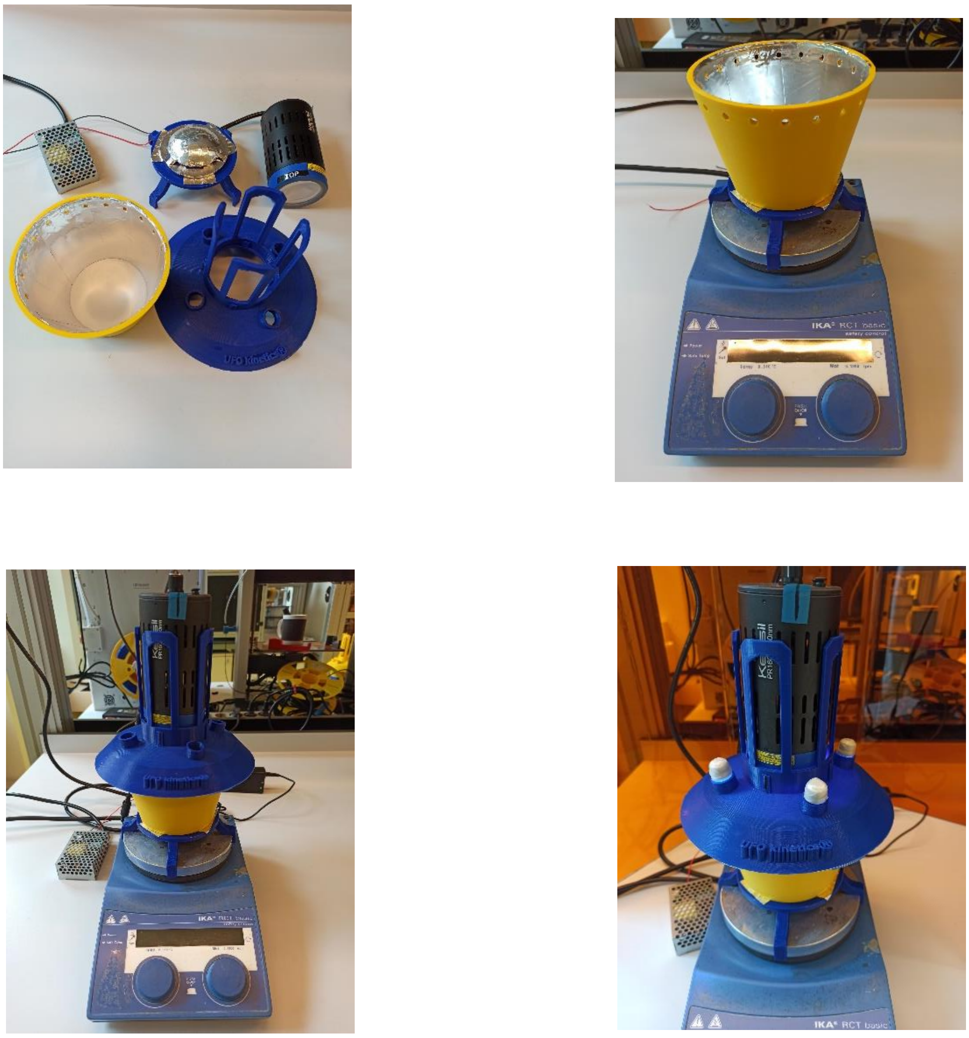

Figure S3: Photoreactor used for the optimization and substrate scope.

4 vials photoreactor (1 mmol scale)

A 30 mL vial equipped with a stirring bar and charged with the reaction mixture was irradiated using the photoreactor shown below (**Figure S4**).[10] A 52 W Kessil PR160L-390 nm LED was used as the light source, while the temperature was maintained around 30–35 °C via a fan positioned underneath the reactor. The assembled setup was placed behind a UV-light shielding amber acrylic panel for the entire duration of the reaction.

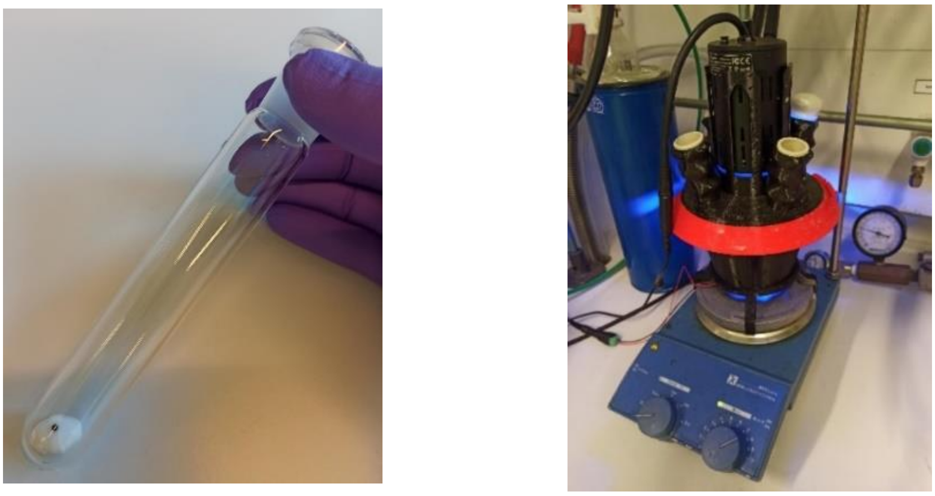

Figure S4: Photoreactor setup for 1.0 mmol scale reaction.

5. Optimization of the photocatalyzed alkene oxo-amidomethylation

Table S1: Oxygen loading screening.

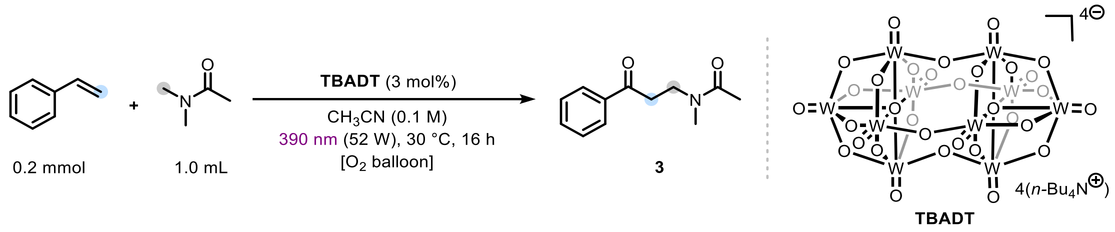

Entry	Deviations	Yield of 3 (%)[a]
1	None[b]	36
2	Sparging O_2 for 5 min	54
3	Sparging O_2 for 5 min, + 1 M HCl (0.4 mL)	75
4	Air instead of O_2	15
5	No O_2 balloon[c]	5

[a] Determined via ^{1}H NMR using 1,1,2-trichloroethylene as an internal standard.
[b] The vial was fitted with an oxygen balloon attached to a syringe needle.
[c] Reaction mixture prepared and sealed under air.

Table S2: Reaction components ratio screening.

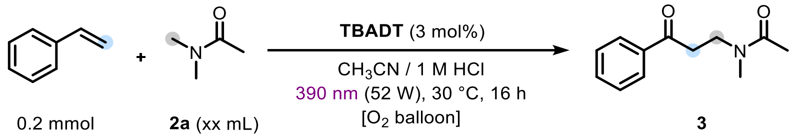

Entry	CH_3CN (mL)	2a (mL)	HCl 1 M (mL)	Yield of 3 (%)[a]
1	1.0	1.0	0.4	75
2	2.0	1.0	-	57
3	1.0	0.5	0.4	50 (61)[b]
4	0.5	1.0	0.4	70
5	0.5	0.5	0.4	60
6	3.0	0.5	0.4	30
7	0.5	0.5	1.0	35
8	0.25	0.5	0.4	61

[a] Determined via ^{1}H NMR using 1,1,2-trichloroethylene as an internal standard.
[b] 40 h irradiation time.

Table S3: DMA loading screening.

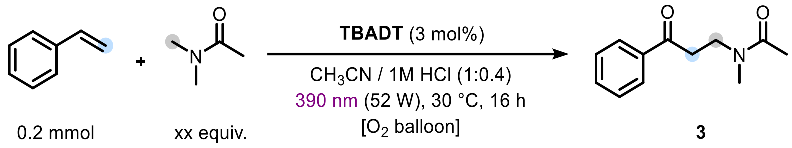

Entry	DMA loading	Yield of 3 (%)[a]
1	54 equiv. (1 mL)	75
2	27 equiv. (0.5 mL)	50
3	10 equiv. (0.2 mL)	15

[a] Determined via ^{1}H NMR using 1,1,2-trichloroethylene as an internal standard.

Table S4: Catalyst screening.

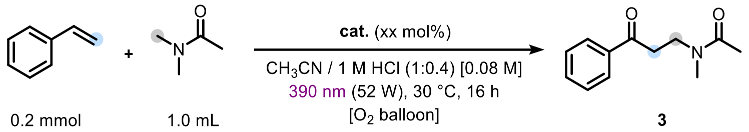

Entry	cat. (xx mol%)	Yield of 3 (%)[a]
1	**TBADT** (3)	75
2	**AQMS** (10)	50
3	**AQDS** (10)	50
4	**AQ** (10)	49
5	**4Cl$_2$-BP** (20)	25
6	$FeCl_3$ (15)	18
7	**EosinY** (10)[b]	n.d.

[a] Determined via ^{1}H NMR using 1,1,2-trichloroethylene as an internal standard.
[b] A 40 W Kessil PR160L-456 nm LED was used.

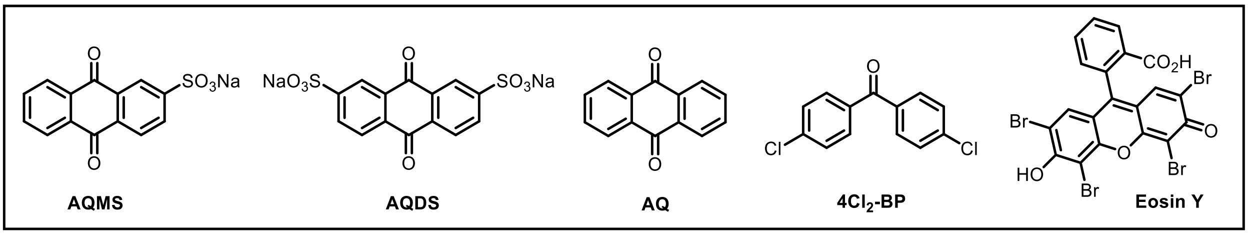

Table S5: Additive screening.

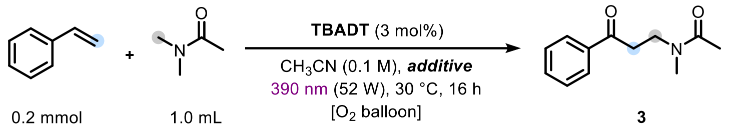

Entry	Additive	Yield of 3 (%)[a]
1	1 M HCl (0.4 mL)	75
2	H_2O (0.4 mL)	30
3	1 M Na_3PO_4 (0.4 mL)	13
4	1 M TFA (0.4 mL)	68
5	*n*-$Bu_4N{\cdot}Cl$ (2 equiv.)	<5

[a] Determined via ^1H NMR using 1,1,2-trichloroethylene as an internal standard.

Table S6: Acid screening.

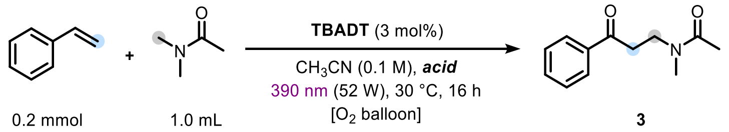

Entry	Acid	Yield of 3 (%)[a]
1	1 M HCl (0.4 mL)	75
2	2 M HCl (0.4 mL)	65
3	3 M HCl (0.4 mL)	64
4	conc. HCl (0.3 mL)	58
5	conc. HCl (33.0 μL)	60

[a] Determined via ^1H NMR using 1,1,2-trichloroethylene as an internal standard.

Table S7: TBADT loading screening.

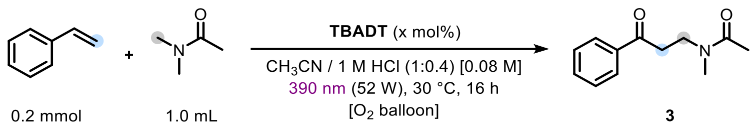

Entry	TBADT loading	Yield of 3 (%)[a]
1	3 mol%	75
2	2 mol%	70
3	1 mol%	67
4	-	n.d.

[a] Determined via ^{1}H NMR using 1,1,2-trichloroethylene as an internal standard.

Table S8: Effect of acid addition on reaction yield.

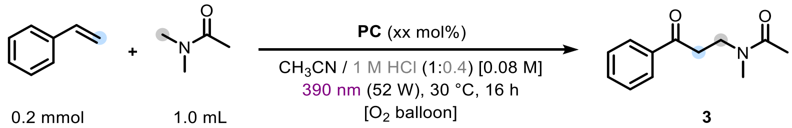

Entry	PC (xx mol%)	1 M HCl Added	Yield of 3 (%)[a]
1	**TBADT** (3)	yes	75
2	**TBADT** (3)	no	54
3	**AQDS** (10)	yes	50
4	**AQDS** (10)	no[b]	21
5	**4Cl$_2$-BP** (20)	yes	25
6	**4Cl$_2$-BP** (20)	no	15

[a] Determined via ^{1}H NMR using 1,1,2-trichloroethylene as an internal standard.
[b] The solubility of **AQDS** in organic solvents was noticeably enhanced by the addition of acid and small amounts of water.

6. General Procedure (GP2): Photocatalyzed alkene oxo-amidomethylation

In a typical experiment: to a 7 mL vial equipped with a stirring bar were added aryl olefin (0.3 mmol, 1.0 equiv., *if solid*), **TBADT** (30.0 mg, 3 mol%), dry CH_3CN (1.5 mL), amide (1.5 mL) and 1 M aqueous HCl (0.6 mL). After sealing the vial, the solution was sparged with an oxygen balloon for 10 min. During this process, the vial was cooled to 0 °C to minimize solvent evaporation. Afterwards, aryl olefin (0.30 mmol, 1.0 equiv., *if liquid*) was added via syringe. The vial was fitted with an oxygen balloon attached to a syringe needle. The reaction mixture was stirred and irradiated (52 W Kessil PR160L-390 nm LED, 100% light intensity) in the photochemical reactor (see **Section 4**) for 16 h. The temperature was maintained around 30 °C during the course of the reaction. Then, the vial was removed from the photochemical reactor and the mixture was added to a separatory funnel containing sat. aq. $NaHCO_3$ solution (10 mL) and EtOAc (10 mL). The aqueous layer was extracted with EtOAc (3 x 10 mL) and the combined organic phase was washed with sat. aq. NaCl (3 x 10 mL), water (2 x 10 mL) and dried over anhydrous Na_2SO_4. The crude reaction mixture was then concentrated *in vacuo* and purified via flash column chromatography on silica gel. See **Section 11** for characterization data.

7. Mechanistic investigation

7.1 Quenching experiments

Table S9: quenching experiments.

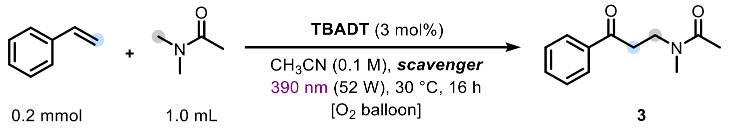

Entry	Scavenger (x equiv.) *[quenched species]*	1 M HCl	Yield of 3 (%)[a]
1	-	yes	75
2	-	no	54
3	$P(OEt)_3$ (3) *[hydroperoxide]*	yes	39
4	^{t}BuOH (3) *[hydroxyl radical]*	yes	73
5	DABCO (3) *[singlet oxygen]*	no	32
6	NaN_3 (3) *[singlet oxygen]*	no	19

[a] Determined via ^{1}H NMR using 1,1,2-trichloroethylene as an internal standard.

As shown in **Table S9**, the reaction is inhibited in the presence of triethyl phosphite (Entry 3), a well-established quencher of hydroperoxides. This observation supports the proposed involvement of a benzylic hydroperoxidic intermediate, in line with literature precedent for radical processes involving triplet oxygen ($^3O_2$).[11,12] Additional quenchers were evaluated based on reported mechanistic studies.[13,14] In particular, the use of ^{t}BuOH, a scavenger of hydroxyl radicals, had no effect on the reaction outcome (Entry 4). In contrast, the addition of DABCO and NaN_3 led to a decrease in product yield (Entries 5-6). However, these results are not conclusive regarding the involvement of singlet oxygen ($^1O_2$) as: *(i)* in the presence of these additives, the reaction mixtures remained colorless or developed only a faint blue color after 4-5 hours of irradiation, whereas under the standard conditions a deep blue color appeared within 30 seconds, consistent with the rapid formation of the **TBADT**−H species. A similar immediate color change was observed in Entries 3 and 4. This suggests that under conditions of Entries 5 and 6 the first HAT step is slowed down.

(ii) DABCO ($E_{1/2}$ = +0.69 V *vs* SCE)[15] or the azide anion ($E_{1/2}$ = +0.87 V *vs* SCE)[16] may undergo oxidation to DABCO radical cation or azide radical by ***TBADT** (*E^0 = +2.44 *vs* SCE),[17] potentially interfering with the productive catalytic cycle through undesired quenching pathways.

7.2 Radical trapping and inhibition experiment

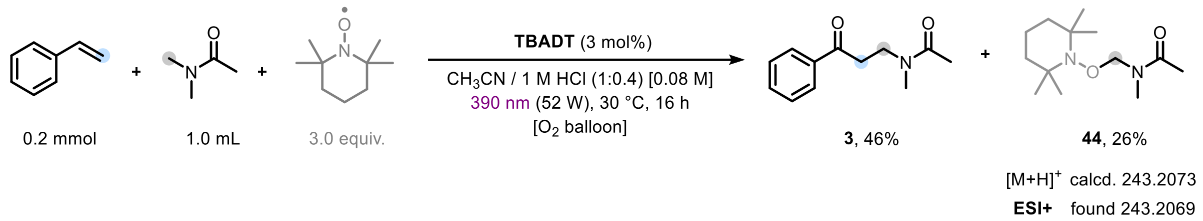

A 7 mL vial equipped with a stirring bar was charged with 2,2,6,6-tetramethyl-1-piperidinyloxyl (TEMPO, 94.0 mg, 0.60 mmol, 3.0 equiv.), **TBADT** (20.0 mg, 3 mol%), dry CH_3CN (1.0 mL), dry DMA (1.0 mL) and 1 M aqueous HCl (0.4 mL). After sealing the vial, the solution was sparged with an oxygen balloon for 5 min. During this process, the vial was cooled to 0 °C to minimize solvent evaporation. Afterwards, styrene (23.0 µL, 0.20 mmol, 1.0 equiv.) was added via syringe. The vial was fitted with an oxygen balloon attached to a syringe needle. The reaction mixture was stirred and irradiated (52 W Kessil PR160L-390 nm LED, 100% light intensity) in the photochemical reactor for 16 h. The temperature was maintained around 30 °C during the course of the reaction. Then, the vial was removed from the photochemical reactor and the mixture was added to a separatory funnel containing sat. aq. $NaHCO_3$ solution (10 mL) and EtOAc (10 mL). The aqueous layer was extracted with EtOAc (3 x 10 mL) and the combined organic phase was washed with sat. aqueous NaCl (3 x 10 mL), water (2 x 10 mL) and dried over anhydrous Na_2SO_4. The solvent was removed *in vacuo* and the crude reaction mixture was analyzed via ^{1}H NMR using 1,1,2-trichloroethylene (1.0 equiv.) as an internal standard, GC-MS and HR-MS (ESI+).

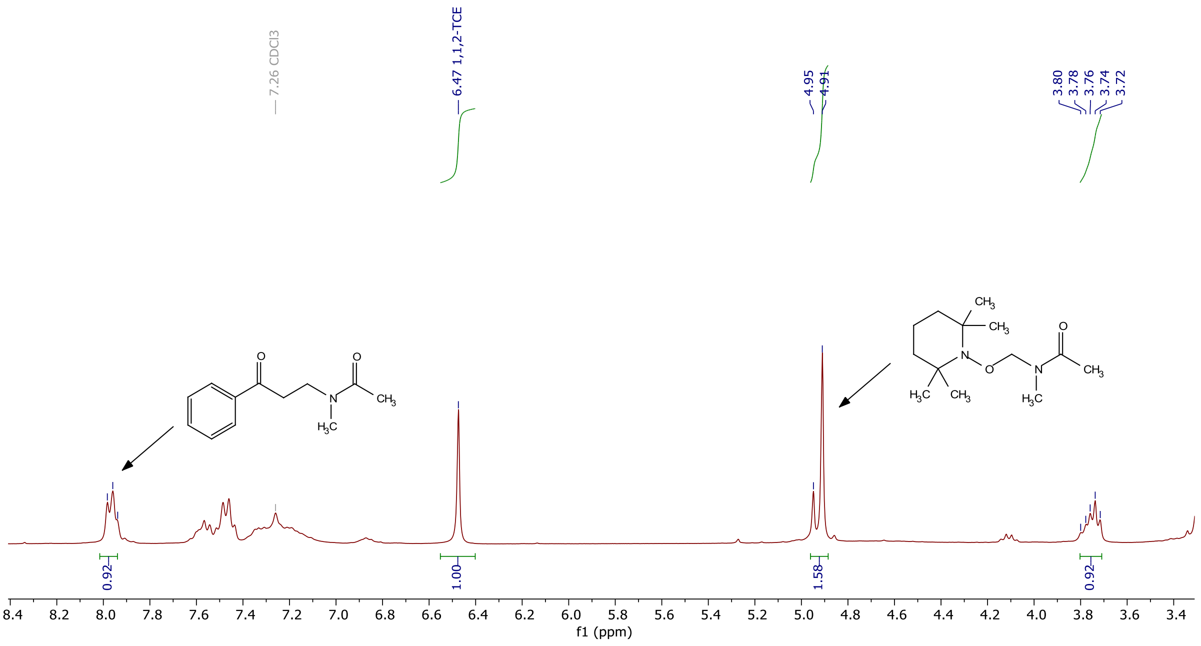

Figure S5: ^{1}H NMR of the crude reaction mixture in the presence of TEMPO (3.0 equiv.).

As shown in **Figure S5**, the reaction is inhibited in the presence of TEMPO, resulting in a 46% yield of target product **3**. Moreover, the TEMPO adduct **44** with the *α*-amido radical derived from DMA was observed in 26% yield, with ^{1}H NMR chemical shifts in accordance with the literature.[18]

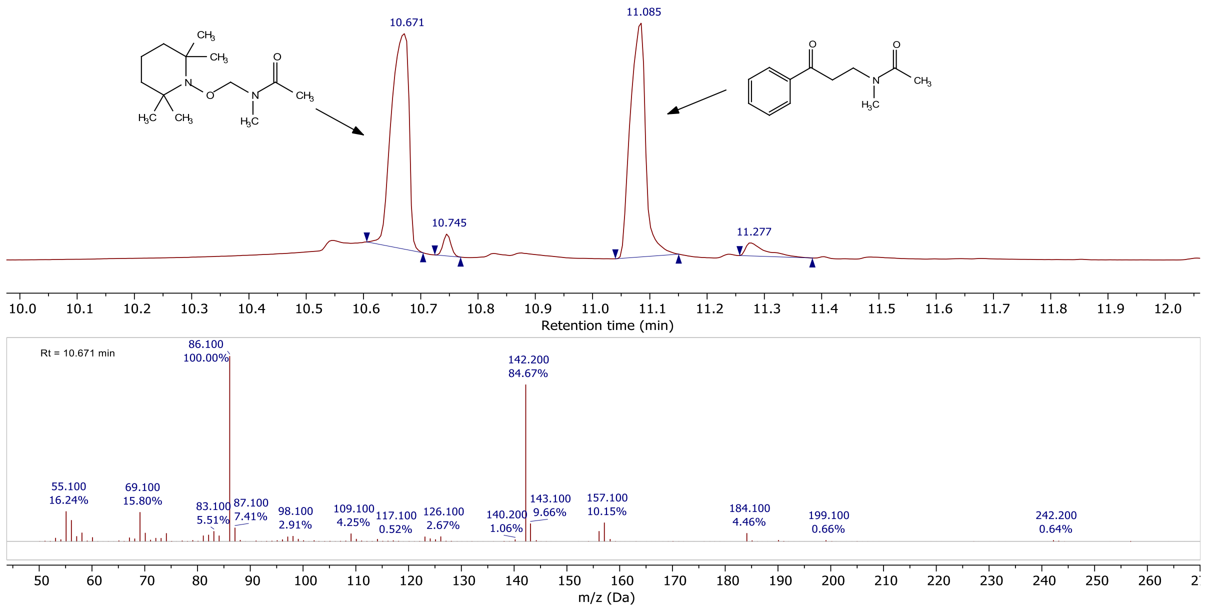

Figure S6: GC-MS chromatogram of a sample from reaction with TEMPO *(top)* and electron ionization (EI+) mass spectrum of the TEMPO adduct *(bottom)*.

Furthermore, examination of the crude reaction mixture via GC-MS (**Figure S6**) showed a peak with a mass spectrum and fragmentation pattern matching the literature data for *N*-methyl-*N*-(((2,2,6,6-tetramethylpiperidin-1-yl)oxy)methyl)acetamide.[19] HR-MS (ESI+) analysis is shown in **Figure S7**.

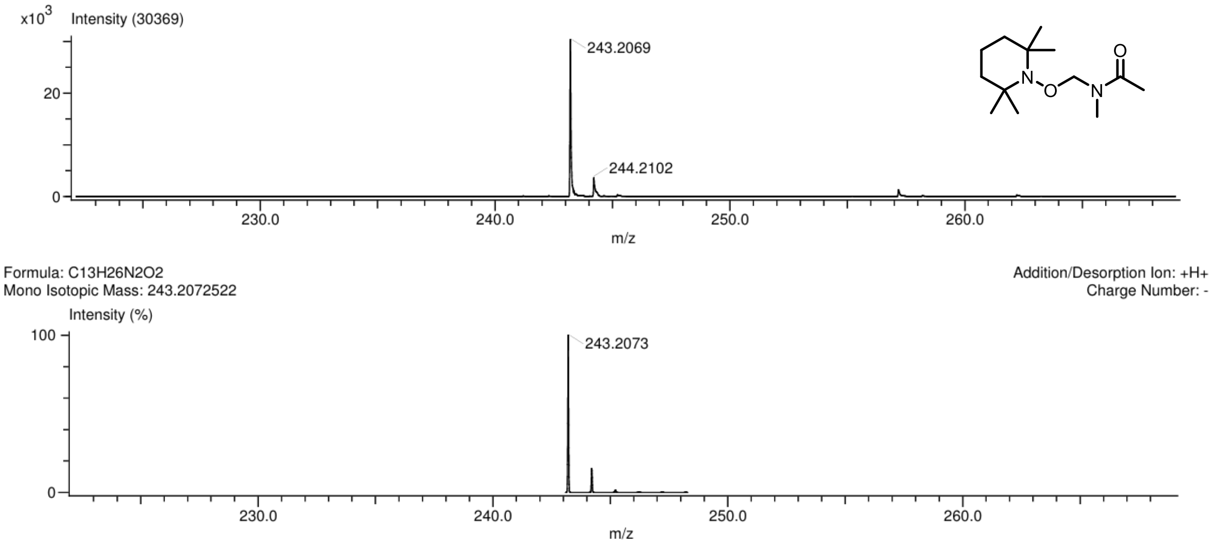

Figure S7: HR-MS of the TEMPO adduct.

7.3 Reactivity of *α*-methylstyrene

A 7 mL vial equipped with a stirring bar was charged with **TBADT** (20.0 mg, 3 mol%), dry CH_3CN (1.0 mL), dry DMA (1.0 mL) and 1 M aq. HCl (0.4 mL). After sealing the vial, the solution was sparged with an oxygen balloon for 5 min. During this process, the vial was cooled to 0 °C to minimize solvent evaporation. Afterwards, *α*-methylstyrene (26.0 µL, 0.20 mmol, 1.0 equiv.) was added via syringe. The vial was fitted with an oxygen balloon attached to a syringe needle. The reaction mixture was stirred and irradiated (52 W Kessil PR160L-390 nm LED, 100% light intensity) in the photochemical reactor for 16 h. The temperature was maintained around 30 °C during the course of the reaction. The vial was removed from the photochemical reactor, transferred to a round-bottomed flask, and EtOAc (15 mL) was added. The resulting solution was dried over anhydrous Na_2SO_4 and the solvent was then removed *in vacuo*. The crude reaction mixture was analyzed via ^{1}H NMR using 1,1,2-trichloroethylene (1.0 equiv.) as an internal standard and was also analyzed by GC-MS.

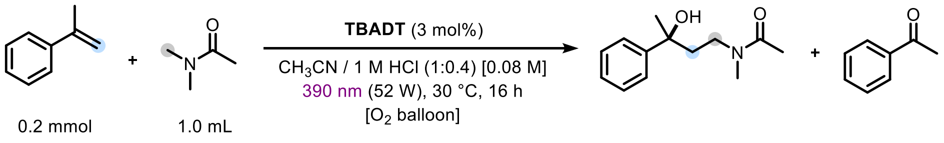

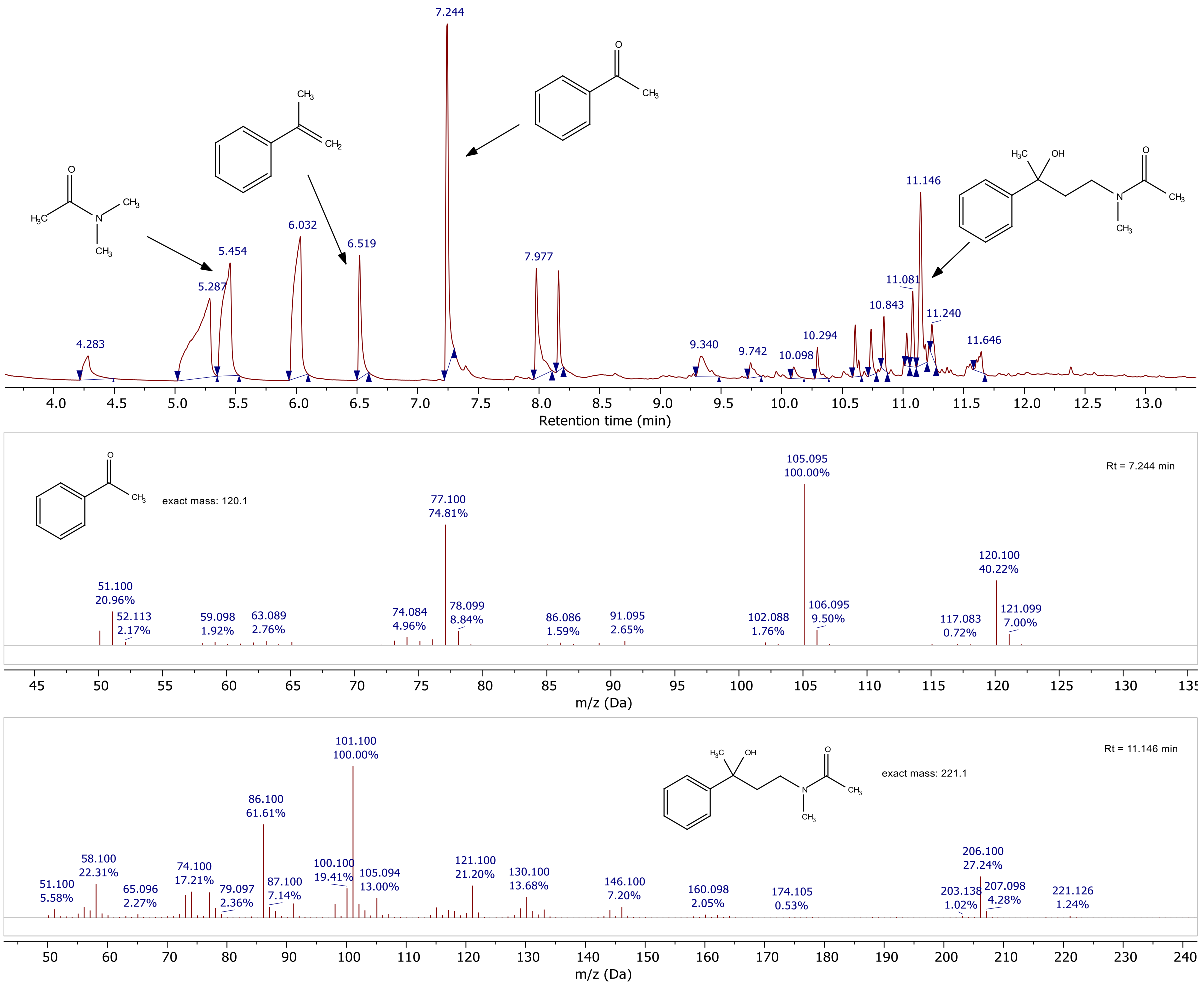

Figure S8: GC-MS chromatogram of a sample from reaction with *α*-methylstyrene and DMA *(top)*; electron ionization (EI+) mass spectra of acetophenone and *N*-(3-hydroxy-3-phenylbutyl)-*N*-methylacetamide *(bottom)*.

^{1}H NMR analysis revealed the formation of acetophenone in 45% yield. This result is consistent with the propensity of *α*-substituted styrenes to undergo direct photocatalyzed oxidative cleavage in the presence of **TBADT** and O_2 (or air), as reported in the works of Xie, Ryu and co-workers.[13,14] The formation of acetophenone as the main product is further supported by the GC trace, showing a peak at 7.2 min with a mass spectrum and fragmentation pattern matching literature data.[20] In contrast, the corresponding oxidative cleavage of unbranched styrenes to their corresponding aldehydes were not observed by ^{1}H NMR or GC-MS, as also shown in **Figure S9** for the reactions of styrene with DMA and 4-chlorostyrene with 1-methylpyrrolidin-2-one (NMP), respectively.

Moreover, the GC-MS trace in **Figure S8** showed a peak at 11.1 min with a mass spectrum consistent with the target oxy-amidomethylation product. However, quantification by ^{1}H NMR was not possible due to the absence of an unobstructed aliphatic peak in the crude mixture. The formation of the alcohol is envisioned to occur through reduction of the hydroperoxide intermediate.[21] In particular, the single electron transfer (SET) process could be mediated by the reduced form of $[W_{10}O_{32}]^{4-}$ (**TBADT**), $H^+[W_{10}O_{32}]^{5-}$ (E_{red} = −0.97 V *vs* SCE, **TBADT**−H),[22] or by the doubly reduced form, $2H^+[W_{10}O_{32}]^{6-}$ (E_{red} = −1.48 V *vs* SCE),[23–25] which can be generated in the reaction medium via disproportionation of $H^+[W_{10}O_{32}]^{5-}$, with concomitant regeneration of $[W_{10}O_{32}]^{4-}$.

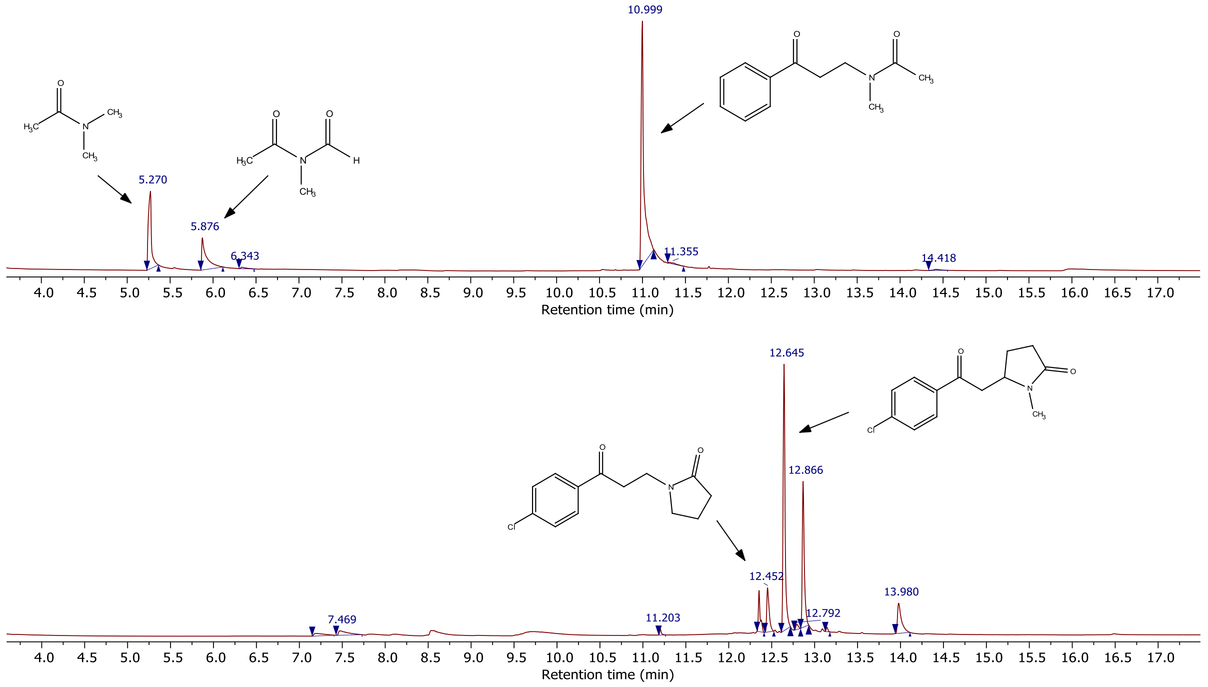

Figure S9: GC-MS chromatogram of a sample from oxo-amidomethylation reaction with styrene and DMA *(top);* GC-MS chromatogram of a sample from oxo-amidomethylation reaction with 4-chlorostyrene and NMP *(bottom)*.

With these results in hand, we questioned the origin of the byproduct formation observed in *α*-branched vinyl arenes. Since oxidative cleavage of C=C bonds has been reported in the literature to proceed via singlet oxygen formation,[13] a trapping experiment was carried out using 9,10-dimethylanthracene. The reaction mixture was stirred and irradiated (52 W Kessil PR160L-390 nm LED, 100% light intensity) in the photochemical reactor for 16 h. The temperature was maintained around 30 °C during the course of the reaction. The vial was removed from the photochemical reactor, transferred

to a round-bottom flask, and the solvent was then removed carefully *in vacuo*. The crude reaction mixture was analyzed via ^{1}H NMR using 1,1,2-trichloroethylene (1.0 equiv.) as an internal standard. The characteristic signals corresponding to 9,10-dimethyl-9,10-dihydro-9,10-epidioxyanthracene were detected in 38% yield, consistent with literature data,[26] thus confirming the generation of singlet oxygen under the reaction conditions (**Figure S10**). The formation of the substituted ketone can be rationalized by a [2+2] cycloaddition of singlet oxygen with the alkene, followed by decomposition of the resulting dioxetane intermediate to afford the corresponding carbonyl compound with concomitant release of formaldehyde.[27]

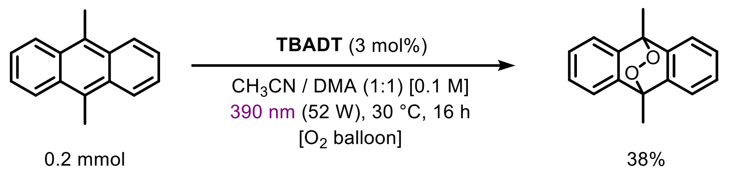

Figure S10: Singlet Oxygen ($^1O_2$) trapping experiment with 9,10-dimethylanthracene.

Finally, while benzaldehyde was never detected when **TBADT** was employed as the photocatalyst with unbranched styrenes, the use of **AQDS** under otherwise identical conditions led to the formation of benzaldehyde in approximately 11% yield in the crude reaction mixture (**Table S10**). This observation can be explained by a slower hydrogen atom transfer (HAT) step in the case of **AQDS**, giving way to accumulation of $^1O_2$ and thus promoting competitive oxidative C=C bond cleavage.

Table S10: Effect of the photocatalyst on benzaldehyde formation.

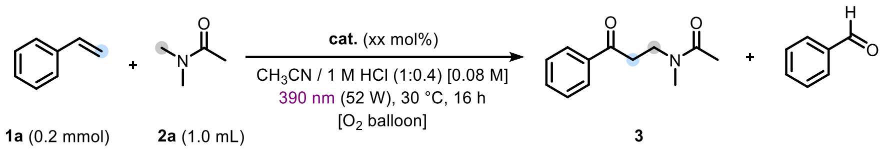

Entry	cat. (xx mol%)	Yield of 3 (%)[a]	Yield of benzaldehyde (%)[a]
1	**TBADT** (3)	75	n.d.
2	**AQMS** (10)	50	2
3	**AQDS** (10)	50	11
4	**AQ** (10)	49	2
5	**4Cl$_2$-BP** (20)	25	n.d.
6	$FeCl_3$ (15)	18	n.d.
7	**EosinY** (10)	n.d.	n.d.

[a] Determined via ^{1}H NMR using 1,1,2-trichloroethylene as an internal standard.
[b] A 40 W Kessil PR160L-456 nm LED was used.

7.4 Reaction kinetics

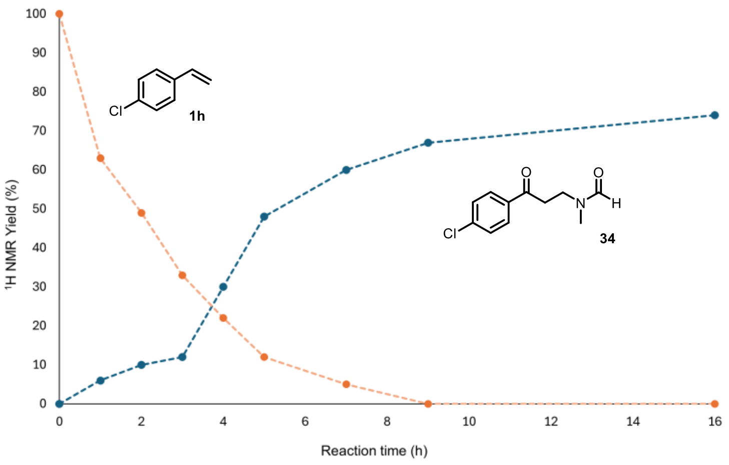

Time	Conversion of 1h (%)[a]	Yield of 34 (%)[a]
1 h	37	6
2 h	51	10
3 h	67	12
4 h	78	30
5 h	88	48
7 h	95	60
9 h	100	67
16 h	100	74

[a] 1,1,2-trichloroethylene as an internal standard.

Figure S11: Kinetic profile of oxo-amidomethylation reaction of **1h** and **2b**.

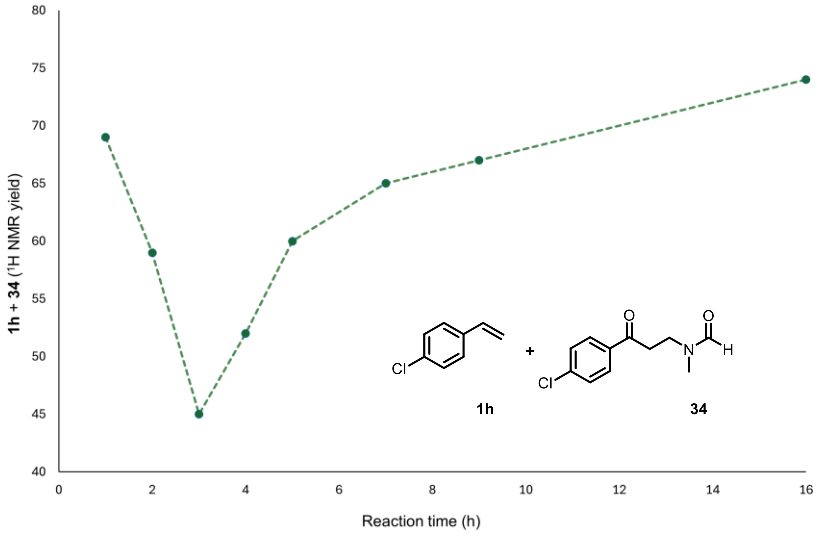

Time	1h + 34 (%)
1 h	69
2 h	59
3 h	45
4 h	52
5 h	60
7 h	65
9 h	67
16 h	74

Figure S12: Temporary evolution of combined starting material (**1h**) and product (**34**) ^{1}H NMR yields.

7.5 Kinetic isotope effect (KIE)

Parallel experiments

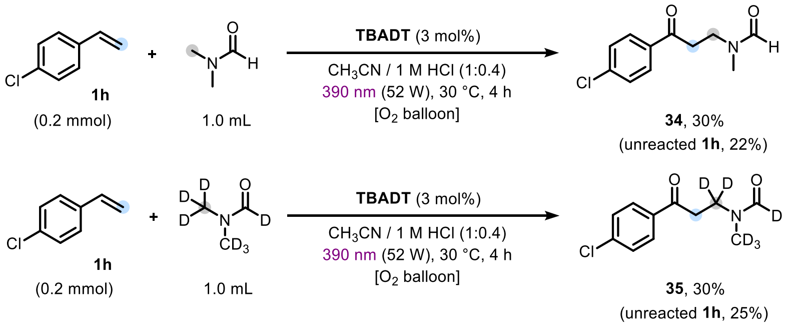

Figure S13: KIE parallel experiments.

Competition experiment

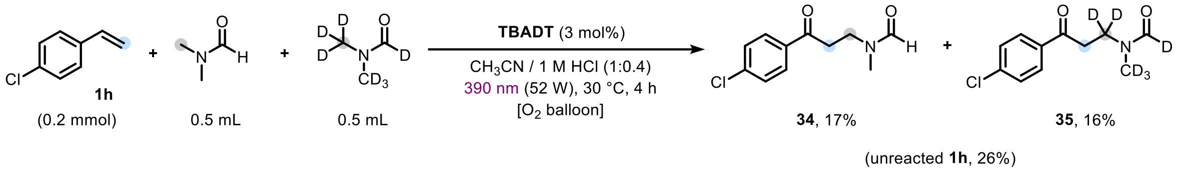

Figure S14: KIE competition experiment.

A 7 mL vial equipped with a stirring bar was charged with **TBADT** (20.0 mg, 3 mol%), dry CH_3CN (1.0 mL), dry DMF-h_7 (1.0 or 0.5 mL) and/or DMF-d_7 (1.0 mL or 0.5 mL) as specified in **Figure S13** and **Figure S14** and 1 M aq. HCl (0.4 mL). After sealing the vial, the solution was sparged with an oxygen balloon for 5 min. During this process, the vial was cooled to 0 °C to minimize solvent evaporation. Afterwards, 4-chlorostyrene (24.0 µL, 0.2 mmol, 1.0 equiv.) was added via syringe. The vial was fitted with an oxygen balloon attached to a syringe needle. The reaction mixture was stirred and irradiated (52 W Kessil PR160L-390 nm LED, 100% light intensity) in the photochemical reactor for 4 h. The temperature was maintained around 30 °C during the course of the reaction. Then, the vial was removed from the photochemical reactor and the mixture was added to a separatory funnel containing sat. aq. $NaHCO_3$ solution (10 mL) and EtOAc (10 mL). The aqueous layer was extracted with EtOAc (3 x 10 mL) and the combined organic phase was washed with sat. aq. NaCl (3 x 10 mL), water (2 x 10 mL) and dried over anhydrous Na_2SO_4. The solvent was then removed *in vacuo* and the crude reaction mixture was analyzed via ^{1}H NMR using 1,1,2-trichloroethylene (1.0 equiv.) as an internal standard.

In all these experiments, care was taken to run the reaction for a shorter (4 h) reaction time to ensure non-completion. Kinetic isotope effects (KIEs) were consistent between parallel experiments (1.0) and the competition experiment (1.1) involving DMF-h_7 *vs* DMF-d_7. This indicates that hydrogen atom transfer (HAT) is not the rate-determining step, or that the photocatalyst being already in such a high energy excited state (a photon of 390 nm absorbed by **TBADT** corresponds to an input energy of 73.3 kcal mol^{-1}) does not discriminate well between the small difference in C−H and C−D bond strengths (~1.2 kcal mol^{-1}).[28]

8. Scale-up procedures

1 mmol scale

A 30 mL vial equipped with a stirring bar was charged with **TBADT** (0.1 g, 3 mol%), dry CH_3CN (5 mL), **2b** (5.0 mL) and 1 M aq. HCl (2.0 mL). After sealing the vial with a rubber septum, the solution was sparged with an oxygen balloon for 25 min. During this process, the vial was cooled to 0 °C to minimize solvent evaporation. Afterwards, **1h** (0.12 mL, 1.0 mmol, 1.0 equiv.) was added via syringe. The vial was fitted with an oxygen balloon attached to a syringe needle. The reaction mixture was stirred and irradiated (52 W Kessil PR160L-390 nm, 100% light intensity) in the photochemical reactor (**Figure S15**) for 16 h. The temperature was maintained around 30 °C during the course of the reaction. Then, the vial was removed from the photochemical reactor and the mixture was added to a separatory funnel containing sat. aq. $NaHCO_3$ solution (25 mL) and EtOAc (25 mL). The aqueous layer was extracted with EtOAc (3 x 25 mL) and the combined organic phase was washed with sat. aq. NaCl (3 x 25 mL), water (2 x 25 mL) and dried over anhydrous Na_2SO_4. The crude reaction mixture was then concentrated *in vacuo* and purified via flash column chromatography on silica gel (gradient from Pentane:EtOAc 1:1 to 100% EtOAc) to afford **34** as a white solid (181.0 mg, 80% yield).

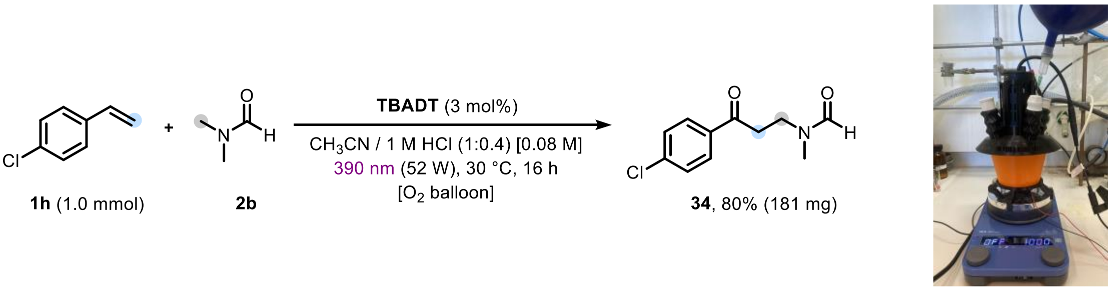

Figure S15: Reaction setup for a 1.0 mmol scale reaction.

5 mmol scale

A 250 mL Schlenk tube equipped with a stirring bar was charged with **TBADT** (0.5 g, 3 mol%), dry CH_3CN (25.0 mL), **2b** (25.0 mL) and 1 M aqueous HCl (10.0 mL). After sealing the tube with a rubber septum, the solution was sparged with an oxygen balloon for 45 min. During this process, the mixture was cooled to 0 °C to minimize solvent evaporation. Afterwards, **1h** (0.6 mL, 5.0 mmol, 1.0 equiv.) was added via syringe. The tube was fitted with an oxygen balloon attached to a syringe needle. The reaction mixture was stirred and irradiated using two 52 W Kessil PR160L-390 nm LEDs (100% light intensity, one with a linear reflector aligned horizontally and one aligned vertically to the tube) for 16 h as shown in **Figure S16**. A high-power CPU fan was used to maintain the temperature at 30 °C during the course of the reaction. Then, the Schlenk tube was removed from the photochemical reactor and the mixture was added to a separatory funnel containing sat. aq. $NaHCO_3$ solution (50 mL) and EtOAc (50 mL). The aqueous layer was extracted with EtOAc (3 x 50 mL) and the combined organic phase was washed with sat. aq. NaCl (3 x 50 mL), water (2 x 50 mL) and dried over anhydrous Na_2SO_4. The crude reaction mixture was then concentrated *in vacuo* and purified via flash column chromatography on silica gel (gradient from Pentane:EtOAc 1:1 to 100% EtOAc) to afford **34** as a white solid (580.0 mg, 51% yield).

Extension of the irradiation time from 16 h to 40 h enabled full conversion of **1h** (as monitored by TLC), affording **34** in 64% yield (720.0 mg) after flash column chromatography.

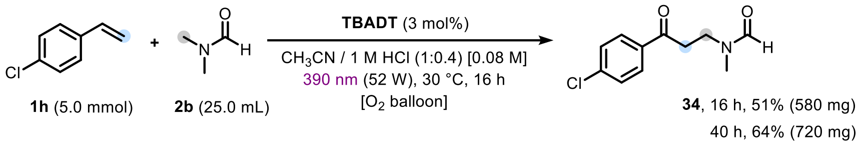

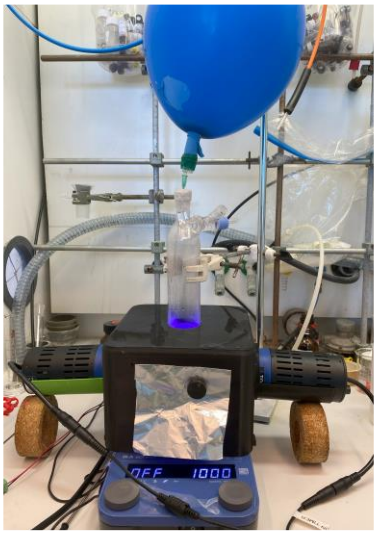

Figure S16: Reaction setup for a 5.0 mmol scale reaction.

9. Post-functionalizations

9.1 Synthesis of 45

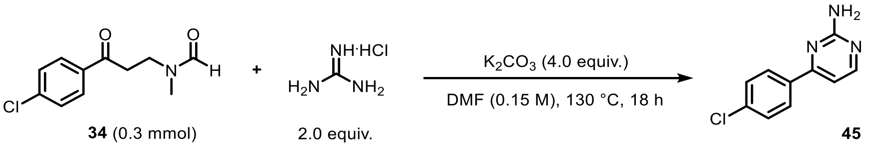

Compound **45** was synthesized adapting a procedure reported in the literature.[29] A 7 mL vial was charged with **34** (68.0 mg, 0.3 mmol, 1.0 equiv.), guanidine hydrochloride (57.0 mg, 0.6 mmol, 2.0 equiv.) and K_2CO_3 (166.0 mg, 1.2 mmol, 4 equiv.). DMF (2.0 mL) was added and the mixture was heated at 130 °C for 18 h. Upon completion, the solution was cooled to r.t., diluted with 1 M aq. NaOH (20 mL) and the aqueous phase was extracted with EtOAc (3 × 20 mL). The combined organic phase was dried over anhydrous Na_2SO_4, concentrated *in vacuo*, and the crude reaction mixture was purified via flash column chromatography on silica gel (Pentane:EtOAc 2:1) to afford **45** as a white solid (18.4 mg, 30% yield). Characterization data are in accordance with literature.[30]

^{1}H NMR (300 MHz, $CDCl_3$) δ 8.35 (d, *J* = 5.3 Hz, 1H), 7.95 (d, *J* = 8.6 Hz, 2H), 7.44 (d, *J* = 8.6 Hz, 2H), 7.01 (d, *J* = 5.2 Hz, 1H), 5.17 (br. s, 2H).
^{13}C NMR (75 MHz, $CDCl_3$) δ 164.3, 163.4, 159.0, 137.0, 135.7, 129.2, 128.5, 107.6.

9.2 Synthesis of 46

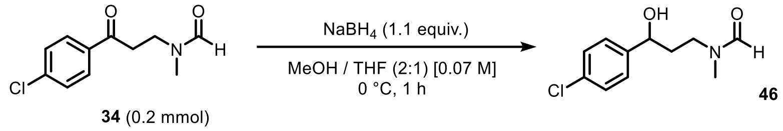

Compound **46** was synthesized adapting a procedure reported in the literature.[31] A 7 mL vial was charged with **34** (45.0 mg, 0.20 mmol, 1.0 equiv.), dry THF (1.0 mL) and MeOH (2.0 mL). The resulting solution was cooled to 0 °C and $NaBH_4$ (8.0 mg, 220.0 μmol, 1.1 equiv.) was added portionwise. Upon completion, MeOH (5 mL) was added and the mixture was concentrated *in vacuo*. The procedure was repeated three times. Afterwards, the crude was dissolved in DCM (10 mL) and 1 M aqueous HCl (10 mL) was added. The aqueous layer was extracted with DCM (3 x 5 mL) and the combined organic phase was washed with sat. aqueous NaCl (2 x 10 mL) and dried over anhydrous Na_2SO_4. Evaporation of the solvent afforded the product (1.5:1.0 mixture of rotamers) as a colourless oil (40.0 mg, 87% yield).

^{1}H NMR (300 MHz, $CDCl_3$) δ 8.00 (s, 0.6H), 7.99 (s, 0.4H), 7.32 – 7.27 (m, 2H), 7.26 – 7.21 (m, 2H), 4.57 (t, *J* = 6.6 Hz, 0.4H), 4.49 (dd, *J* = 9.9, 3.5 Hz, 0.6H), 4.21 (br. s, 1H), 3.97 – 3.86 (m, 0.6H), 3.51 – 3.41 (m, 0.4H), 3.28 – 3.21 (m, 0.4H), 3.11 – 3.02 (m, 0.6H), 2.95 (s, 1.8H), 2.87 – 2.71 (m, 1.2H), 1.90 – 1.67 (m, 2H).
^{13}C NMR (75 MHz, $CDCl_3$) δ 163.7, 163.2, 142.9, 142.5, 133.5, 133.0, 128.9, 128.6, 127.2, 70.2, 69.6, 46.3, 41.6, 36.9, 36.3, 35.1, 29.5.
HR-MS (ESI+) m/z $[M+H]^+$ calcd. for $C_{11}H_{14}ClNO_2$ 228.0786, found 228.0795.

9.3 Synthesis of 47

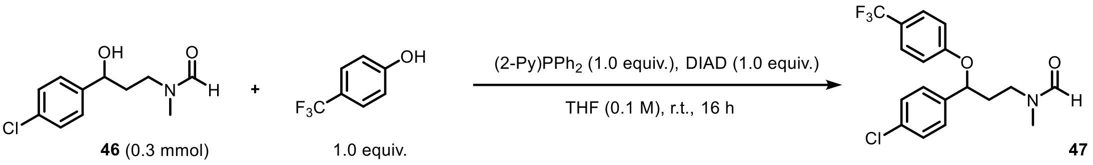

Compound **47** was synthesized adapting a procedure reported in the literature.[32] A 7 mL vial was charged with **46** (68.0 mg, 0.3 mmol, 1.0 equiv.) and 4-(trifluoromethyl)phenol (49.0 mg, 1.0 equiv.). After sealing the vial, it was evacuated and back-filled with N_2 three times and dry THF (3.0 mL, 0.1 M) was added via syringe under N_2 atmosphere. To the resulting stirring mixture, diphenyl-2-pyridylphosphine (79.0 mg, 0.3 mmol, 1.0 equiv.)* and diisopropyl diazene-1,2-dicarboxylate (59.0 µL, 0.3 mmol, 1.0 equiv.) were added and the mixture was stirred at r.t. for 16 h. Upon completion, the crude reaction mixture was concentrated *in vacuo* and purified via flash column chromatography on silica gel (gradient from Pentane:Acetone 10:1 to Pentane:Acetone 4:1) to afford **47** with a minor aliphatic impurity. Further purification via a second flash column chromatography on silica gel (gradient from Pentane:EtOAc 10:1 to Pentane:EtOAc 2:1) yielded the pure product (1.2:1.0 mixture of rotamers) as a colorless oil (37.0 mg, 33% yield).

^{1}H NMR (300 MHz, $CDCl_3$) δ 8.03 (s, 0.45H), 7.99 (s, 0.55H), 7.46 – 7.41 (m, 2H), 7.35 – 7.22 (m, 4H), 6.86 (dd, *J* = 8.7, 3.8 Hz, 2H), 5.18 (dd, *J* = 8.6, 4.4 Hz, 0.45H), 5.11 (dd, *J* = 9.0, 4.0 Hz, 0.45H), 3.60 – 3.31 (m, 2H), 2.94 (s, 1.35H), 2.90 (s, 1.65H), 2.21 – 2.07 (m, 2H).
^{13}C NMR (75 MHz, $CDCl_3$) δ 162.9, 162.8, 160.1, 159.7, 139.1, 138.6, 134.3, 134.0, 129.5, 129.3, 127.3, 127.2, 127.0 (q, *J* = 3.8 Hz), 124.4 (q, *J* = 270.0 Hz), 124.3 (q, *J* = 270.0 Hz), 123.8 (q, *J* = 32.3 Hz), 123.4 (q, *J* = 32.3 Hz), 115.9, 115.8, 77.7, 76.5, 46.0, 41.6, 37.0, 35.9, 35.0, 29.8.
^{19}F NMR (282 MHz, $CDCl_3$) δ −61.7 (2x).
HR-MS (FD+) m/z $[M]^+$ calcd. for $C_{18}H_{17}ClF_3NO_2$ 371.0894, found 371.0887.

*Note: The choice of diphenyl-2-pyridylphosphine was primarily guided by purification considerations. In fact, when triphenylphosphine was employed, isolation of the target compound proved challenging due to co-elution with triphenylphosphine oxide during silica gel chromatography. While the use of triphenylphosphine resulted in a higher ^{1}H NMR yield (53%), diphenyl-2-pyridylphosphine was preferred as it enabled successful chromatographic separation of the desired product.

10. Characterization data of aryl olefins

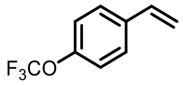

***1-(trifluoromethoxy)-4-vinylbenzene* (1f).** Prepared according to **GP1** from 4-(trifluoromethoxy)benzaldehyde (0.72 mL, 5.0 mmol). Purified via flash column chromatography on silica gel (Pentane) to afford the product as a colourless oil (440.0 mg, 46% yield).

Characterization data are in accordance with literature.[33]
^{1}H NMR (300 MHz, $CDCl_3$) δ 7.45 – 7.40 (m, 2H), 7.21 – 7.14 (m, 2H), 6.71 (dd, *J* = 17.6, 10.9 Hz, 1H), 5.74 (dd, *J* = 17.6, 0.8 Hz, 1H), 5.30 (dd, *J* = 10.9, 0.8 Hz, 1H).
^{19}F NMR (282 MHz, $CDCl_3$) δ −57.9.

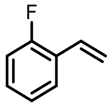

***1-fluoro-2-vinylbenzene* (1q).** Prepared according to **GP1** from 2-fluorobenzaldehyde (0.6 mL, 5.6 mmol). Purified via flash column chromatography on silica gel (Pentane) to afford the product as a colourless oil (465.0 mg, 68% yield).

Characterization data are in accordance with literature.[34]
^{1}H NMR (300 MHz, $CDCl_3$) δ 7.50 (td, *J* = 7.7, 1.9 Hz, 1H), 7.24 (m, 1H), 7.15 – 7.01 (m, 2H), 6.90 (dd, *J* = 17.8, 11.2 Hz, 1H), 5.84 (dd, *J* = 17.8, 1.2 Hz, 1H), 5.39 (dd, *J* = 11.2, 1.2 Hz, 1H).
^{19}F NMR (282 MHz, $CDCl_3$) δ −118.6.

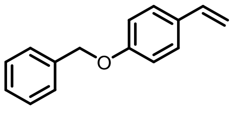

***1-(benzyloxy)-4-vinylbenzene* (1t).** Prepared according to **GP1** from 4-(benzyloxy)benzaldehyde (1.06 g, 5.0 mmol). Purified via flash column chromatography on silica gel (Pentane) to afford the product as a white solid (810.0 mg, 77% yield).

Characterization data are in accordance with literature.[35]
^{1}H NMR (300 MHz, $CDCl_3$) δ 7.46 – 7.32 (m, 7H), 6.97 – 6.88 (m, 2H), 6.66 (dd, *J* = 17.6, 10.9 Hz, 1H), 5.61 (dd, *J* = 17.6, 1.0 Hz, 1H), 5.13 (dd, *J* = 10.9, 1.0 Hz, 1H), 5.08 (s, 2H).

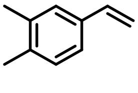

***1,2-dimethyl-4-vinylbenzene* (1v).** Prepared according to **GP1** from 3,4-dimethylbenzaldehyde (1.0 mL, 7.4 mmol). Purified via flash column chromatography on silica gel (Pentane) to afford the product as a colourless oil (943.0 mg, 96% yield).

Characterization data are in accordance with literature.[36]

^{1}H NMR (300 MHz, $CDCl_3$) δ 7.34 – 7.16 (m, 3H), 6.87 – 6.70 (m, 1H), 5.81 (dd, J = 17.5, 2.1 Hz, 1H), 5.29 (dd, J = 10.9, 2.1 Hz, 1H), 2.37 (s, 3H), 2.36 (s, 3H).

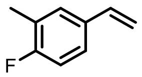

1-fluoro-2-methyl-4-vinylbenzene **(1z).** Prepared according to **GP1** from 4-fluoro-3-methylbenzaldehyde (690.0 mg, 5.0 mmol). Purified via flash column chromatography on silica gel (Pentane) to afford the product as a colourless oil (240.0 mg, 35% yield).

Characterization data are in accordance with literature.[37]
^{1}H NMR (300 MHz, $CDCl_3$) δ 7.21 (ddd, J = 13.4, 7.5, 2.3 Hz, 2H), 6.96 (dd, J = 9.4, 8.3 Hz, 1H), 6.66 (dd, J = 17.6, 10.9 Hz, 1H), 5.66 (d, J = 17.6 Hz, 1H), 5.20 (d, J = 10.9 Hz, 1H), 2.28 (d, J = 2.0 Hz, 3H).
^{19}F NMR (282 MHz, $CDCl_3$) δ −118.6.

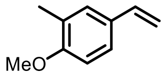

1-methoxy-2-methyl-4-vinylbenzene **(1aa).** Prepared according to **GP1** from 4-methoxy-3-methylbenzaldehyde (0.5 mL, 3.3 mmol). Purified via flash column chromatography on silica gel (Pentane) to afford the product as a colourless oil (488.0 mg, 99% yield).

Characterization data are in accordance with literature.[38]
^{1}H NMR (300 MHz, $CDCl_3$) δ 7.28 – 7.21 (m, 2H), 6.81 (dd, J = 8.3, 1.5 Hz, 1H), 6.76 – 6.65 (m, 1H), 5.66 (dd, J = 17.2, 1.2 Hz, 1H), 5.16 (dd, J = 10.9, 1.2 Hz, 1H), 3.87 (s, 3H), 2.28 (s, 3H).

11. Characterization data of oxo-amidomethylation products

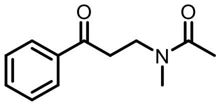

***N-methyl-N-(3-oxo-3-phenylpropyl)acetamide* (3).** Prepared according to **GP2** from **1a** (34.0 µL, 0.3 mmol) and **2a** (1.5 mL). Purified via flash column chromatography on silica gel (EtOAc) to afford the product (2:1 mixture of rotamers) as a colourless oil (46.0 mg, 75% yield).

Characterization data are in accordance with literature.[39]
^{1}H NMR (300 MHz, $CDCl_3$) δ 7.98 – 7.90 (m, 2H), 7.60 – 7.54 (m, 1H), 7.49 – 7.44 (m, 2H), 3.80 – 3.71 (m, 2H), 3.32 – 3.23 (m, 2H), 3.07 (s, 2H), 2.94 (s, 1H), 2.14 (s, 1H), 2.05 (s, 2H).
^{13}C NMR (75 MHz, $CDCl_3$) δ 199.2, 197.8, 171.0, 170.7, 136.8, 136.5, 133.8, 133.4, 128.9, 128.8, 128.3, 128.1, 46.0, 44.5, 37.7, 37.0, 37.0, 33.3, 22.1, 21.4.

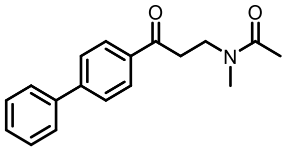

***N-(3-([1,1'-biphenyl]-4-yl)-3-oxopropyl)-N-methylacetamide* (4).** Prepared according to **GP2** from **1b** (54.0 mg, 0.3 mmol) and **2a** (1.5 mL). Purified via flash column chromatography on silica gel (gradient from Pentane:EtOAc 1:1 to 100% EtOAc) to afford the product (2:1 mixture of rotamers) as a white solid (73.0 mg, 86% yield).

^{1}H NMR (300 MHz, $CDCl_3$) δ 8.06 – 8.00 (m, 2H), 7.73 – 7.65 (m, 2H), 7.65 – 7.60 (m, 2H), 7.48 – 7.39 (m, 3H), 3.81 – 3.73 (m, 2H), 3.34 – 3.24 (m, 2H), 3.09 (s, 2H), 2.96 (s, 1H), 2.17 (s, 1H), 2.07 (s, 2H).
^{13}C NMR (75 MHz, $CDCl_3$) δ 198.8, 197.3, 171.0, 170.6, 146.4, 146.0, 139.9, 139.7, 135.5, 135.2, 129.1 (2x), 128.9, 128.7, 128.5, 128.4, 127.5, 127.5, 127.4, 46.0, 44.6, 37.7, 37.1, 37.1, 33.3, 22.1, 21.5.
HR-MS (ESI+) m/z $[M+H]^+$ calcd. for $C_{18}H_{19}NO_2$ 282.1489, found 282.1491.

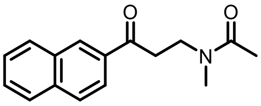

***N-methyl-N-(3-(naphthalen-2-yl)-3-oxopropyl)acetamide* (5).** Prepared according to **GP2** from **1c** (46.0 mg, 0.3 mmol) and **2a** (1.5 mL). Purified via flash column chromatography on silica gel (gradient from Pentane:EtOAc 1:1 to 100% EtOAc) to afford the product (2:1 mixture of rotamers) as a colourless oil (35.0 mg, 46% yield).

^{1}H NMR (300 MHz, $CDCl_3$) δ 8.48 (dd, *J* = 16.5, 1.8 Hz, 1H), 8.05 – 7.86 (m, 4H), 7.61 – 7.54 (m, 2H), 3.86 – 3.76 (m, 2H), 3.47 – 3.36 (m, 2H), 3.10 (s, 2H), 2.98 (s, 1H), 2.18 (s, 1H), 2.07 (s, 2H).
^{13}C NMR (75 MHz, $CDCl_3$) δ 199.1, 197.7, 171.1, 170.7, 135.9, 135.8, 134.1, 133.8, 132.6 (2x), 130.3, 130.0, 129.8, 129.7, 129.0, 128.9, 128.7, 128.6, 128.0, 127.9, 127.2, 126.9, 123.8, 123.6, 46.1, 44.7, 37.8, 37.1 (2x), 33.4, 22.1, 21.5.
HR-MS (ESI+) m/z $[M+H]^+$ calcd. for $C_{16}H_{17}NO_2$ 256.1332, found 256.1327.

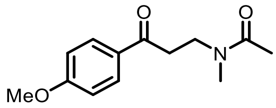

***N*-(3-(4-methoxyphenyl)-3-oxopropyl)-*N*-methylacetamide** **(6).** Prepared according to **GP2** from **1d** (40.0 µL, 0.3 mmol) and **2a** (1.5 mL). Purified via flash column chromatography on silica gel (EtOAc) to afford the product (2:1 mixture of rotamers) as a white solid (44.0 mg, 63% yield).

Characterization data are in accordance with literature.[39]
^{1}H NMR (300 MHz, $CDCl_3$) δ 8.07 – 7.79 (m, 2H), 7.08 – 6.73 (m, 2H), 3.88 (s, 1H), 3.86 (s, 2H), 3.79 – 3.69 (m, 2H), 3.27 – 3.15 (m, 2H), 3.07 (s, 2H), 2.94 (s, 1H), 2.15 (s, 1H), 2.06 (s, 2H).
^{13}C NMR (75 MHz, $CDCl_3$) δ 197.8, 196.3, 171.0, 170.7, 164.1, 163.8, 130.6, 130.4, 130.0, 129.7, 114.1, 113.9, 55.7, 55.6, 46.2, 44.7, 37.7, 36.7 (2x), 33.4, 22.1, 21.5.

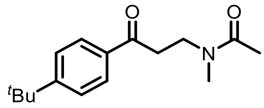

***N*-(3-(4-(tert-butyl)phenyl)-3-oxopropyl)-*N*-methylacetamide** **(7).** Prepared according to **GP2** from **1e** (55.0 µL, 0.3 mmol) and **2a** (1.5 mL). Purified via flash column chromatography on silica gel (EtOAc) to afford the product (2:1 mixture of rotamers) as a colourless oil (48.0 mg, 62% yield).

Characterization data are in accordance with literature.[39]
^{1}H NMR (300 MHz, $CDCl_3$) δ 7.95 – 7.83 (m, 2H), 7.51 – 7.44 (m, 2H), 3.80 – 3.69 (m, 2H), 3.30 – 3.17 (m, 2H), 3.06 (s, 2H), 2.94 (s, 1H), 2.14 (s, 1H), 2.05 (s, 2H), 1.34 (s, 3H), 1.33 (s, 6H).
^{13}C NMR (75 MHz, $CDCl_3$) δ 198.9, 197.4, 171.0, 170.7, 157.7, 157.2, 134.2, 134.0, 128.3, 128.1, 125.9, 125.7, 46.1, 44.5, 37.7, 37.0, 35.3, 35.2, 33.3, 31.2, 31.2, 22.1, 21.4.

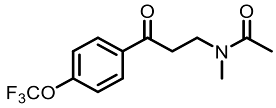

***N*-methyl-*N*-(3-oxo-3-(4-(trifluoromethoxy)phenyl)propyl)acetamide** **(8).** Prepared according to **GP2** from **1f** (56.0 mg, 0.3 mmol) and **2a** (1.5 mL). Purified via flash column chromatography on silica gel (gradient from Pentane:EtOAc 1:1 to 100% EtOAc) to afford the product (3:1 mixture of rotamers) as a colourless oil (47.0 mg, 55% yield).

^{1}H NMR (300 MHz, $CDCl_3$) δ 8.05 – 7.98 (m, 2H), 7.33 – 7.27 (m, 2H), 3.80 – 3.68 (m, 2H), 3.27 – 3.15 (m, 2H), 3.08 (s, 2.2H), 2.94 (s, 0.8H), 2.15 (s, 0.8H), 2.05 (s, 2.2H).
^{13}C NMR (75 MHz, $CDCl_3$) δ 197.6, 196.2, 171.1, 170.6, 153.1 (q, J = 1.7 Hz), 152.9 (q, J = 1.7 Hz), 135.0, 134.7, 130.3, 130.1, 120.7, 120.5, 120.3 (q, J = 258.8 Hz), 45.8, 44.5, 37.7, 37.1, 37.0, 33.3, 22.1, 21.4.
^{19}F NMR (282 MHz, $CDCl_3$) δ −57.6 (no rotameric peaks were observed).
HR-MS (ESI+) m/z $[M+H]^+$ calcd. for $C_{13}H_{14}F_3NO_3$ 290.0999, found 290.1003.

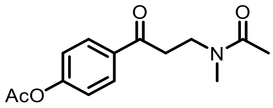

4-(3-(N-methylacetamido)propanoyl)phenyl acetate **(9).** Prepared according to **GP2** from **1g** (45.0 µL, 0.3 mmol) and **2a** (1.5 mL). Purified via flash column chromatography on silica gel (gradient from Pentane:EtOAc 1:1 to 100% EtOAc) to afford the product (2:1 mixture of rotamers) as a colourless oil (58.0 mg, 73% yield).

^{1}H NMR (300 MHz, $CDCl_3$) δ 8.02 – 7.95 (m, 2H), 7.22 – 7.15 (m, 2H), 3.73 (dt, *J* = 9.5, 6.8 Hz, 2H), 3.23 (dt, *J* = 10.3, 6.8 Hz, 2H), 3.06 (s, 2H), 2.93 (s, 1H), 2.31 (s, 1H), 2.30 (s, 2H), 2.14 (s, 1H), 2.05 (s, 2H).
^{13}C NMR (75 MHz, $CDCl_3$) δ 197.9, 196.4, 171.1, 170.7, 168.9 (2x), 154.8, 154.6, 134.3, 134.0, 129.9, 129.7, 122.2, 122.0, 45.9, 44.5, 37.7, 37.0 (2x), 33.3, 22.1, 21.4, 21.2.
HR-MS (ESI+) m/z $[M+H]^+$ calcd. for $C_{12}H_{17}NO_4$ 264.1230, found 264.1239.

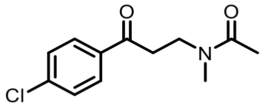

N-(3-(4-chlorophenyl)-3-oxopropyl)-N-methylacetamide **(10).** Prepared according to **GP2** from **1h** (36.0 µL, 0.3 mmol) and **2a** (1.5 mL). Purified via flash column chromatography on silica gel (EtOAc) to afford the product (3:1 mixture of rotamers) as a colourless oil (49.0 mg, 69% yield).

Characterization data are in accordance with literature.[39]
^{1}H NMR (300 MHz, $CDCl_3$) δ 7.93 – 7.86 (m, 2H), 7.48 – 7.40 (m, 2H), 3.79 – 3.68 (m, 2H), 3.30 – 3.18 (m, 2H), 3.07 (s, 2.2H), 2.93 (s, 0.8H), 2.14 (s, 0.8H), 2.05 (s, 2.2H).
^{13}C NMR (75 MHz, $CDCl_3$) δ 197.9, 196.5, 171.0, 170.6, 140.3, 139.9, 135.1, 134.8, 129.7, 129.5, 129.3, 129.1, 45.8, 44.5, 37.7, 37.0 (2x), 33.3, 22.1, 21.4.

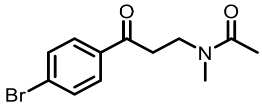

N-(3-(4-bromophenyl)-3-oxopropyl)-N-methylacetamide **(11).** Prepared according to **GP2** from **1i** (39.0 µL, 0.3 mmol) and **2a** (1.5 mL). Purified via flash column chromatography on silica gel (gradient from Pentane:EtOAc 1:1 to 100% EtOAc) to afford the product (3:1 mixture of rotamers) as a colourless oil (56.0 mg, 66% yield).

^{1}H NMR (300 MHz, $CDCl_3$) δ 7.86 – 7.77 (m, 2H), 7.65 – 7.56 (m, 2H), 3.78 – 3.67 (m, 2H), 3.26 – 3.18 (m, 2H), 3.06 (s, 2.2H), 2.92 (s, 0.8H), 2.13 (s, 0.8H), 2.04 (s, 2.2H).
^{13}C NMR (75 MHz, $CDCl_3$) δ 198.2, 196.7, 171.1, 170.7, 135.5, 135.2, 132.3, 132.1, 129.8, 129.6, 129.1, 128.7, 45.8, 44.5, 37.8, 37.0 (2x), 33.4, 22.1, 21.4.
HR-MS (ESI+) m/z $[M+H]^+$ calcd. for $C_{12}H_{14}BrNO_2$ 284.0281, found 284.0290.

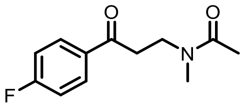

***N*-(3-(4-fluorophenyl)-3-oxopropyl)-*N*-methylacetamide (12).** Prepared according to **GP2** from **1j** (36.0 µL, 0.3 mmol) and **2a** (1.5 mL). Purified via flash column chromatography on silica gel (gradient from Pentane:EtOAc 1:1 to 100% EtOAc) to afford the product (3:1 mixture of rotamers) as a colourless oil (42.0 mg, 62% yield).

Characterization data are in accordance with literature.[39]

^{1}H NMR (300 MHz, $CDCl_3$) δ 8.05 – 7.93 (m, 2H), 7.18 – 7.08 (m, 2H), 3.73 (dt, *J* = 11.4, 6.8 Hz, 2H), 3.22 (dt, *J* = 10.1, 6.8 Hz, 2H), 3.07 (s, 2.2H), 2.93 (s, 0.8H), 2.14 (s, 0.8H), 2.05 (s, 2.2H).

^{13}C NMR (75 MHz, $CDCl_3$) δ 197.5, 196.1, 171.0, 170.6, 166.1 (d, *J* = 254.2 Hz), 166.0 (d, *J* = 253.5 Hz), 133.2 (d, *J* = 2.9 Hz), 132.9 (d, *J* = 3.0 Hz), 130.9 (d, *J* = 9.4 Hz), 130.8 (d, *J* = 9.5 Hz), 116.1 (d, *J* = 21.7 Hz), 115.9 (d, *J* = 21.7 Hz), 45.9, 44.6, 37.7, 37.0, 36.9, 33.3, 22.1, 21.4.

^{19}F NMR (282 MHz, $CDCl_3$) δ −104.1, −104.9.

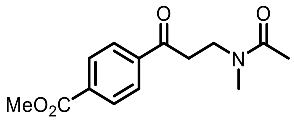

***methyl 4-(3-(N-methylacetamido)propanoyl)benzoate* (13).** Prepared according to **GP2** from **1k** (49.0 mg, 0.3 mmol) and **2a** (1.5 mL). Purified via flash column chromatography on silica gel (gradient from Pentane:EtOAc 1:1 to 100% EtOAc) to afford the product (2:1 mixture of rotamers) as a white solid (43.0 mg, 54% yield).

^{1}H NMR (300 MHz, $CDCl_3$) δ 8.16 – 8.09 (m, 2H), 8.04 – 7.95 (m, 2H), 3.94 (s, 1H), 3.93 (s, 2H), 3.75 (dt, *J* = 11.2, 6.7 Hz, 2H), 3.29 (dt, *J* = 10.9, 6.6 Hz, 2H), 3.08 (s, 2H), 2.94 (s, 1H), 2.15 (s, 1H), 2.05 (s, 2H).

^{13}C NMR (75 MHz, $CDCl_3$) δ 198.7, 197.3, 171.1, 170.7, 166.3, 166.1, 139.9, 139.5, 134.5, 134.1, 130.1, 130.0, 128.1, 128.0, 52.7, 52.6, 45.8, 44.4, 37.7, 37.4, 37.3, 33.3, 22.1, 21.4.

HR-MS (ESI+) m/z $[M+H]^+$ calcd. for $C_{14}H_{17}NO_4$ 264.1230, found 264.1224.

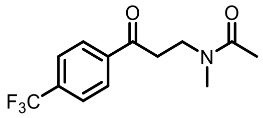

***N*-methyl-*N*-(3-(4-octylphenyl)-3-oxopropyl)acetamide (14).** Prepared according to **GP2** from **1l** (44.0 µL, 0.3 mmol) and **2a** (1.5 mL). Purified via flash column chromatography on silica gel (gradient from Pentane:EtOAc 1:1 to 100% EtOAc) to afford the product (2:1 mixture of rotamers) as a colourless oil (28.0 mg, 34% yield).

^{1}H NMR (300 MHz, $CDCl_3$) δ 8.10 – 8.02 (m, 2H), 7.77 – 7.69 (m, 2H), 3.82 – 3.70 (m, 2H), 3.34 – 3.24 (m, 2H), 3.09 (s, 2H), 2.94 (s, 1H), 2.15 (s, 1H), 2.06 (s, 2H).

^{13}C NMR (75 MHz, $CDCl_3$) δ 198.2, 196.8, 171.1, 170.6, 139.4, 139.1, 134.7 (q, *J* = 32.5 Hz), 134.6 (q, *J* = 32.5 Hz), 128.6, 128.5, 126.1 (q, *J* = 3.8 Hz), 125.9 (q, *J* = 3.7 Hz), 123.7 (q, *J* = 271.5 Hz), 45.7, 44.4, 37.8, 37.4, 37.3, 33.3, 22.1, 21.5.

^{19}F NMR (282 MHz, $CDCl_3$) δ −63.2 (2x).

HR-MS (ESI+) m/z $[M+H]^+$ calcd. for $C_{13}H_{14}F_3NO_2$ 274.1049, found 274.1049.

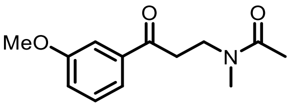

N-(3-(3-methoxyphenyl)-3-oxopropyl)-N-methylacetamide **(15).** Prepared according to **GP2** from **1m** (42.0 µL, 0.3 mmol) and **2a** (1.5 mL). Purified via flash column chromatography on silica gel (gradient from Pentane:EtOAc 1:1 to 100% EtOAc) to afford the product (2:1 mixture of rotamers) as a colourless oil (40.0 mg, 56% yield).

^{1}H NMR (300 MHz, $CDCl_3$) δ 7.58 – 7.44 (m, 2H), 7.41 – 7.33 (m, 1H), 7.15 – 7.08 (m, 1H), 3.85 (s, 1H), 3.84 (s, 2H), 3.74 (dt, *J* = 11.9, 6.9 Hz, 2H), 3.24 (dt, *J* = 11.9, 6.8 Hz, 2H), 3.07 (s, 2H), 2.93 (s, 1H), 2.14 (s, 1H), 2.05 (s, 2H).
^{13}C NMR (75 MHz, $CDCl_3$) δ 199.0, 197.6, 171.0, 170.6, 160.1, 160.0, 138.1, 137.8, 129.9, 129.8, 121.0, 120.7, 120.2, 120.1, 112.4, 112.2, 55.6 (2x), 46.0, 44.5, 37.7, 37.2 (2x), 33.3, 22.1, 21.4.
HR-MS (ESI+) m/z $[M+H]^+$ calcd. for $C_{13}H_{17}NO_3$ 236.1281, found 236.1293.

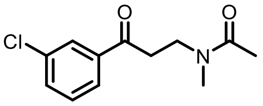

N-(3-(3-chlorophenyl)-3-oxopropyl)-N-methylacetamide **(16).** Prepared according to **GP2** from **1n** (38.0 µL, 0.3 mmol) and **2a** (1.5 mL). Purified via flash column chromatography on silica gel (gradient from Pentane:EtOAc 1:1 to 100% EtOAc) to afford the product (3:1 mixture of rotamers) as a pale yellow oil (35.0 mg, 49% yield).

^{1}H NMR (300 MHz, $CDCl_3$) δ 7.91 (dt, *J* = 6.7, 1.9 Hz, 1H), 7.82 (ddt, *J* = 9.2, 7.7, 1.4 Hz, 1H), 7.54 (dddd, *J* = 11.3, 8.0, 2.1, 1.1 Hz, 1H), 7.41 (dt, *J* = 10.3, 7.8 Hz, 1H), 3.73 (dt, *J* = 13.2, 6.8 Hz, 2H), 3.23 (dt, *J* = 11.0, 6.7 Hz, 2H), 3.07 (s, 2.2H), 2.93 (s, 0.8H), 2.14 (s, 0.8H), 2.05 (s, 2.2H).
^{13}C NMR (75 MHz, $CDCl_3$) δ 197.8, 196.5, 171.0, 170.6, 138.3, 138.0, 135.3, 135.1, 133.7, 133.3, 130.3, 130.1, 128.3, 128.2, 126.4, 126.1, 45.7, 44.4, 37.7, 37.2, 37.1, 33.3, 22.1, 21.4.
HR-MS (ESI+) m/z $[M+H]^+$ calcd. for $C_{12}H_{14}ClNO_2$ 240.0786, found 240.0797.

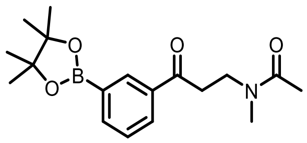

N-methyl-N-(3-oxo-3-(3-(4,4,5,5-tetramethyl-1,3,2-dioxaborolan-2-yl)phenyl)propyl)acetamide **(17).** Prepared according to **GP2** from **1o** (69.0 mg, 0.3 mmol) and **2a** (1.5 mL). Purified via flash column chromatography on silica gel (gradient from Pentane:EtOAc 1:1 to 100% EtOAc) to afford the product (2:1 mixture of rotamers) as a white solid (28.0 mg, 28% yield).

^{1}H NMR (300 MHz, $CDCl_3$) δ 8.33 (m, 1H), 8.12 – 8.01 (m, 1H), 8.01 – 7.93 (m, 1H), 7.51 – 7.41 (m, 1H), 3.80 – 3.71 (m, 2H), 3.39 – 3.25 (m, 2H), 3.08 (s, 2H), 2.95 (s, 1H), 2.16 (s, 1H), 2.06 (s, 2H), 1.36 (s, 4H), 1.35 (s, 8H).
^{13}C NMR (75 MHz, $CDCl_3$) δ 199.3, 198.0, 171.0, 170.7, 140.0, 139.6, 136.2, 135.9, 134.6, 134.5, 130.7, 130.6, 128.5, 128.2, 84.4, 84.3, 46.0, 44.2, 37.8, 37.2, 37.1, 33.9, 25.0, 22.1, 21.5.
^{11}B NMR (96 MHz, $CDCl_3$) δ 32.0.
HR-MS (ESI+) m/z $[M+H]^+$ calcd. for $C_{18}H_{26}BNO_4$ 332.2028, found 332.2035.

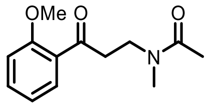

***N*-(3-(2-methoxyphenyl)-3-oxopropyl)-*N*-methylacetamide** **(18).** Prepared according to **GP2** from **1p** (40.0 µL, 0.3 mmol) and **2a** (1.5 mL). Purified via flash column chromatography on silica gel (gradient from Pentane:EtOAc 1:1 to 100% EtOAc) to afford the product (1.1:1.0 mixture of rotamers) as a colourless oil (29.0 mg, 40% yield).

^{1}H NMR (300 MHz, $CDCl_3$) δ 7.72 – 7.66 (m, 1H), 7.52 – 7.41 (m, 1H), 7.06 – 6.92 (m, 2H), 3.91 (s, 1.4H), 3.89 (s, 1.6H), 3.74 – 3.65 (m, 2H), 3.28 – 3.20 (m, 2H), 3.05 (s, 1.6H), 2.92 (s, 1.4H), 2.13 (s, 1.4H), 2.04 (s, 1.6H).
^{13}C NMR (75 MHz, $CDCl_3$) δ 201.1, 199.8, 170.7 (2x), 158.9 (2x), 134.3, 133.9, 130.6, 130.4, 128.0, 127.5, 121.0, 120.7, 111.7 (2x), 55.7, 55.6, 46.3, 44.1, 42.3, 42.0, 37.4, 33.3, 22.1, 21.4.
HR-MS (ESI+) m/z $[M+H]^+$ calcd. for $C_{13}H_{17}NO_3$ 236.1281, found 236.1283.

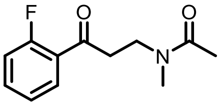

***N*-(3-(2-fluorophenyl)-3-oxopropyl)-*N*-methylacetamide** **(19).** Prepared according to **GP2** from **1q** (36.0 µL, 0.3 mmol) and **2a** (1.5 mL). Purified via flash column chromatography on silica gel (gradient from Pentane:EtOAc 1:1 to 100% EtOAc) to afford the product (1.6:1.0 mixture of rotamers) as a colourless oil (34.0 mg, 51% yield).

^{1}H NMR (300 MHz, $CDCl_3$) δ 7.88 – 7.80 (m, 1H), 7.55 – 7.45 (m, 1H), 7.24 – 7.17 (m, 1H), 7.10 (m, 1H), 3.72 (m, 2H), 3.24 (m, 2H), 3.06 (s, 1.85H), 2.92 (s, 1.15H), 2.13 (s, 1.15H), 2.04 (s, 1.85H).
^{13}C NMR (75 MHz, $CDCl_3$) δ 197.2 (d, *J* = 3.9 Hz), 196.0 (d, *J* = 3.9 Hz), 170.8, 170.7, 162.2 (d, *J* = 252.7 Hz), 162.1 (d, *J* = 253.5 Hz), 135.3 (d, *J* = 9.2 Hz), 134.8 (d, *J* = 9.1 Hz), 130.6 (d, *J* = 2.2 Hz), 130.5 (d, *J* = 2.9 Hz), 125.5 (d, *J* = 12.6 Hz), 125.1 (d, *J* = 12.7 Hz), 124.8 (d, *J* = 3.3 Hz), 124.5 (d, *J* = 3.4 Hz), 116.9 (d, *J* = 24.0 Hz), 116.8 (d, *J* = 24.0 Hz), 45.7 (d, *J* = 2.3 Hz), 43.8 (d, *J* = 2.2 Hz), 42.0 (d, *J* = 7.9 Hz), 41.6 (d, *J* = 7.3 Hz), 37.4, 33.2, 22.0, 21.4.
^{19}F NMR (282 MHz, $CDCl_3$) δ −109.3, −109.5.
HR-MS (ESI+) m/z $[M+H]^+$ calcd. for $C_{12}H_{14}FNO_2$ 224.1081, found 224.1091.

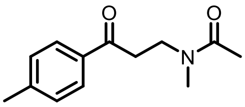

***N*-methyl-*N*-(3-oxo-3-(*p*-tolyl)propyl)acetamide** **(20).** Prepared according to **GP2** from **1r** (40.0 µL, 0.3 mmol) and **2a** (1.5 mL). Purified via flash column chromatography on silica gel (gradient from Pentane:EtOAc 1:1 to 100% EtOAc) to afford the product (2:1 mixture of rotamers) as a colourless oil (46.0 mg, 70% yield).

Characterization data are in accordance with literature.[39]
^{1}H NMR (300 MHz, $CDCl_3$) δ 7.88 – 7.81 (m, 2H), 7.30 – 7.21 (m, 2H), 3.78 – 3.68 (m, 2H), 3.28 – 3-17 (m, 2H), 3.05 (s, 2H), 2.93 (s, 1H), 2.41 (s, 1H), 2.39 (s, 2H), 2.13 (s, 1H), 2.04 (s, 2H).
^{13}C NMR (75 MHz, $CDCl_3$) δ 198.8, 197.4, 170.9, 170.6, 144.7, 144.2, 134.3, 134.0, 129.6, 129.4, 128.4, 128.2, 46.1, 44.5, 37.7, 36.9 (2x), 33.3, 22.1, 21.8, 21.7, 21.4.

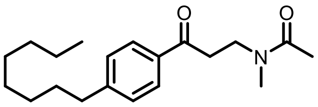

***N*-methyl-*N*-(3-(4-octylphenyl)-3-oxopropyl)acetamide (21).** Prepared according to **GP2** from **1s** (74.0 µL, 0.3 mmol) and **2a** (1.5 mL). Purified via flash column chromatography on silica gel (gradient from Pentane:EtOAc 1:1 to 100% EtOAc) to afford the product (2:1 mixture of rotamers) as a colourless oil (49.0 mg, 52% yield).

^{1}H NMR (300 MHz, $CDCl_3$) δ 7.87 (m, 2H), 7.29 – 7.24 (m, 2H), 3.79 – 3.69 (m, 2H), 3.29 – 3.18 (m, 2H), 3.06 (s, 2H), 2.94 (s, 1H), 2.65 (td, *J* = 7.8, 5.4 Hz, 2H), 2.14 (s, 1H), 2.05 (s, 2H), 1.65 – 1.57 (m, 2H), 1.32 – 1.23 (dt, *J* = 13.3, 4.1 Hz, 10H), 0.88 (t, 3H).
^{13}C NMR (75 MHz, $CDCl_3$) δ 198.9, 197.4, 171.0, 170.6, 149.7, 149.2, 134.6, 134.2, 129.0, 128.8, 128.4, 128.2, 46.1, 44.5, 37.7, 36.9, 36.1, 33.3, 32.0, 31.2, 29.5, 29.4, 29.3, 22.8, 22.1, 21.4, 14.2.
HR-MS (ESI+) m/z $[M+H]^+$ calcd. for $C_{20}H_{31}NO_2$ 318.2428, found 318.2422.

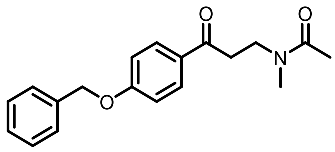

***N*-(3-(4-(benzyloxy)phenyl)-3-oxopropyl)-*N*-methylacetamide (22).** Prepared according to **GP2** from **1t** (63.0 mg, 0.3 mmol) and **2a** (1.5 mL). Purified via flash column chromatography on silica gel (gradient from Pentane:EtOAc 1:1 to 100% EtOAc) to afford the product (2:1 mixture of rotamers) as a colourless oil (35.0 mg, 38% yield).

^{1}H NMR (300 MHz, $CDCl_3$) δ 8.00 – 7.89 (m, 2H), 7.44 – 7.33 (m, 5H), 7.07 – 6.98 (m, 2H), 5.13 (s, 0.65H), 5.12 (s, 1.35H), 3.78 – 3.68 (m, 2H), 3.25 – 3.14 (m, 2H), 3.05 (s, 2H), 2.93 (s, 1H), 2.14 (s, 1H), 2.05 (s, 2H).
^{13}C NMR (75 MHz, $CDCl_3$) δ 197.7, 196.2, 171.0, 170.6, 163.1, 162.9, 136.2, 136.1, 130.6, 130.4, 130.1, 129.8, 128.8 (2x), 128.4, 128.3, 127.6, 114.9, 114.7, 70.3, 70.2, 46.1, 44.7, 37.7, 36.7 (2x), 33.3, 22.1, 21.4.
HR-MS (ESI+) m/z $[M+H]^+$ calcd. for $C_{19}H_{21}NO_3$ 312.1594, found 312.1605.

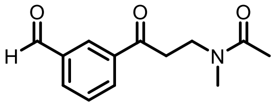

***N*-(3-(3-formylphenyl)-3-oxopropyl)-*N*-methylacetamide (23).** Prepared according to **GP2** from **1u** (38.0 µL, 0.3 mmol) and **2a** (1.5 mL). Purified via flash column chromatography on silica gel (gradient from Pentane:EtOAc 1:1 to 100% EtOAc) to afford the product (3:1 mixture of rotamers) as a colourless oil (36.0 mg, 51% yield).

^{1}H NMR (300 MHz, $CDCl_3$) δ 10.08 (s, 0.25H), 10.06 (s, 0.75H), 8.46 – 8.39 (m, 1H), 8.25 – 8.18 (m, 1H), 8.12 – 8.04 (m, 1H), 7.72 – 7.60 (m, 1H), 3.83 – 3.69 (m, 2H), 3.37 – 3.26 (m, 2H), 3.09 (s, 2.2H), 2.94 (s, 0.8H), 2.15 (s, 0.8H), 2.05 (s, 2.2H).

^{13}C NMR (75 MHz, $CDCl_3$) δ 198.0, 196.7, 191.5, 191.3, 171.1, 170.6, 137.4, 137.2, 136.9, 136.8, 134.4, 133.7, 133.6, 133.4, 129.9, 129.7, 129.6, 129.0, 45.7, 44.3, 37.7, 37.2, 37.1, 33.3, 22.0, 21.4.
HR-MS (ESI+) m/z $[M+H]^+$ calcd. for $C_{13}H_{15}NO_3$ 234.1125, found 234.1127.

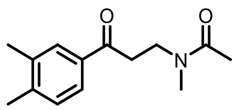

N-(3-(3,4-dimethylphenyl)-3-oxopropyl)-N-methylacetamide* (24)*. Prepared according to **GP2** from **1v** (40.0 mg, 0.3 mmol) and **2a** (1.5 mL). Purified via flash column chromatography on silica gel (gradient from Pentane:EtOAc 1:1 to 100% EtOAc) to afford the product (2:1 mixture of rotamers) as a pale yellow oil (43.0 mg, 61% yield).

^1H NMR (300 MHz, $CDCl_3$) δ 7.73 – 7.62 (m, 2H), 7.19 (t, *J* = 7.8 Hz, 1H), 3.77 – 3.68 (m, 2H), 3.27 – 3.16 (m, 2H), 3.04 (s, 2H), 2.92 (s, 1H), 2.30 (s, 2H), 2.28 (s, 4H), 2.12 (s, 2H), 2.03 (s, 1H).
^{13}C NMR (75 MHz, $CDCl_3$) δ 199.0, 197.6, 170.9, 170.6, 143.4, 142.9, 137.3, 137.0, 134.7, 134.4, 130.1, 129.9, 129.3, 129.1, 125.9, 125.8, 46.1, 44.5, 37.6, 36.9, 36.8, 33.3, 22.0, 21.4, 20.1 (2x), 19.9, 19.8.
HR-MS (ESI+) m/z $[M+H]^+$ calcd. for $C_{14}H_{19}NO_2$ 234.1489, found 234.1493.

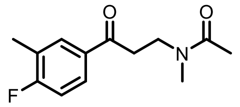

***N-(3-(4-fluoro-3-methylphenyl)-3-oxopropyl)-N-methylacetamide* (25).** Prepared according to **GP2** from **1w** (41.0 mg, 0.3 mmol) and **2a** (1.5 mL). Purified via flash column chromatography on silica gel (gradient from Pentane:EtOAc 1:1 to 100% EtOAc) to afford the product (2.5:1.0 mixture of rotamers) as a colourless oil (35.0 mg, 49% yield).

^1H NMR (300 MHz, $CDCl_3$) δ 7.85 – 7.73 (m, 2H), 7.05 (q, *J* = 9.0 Hz, 1H), 3.72 (dt, *J* = 11.5, 6.8 Hz, 2H), 3.20 (dt, *J* = 10.0, 6.8 Hz, 2H), 3.05 (s, 2.15H), 2.92 (s, 0.85H), 2.31 (d, *J* = 2.1 Hz, 0.85H), 2.29 (d, *J* = 2.1 Hz, 2.15H), 2.13 (s, 0.85H), 2.04 (s, 2.15H).
^{13}C NMR (75 MHz, $CDCl_3$) δ 197.8, 196.4, 171.0, 170.6, 164.7 (d, *J* = 252.7 Hz), 164.5 (d, *J* = 252.0 Hz), 132.9 (d, *J* = 3.3 Hz), 132.7 (d, *J* = 3.5 Hz), 132.1 (d, *J* = 6.7 Hz), 131.9 (d, *J* = 6.6 Hz), 128.2 (d, *J* = 9.3 Hz), 128.0 (d, *J* = 9.4 Hz), 125.8 (d, *J* = 17.9 Hz), 125.5 (d, *J* = 17.8 Hz), 115.6 (d, *J* = 23.3 Hz), 115.4 (d, *J* = 22.5 Hz), 45.9, 44.5, 37.7, 36.9 (2x), 33.3, 22.1, 21.4, 14.7 (d, *J* = 3.5 Hz), 14.6 (d, *J* = 3.5 Hz).
^{19}F NMR (282 MHz, $CDCl_3$) δ −108.2, −109.1.
HR-MS (ESI+) m/z $[M+H]^+$ calcd. for $C_{13}H_{14}F_3NO_3$ 238.1238, found 238.1245.

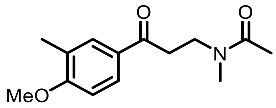

***N-(3-(4-methoxy-3-methylphenyl)-3-oxopropyl)-N-methylacetamide* (26).** Prepared according to **GP2** from **1x** (45.0 mg, 0.3 mmol) and **2a** (1.5 mL). Purified via flash column chromatography on silica gel (gradient from Pentane:EtOAc 1:1 to 100% EtOAc) to afford the product (2:1 mixture of rotamers) as a colourless oil (32.0 mg, 43% yield).

^{1}H NMR (300 MHz, $CDCl_3$) δ 7.88 – 7.73 (m, 2H), 6.84 (dd, *J* = 8.6, 5.8 Hz, 1H), 3.89 (s, 1H), 3.88 (s, 2H), 3.78 – 3.68 (m, 2H), 3.25 – 3.15 (m, 2H), 3.05 (s, 2H), 2.93 (s, 1H), 2.24 (s, 1H), 2.22 (s, 2H), 2.14 (s, 1H), 2.05 (s, 2H).
^{13}C NMR (75 MHz, $CDCl_3$) δ 198.0, 196.6, 171.0, 170.7, 162.3, 162.0, 130.9, 130.7, 129.4, 129.1, 128.5, 128.3, 127.2, 127.0, 109.5, 109.4, 55.7, 55.6, 46.3, 44.7, 37.7, 36.6, 33.4, 22.1, 21.4, 16.4, 16.3.
HR-MS (ESI+) m/z $[M+H]^+$ calcd. for $C_{14}H_{19}NO_3$ 250.1438, found 250.1446.

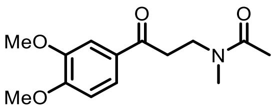

N-(3-(3,4-dimethoxyphenyl)-3-oxopropyl)-N-methylacetamide* (27)*. Prepared according to **GP2** from **1y** (44.0 µL, 0.3 mmol) and **2a** (1.5 mL). Purified via flash column chromatography on silica gel (gradient from Pentane:EtOAc 1:1 to 100% EtOAc) to afford the product (2:1 mixture of rotamers) as a white solid (30.0 mg, 38% yield).

^{1}H NMR (300 MHz, $CDCl_3$) δ 7.64 – 7.47 (m, 2H), 6.87 (dd, *J* = 8.4, 3.6 Hz, 1H), 3.93 (s, 2H), 3.91 (s, 4H), 3.72 (dt, *J* = 9.5, 6.8 Hz, 2H), 3.20 (dt, *J* = 10.0, 6.8 Hz, 2H), 3.05 (s, 2H), 2.92 (s, 1H), 2.13 (s, 1H), 2.04 (s, 2H).
^{13}C NMR (75 MHz, $CDCl_3$) δ 197.7, 196.3, 171.0, 170.6, 153.8, 153.5, 149.3, 149.1, 130.0, 129.8, 123.1, 122.8, 110.2 (2x), 110.1, 110.0, 56.21, 56.2, 56.1 (2x), 46.2, 44.8, 37.7, 36.6, 36.5, 33.3, 22.1, 21.4.
HR-MS (ESI+) m/z $[M+H]^+$ calcd. for $C_{14}H_{19}NO_4$ 266.1387, found 266.1393.

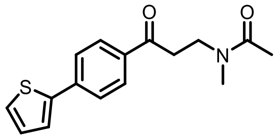

N-methyl-N-(3-oxo-3-(4-(thiophen-2-yl)phenyl)propyl)acetamide* (28)*. Prepared according to **GP2** from **1z** (56.0 mg, 0.3 mmol) and **2a** (1.5 mL). Purified via flash column chromatography on silica gel (gradient from Pentane:EtOAc 1:1 to 100% EtOAc) to afford the product (2:1 mixture of rotamers) as a white solid (36.0 mg, 42% yield).

^{1}H NMR (300 MHz, $CDCl_3$) δ 8.00 – 7.91 (m, 2H), 7.72 – 7.65 (m, 2H), 7.44 – 7.40 (m, 1H), 7.38 – 7.33 (m, 1H), 7.10 (dt, *J* = 5.1, 3.6 Hz, 1H), 3.79 – 3.70 (m, 2H), 3.31 – 3.20 (m, 2H), 3.07 (s, 2H), 2.94 (s, 1H), 2.15 (s, 1H), 2.06 (s, 2H).
^{13}C NMR (75 MHz, $CDCl_3$) δ 198.3, 196.9, 171.0, 170.6, 143.0, 142.7, 139.4, 139.01, 135.3, 135.0, 129.0, 128.9, 128.5 (2x), 126.9, 126.6, 125.9, 125.8, 124.9, 124.8, 46.0, 44.5, 37.7, 36.9, 33.3, 22.1, 21.4
HR-MS (ESI+) m/z $[M+H]^+$ calcd. for $C_{16}H_{17}NO_2S$ 288.1053, found 288.1057.

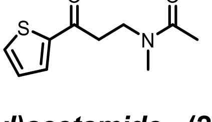

N-methyl-N-(3-oxo-3-(thiophen-2-yl)propyl)acetamide* (29)*. Prepared according to **GP2** from **1aa** (33.0 mg, 0.3 mmol) and **2a** (1.5 mL). Purified via flash column chromatography on silica gel

(gradient from Pentane:EtOAc 1:1 to 100% EtOAc) to afford the product (2:1 mixture of rotamers) as a yellow oil (19.0 mg, 30% yield).

^{1}H NMR (300 MHz, $CDCl_3$) δ 7.75 (ddd, *J* = 14.4, 3.8, 1.1 Hz, 1H), 7.66 (ddd, *J* = 13.1, 4.9, 1.1 Hz, 1H), 7.14 (ddd, *J* = 8.8, 5.0, 3.8 Hz, 1H), 3.79 – 3.69 (m, 2H), 3.25 – 3.14 (m, 2H), 3.06 (s, 2H), 2.94 (s, 1H), 2.15 (s, 1H), 2.05 (s, 2H).
^{13}C NMR (75 MHz, $CDCl_3$) δ 192.1, 190.5, 171.1, 170.7, 144.2, 143.8, 134.6, 134.1, 132.7, 132.4, 128.5, 128.4, 46.1, 44.7, 37.9, 37.8, 33.3, 22.1, 21.4.
HR-MS (ESI+) m/z $[M+H]^+$ calcd. for $C_{10}H_{13}NO_2S$ 212.0740, found 212.0745.

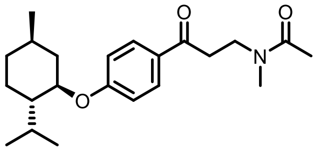

***N*-(3-(4-(((1R,2S,5R)-2-isopropyl-5-methylcyclohexyl)oxy)phenyl)-3-oxopropyl)-N-methylacetamide (30).** Prepared according to **GP2** from **1ab** (77.0 mg, 0.3 mmol) and **2a** (1.5 mL). Purified via flash column chromatography on silica gel (gradient from Pentane:EtOAc 1:1 to 100% EtOAc) to afford the product (2:1 mixture of rotamers) as a colourless oil (42.0 mg, 39% yield).

^{1}H NMR (300 MHz, $CDCl_3$) δ 7.96 – 7.87 (m, 2H), 6.95 – 6.88 (m, 2H), 4.13 (td, *J* = 10.5, 5.3 Hz, 1H), 3.77 – 3.70 (m, 2H), 3.24 – 3.15 (m, 2H), 3.06 (s, 2H), 2.94 (s, 1H), 2.14 (s, 1H), 2.13 – 2.09 (m, 2H), 2.05 (s, 2H), 1.76 – 1.68 (m, 2H), 1.55 – 1.45 (m, 2H), 1.16 – 0.97 (m, 2H), 0.97 – 0.89 (m, 7H), 0.73 (d, *J* = 7.0 Hz, 1H), 0.72 (d, *J* = 7.0 Hz, 2H).
^{13}C NMR (75 MHz, $CDCl_3$) δ 197.6, 196.2, 171.0, 170.7, 163.1, 162.8, 130.7, 130.5, 129.5, 129.1, 115.2, 115.1, 77.9, 77.7, 48.0, 46.3, 44.8, 40.2, 37.7, 36.7, 36.6, 34.5 (2x), 33.4, 31.5, 26.3 (2x), 23.9, 22.2, 22.1, 21.5, 20.8, 16.7.
HR-MS (ESI+) m/z $[M+H]^+$ calcd. for $C_{22}H_{33}NO_3$ 360.2533, found 360.2541.

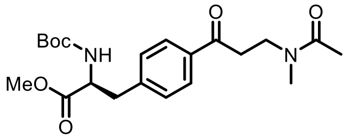

***tert*-butyl (S)-(1-methoxy-3-(4-(3-(N-methylacetamido)propanoyl)phenyl)-1-oxopropan-2-yl)-l2-azanecarboxylate (31).** Prepared according to **GP2** from **1ac** (92.0 mg, 0.3 mmol) and **2a** (1.5 mL). Purified via flash column chromatography on silica gel (gradient from Pentane:EtOAc 1:1 to 100% EtOAc) to afford the product (2:1 mixture of rotamers) as a yellow oil (49.0 mg, 40% yield).

^{1}H NMR (300 MHz, $CDCl_3$) δ 7.88 (m, 2H), 7.24 – 7.18 (m, 2H), 5.08 – 4.98 (m, 1H), 4.64 – 4.53 (m, 1H), 3.74 (d, *J* = 3.3 Hz, 2H), 3.71 (s, 1H), 3.70 (s, 2H), 3.28 – 3.20 (m, 2H), 3.06 (s, 2H), 2.93 (s, 1H), 2.14 (s, 1H), 2.05 (s, 2H), 1.40 (s, 9H).
^{13}C NMR (75 MHz, $CDCl_3$) δ 198.7, 197.3, 172.0 (2x), 171.1, 170.7, 155.1, 142.7, 142.1, 135.6, 135.3, 129.9, 129.8, 128.5, 128.3, 80.3, 54.3, 52.5 (2x), 45.9, 44.5, 38.5, 37.7, 37.0 (2x), 33.4, 28.4, 22.1, 21.4.
HR-MS (ESI+) m/z $[M+H]^+$ calcd. for $C_{21}H_{30}N_2O_6$ 407.2177, found 407.2181.

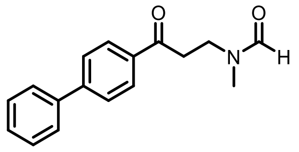

***N*-(3-([1,1'-biphenyl]-4-yl)-3-oxopropyl)-*N*-methylformamide** **(32).** Prepared according to **GP2** from **1b** (54.0 mg, 0.3 mmol) and **2b** (1.5 mL). Purified via flash column chromatography on silica gel (gradient from Pentane:EtOAc 1:1 to 100% EtOAc) to afford the product (1:1 mixture of rotamers) as a white solid (49.0 mg, 61% yield).

^{1}H NMR (300 MHz, $CDCl_3$) δ 8.19 (s, 0.5H), 8.06 – 8.02 (m, 2H), 7.99 (s, 0.5H), 7.72 – 7.67 (m, 2H), 7.64 – 7.61 (m, 2H), 7.49 – 7.45 (m, 2H), 7.43 – 7.40 (m, 1H), 3.75 (td, *J* = 6.7, 2.0 Hz, 2H), 3.32 (t, *J* = 6.7 Hz, 1H), 3.25 (t, *J* = 6.7 Hz, 1H), 3.04 (s, 1.5H), 2.90 (s, 1.5H).
^{13}C NMR (75 MHz, $CDCl_3$) δ 198.1, 196.9, 163.2, 163.0, 146.5, 146.2, 139.8, 139.7, 135.3, 135.1, 129.1 (2x), 128.8, 128.7, 128.5, 128.4, 127.5, 127.4 (2x), 44.6, 40.7, 36.9, 36.4, 35.8, 29.8.
HR-MS (ESI+) m/z $[M+H]^+$ calcd. for $C_{17}H_{17}NO_2$ 268.1332, found 268.1337.

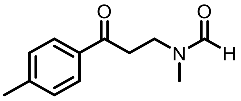

***N*-methyl-*N*-(3-oxo-3-(p-tolyl)propyl)formamide** **(33).** Prepared according to **GP2** from **1r** (40.0 µL, 0.3 mmol) and **2b** (1.5 mL). Purified via flash column chromatography on silica gel (gradient from Pentane:EtOAc 1:1 to 100% EtOAc) to afford the product (1:1 mixture of rotamers) as a colourless oil (43.0 mg, 69% yield).

Characterization data are in accordance with literature.[40]
^{1}H NMR (300 MHz, $CDCl_3$) δ 8.15 (s, 0.5H), 8.01 (s, 0.5H), 7.83 (t, *J* = 7.7 Hz, 2H), 7.28 – 7.23 (t, *J* = 7.7 Hz, 2H), 3.71 (td, *J* = 6.7, 1.9 Hz, 2H), 3.25 (t, *J* = 6.7 Hz, 1H), 3.19 (t, *J* = 6.7 Hz, 1H), 3.01 (s, 1.5H), 2.89 – 2.84 (m, 1.5H), 2.41 (s, 1.5H), 2.40 (s, 1.5H).
^{13}C NMR (75 MHz, $CDCl_3$) δ 198.1, 196.9, 163.2, 162.9, 144.7, 144.4, 134.2, 134.0, 129.6, 129.5, 128.3, 128.2, 44.7, 40.7, 36.7, 36.2, 35.8, 29.7, 21.8, 21.7.

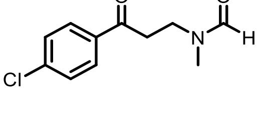

***N*-(3-(4-chlorophenyl)-3-oxopropyl)-*N*-methylformamide** **(34).** Prepared according to **GP2** from **1h** (36.0 µL, 0.3 mmol) and **2b** (1.5 mL). Purified via flash column chromatography on silica gel (gradient from Pentane:EtOAc 1:1 to 100% EtOAc) to afford the product (1.2:1.0 mixture of rotamers) as a white solid (50.0 mg, 74% yield).

Characterization data are in accordance with literature.[40]
^{1}H NMR (300 MHz, $CDCl_3$) δ 8.16 (s, 0.45H), 8.00 (s, 0.55H), 7.91 – 7.84 (m, 2H), 7.46 – 7.40 (m, 2H), 3.71 (td, *J* = 6.7, 3.1 Hz, 2H), 3.24 (t, *J* = 6.7 Hz, 1.1H), 3.18 (t, *J* = 6.6 Hz, 0.9H), 3.01 (s, 1.65H), 2.86 (s, 1.35H).
^{13}C NMR (75 MHz, $CDCl_3$) δ 197.2, 196.1, 163.2, 162.9, 140.3, 140.0, 134.9, 134.8, 129.6, 129.5, 129.3, 129.1, 44.4, 40.6, 36.8, 36.3, 35.8, 29.7.

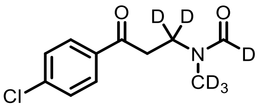

N-(3-(4-chlorophenyl)-3-oxopropyl-1,1-d₂)-N-(methyl-d₃)formamide-d **(35).** Prepared according to **GP2** from **1h** (36.0 µL, 0.3 mmol) and **2c** (1.5 mL). Purified via flash column chromatography on silica gel (gradient from Pentane:EtOAc 1:1 to 100% EtOAc) to afford the product (1:1 mixture of rotamers) as a white solid (43.0 mg, 61% yield).

^{1}H NMR (300 MHz, $CDCl_3$) δ 7.88 – 7.83 (m, 2H), 7.45 – 7.39 (m, 2H), 3.22 (s, 1H), 3.16 (s, 1H).
^{13}C NMR (75 MHz, $CDCl_3$) δ 197.2, 163.1 (t, *J* = 21.1 Hz), 162.8 (t, *J* = 21.1 Hz), 140.2, 139.9, 134.9, 134.7, 129.6, 129.4, 129.2, 129.1, 43.7 (p, *J* = 21.1 Hz), 39.8 (p, *J* = 21.1 Hz), 36.6, 36.1, 35.9 – 34.3 (m), 29.8 – 27.9 (m).
HR-MS (FD+) m/z $[M]^+$ calcd. for $C_{13}H_6D_6ClNO_2$ 231.0928, found 231.0934.

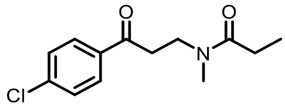

N-(3-(4-chlorophenyl)-3-oxopropyl)-N-methylpropionamide **(36).** Prepared according to **GP2** from **1h** (36.0 µL, 0.3 mmol) and **2d** (1.5 mL). Purified via flash column chromatography on silica gel (gradient from Pentane:EtOAc 1:1 to 100% EtOAc) to afford the product (3:1 mixture of rotamers) as a pale yellow oil (36.0 mg, 47% yield).

^{1}H NMR (300 MHz, $CDCl_3$) δ 7.92 – 7.83 (m, 2H), 7.44 – 7.37 (m, 2H), 3.71 (dt, *J* = 12.3, 6.9 Hz, 2H), 3.19 (dt, *J* = 9.7, 6.9 Hz, 2H), 3.03 (s, 2.2H), 2.92 (s, 0.8H), 2.37 (q, *J* = 7.4 Hz, 0.5H), 2.27 (q, *J* = 7.4 Hz, 2.5H), 1.17 – 1.04 (m, 3H).
^{13}C NMR (75 MHz, $CDCl_3$) δ 198.0, 196.6, 174.1, 173.7, 140.2, 139.8, 135.0, 134.8, 129.7, 129.4, 129.2, 129.0, 44.8 (2x), 37.1, 37.0, 36.7, 33.4, 26.9, 26.3, 9.6, 9.2.
HR-MS (ESI+) m/z $[M+H]^+$ calcd. for $C_{13}H_{16}ClNO_2$ 254.0942, found 254.0949.

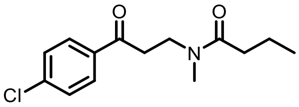

N-(3-(4-chlorophenyl)-3-oxopropyl)-N-methylbutyramide **(37).** Prepared according to **GP2** from **1h** (36.0 µL, 0.3 mmol) and **2e** (1.5 mL). Purified via flash column chromatography on silica gel (gradient Pentane:EtOAc 1:1) to afford the product (3:1 mixture of rotamers) as a colourless oil (30.0 mg, 38% yield).

^{1}H NMR (300 MHz, $CDCl_3$) δ 7.93 – 7.85 (m, 2H), 7.47 – 7.39 (m, 2H), 3.73 (dt, *J* = 13.7, 6.9 Hz, 2H), 3.21 (dt, *J* = 9.9, 6.9 Hz, 2H), 3.05 (s, 2.2H), 2.93 (s, 0.8H), 2.33 (t, *J* = 7.5 Hz, 0.5H), 2.28 – 2.22 (m, 1.5H), 1.68 – 1.57 (m, 2H), 0.94 (t, *J* = 7.4 Hz, 3H).
^{13}C NMR (75 MHz, $CDCl_3$) δ 198.1, 196.6, 173.4, 173.0, 140.3, 139.8, 135.1, 134.8, 129.7, 129.5, 129.3, 129.1, 45.0, 44.8, 37.2, 37.1, 36.9, 35.7, 35.1, 33.5, 18.9, 18.5, 14.1 (2x).
HR-MS (ESI+) m/z $[M+H]^+$ calcd. for $C_{14}H_{18}ClNO_2$ 268.1099, found 268.1108.

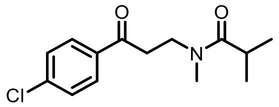

***N*-(3-(4-chlorophenyl)-3-oxopropyl)-*N*-methylisobutyramide** **(38).** Prepared according to **GP2** from **1h** (36.0 µL, 0.3 mmol) and **2f** (1.5 mL). Purified via flash column chromatography on silica gel (Pentane:EtOAc 1:1) to afford the product (3:1 mixture of rotamers) as a colourless oil (41.0 mg, 51% yield).

^{1}H NMR (300 MHz, $CDCl_3$) δ 7.96 – 7.84 (m, 2H), 7.49 – 7.39 (m, 2H), 3.83 – 3.67 (m, 2H), 3.25 – 3.17 (m, 2H), 3.10 (s, 2.2H), 2.94 (s, 0.8H), 2.87 – 2.69 (m, 1H), 1.12 (d, *J* = 6.7 Hz, 1.5H), 1.09 (d, *J* = 6.7 Hz, 4.5H).
^{13}C NMR (75 MHz, $CDCl_3$) δ 198.1, 196.6, 177.4, 177.3, 140.3, 139.9, 135.1, 134.8, 129.8, 129.5, 129.3, 129.1, 45.1, 44.8, 37.6, 37.0, 36.7, 33.8, 30.6, 30.3, 19.9, 19.2.
HR-MS (ESI+) m/z $[M+H]^+$ calcd. for $C_{14}H_{18}ClNO_2$ 268.1099, found 268.1107.

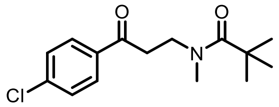

***N*-(3-(4-chlorophenyl)-3-oxopropyl)-*N*-methylpivalamide** **(39).** Prepared according to **GP2** from **1h** (36.0 µL, 0.3 mmol) and **2g** (1.5 mL). Purified via flash column chromatography on silica gel (gradient from Pentane to Pentane:EtOAc 2:1) to afford the product as a colourless oil (25.0 mg, 29% yield).

^{1}H NMR (300 MHz, $CDCl_3$) δ 7.95 – 7.90 (m, 2H), 7.46 – 7.41 (m, 2H), 3.71 (t, *J* = 7.1 Hz, 2H), 3.23 (t, *J* = 7.1 Hz, 2H), 3.15 (s, 3H), 1.26 (s, 9H).
^{13}C NMR (75 MHz, $CDCl_3$) δ 198.1, 177.8, 139.9, 135.2, 129.8, 129.1, 47.0, 38.9, 37.7, 36.8, 28.2.
HR-MS (ESI+) m/z $[M+H]^+$ calcd. for $C_{15}H_{20}ClNO_2$ 282.1255, found 282.1262.

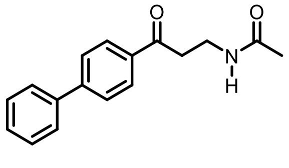

***N*-(3-([1,1'-biphenyl]-4-yl)-3-oxopropyl)acetamide** **(40).** Prepared according to **GP2** from **1b** (54.0 mg, 0.3 mmol) and **2h** (1.5 mL). Purified via flash column chromatography on silica gel (gradient from Pentane:EtOAc 1:1 to 100% EtOAc) to afford the product as a white solid (36.0 mg, 45% yield).

^{1}H NMR (300 MHz, $CDCl_3$) δ 8.02 (d, *J* = 8.5 Hz, 2H), 7.69 (d, *J* = 8.5 Hz, 2H), 7.64 – 7.61 (m, 2H), 7.50 – 7.45 (m, 2H), 7.43 – 7.40 (m, 1H), 6.25 (br. s, 1H), 3.69 (q, *J* = 6.0 Hz, 2H), 3.26 (t, 6.1 Hz, 2H), 1.96 (s, 3H).
^{13}C NMR (75 MHz, $CDCl_3$) δ 199.3, 170.3, 146.4, 139.8, 135.2, 129.1, 128.8, 128.5, 127.5, 127.4, 38.4, 34.5, 23.5.
HR-MS (ESI+) m/z $[M+H]^+$ calcd. for $C_{17}H_{17}NO_2$ 268.1332, found 268.1342.

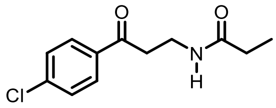

***N*-(3-(4-chlorophenyl)-3-oxopropyl)propionamide** **(41).** Prepared according to **GP2** from **1h** (36.0 µL, 0.3 mmol) and **2i** (1.5 mL). Purified via flash column chromatography on silica gel (gradient from Pentane:EtOAc 1:1 to 100% EtOAc) to afford the product as a white solid (37.0 mg, 51% yield).

^{1}H NMR (300 MHz, $CDCl_3$) δ 7.89 – 7.83 (m, 2H), 7.45 – 7.38 (m, 2H), 6.21 (br. s, 1H), 3.63 (q, J = 5.9 Hz, 2H), 3.17 (t, J = 5.7 Hz, 2H), 2.15 (q, J = 7.6 Hz, 2H), 1.09 (t, J = 7.6 Hz, 3H).
^{13}C NMR (75 MHz, $CDCl_3$) δ 198.4, 174.0, 140.1, 134.9, 129.5, 129.1, 38.4, 34.3, 29.7, 9.9.
HR-MS (ESI+) m/z $[M+H]^+$ calcd. for $C_{12}H_{14}ClNO_2$ 240.0786, found 240.0788.

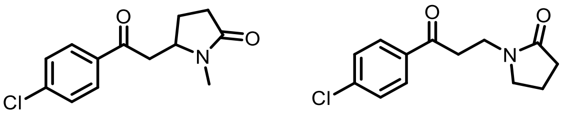

42a *(major)* **42b** *(minor)*

5-(2-(4-chlorophenyl)-2-oxoethyl)-1-methylpyrrolidin-2-one **(42a)** *and* ***1-(3-(4-chlorophenyl)-3-oxopropyl)pyrrolidin-2-one*** **(42b).** Prepared according to **GP2** from **1h** (36.0 µL, 0.3 mmol) and **2j** (1.5 mL). Purified via flash column chromatography on silica gel (gradient from Pentane:EtOAc 1:1 to 100% EtOAc) to afford the two regioisomers as an inseparable mixture as a colourless oil (24.0 mg, 31% yield). *r.r.* ratio of **42** was determined via ^{1}H NMR to be 5.6:1.

Characterization for **42a**
^{1}H NMR (300 MHz, $CDCl_3$) δ 7.88 (d, J = 8.6 Hz, 2H), 7.46 (d, J = 8.7 Hz, 2H), 4.20 – 4.13 (m, 1H), 3.36 (dd, J = 17.2, 4.3 Hz, 1H), 3.00 (dd, J = 17.2, 8.5 Hz, 1H), 2.81 (s, 3H), 2.45 – 2.30 (m, 4H).
^{13}C NMR (75 MHz, $CDCl_3$) δ 196.3, 175.0, 140.3, 135.0, 129.5, 129.3, 56.3, 42.3, 29.8, 28.2, 25.3.

Characterization for **42b**
^{1}H NMR (300 MHz, $CDCl_3$) δ 7.88 (d, J = 8.6 Hz, 2H), 7.46 (d, J = 8.7 Hz, 2H), 3.67 (t, J = 6.6 Hz, 2H), 3.48 – 3.43 (m, 2H), 3.23 (t, J = 6.6 Hz, 2H), 2.05 – 1.91 (m, 2H), 1.71 – 1.66 (m, 2H).
^{13}C NMR (75 MHz, $CDCl_3$) δ 197.4, 175.5, 140.0, 135.0, 129.6, 129.1, 48.6, 38.4, 36.8, 31.1, 18.3.
HR-MS (ESI+) m/z $[M+H]^+$ calcd. for $C_{13}H_{14}ClNO_2$ 252.0786, found 252.0792.

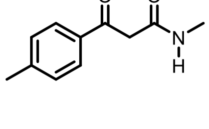

***N*-methyl-3-oxo-3-(p-tolyl)propanamide** **(43).** Prepared according to **GP2** from **1r** (40.0 µL, 0.3 mmol) and **2k** (1.5 mL). Purified via flash column chromatography on silica gel (gradient from Pentane:EtOAc 1:1 to 100% EtOAc) to afford the product as a white solid (17.0 mg, 30% yield).

^{1}H NMR (300 MHz, $CDCl_3$) δ 7.89 (d, J = 8.3 Hz, 2H), 7.28 (d, J = 8.6 Hz, 2H), 3.92 (s, 2H), 2.85 (d, J = 4.8 Hz, 3H), 2.42 (s, 3H).
^{13}C NMR (75 MHz, $CDCl_3$) δ 196.1, 166.6, 145.3, 133.9, 129.7, 128.8, 45.1, 26.5, 21.9.
HR-MS (ESI+) m/z $[M+H]^+$ calcd. for $C_{11}H_{13}NO_2$ 192.1019, found 192.1021.

12. Limitations of the scope

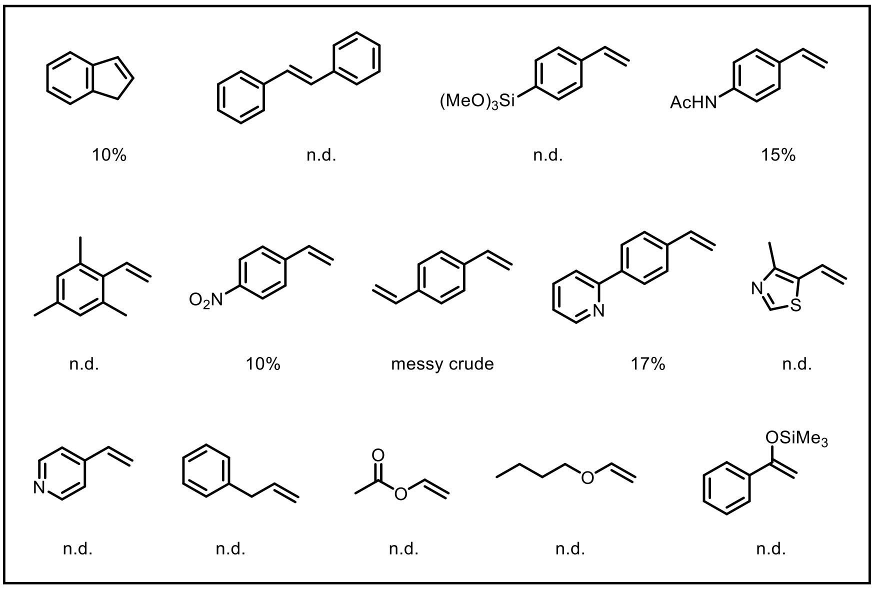

Figure S17: Olefins in the oxo-amidomethylation which were unsuccessful when subjected to the optimized reaction conditions. ^{1}H NMR yields determined using 1,1,2-trichloroethylene as an internal standard.

The reaction with 4-vinylpyridine was also investigated using a weaker acid and under acid-free conditions, as described in **Table S11**. The results show that the target product was not formed in any of the entries. Therefore, it was concluded that the presence of HCl in the system was not responsible for the lack of target product formation.

Table S11: Acid screening for the reaction with 4-vinylpyridine as radical acceptor.

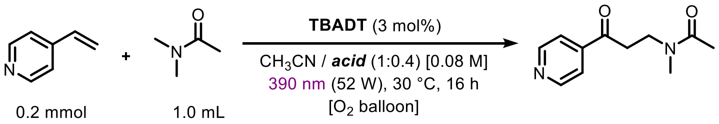

Entry	Acid	Conversion (%)[a]	Yield of 3 (%)[a]
1	1 M HCl	>95	n.d.
2	1 M AcOH	>95	n.d.
3	-	>95	n.d.

[a] Determined via ^{1}H NMR using 1,1,2-trichloroethylene as an internal standard.

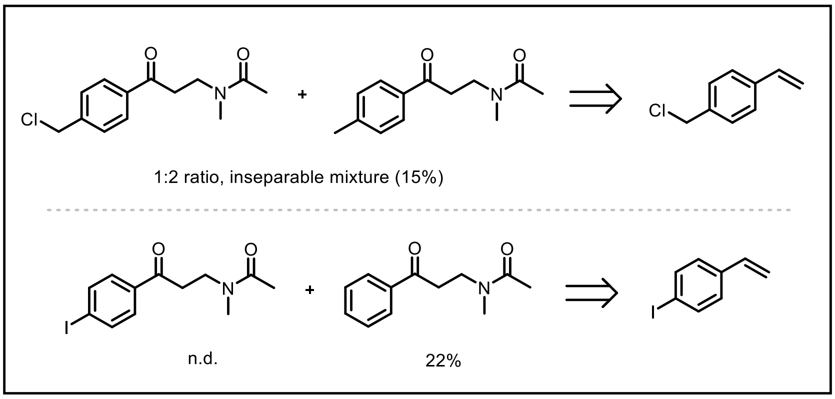

Figure S18: Results obtained by subjecting 1-(chloromethyl)-4-vinylbenzene *(top)* and 1-iodo-4-vinylbenzene *(bottom)* to standard oxo-amidomethylation reaction conditions. ^{1}H NMR yields determined using 1,1,2-trichloroethylene as an internal standard.

Although detailed studies have not been conducted, a possible explanation is that, once formed, the target products shown in **Figure S18** are reduced by $2H^+[W_{10}O_{32}]^{6-}$ (E_{red} = −1.48 V *vs* SCE),[23–25] generated via disproportionation of $H^+[W_{10}O_{32}]^{5-}$.

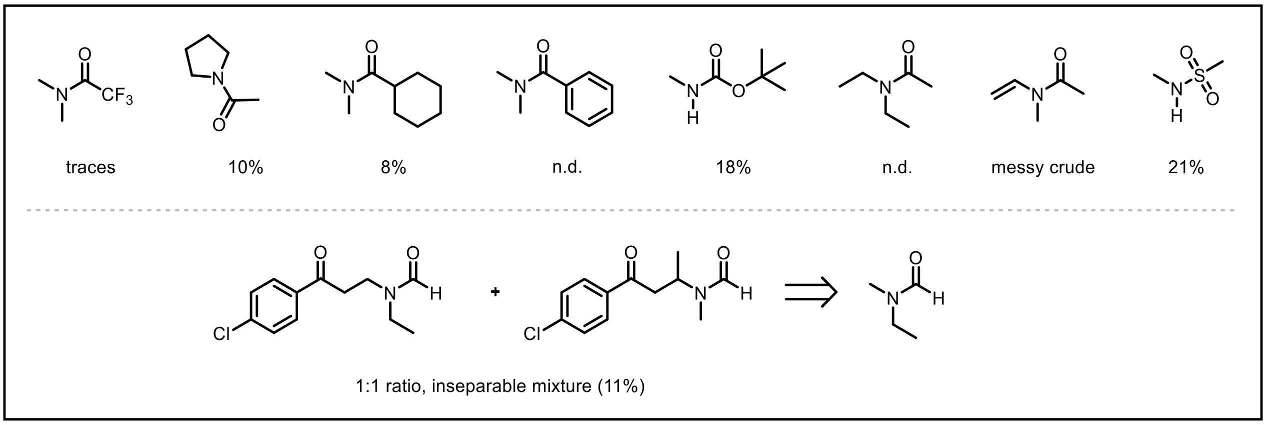

Figure S19: Amides in the oxo-amidomethylation which were unsuccessful when subjected to the optimized reaction conditions. ^{1}H NMR yields determined using 1,1,2-trichloroethylene as an internal standard.

13. NMR spectra of oxo-amidomethylation products

^{1}H NMR (300 MHz, $CDCl_3$) of **3**

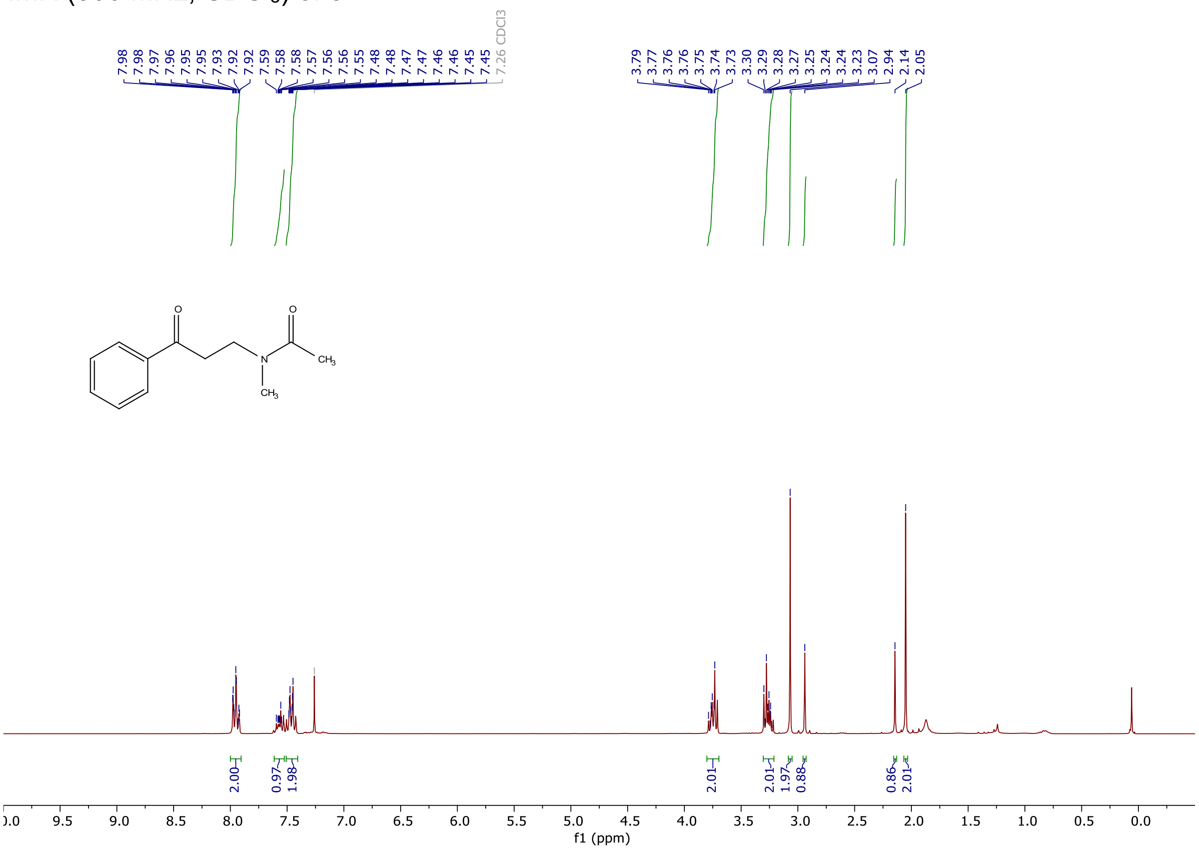

^{13}C NMR (75 MHz, $CDCl_3$) of **3**

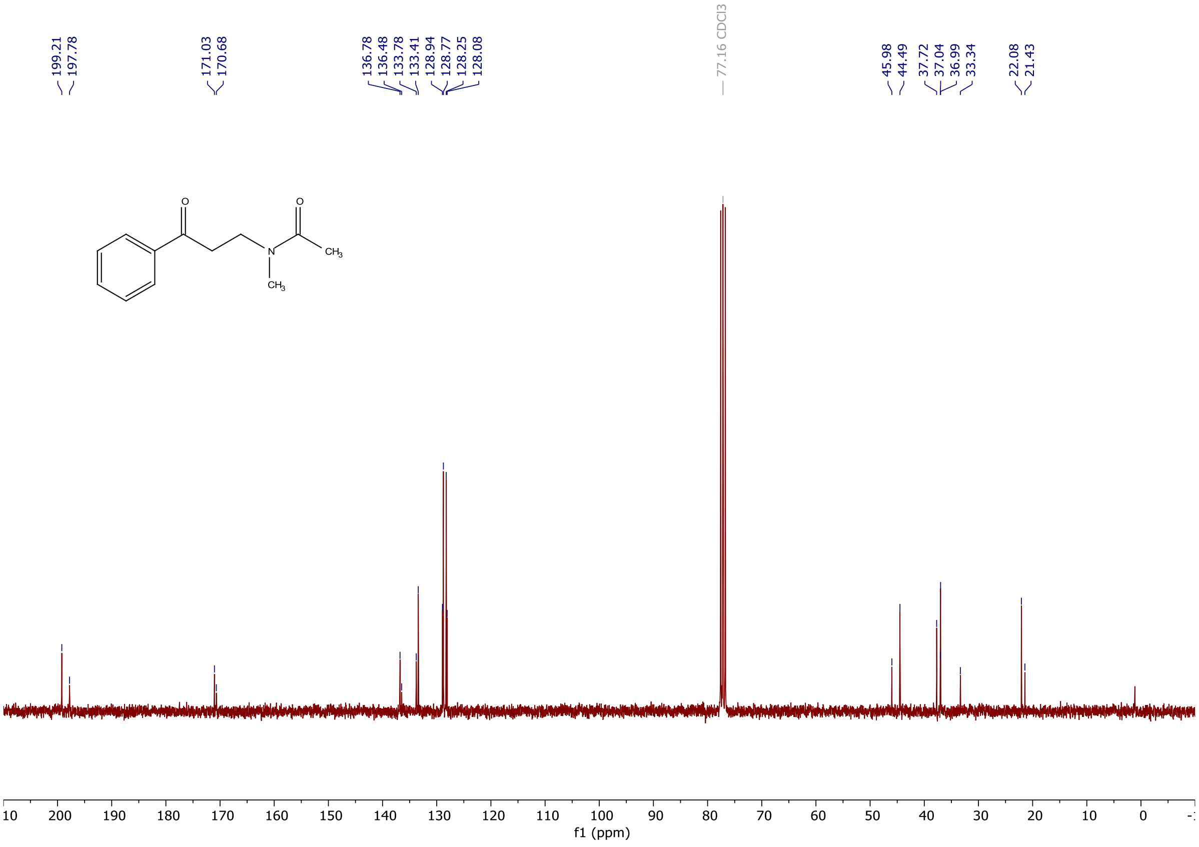

^{1}H NMR (300 MHz, $CDCl_3$) of **4**

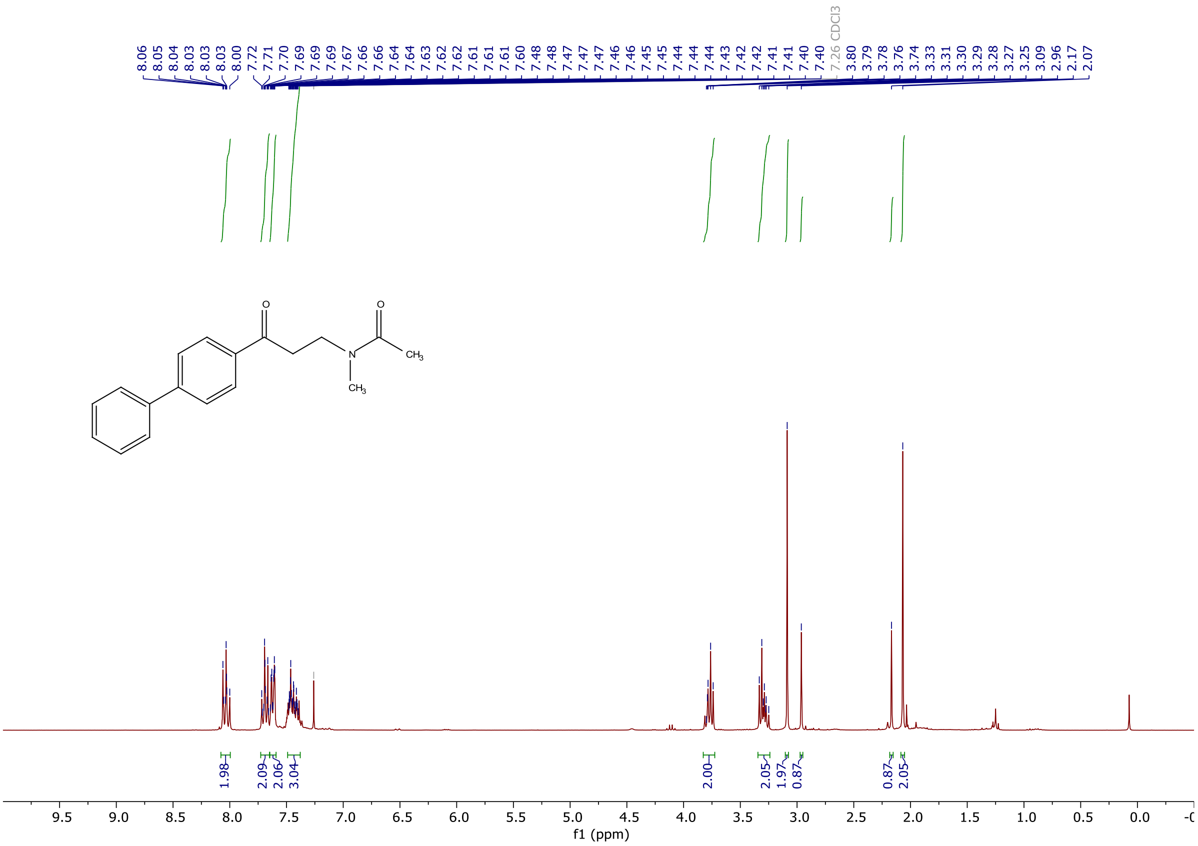

^{13}C NMR (75 MHz, $CDCl_3$) of **4**

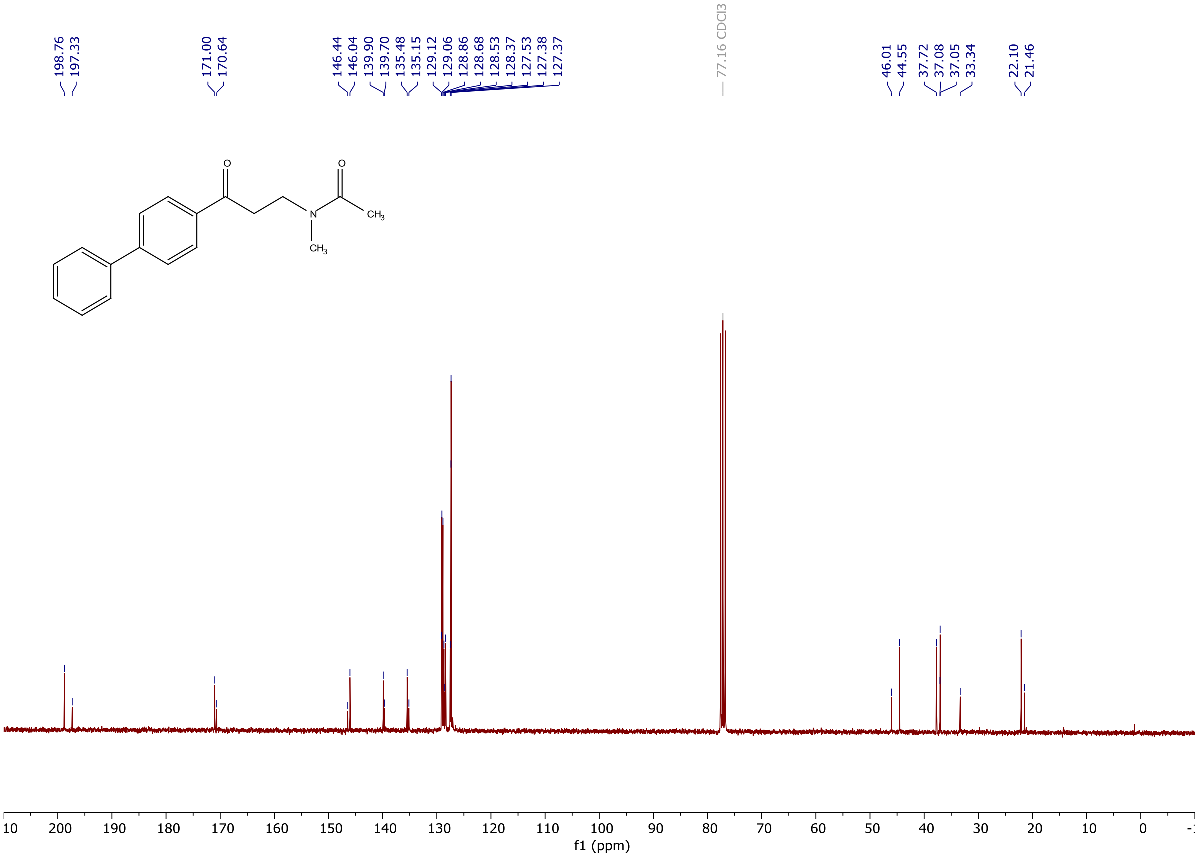

^{1}H NMR (300 MHz, $CDCl_3$) of **5**

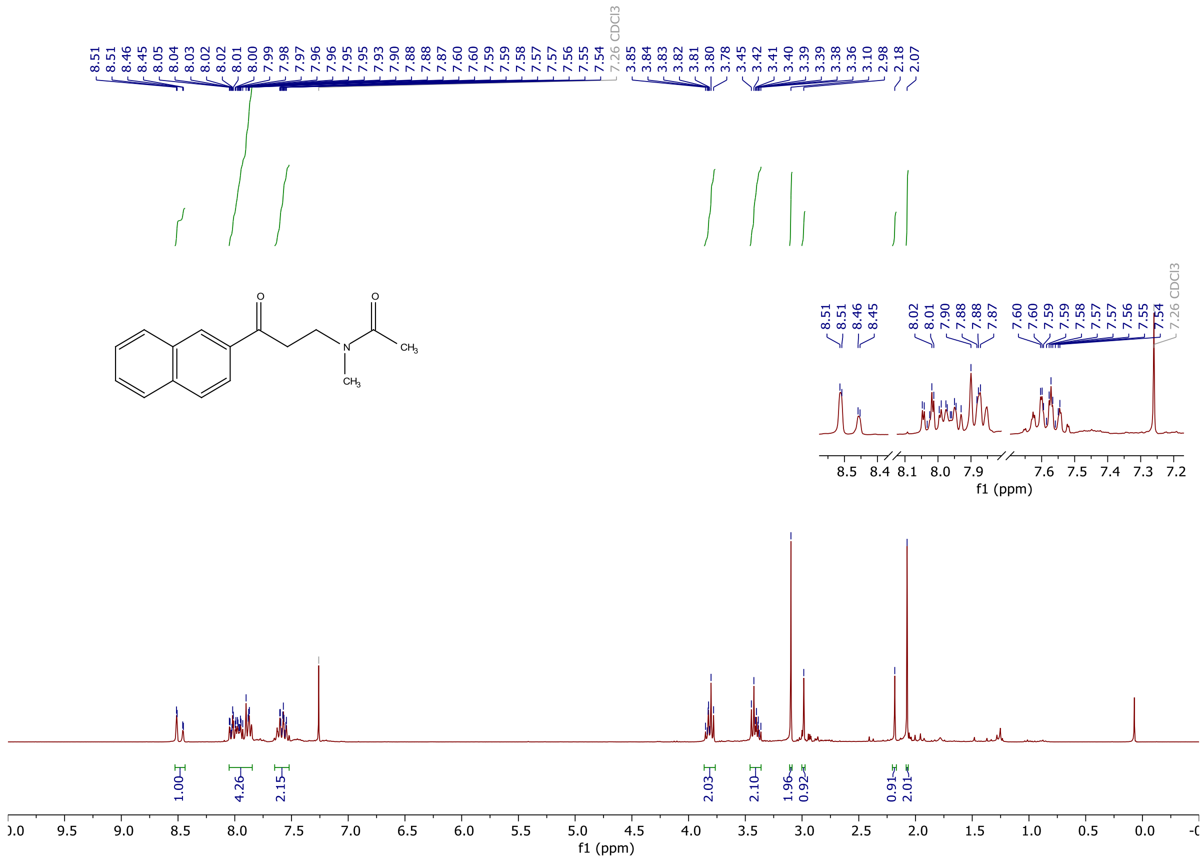

^{13}C NMR (75 MHz, $CDCl_3$) of **5**

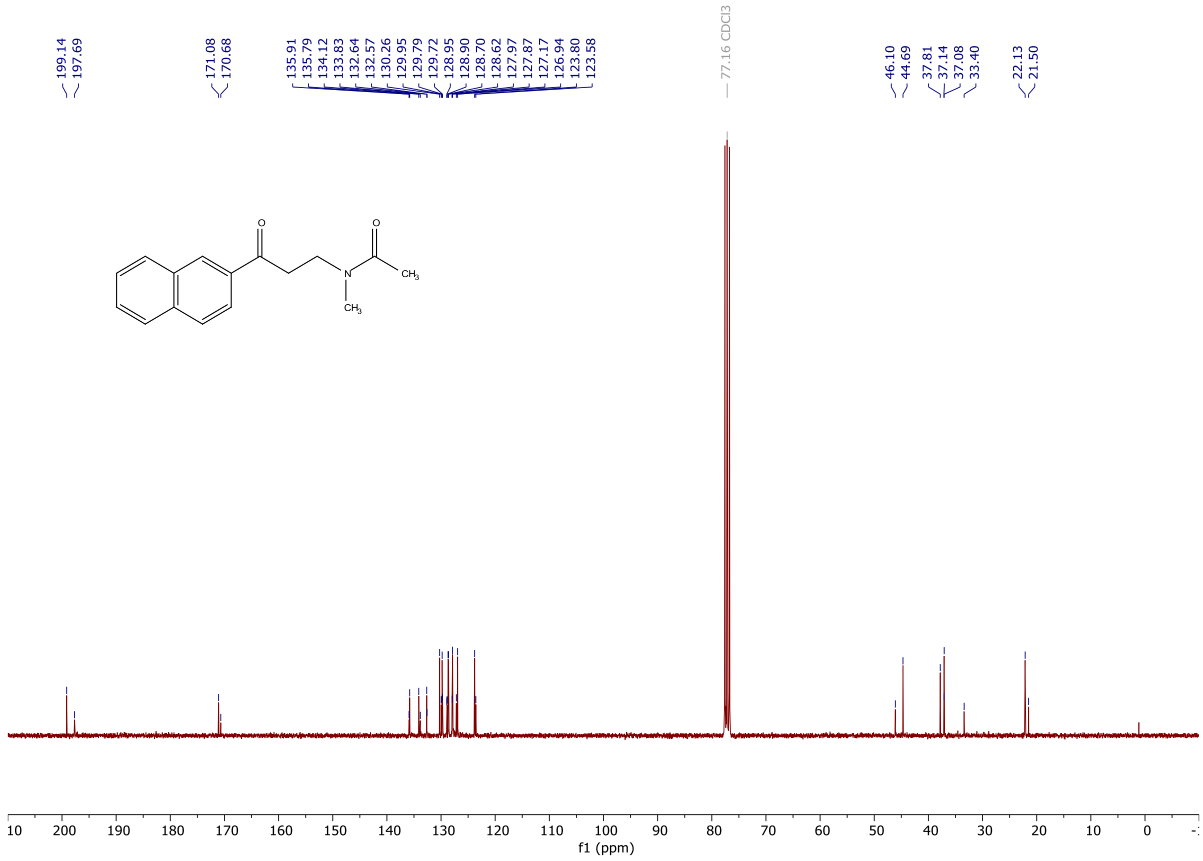

^{1}H NMR (300 MHz, $CDCl_3$) of **6**

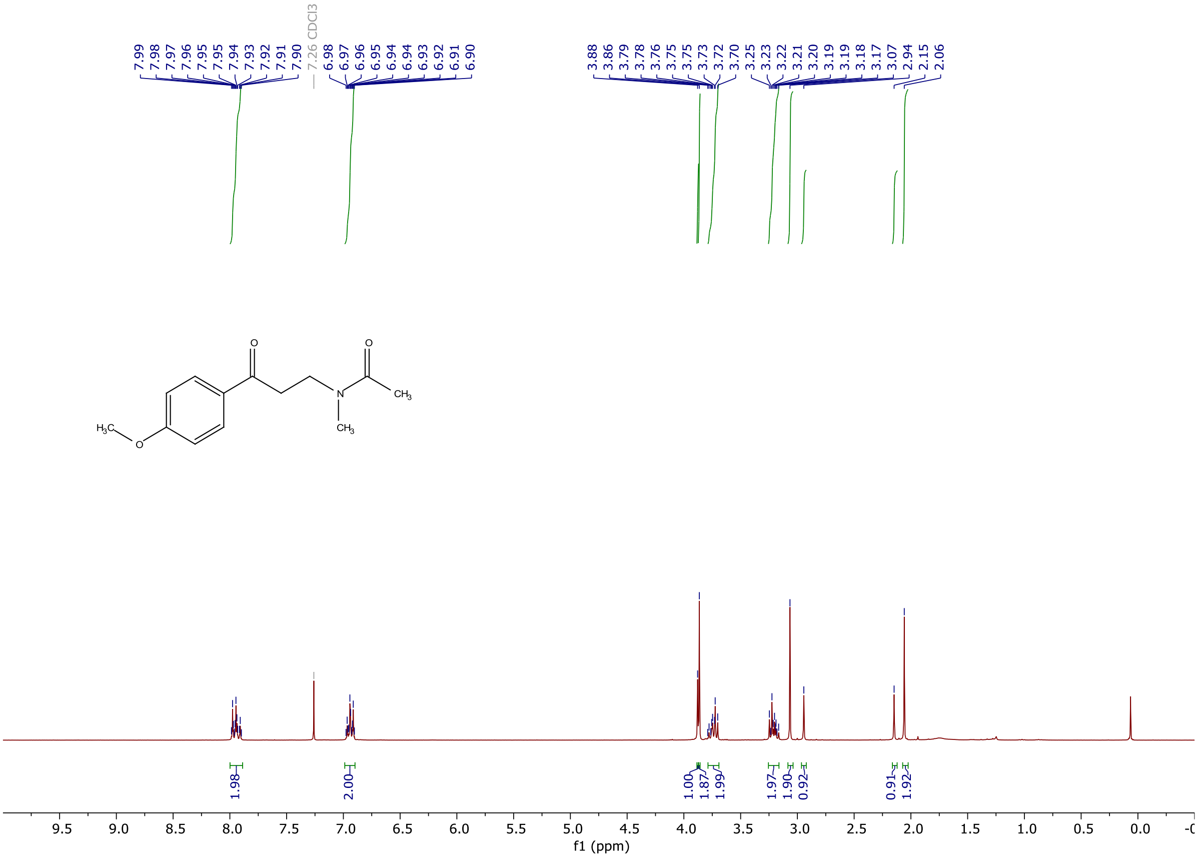

^{13}C NMR (75 MHz, $CDCl_3$) of **6**

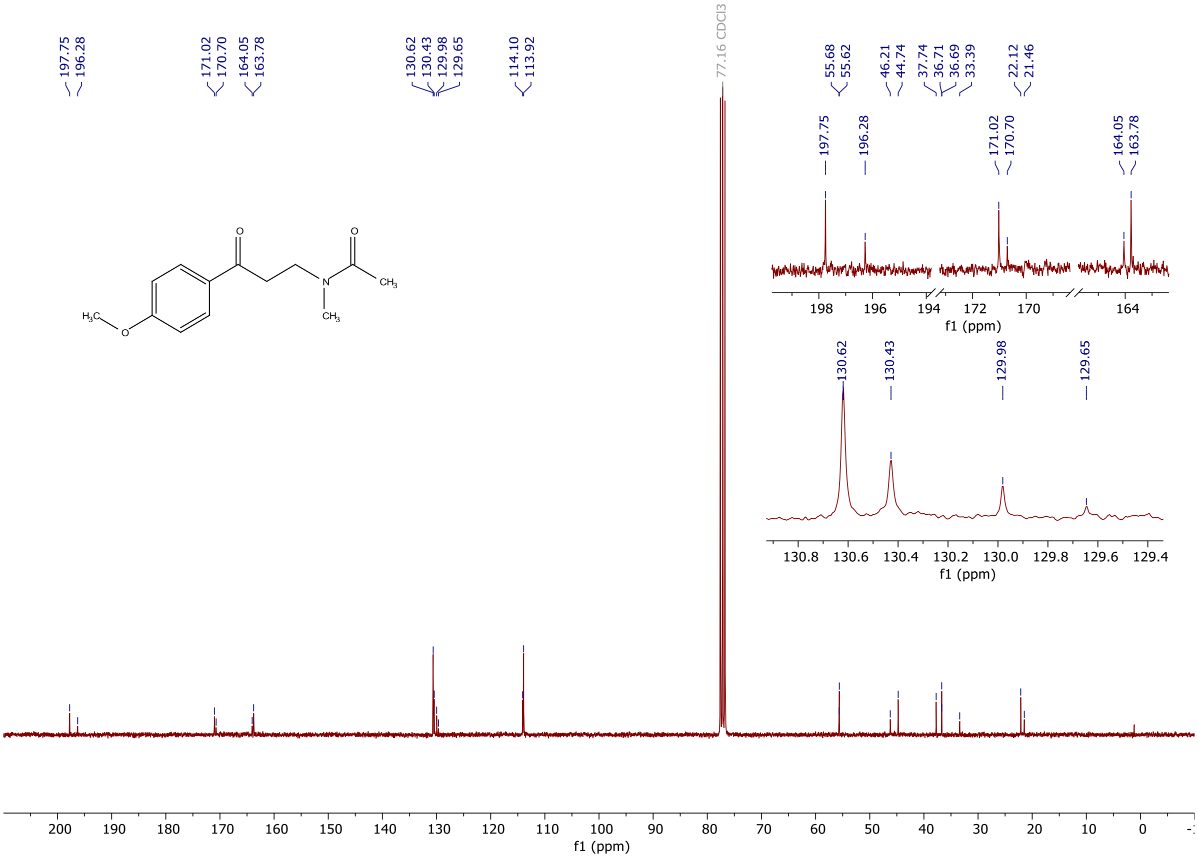

^{1}H NMR (300 MHz, $CDCl_3$) of **7**

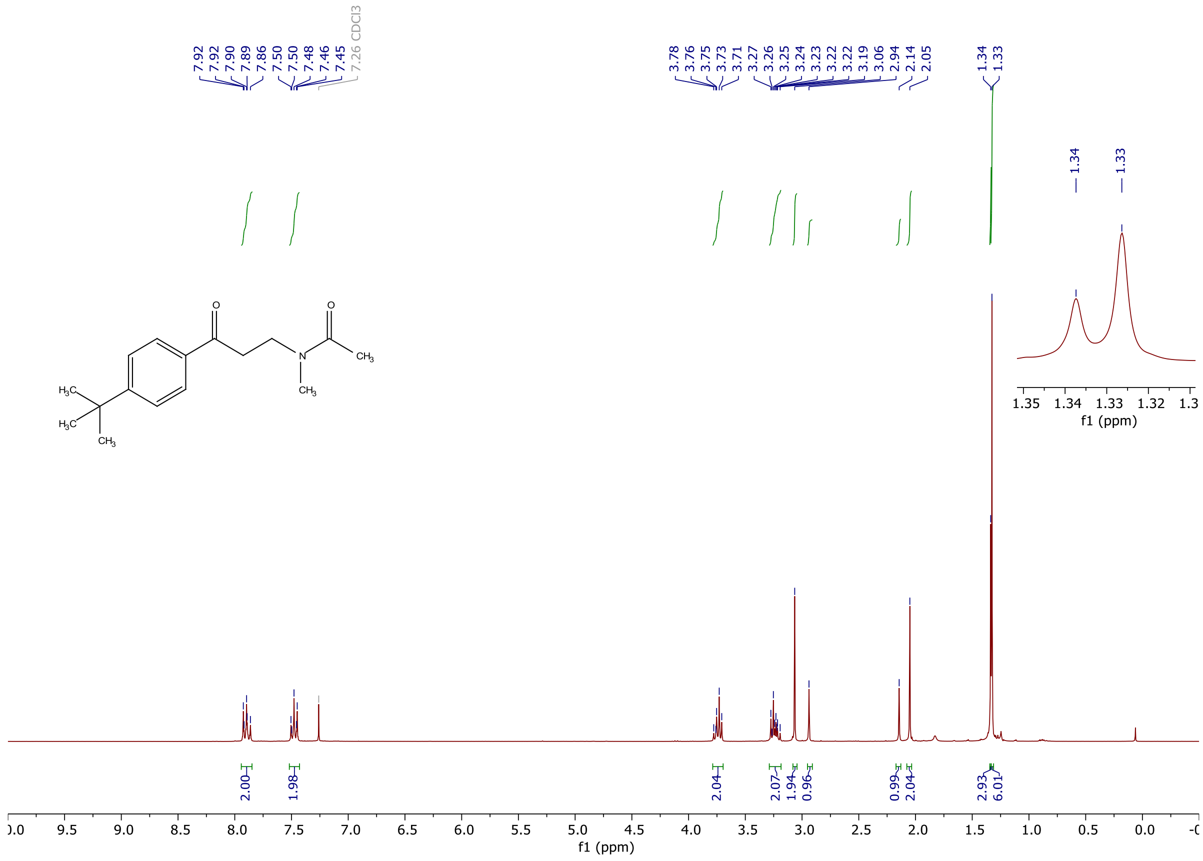

^{13}C NMR (75 MHz, $CDCl_3$) of **7**

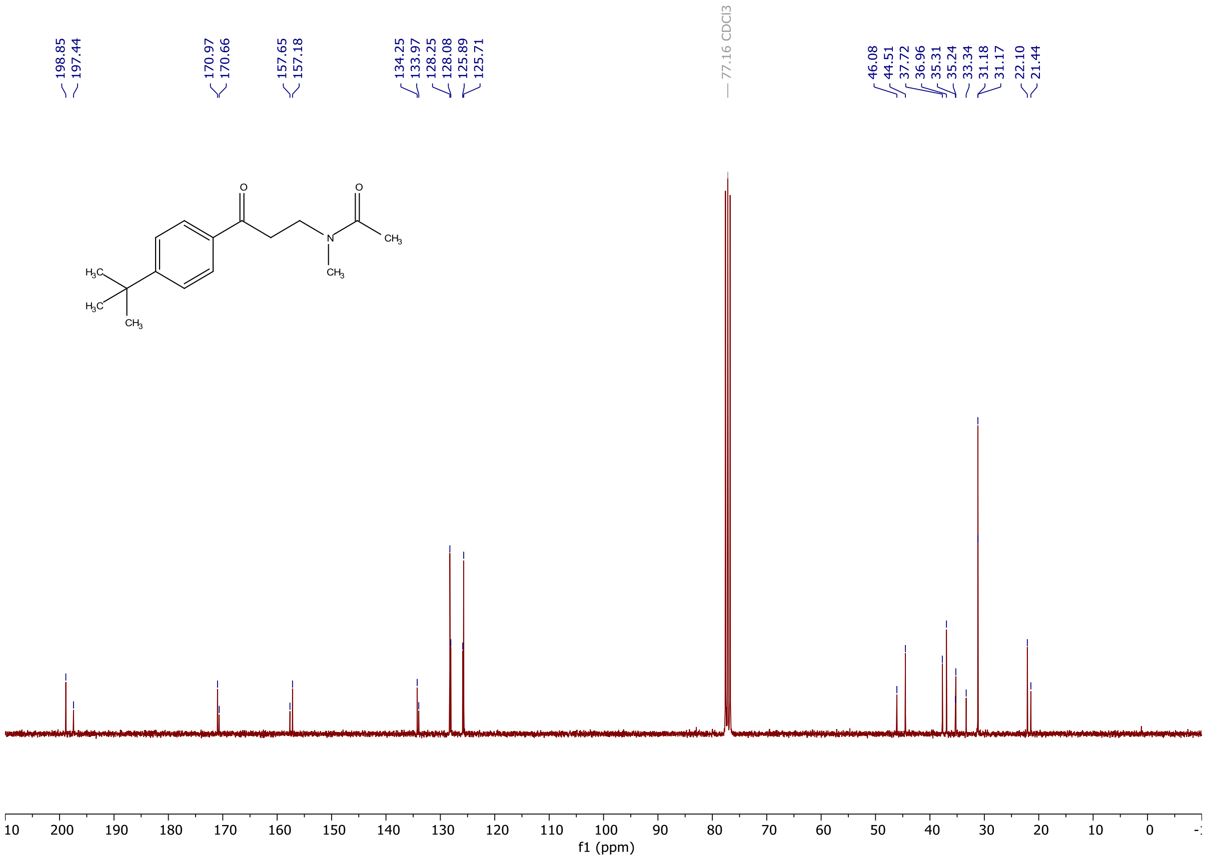

^{1}H NMR (300 MHz, $CDCl_3$) of **8**

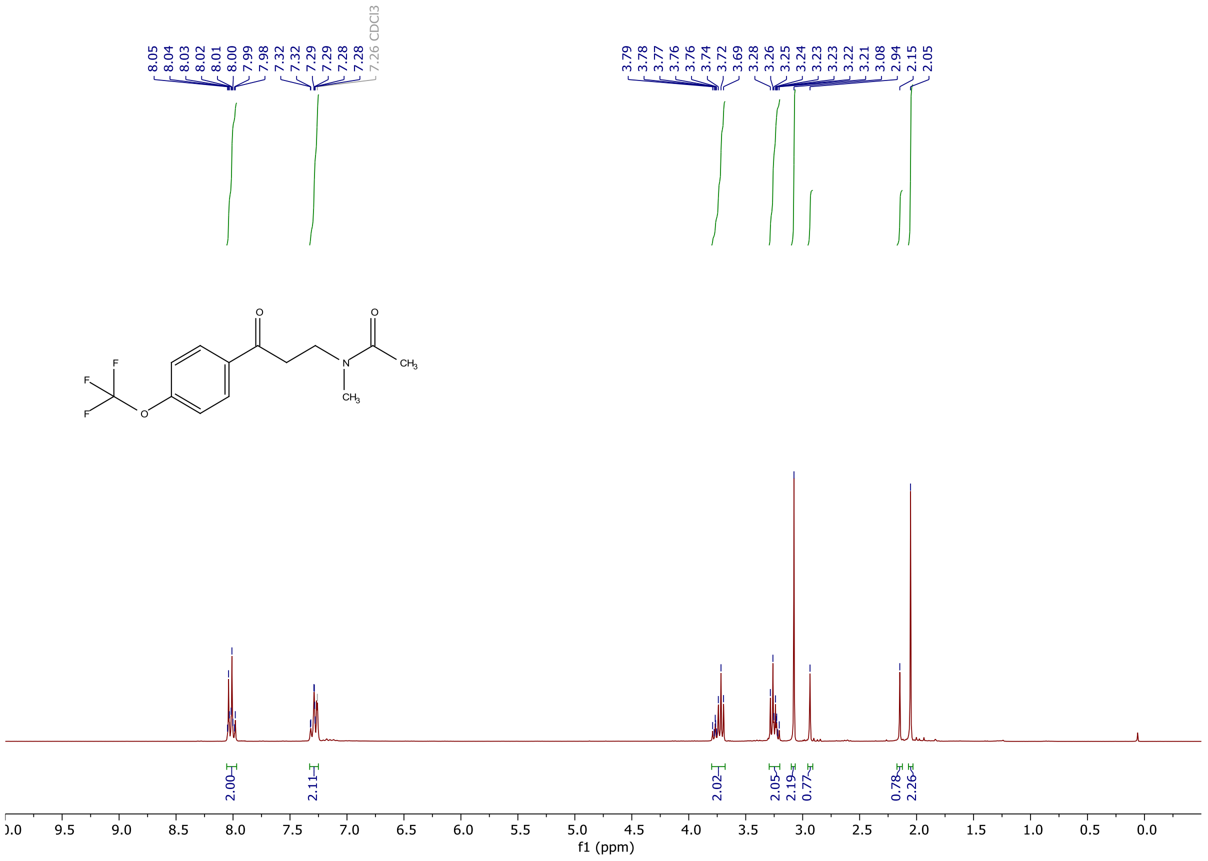

^{13}C NMR (75 MHz, $CDCl_3$) of **8**

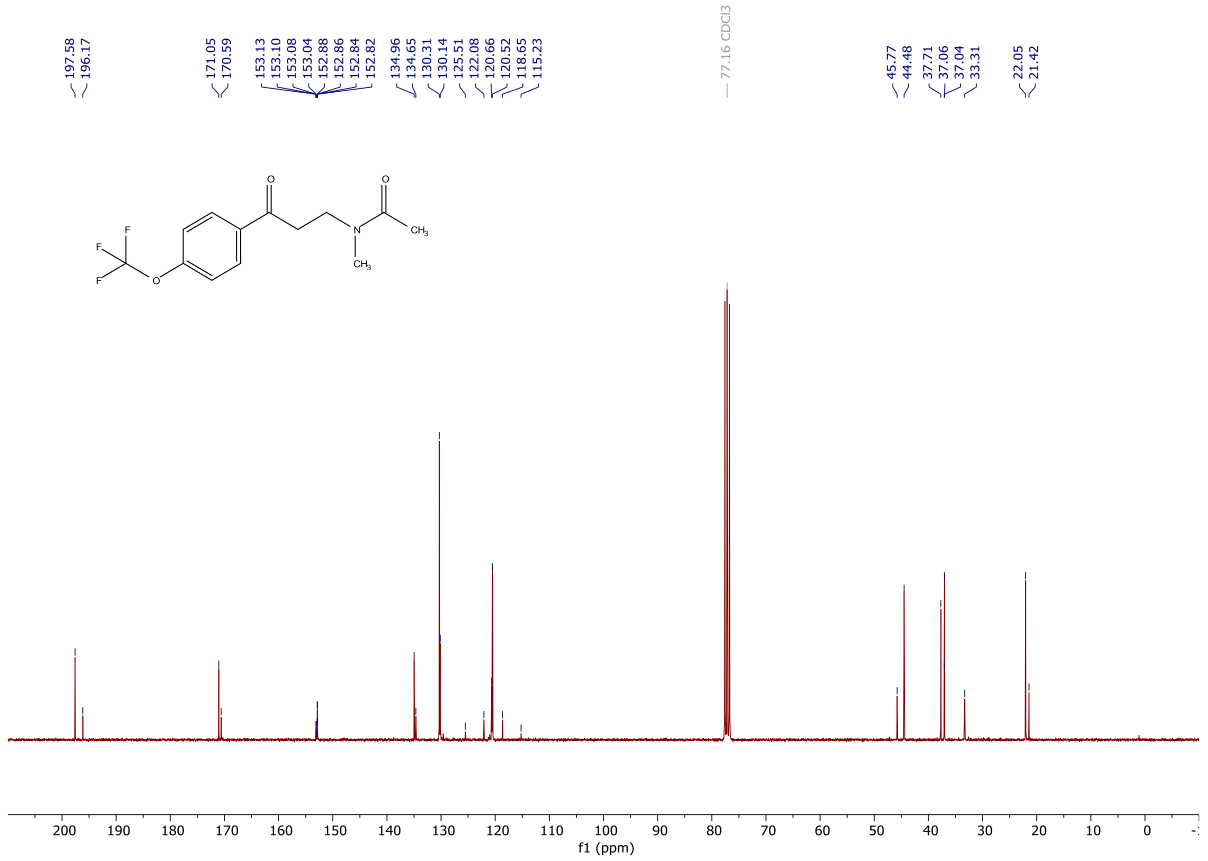

^{19}F NMR (282 MHz, $CDCl_3$) of **8**

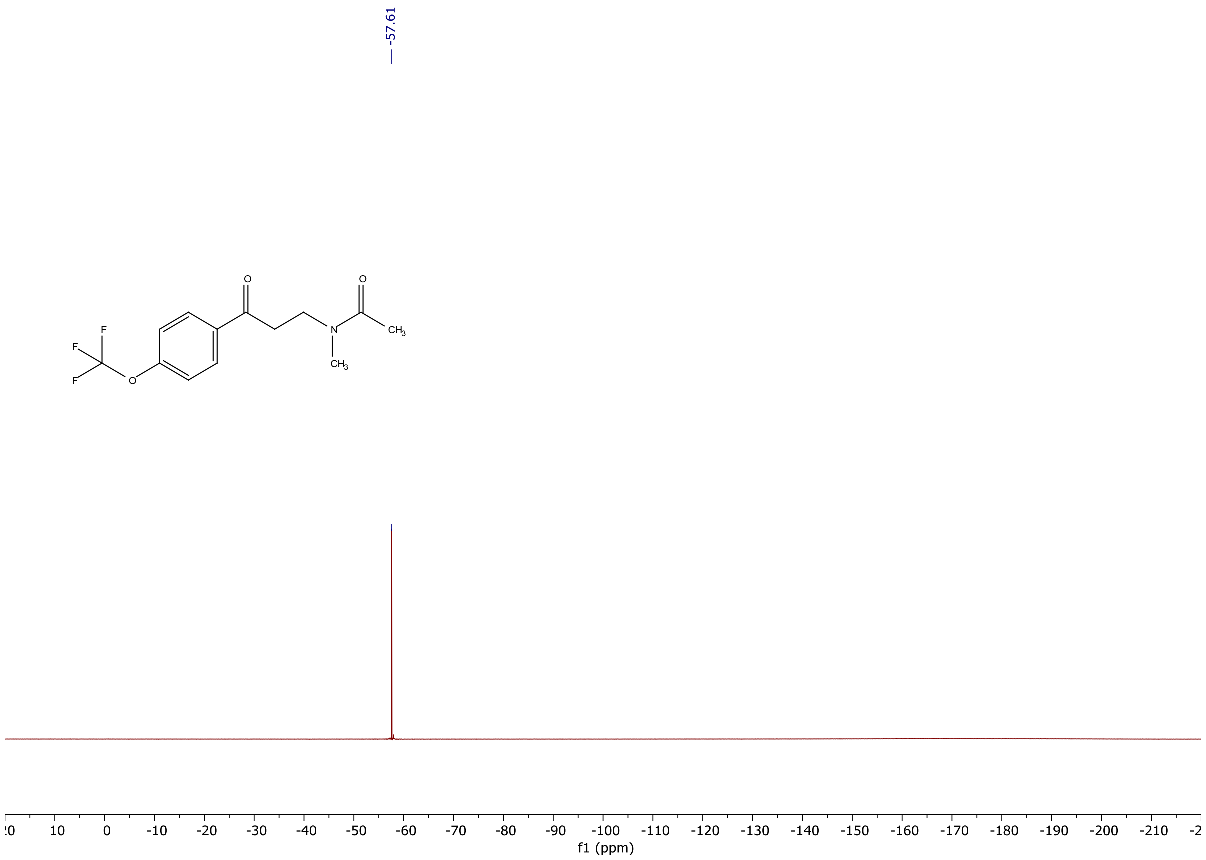

^{1}H NMR (300 MHz, $CDCl_3$) of **9**

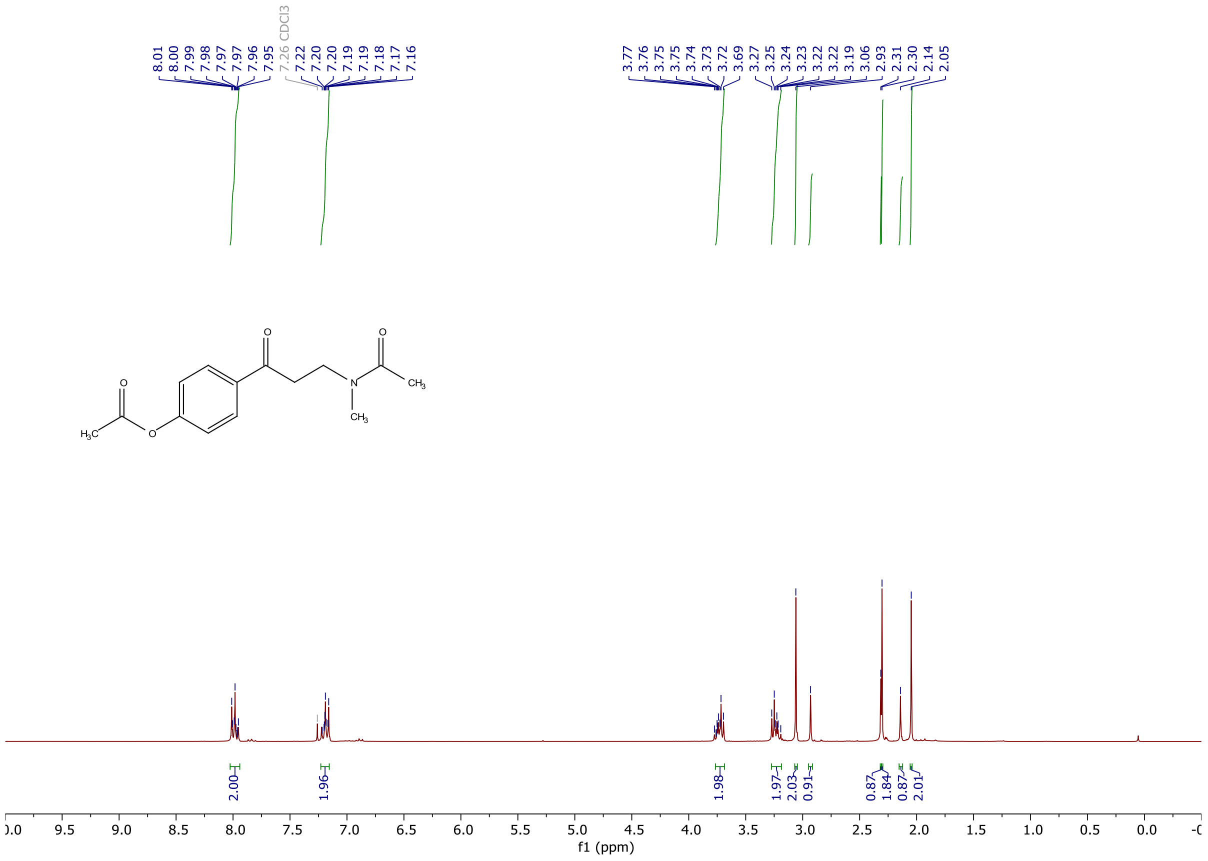

^{13}C NMR (75 MHz, $CDCl_3$) of **9**

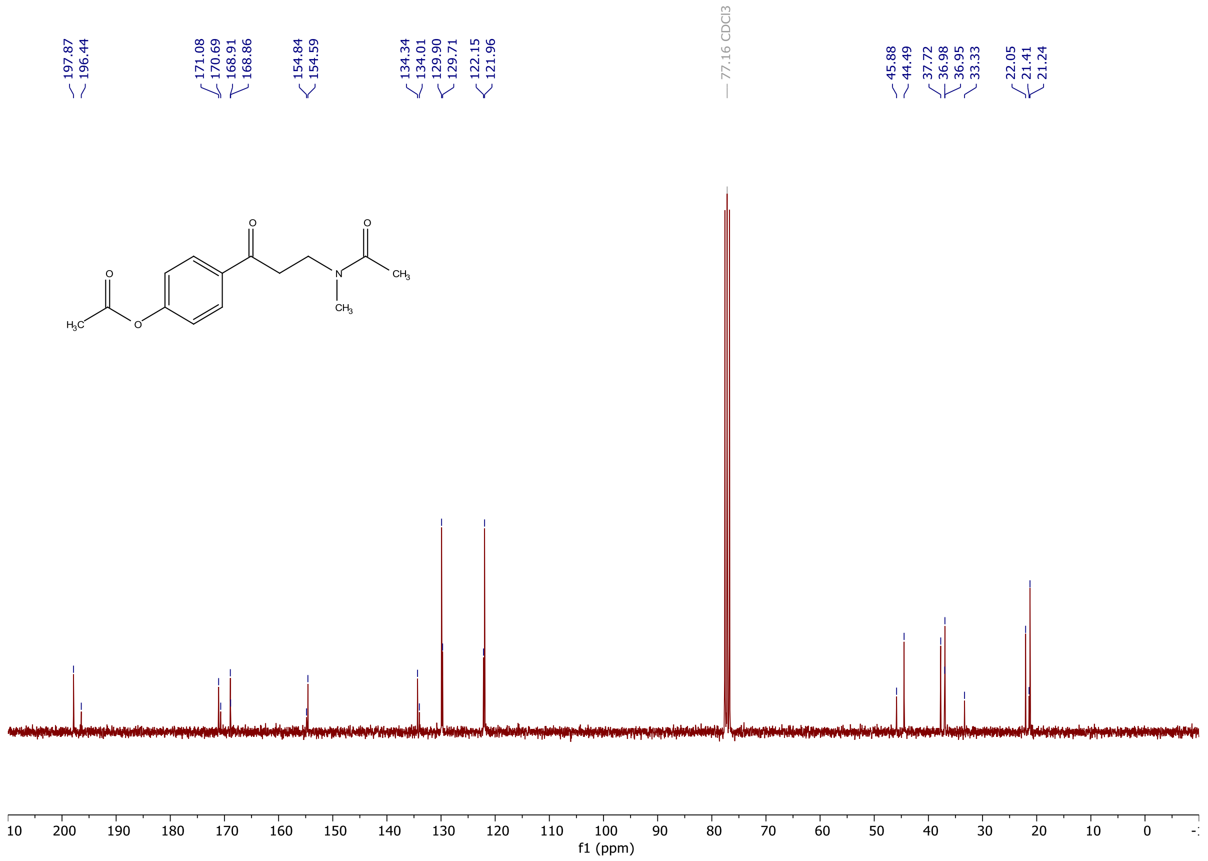

^{1}H NMR (300 MHz, $CDCl_3$) of **10**

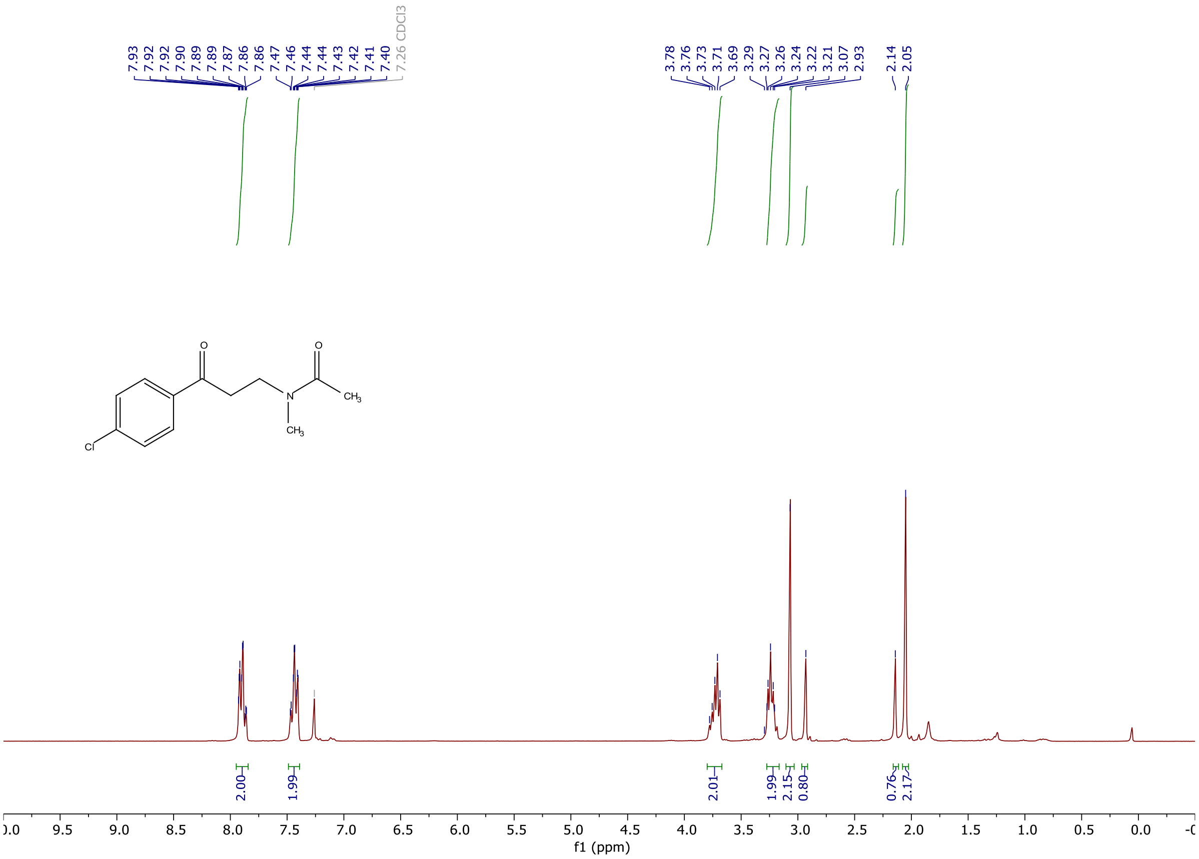

^{13}C NMR (75 MHz, $CDCl_3$) of **10**

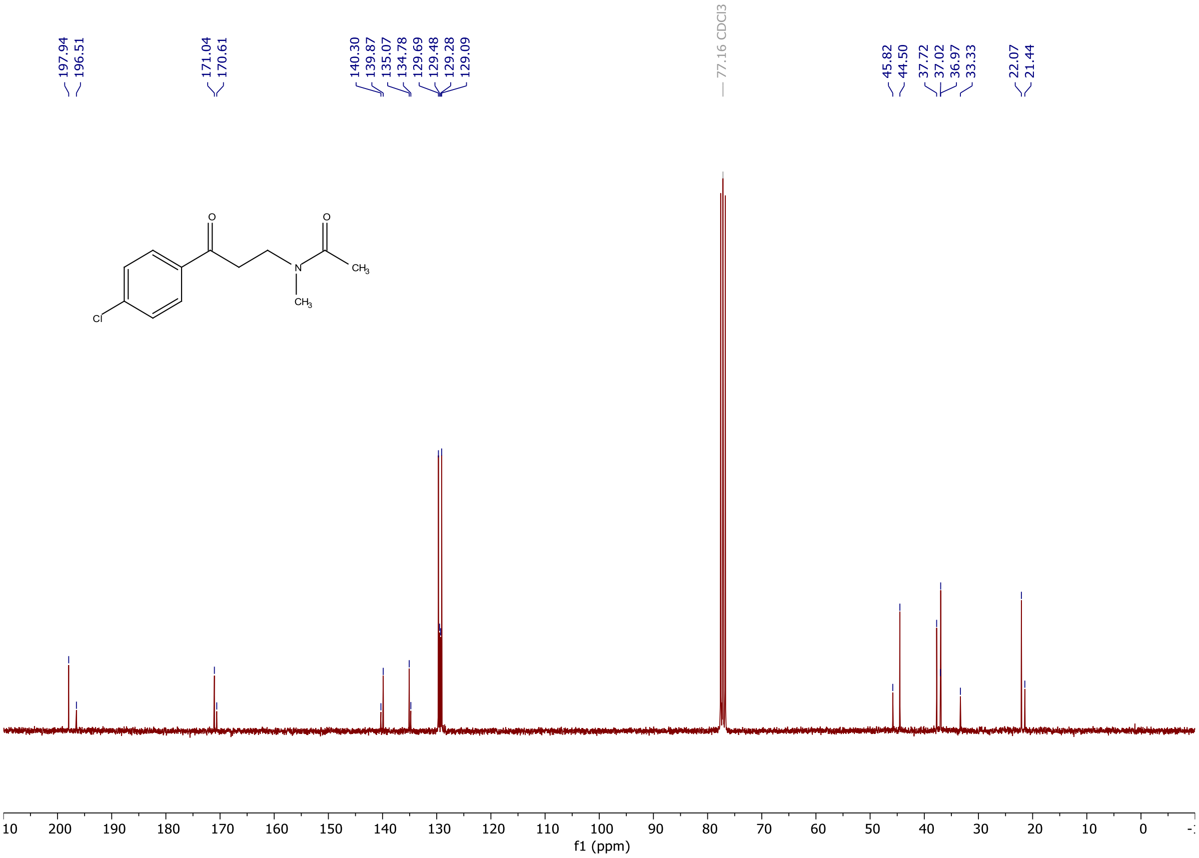

^{1}H NMR (300 MHz, $CDCl_3$) of **11**

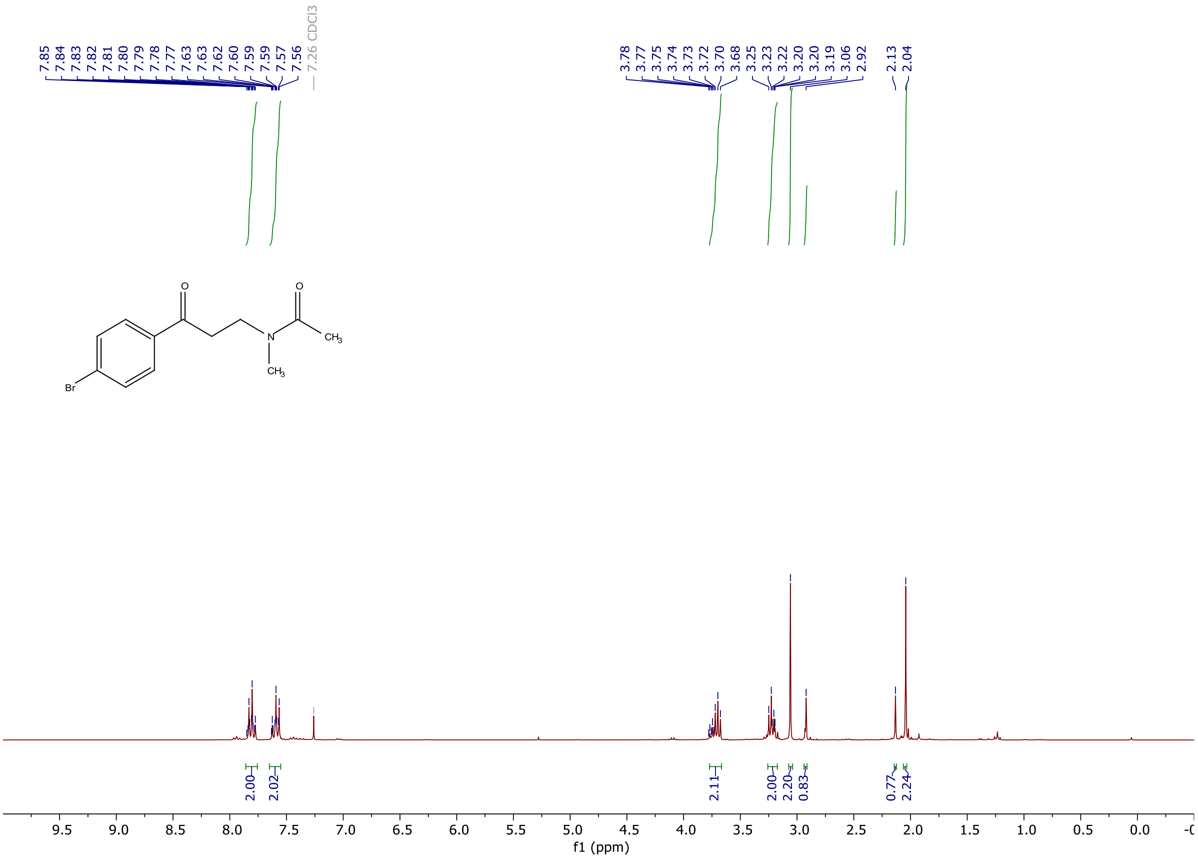

^{13}C NMR (75 MHz, $CDCl_3$) of **11**

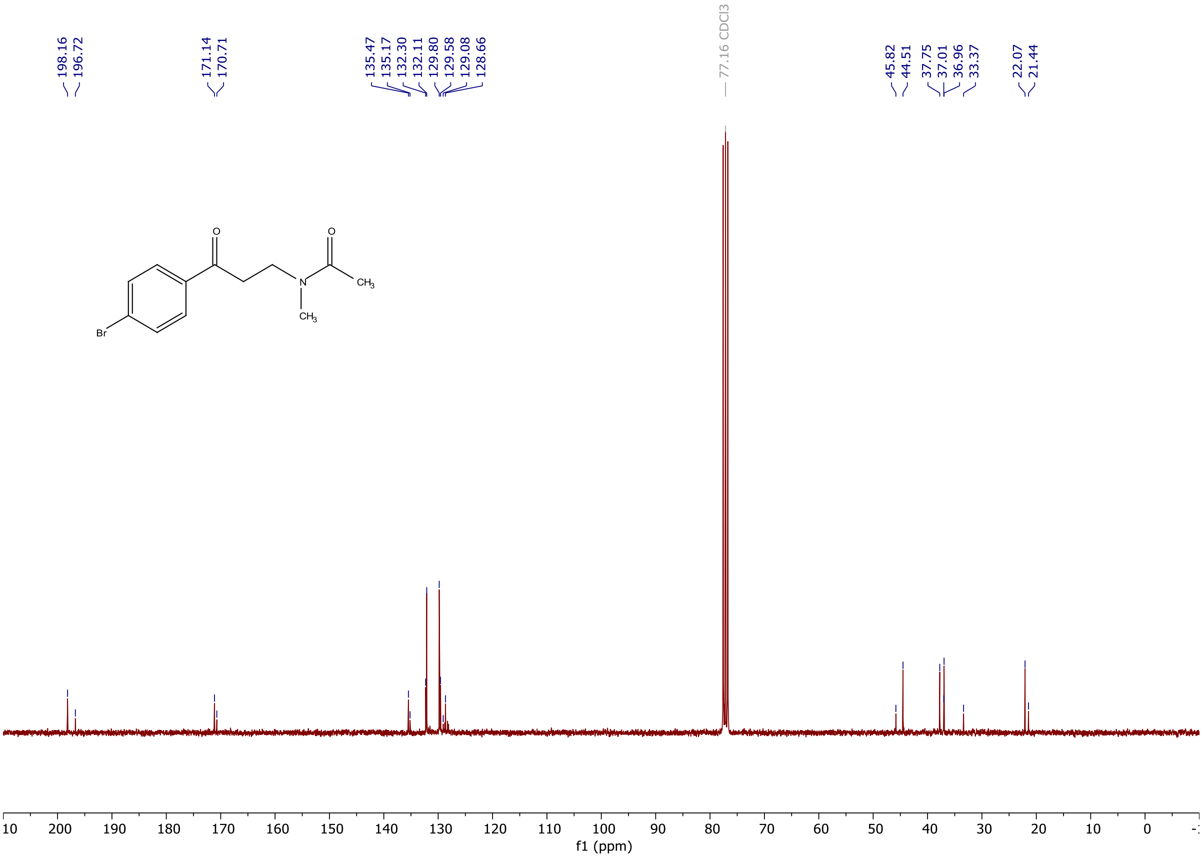

^{1}H NMR (300 MHz, $CDCl_3$) of **12**

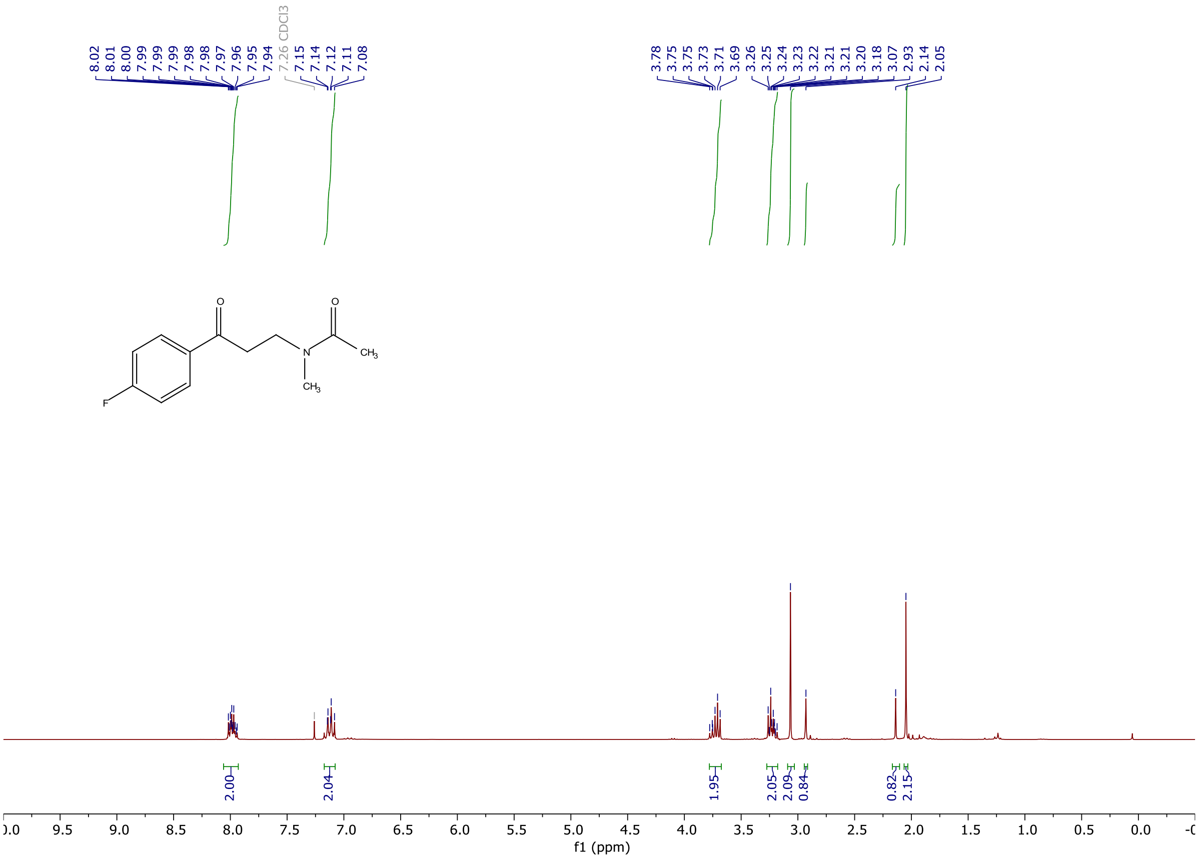

^{13}C NMR (75 MHz, $CDCl_3$) of **12**

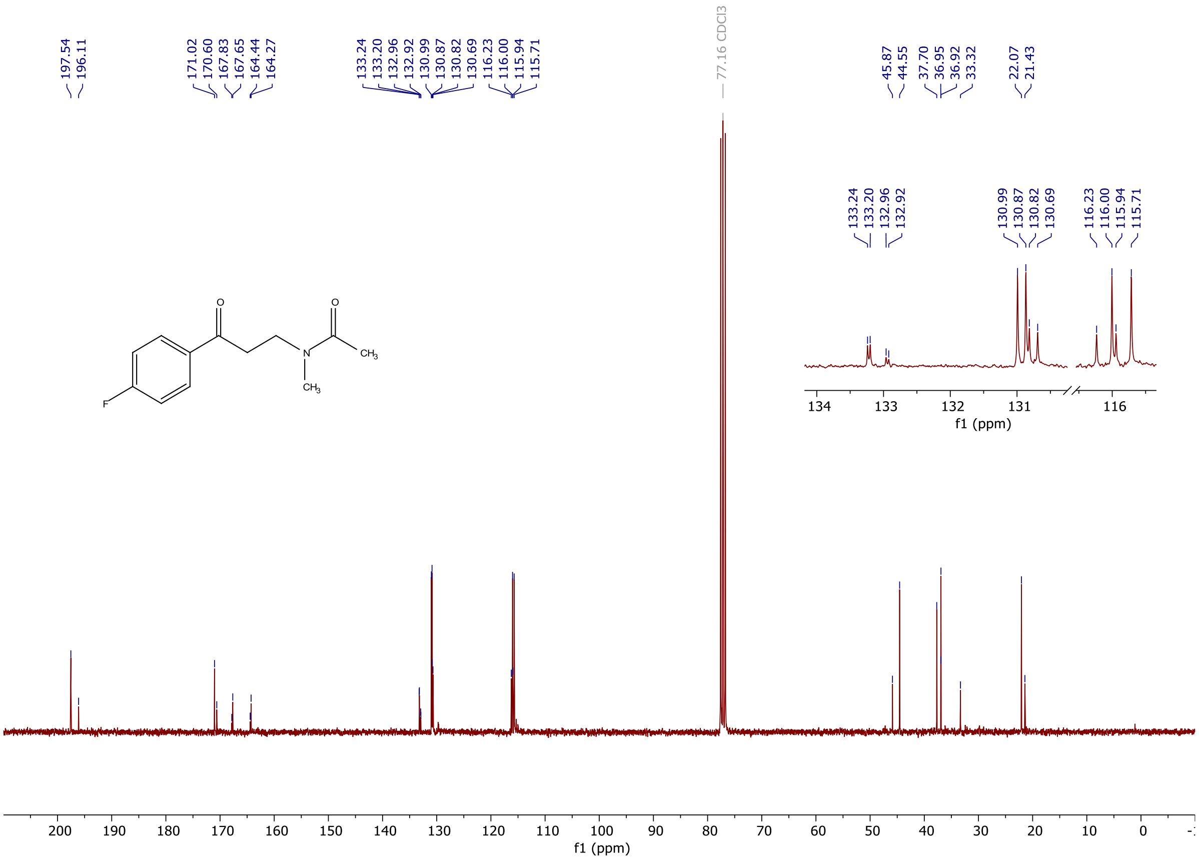

^{19}F NMR (282 MHz, $CDCl_3$) of **12**

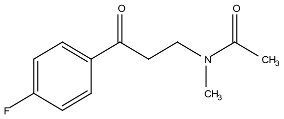

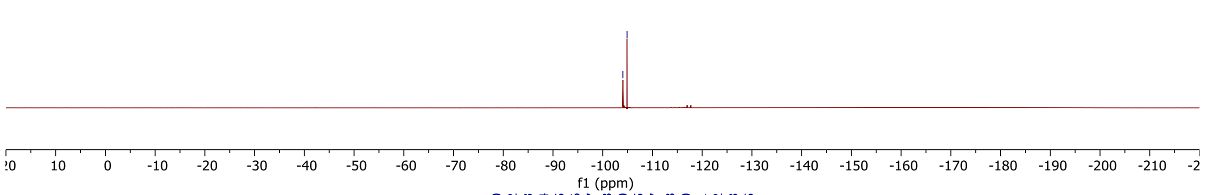

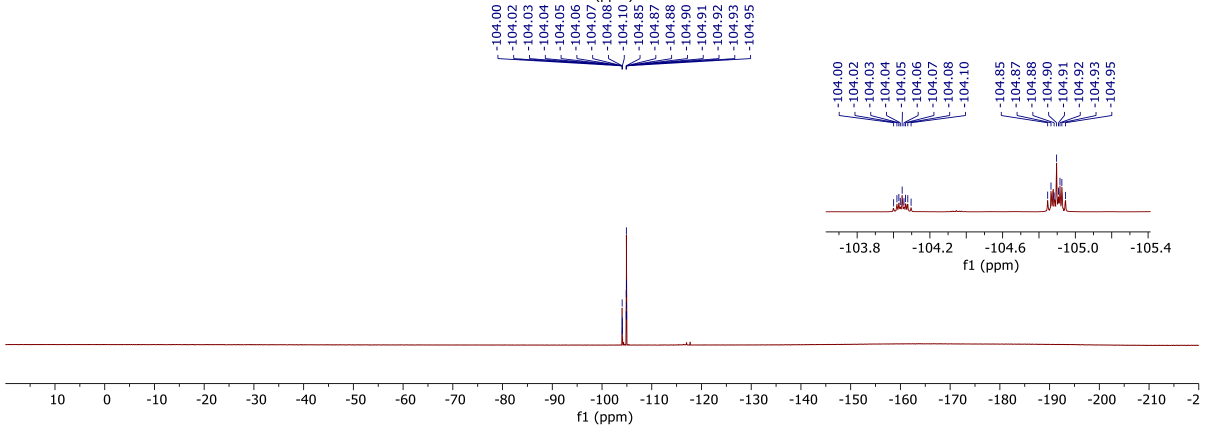

^{1}H NMR (300 MHz, $CDCl_3$) of **13**

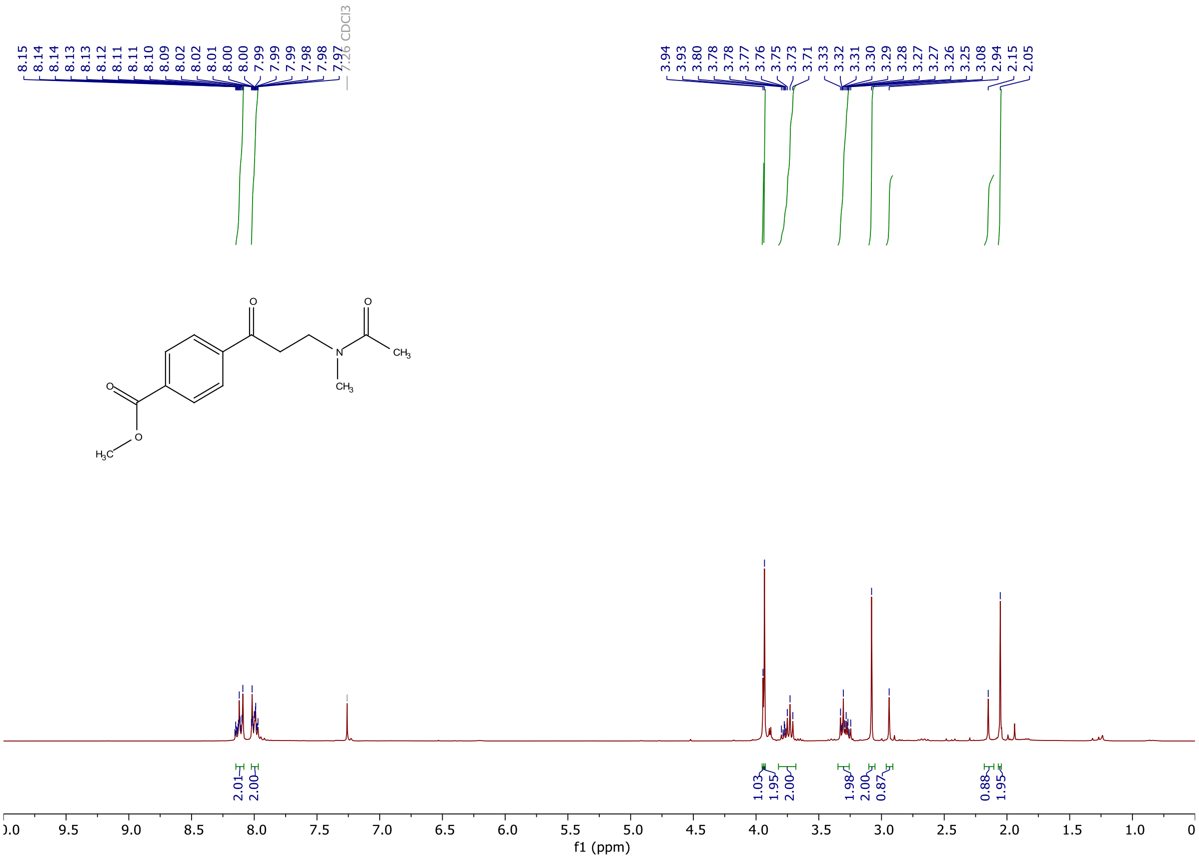

^{13}C NMR (75 MHz, $CDCl_3$) of **13**

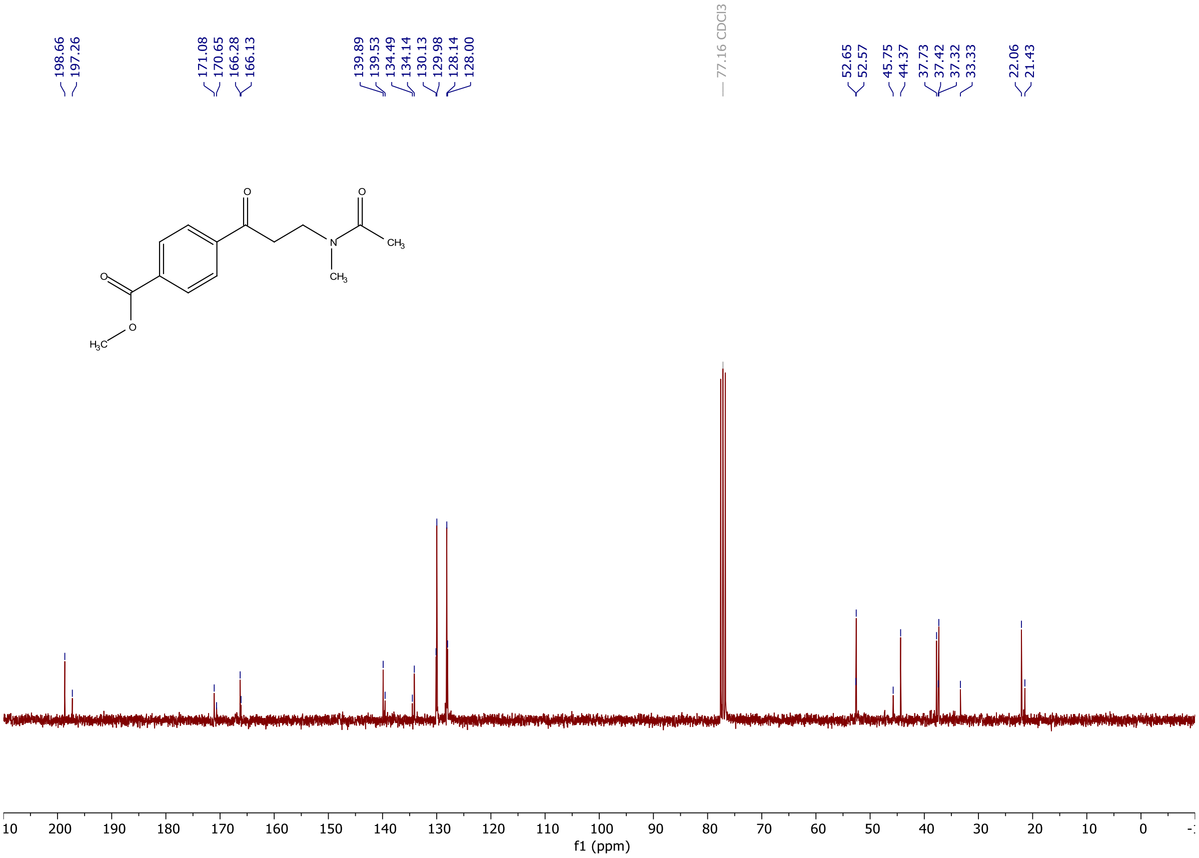

^{1}H NMR (300 MHz, $CDCl_3$) of **14**

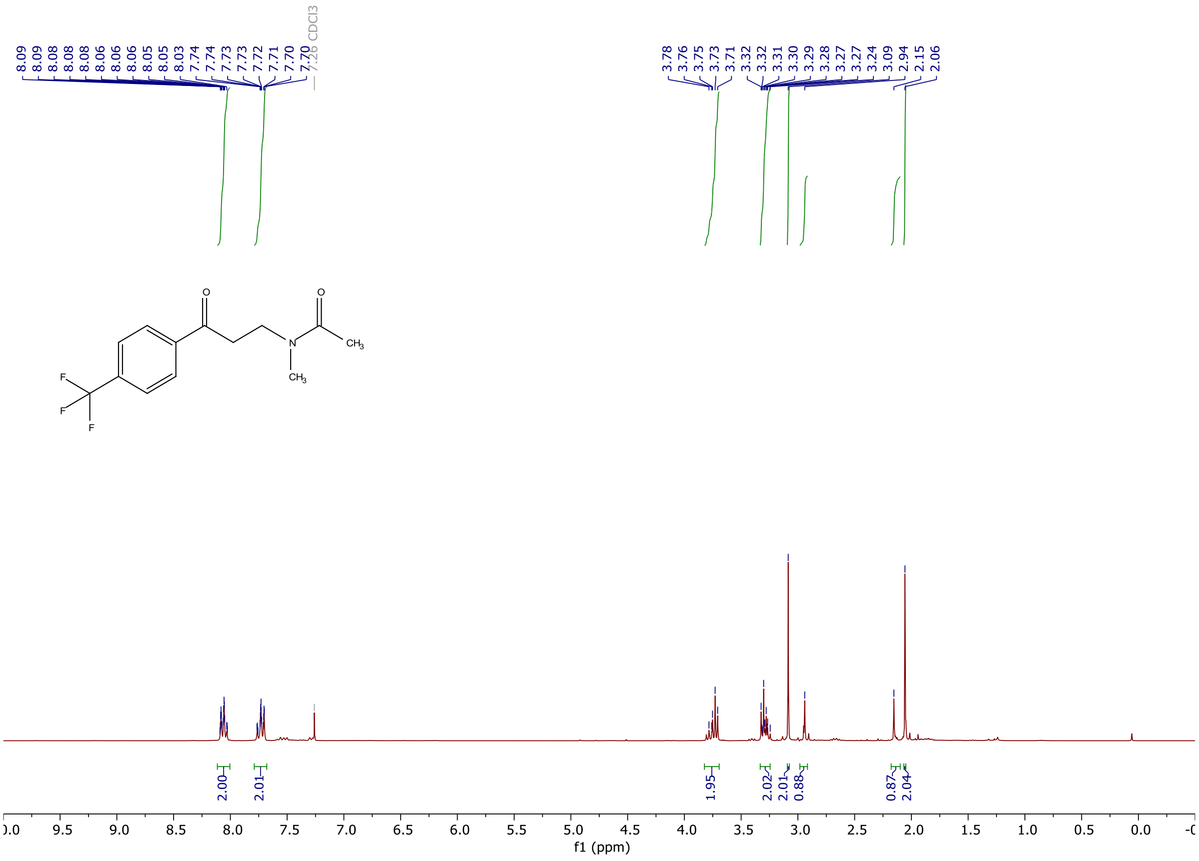

^{13}C NMR (75 MHz, $CDCl_3$) of **14**

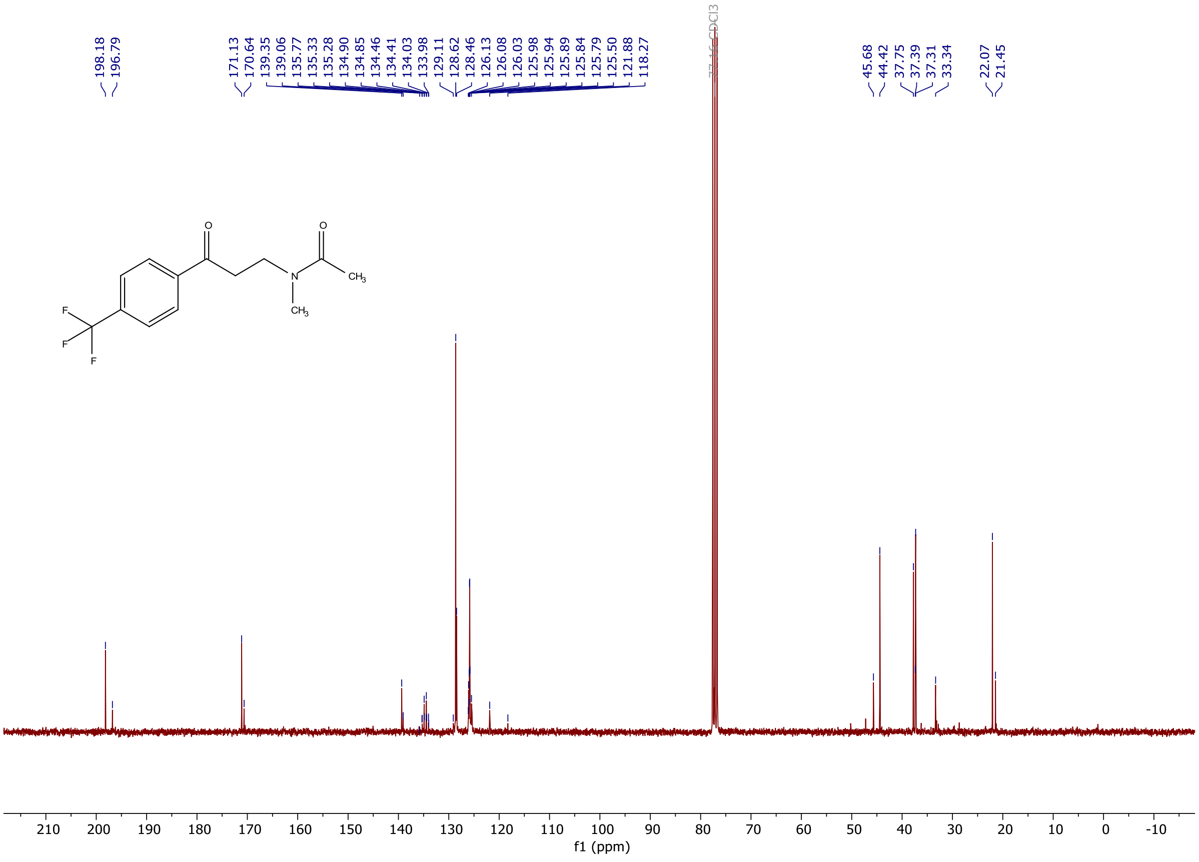

^{19}F NMR (282 MHz, $CDCl_3$) of **14**

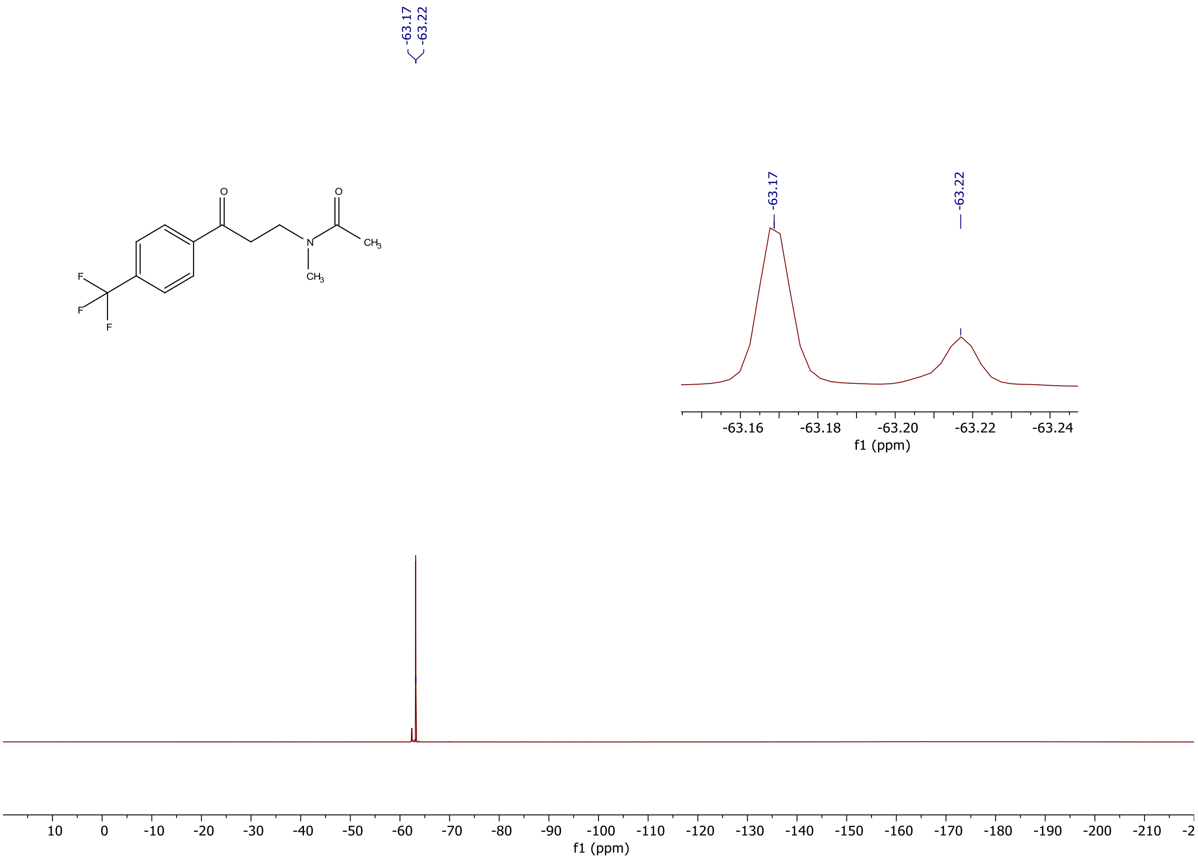

^{1}H NMR (300 MHz, $CDCl_3$) of **15**

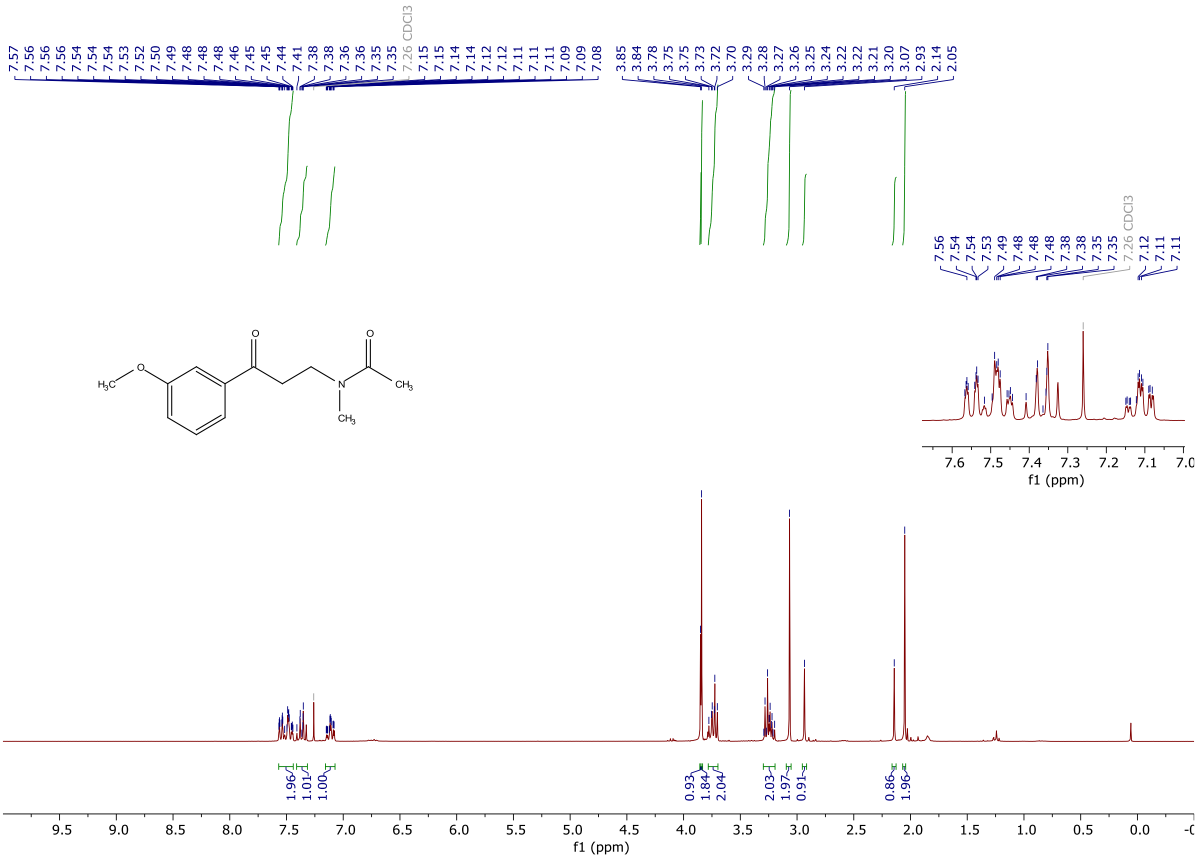

^{13}C NMR (75 MHz, $CDCl_3$) of **15**

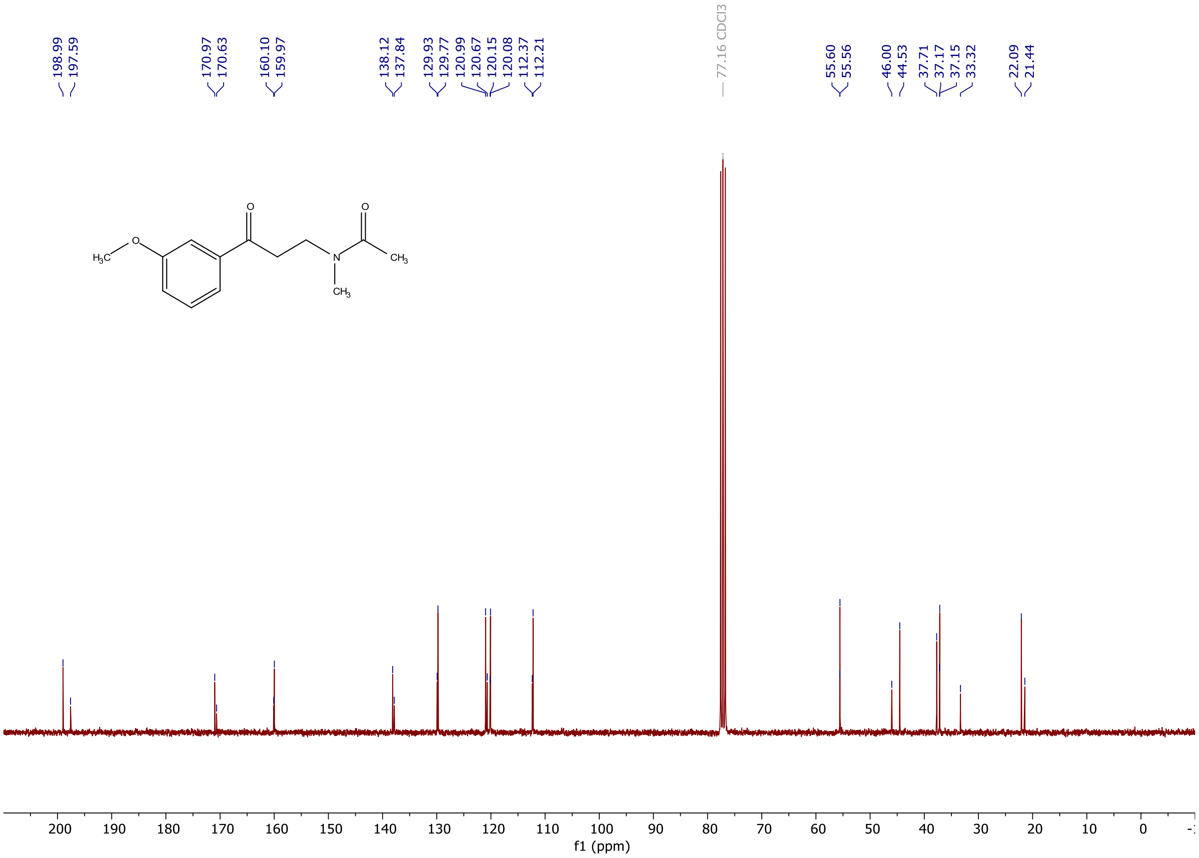

^{1}H NMR (300 MHz, $CDCl_3$) of **16**

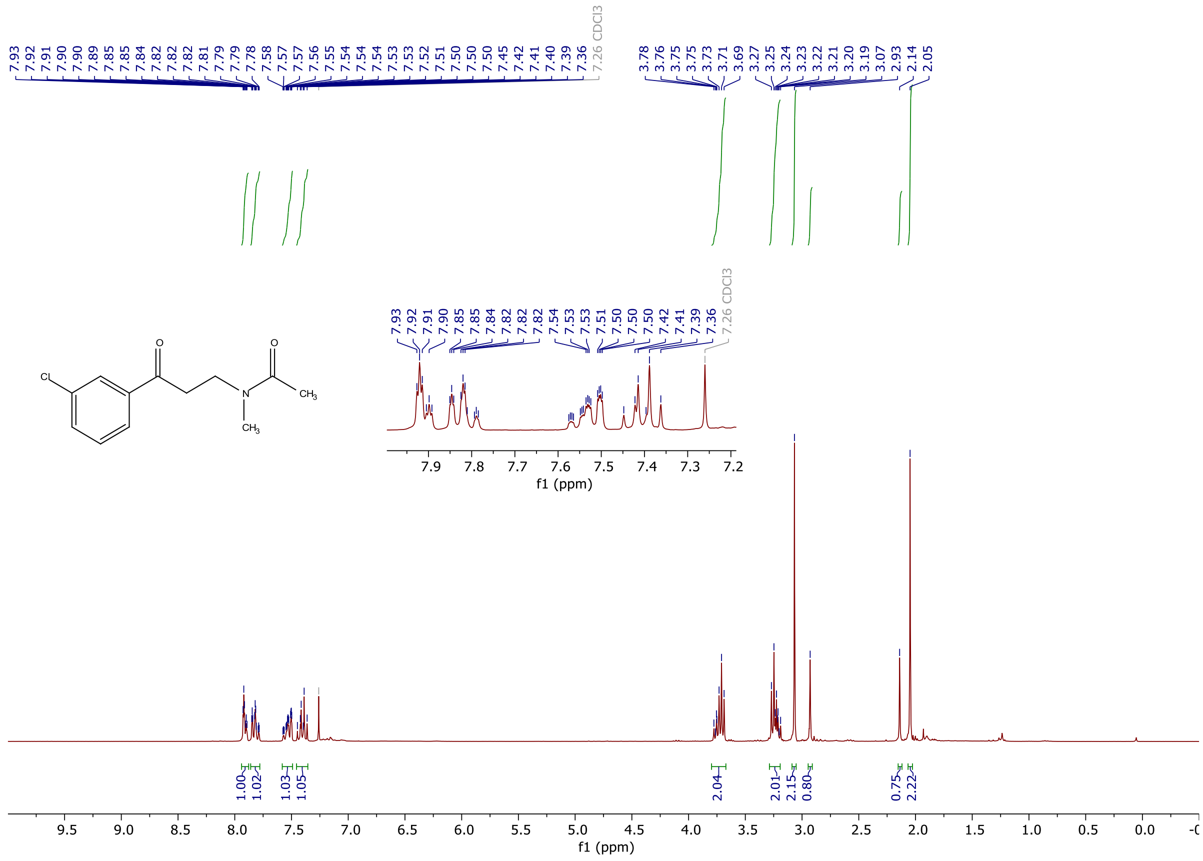

^{13}C NMR (75 MHz, $CDCl_3$) of **16**

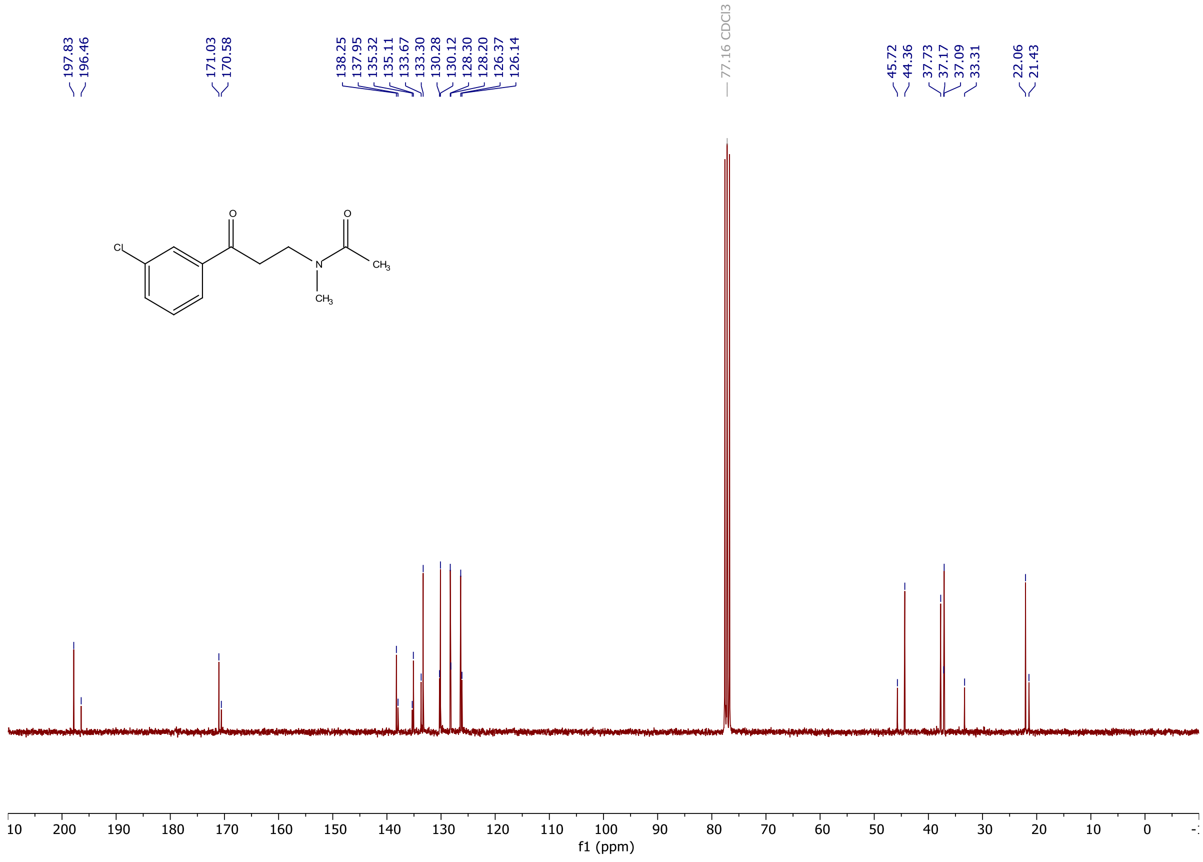

^{1}H NMR (300 MHz, $CDCl_3$) of **17**

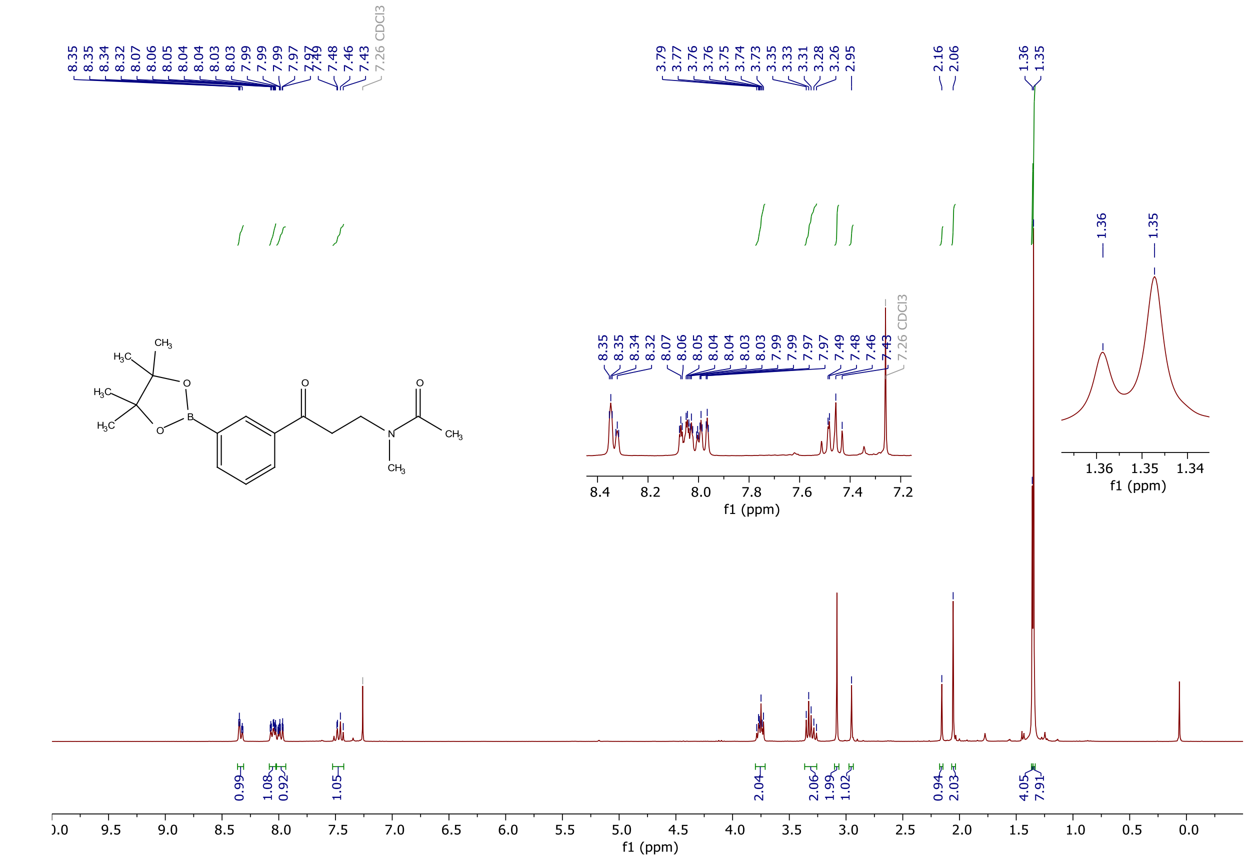

^{13}C NMR (75 MHz, $CDCl_3$) of **17**

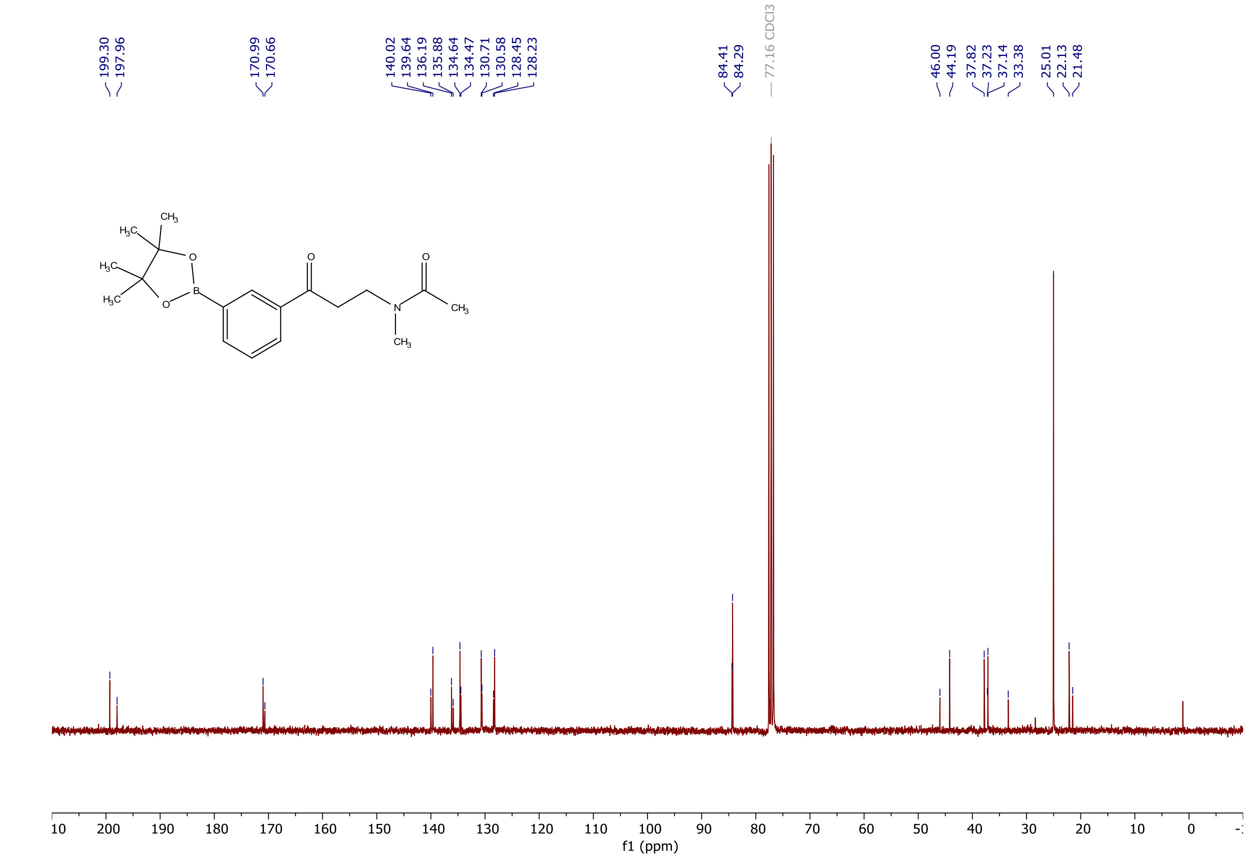

^{11}B NMR (96 MHz, $CDCl_3$) of **17**

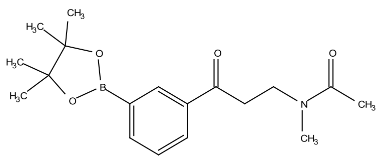

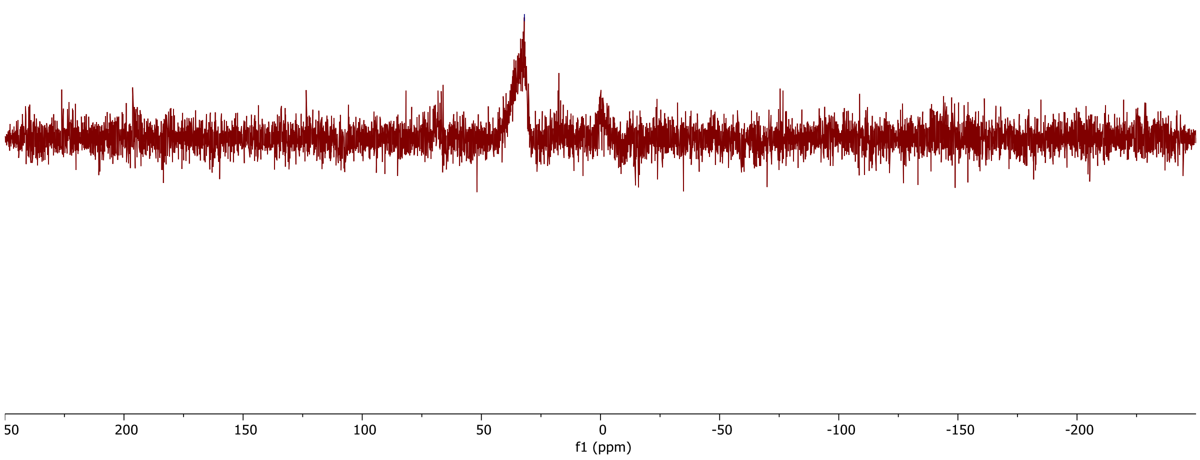

^{1}H NMR (300 MHz, $CDCl_3$) of **18**

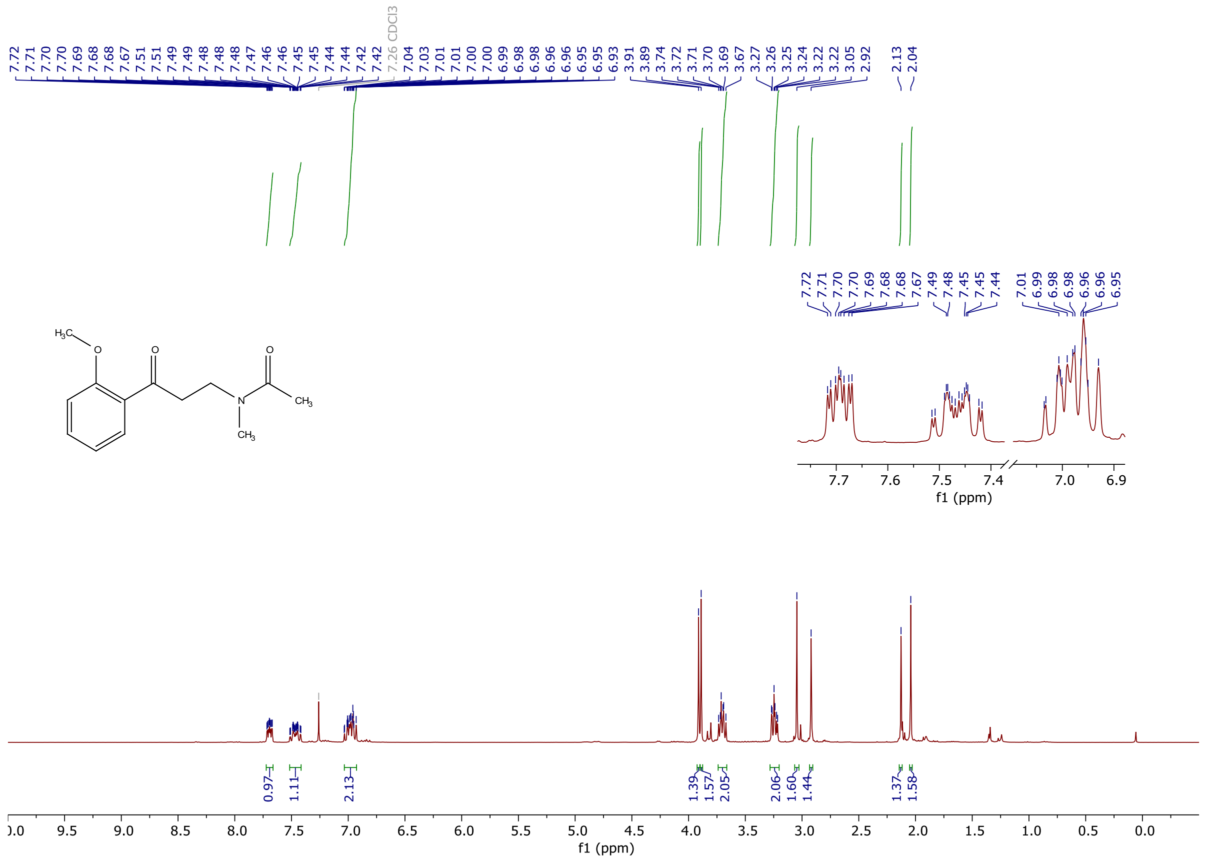

^{13}C NMR (75 MHz, $CDCl_3$) of **18**

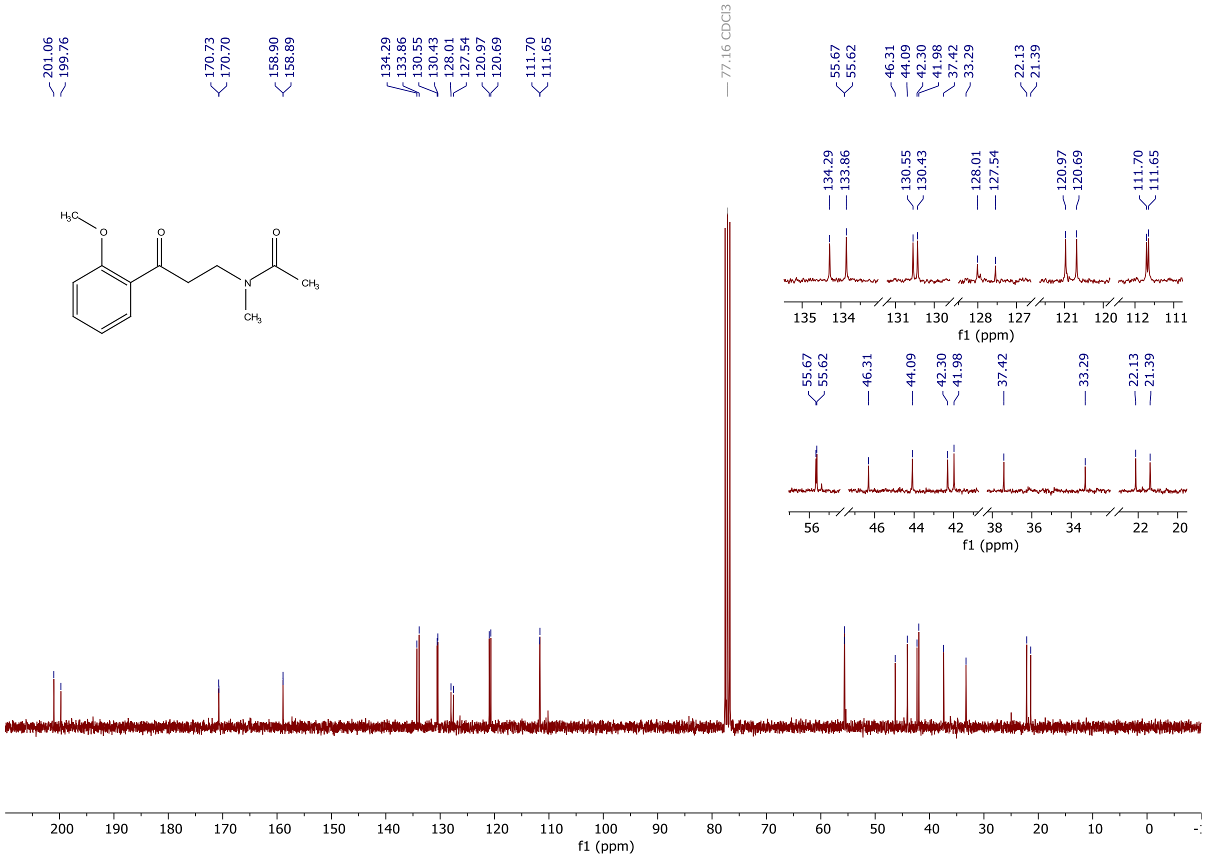

^{1}H NMR (300 MHz, $CDCl_3$) of **19**

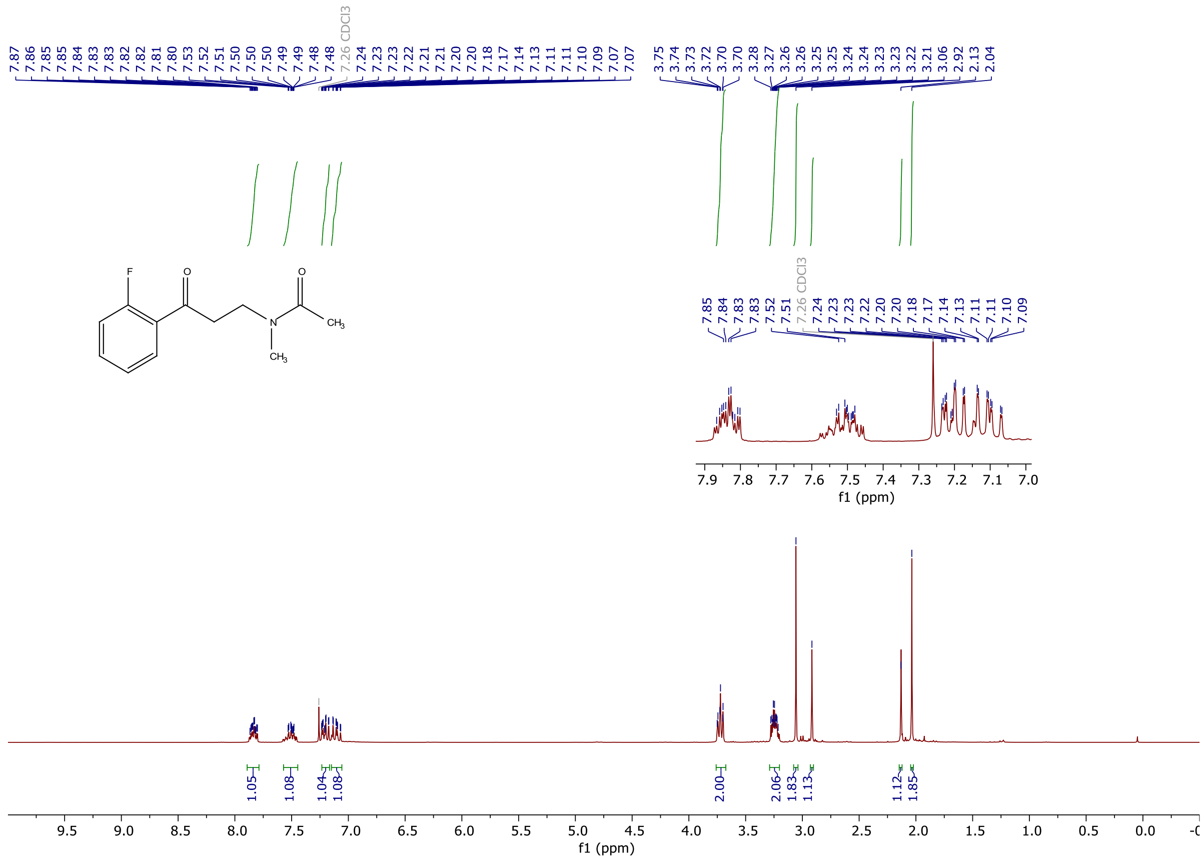

^{13}C NMR (75 MHz, $CDCl_3$) of **19**

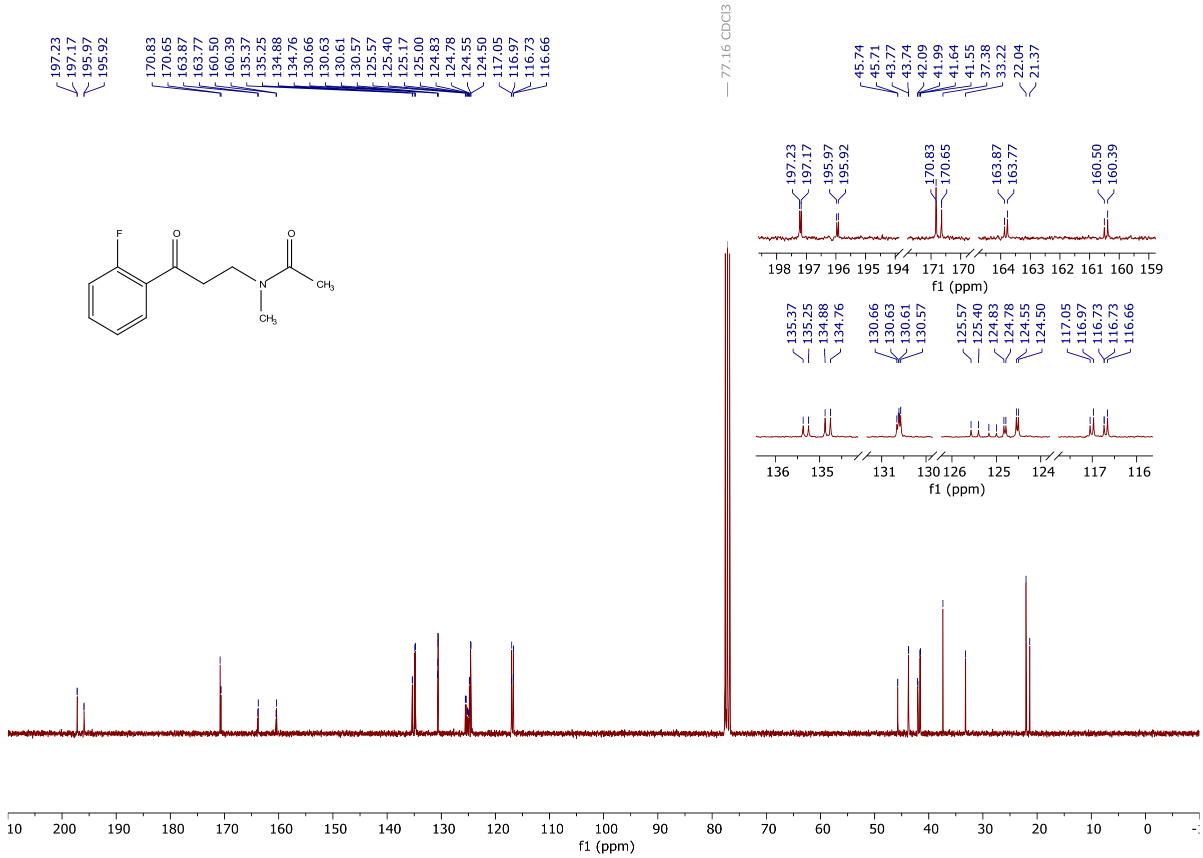

^{19}F NMR (282 MHz, $CDCl_3$) of **19**

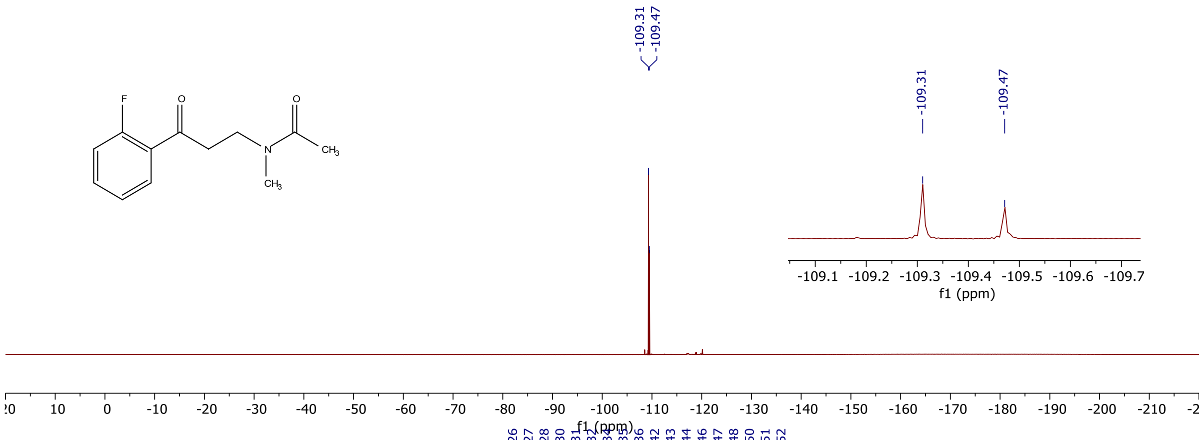

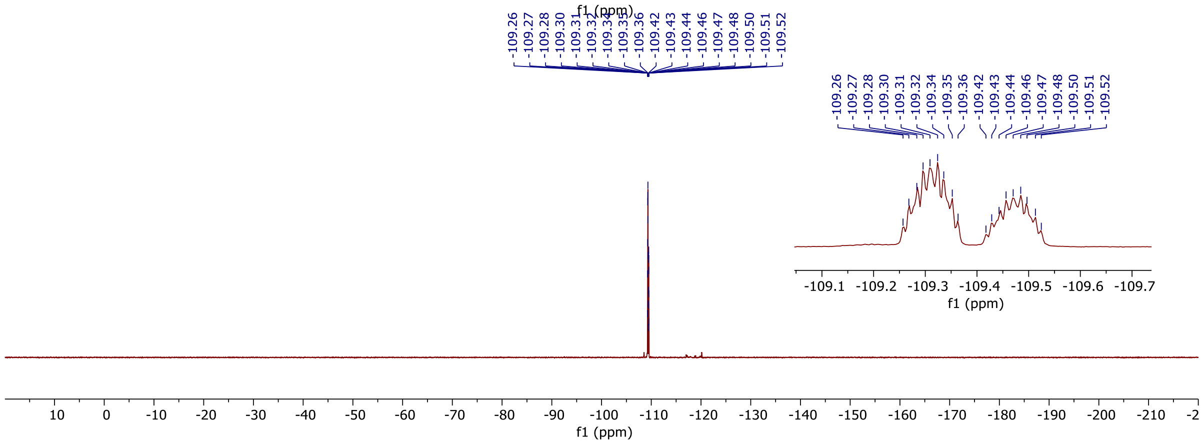

^{1}H NMR (300 MHz, $CDCl_3$) of **20**

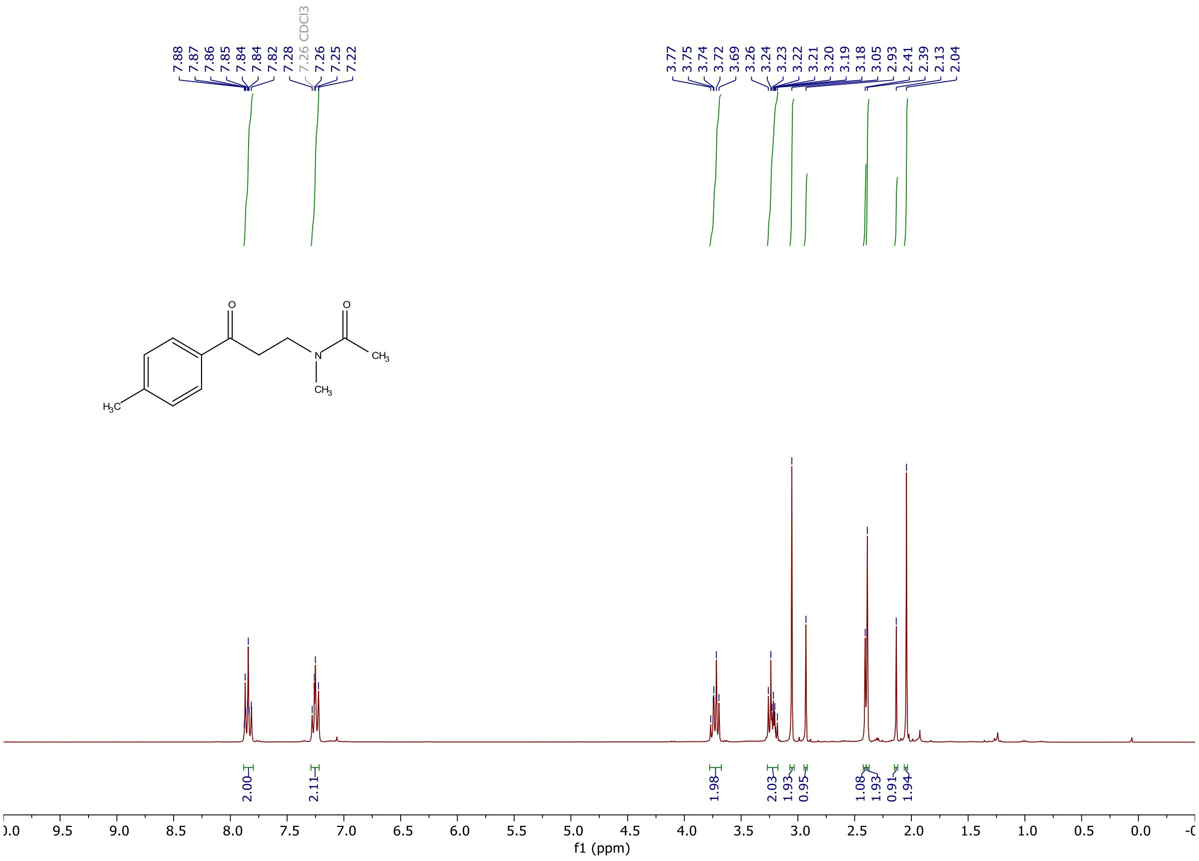

^{13}C NMR (75 MHz, $CDCl_3$) of **20**

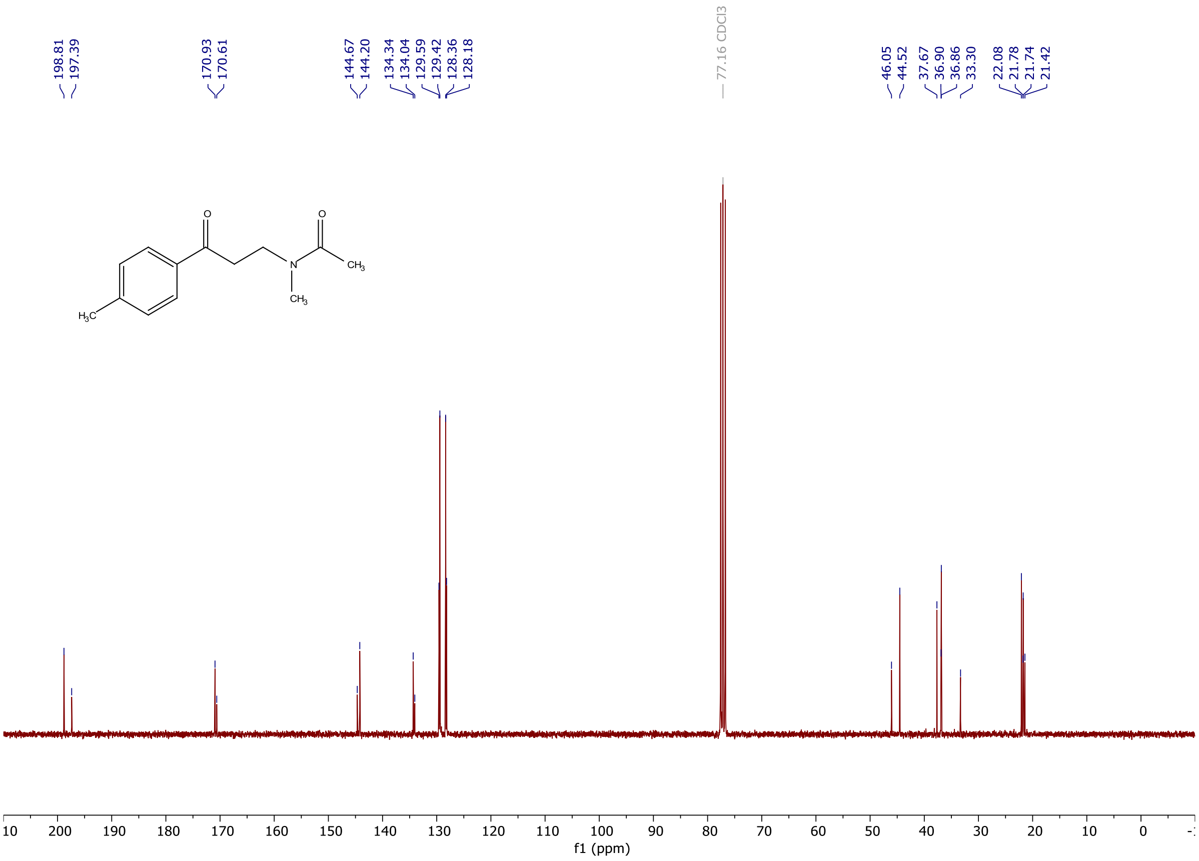

^{1}H NMR (300 MHz, $CDCl_3$) of **21**

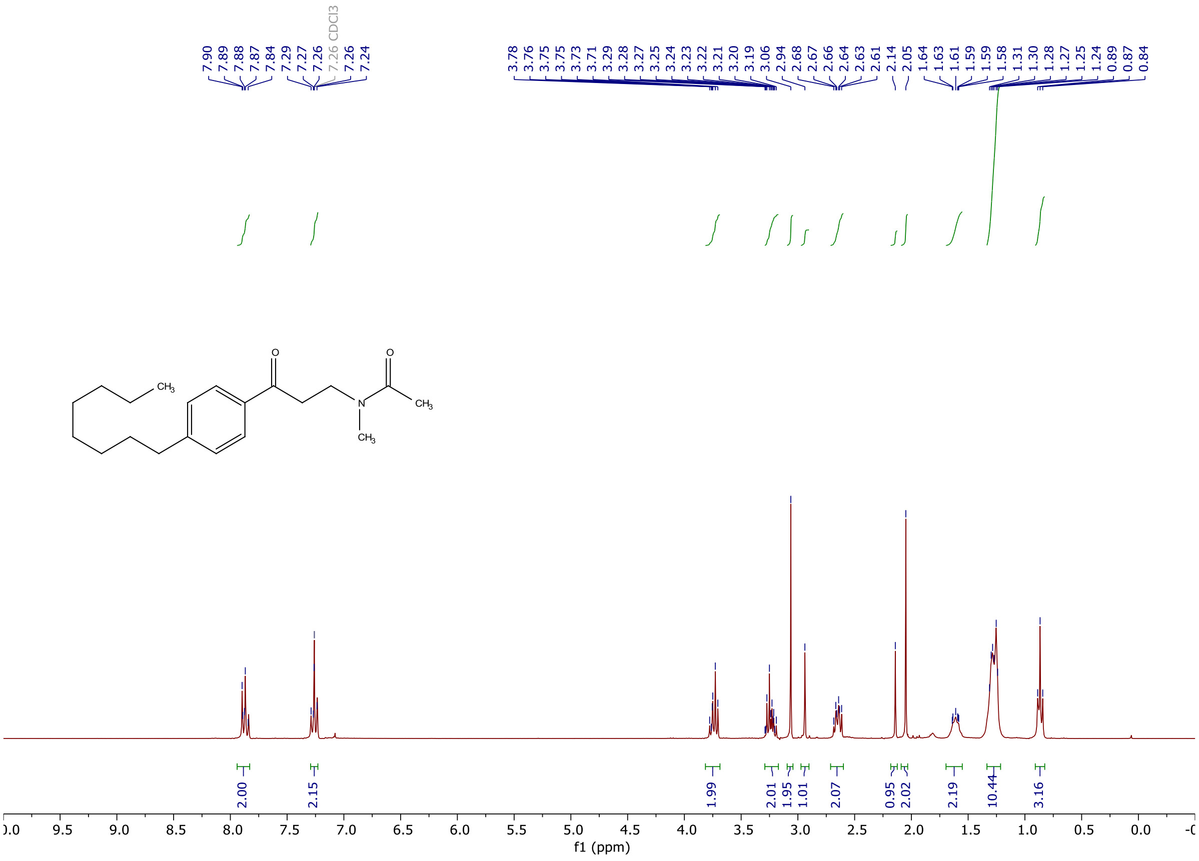

^{13}C NMR (75 MHz, $CDCl_3$) of **21**

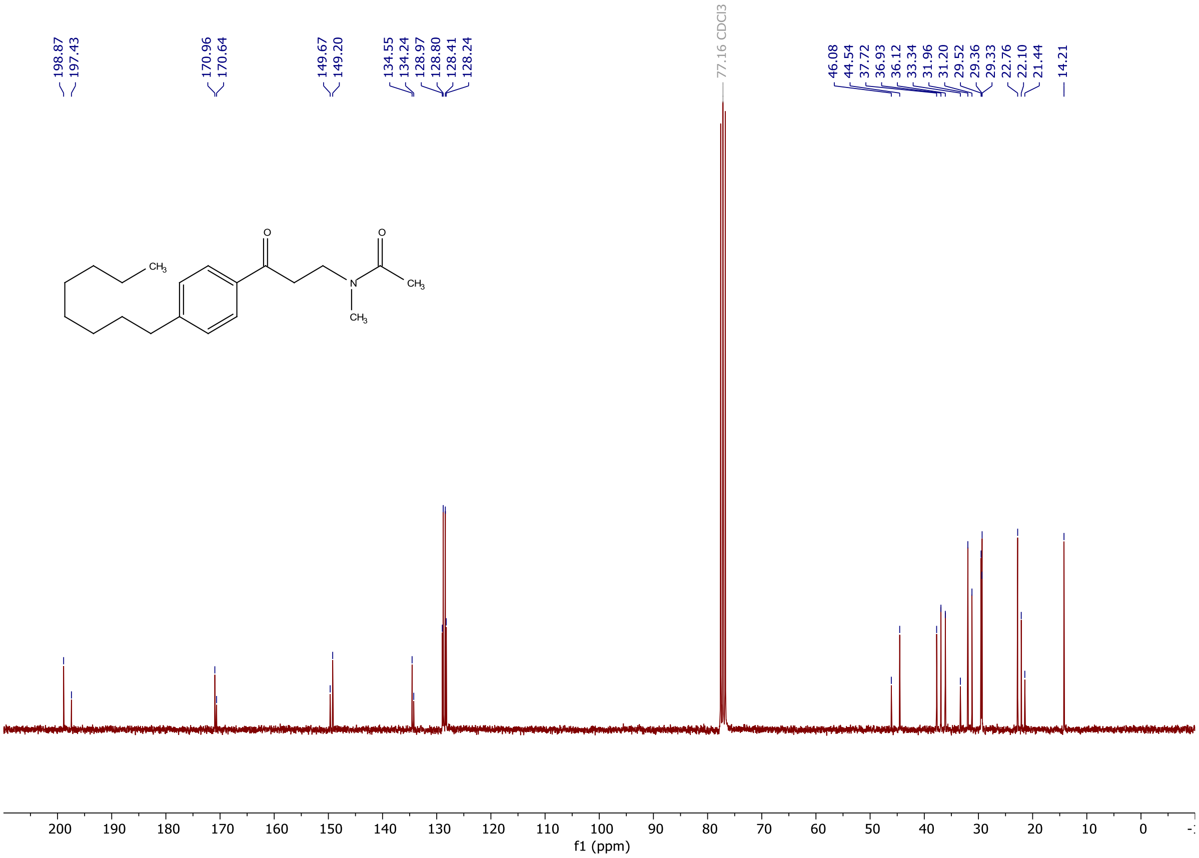

^{1}H NMR (300 MHz, $CDCl_3$) of **22**

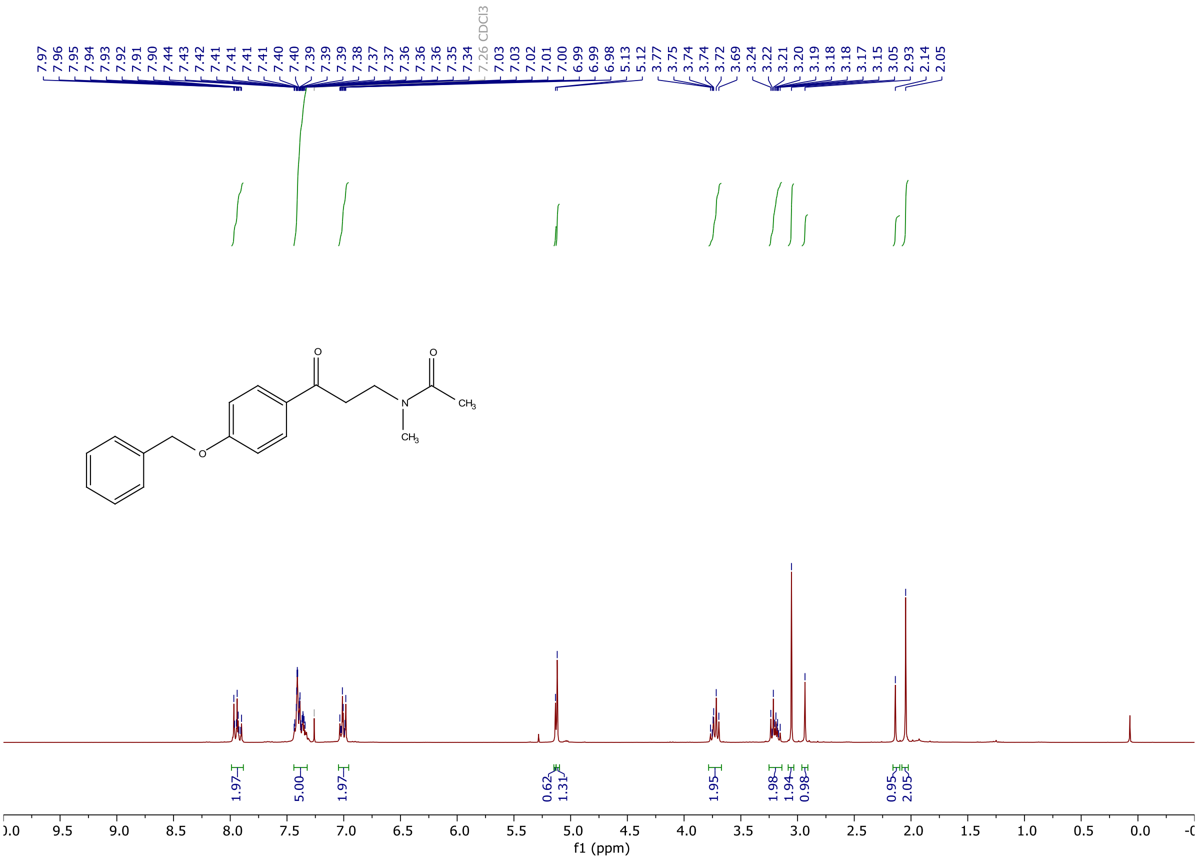

^{13}C NMR (75 MHz, $CDCl_3$) of **22**

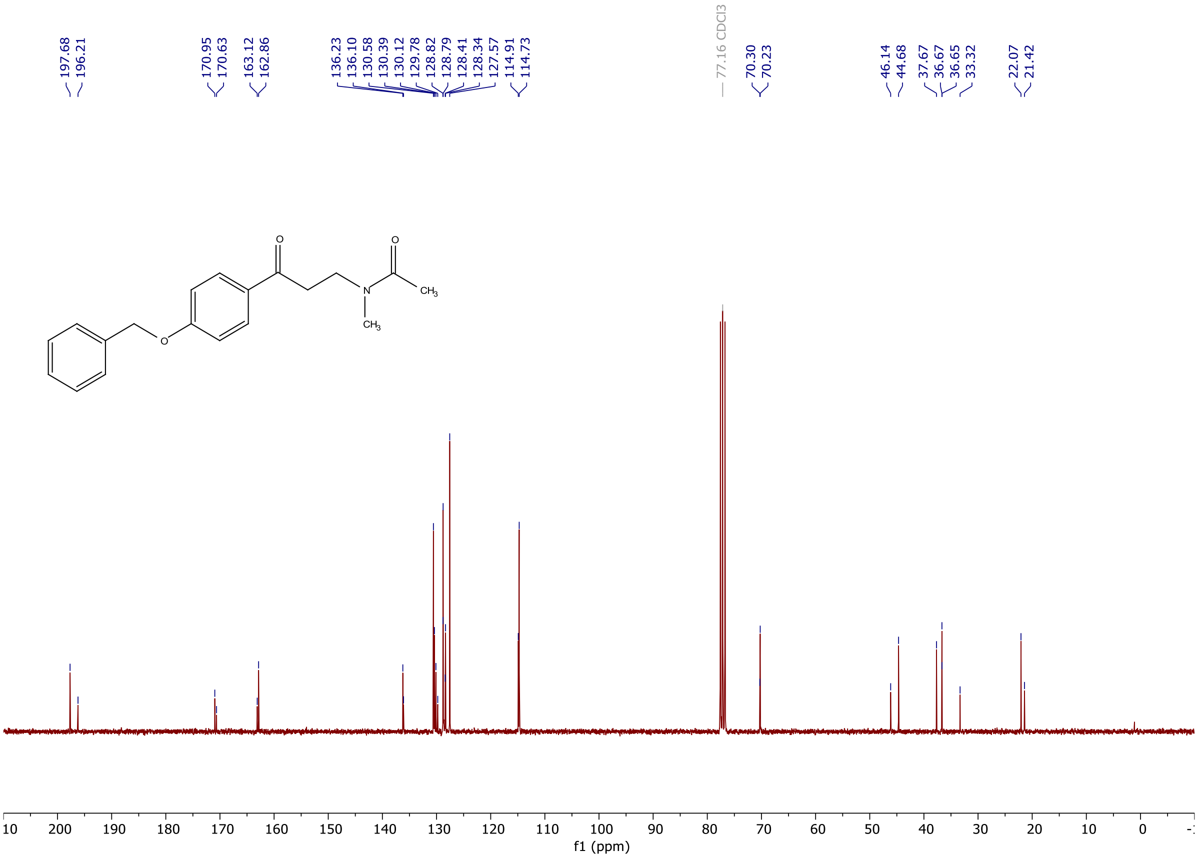

^{1}H NMR (300 MHz, $CDCl_3$) of **23**

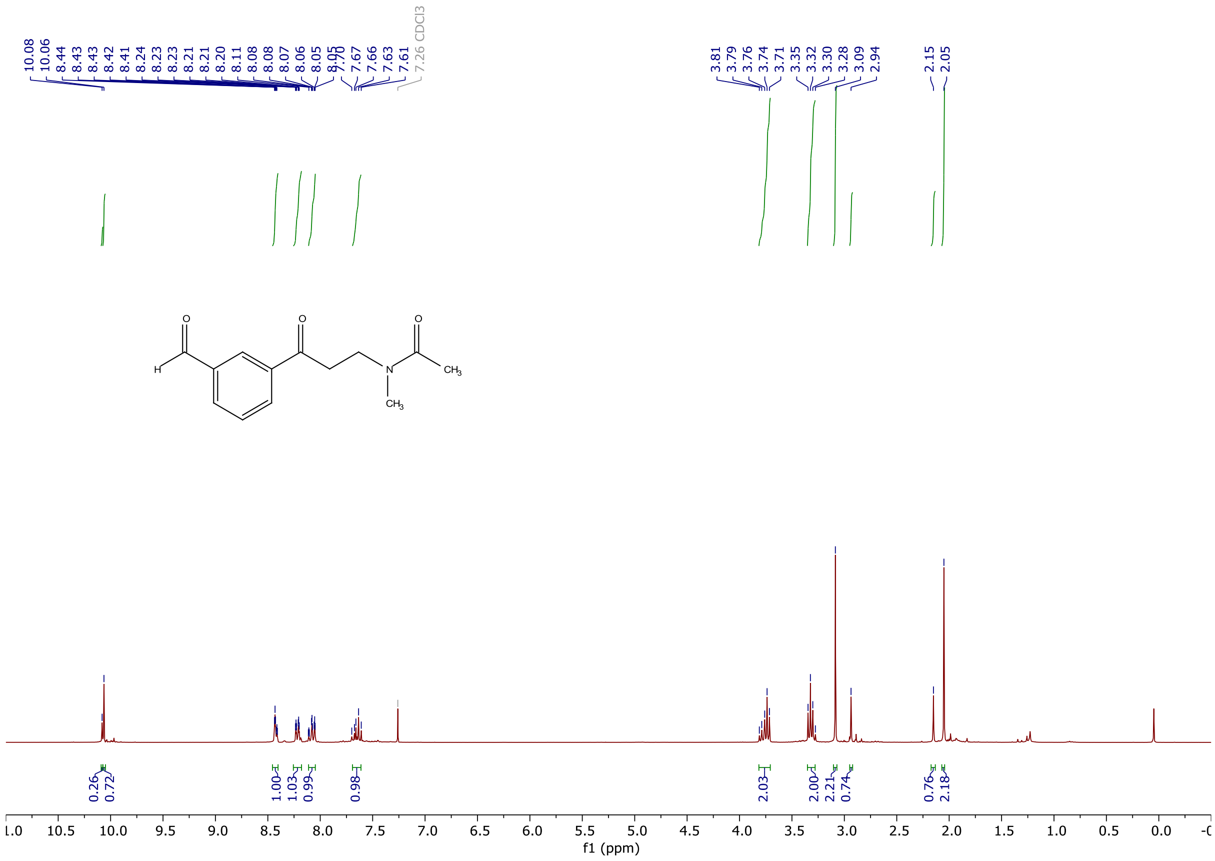

^{13}C NMR (75 MHz, $CDCl_3$) of **23**

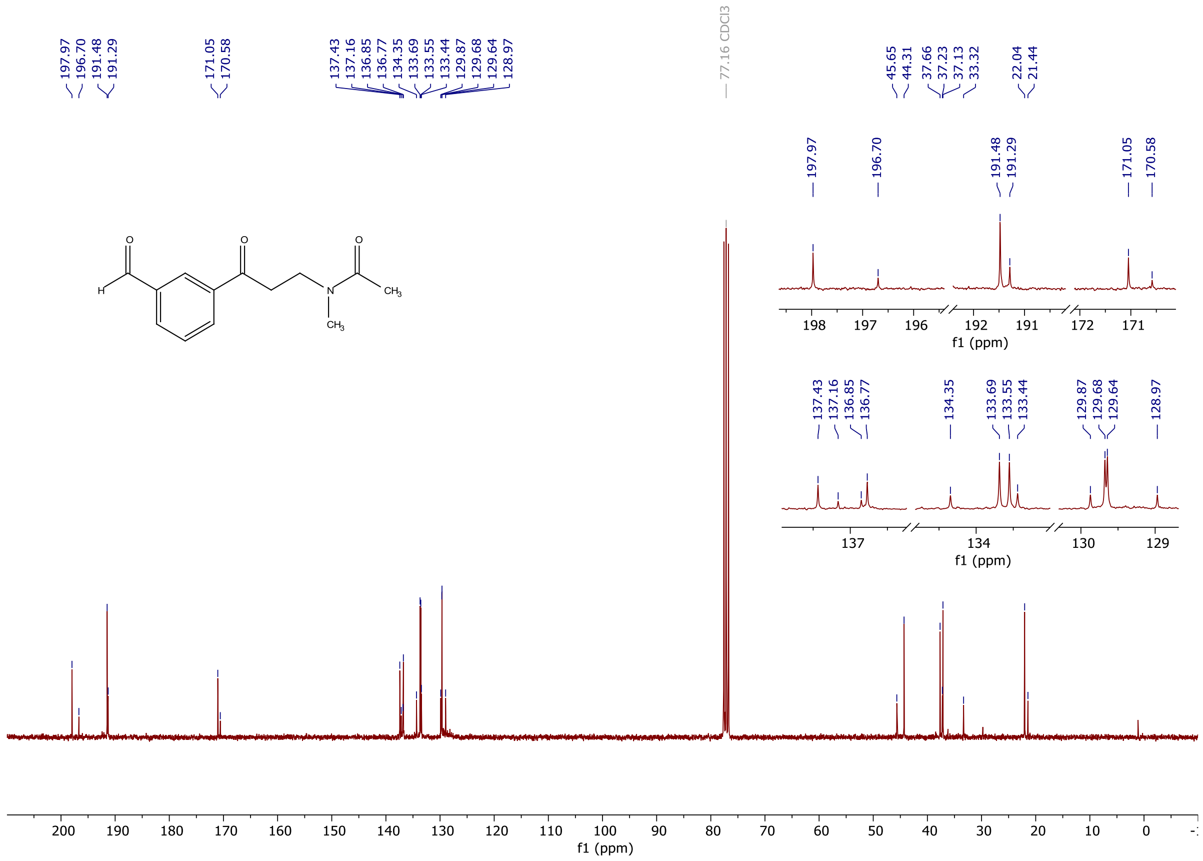

^{1}H NMR (300 MHz, $CDCl_3$) of **24**

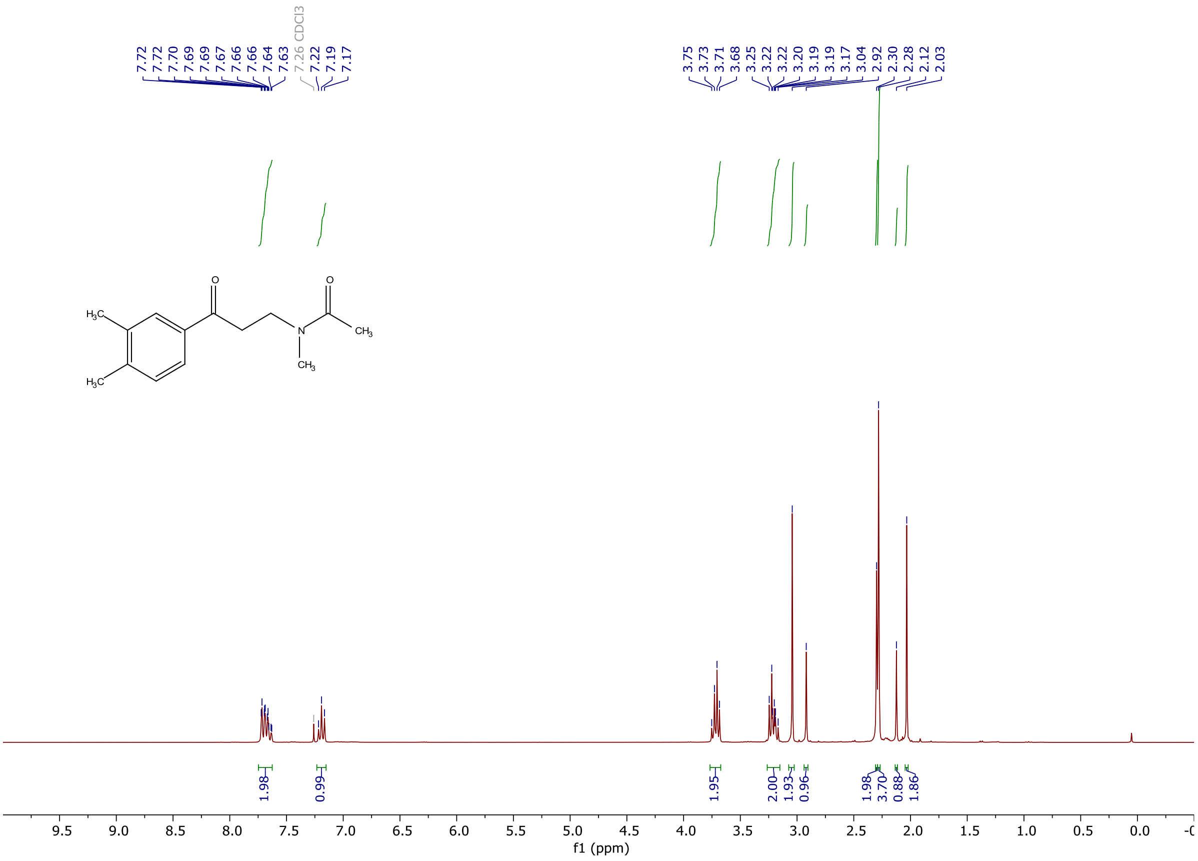

^{13}C NMR (75 MHz, $CDCl_3$) of **24**

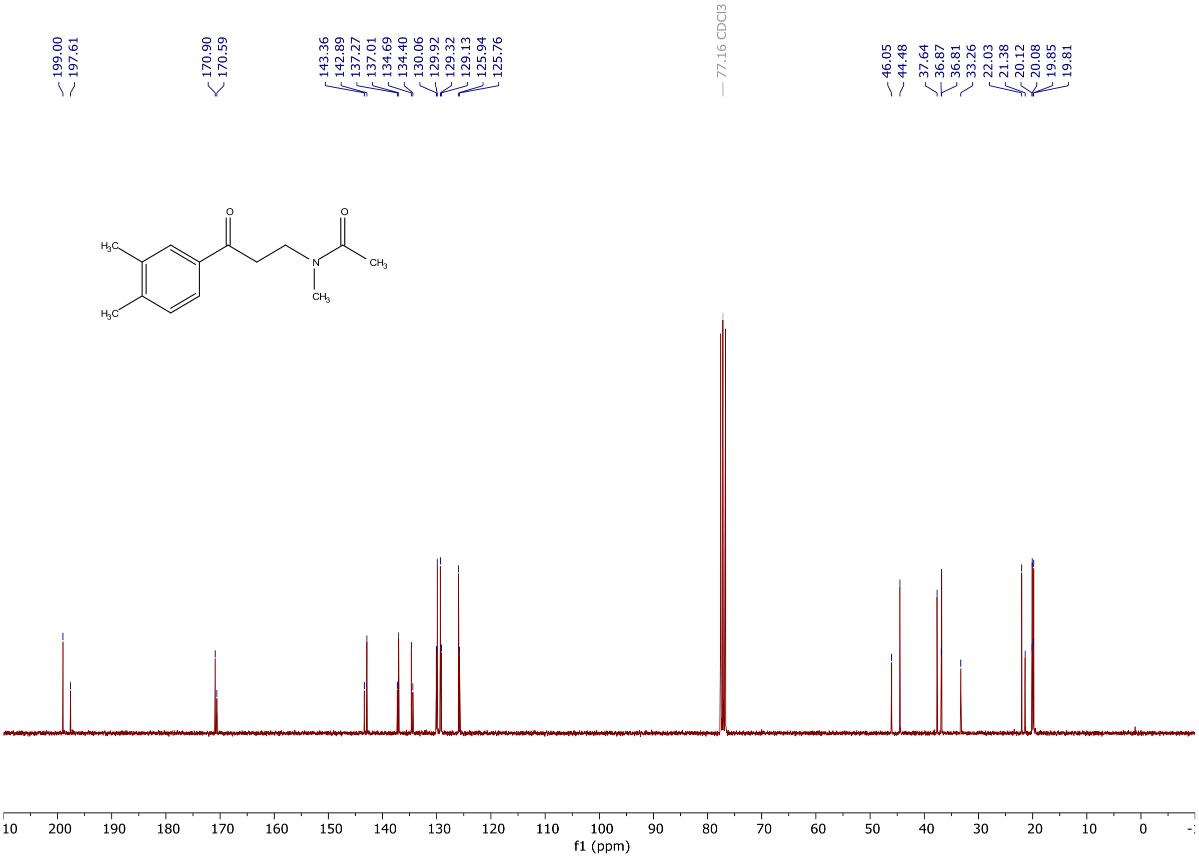

^{1}H NMR (300 MHz, $CDCl_3$) of **25**

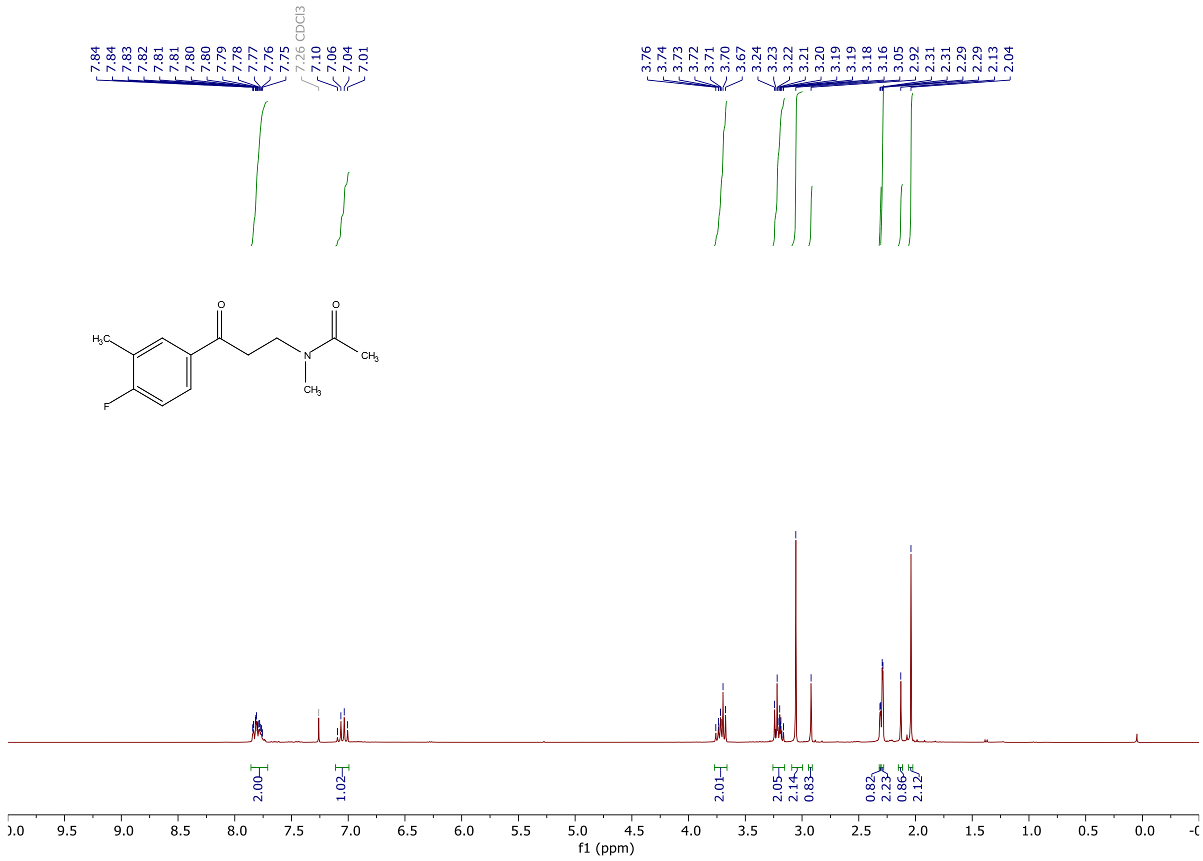

^{13}C NMR (75 MHz, $CDCl_3$) of **25**

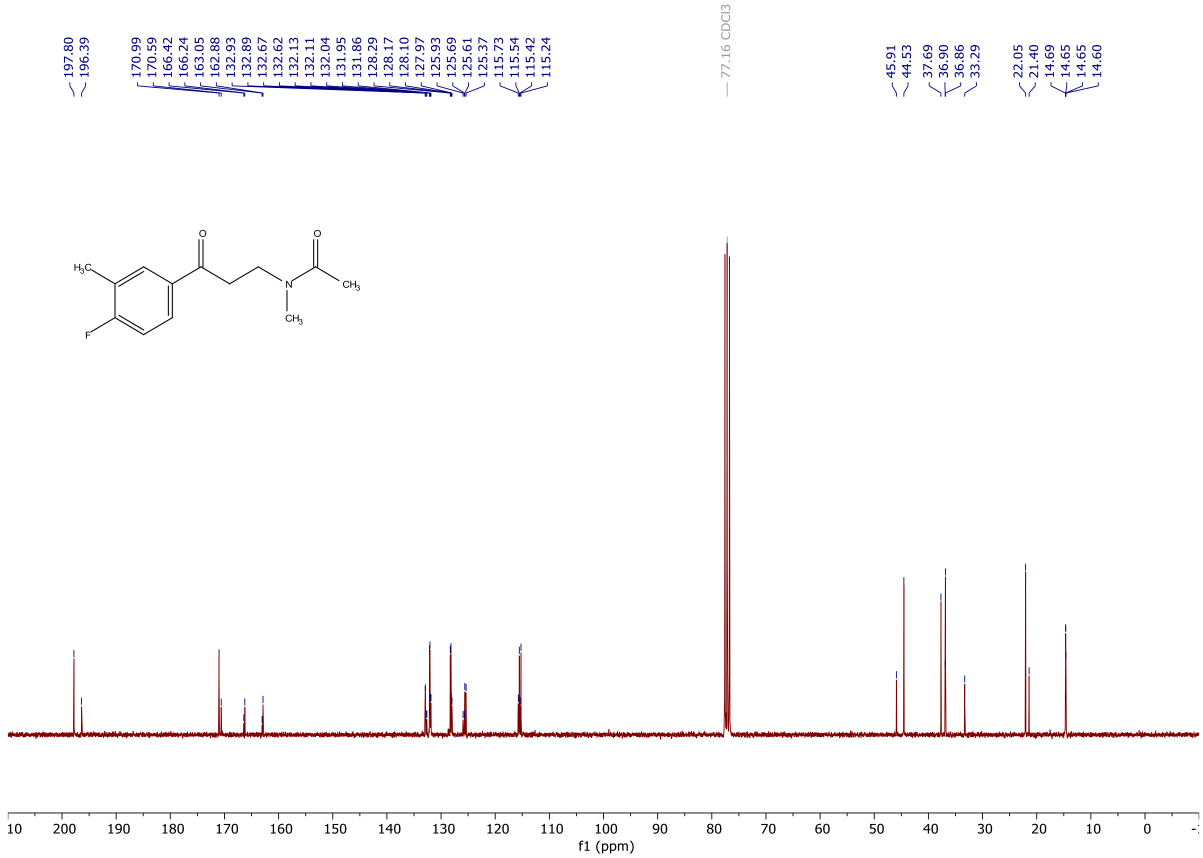

^{19}F NMR (282 MHz, $CDCl_3$) of **25**

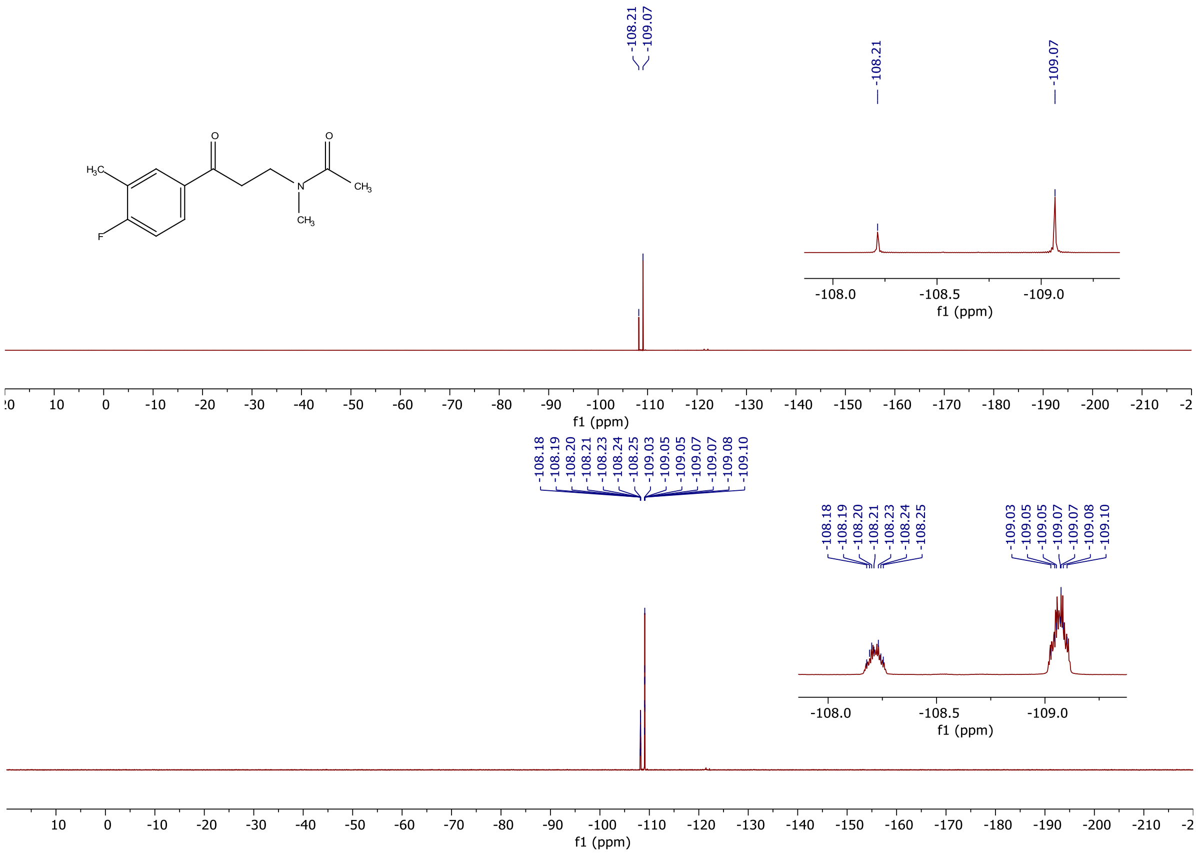

^1H NMR (300 MHz, $CDCl_3$) of **26**

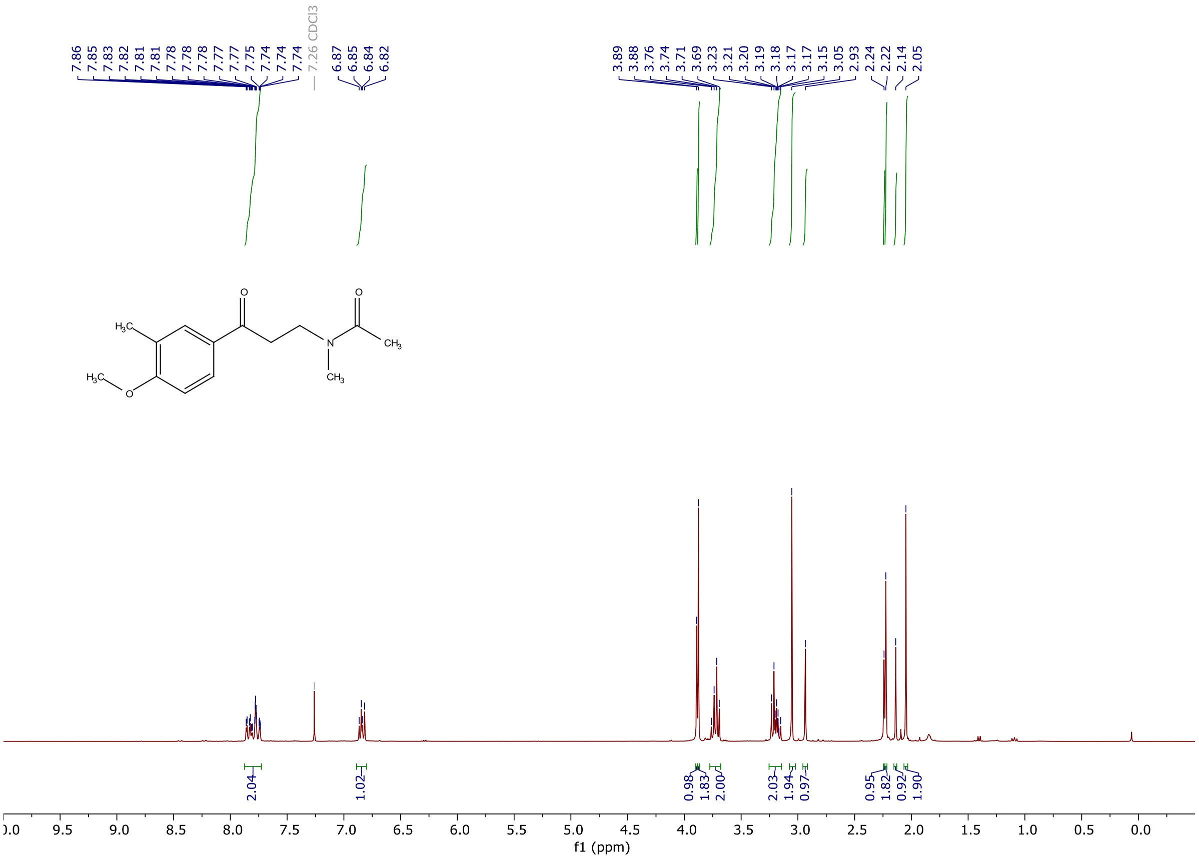

^{13}C NMR (75 MHz, $CDCl_3$) of **26**

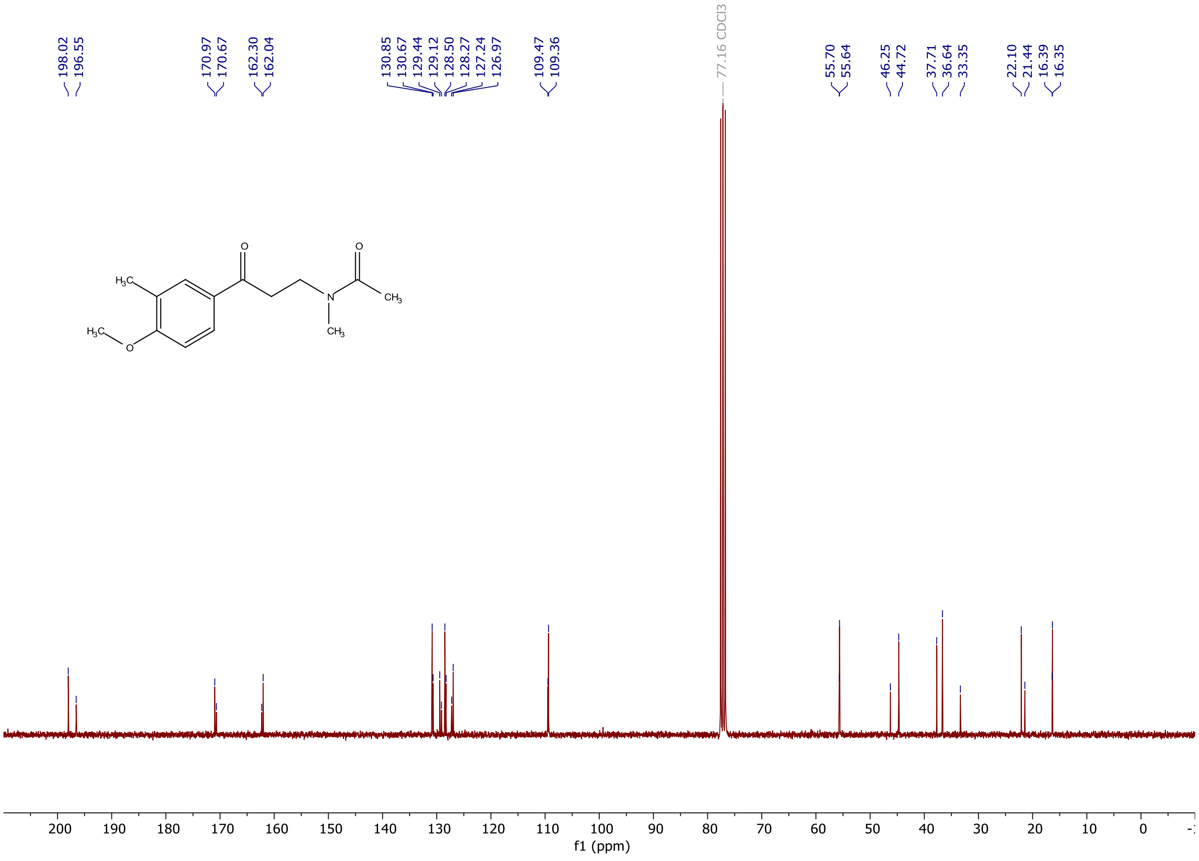

^{1}H NMR (300 MHz, $CDCl_3$) of **27**

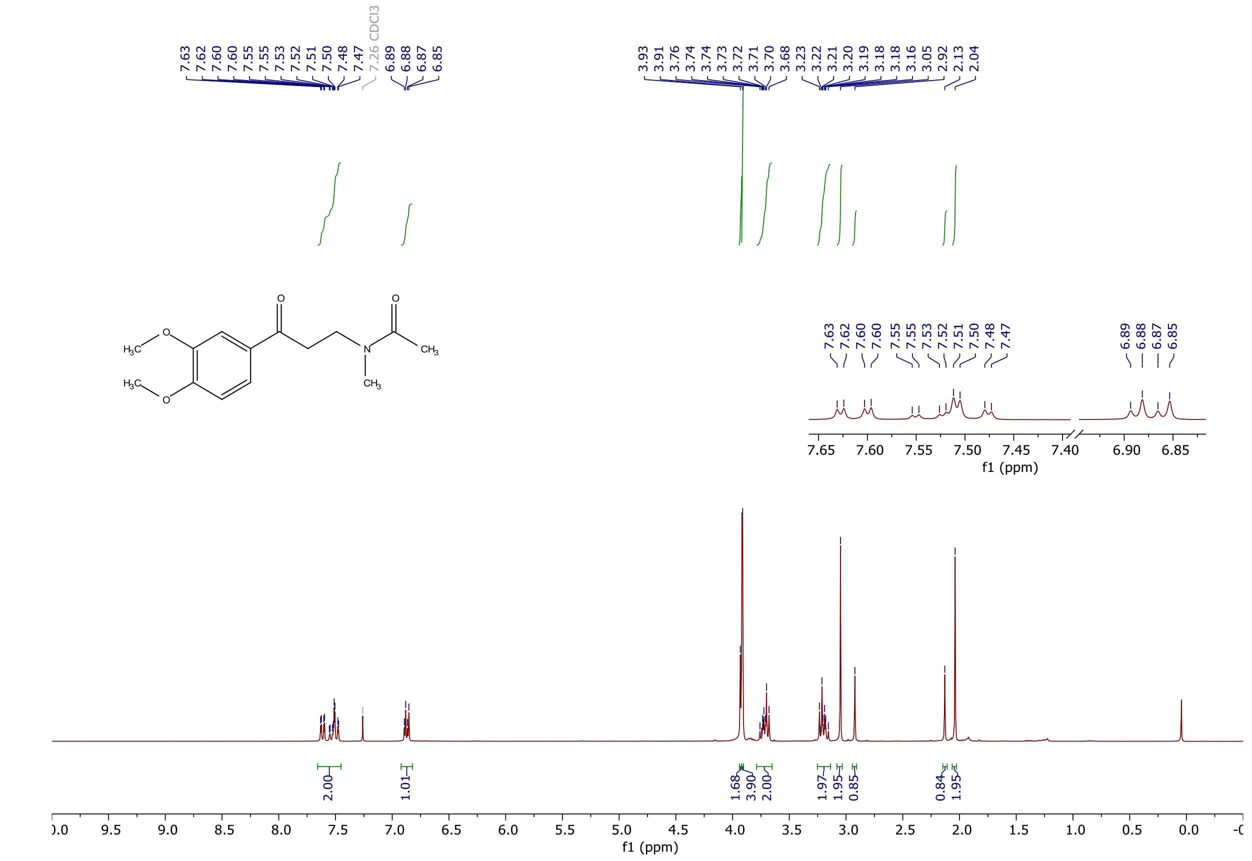

^{13}C NMR (75 MHz, $CDCl_3$) of **27**

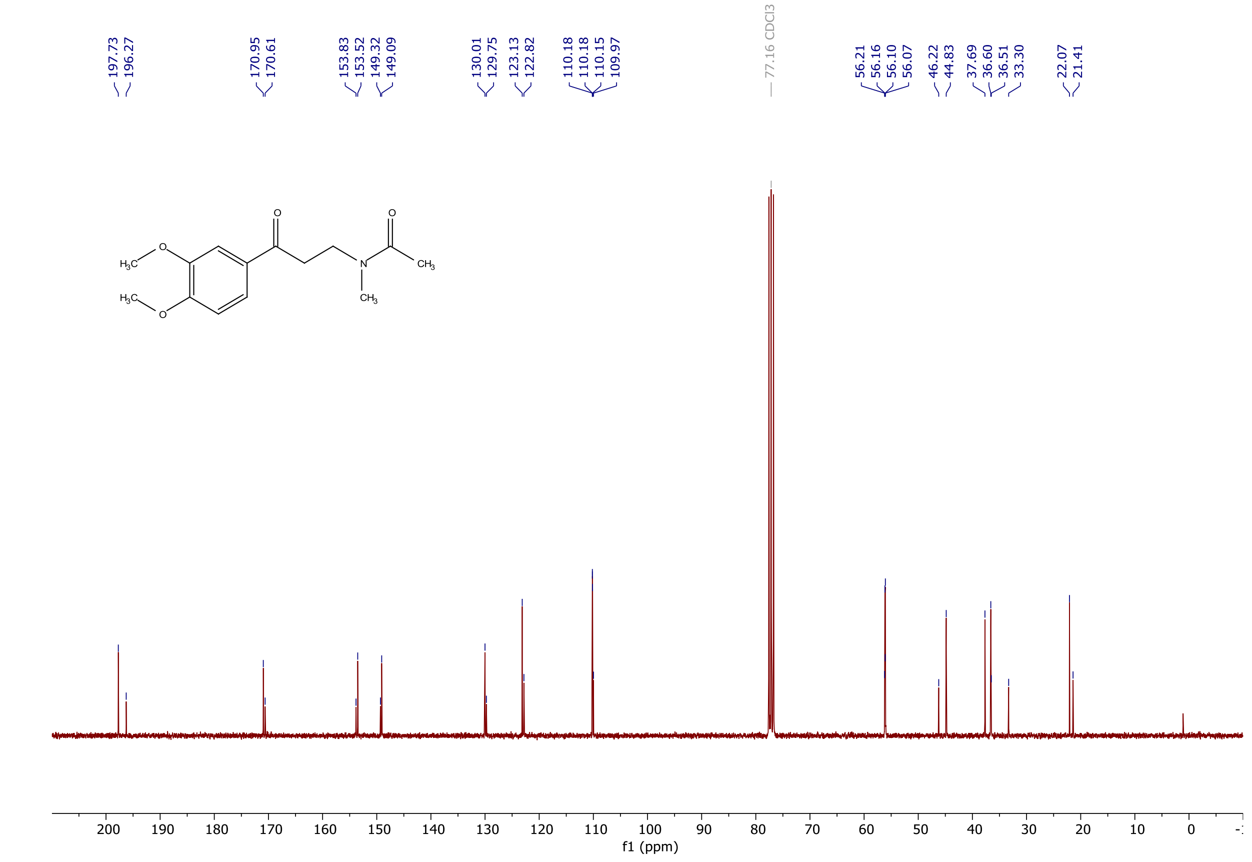

^{1}H NMR (300 MHz, $CDCl_3$) of **28**

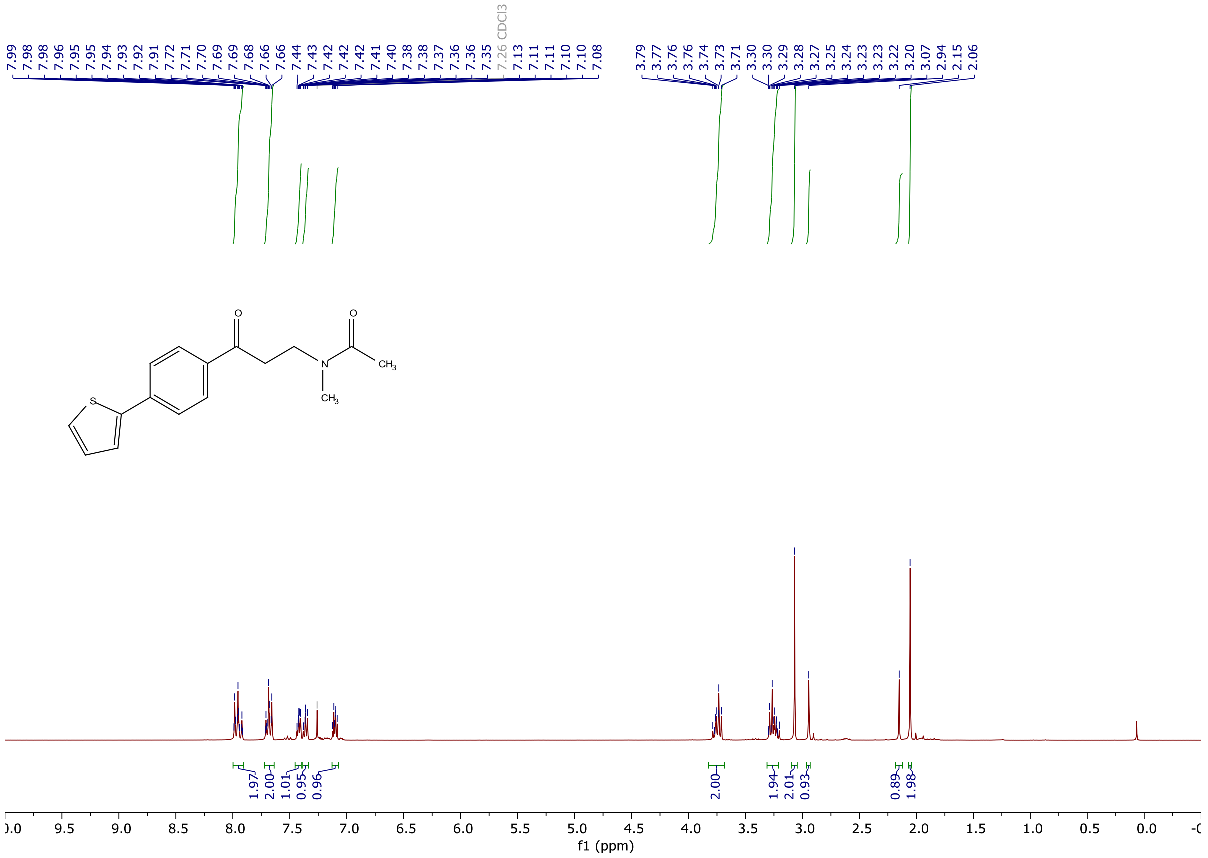

^{13}C NMR (75 MHz, $CDCl_3$) of **28**

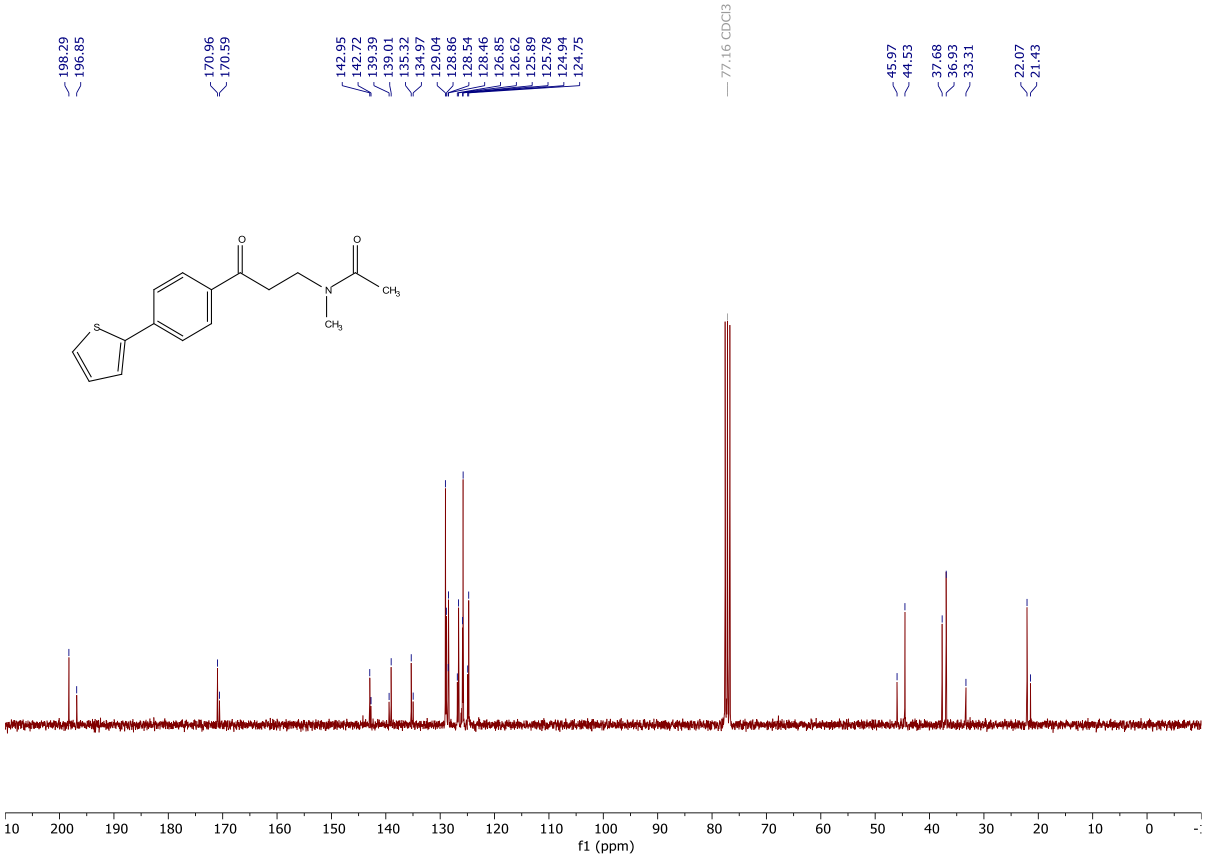

^{1}H NMR (300 MHz, $CDCl_3$) of **29**

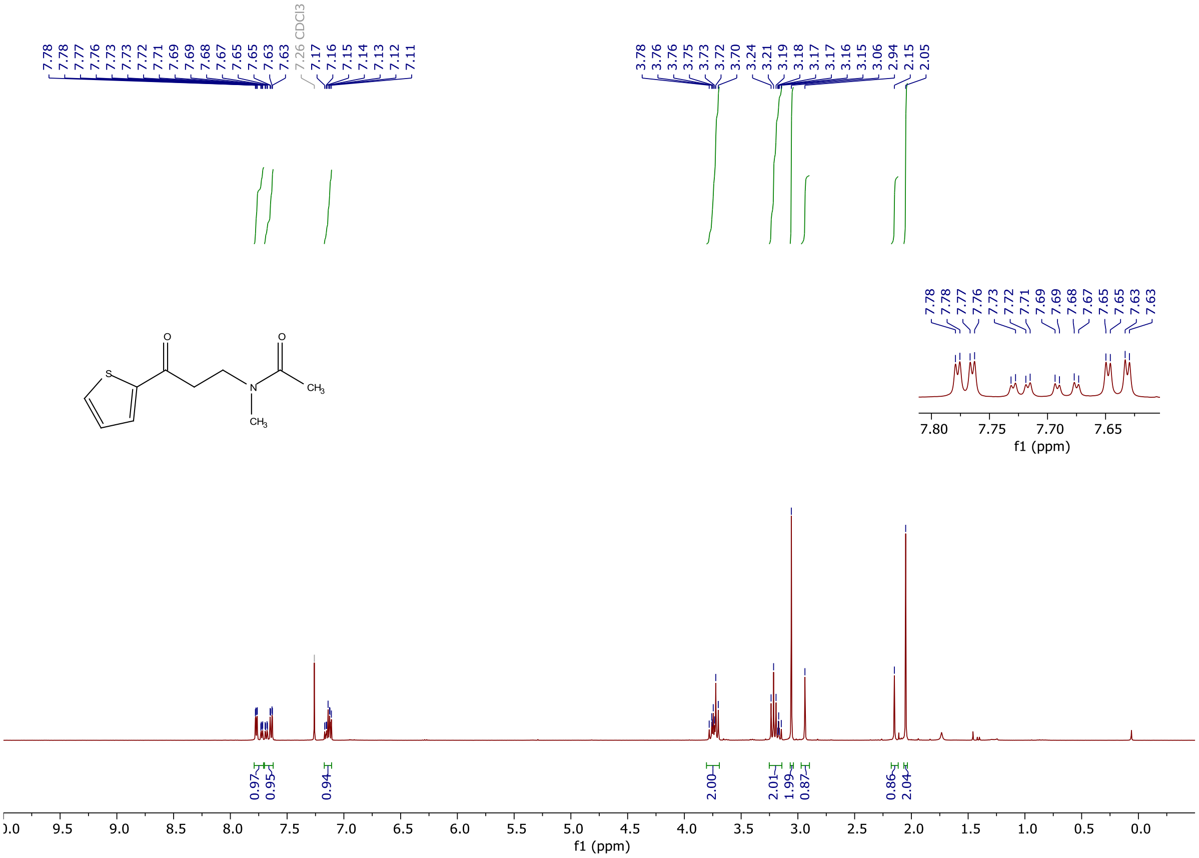

^{13}C NMR (75 MHz, $CDCl_3$) of **29**

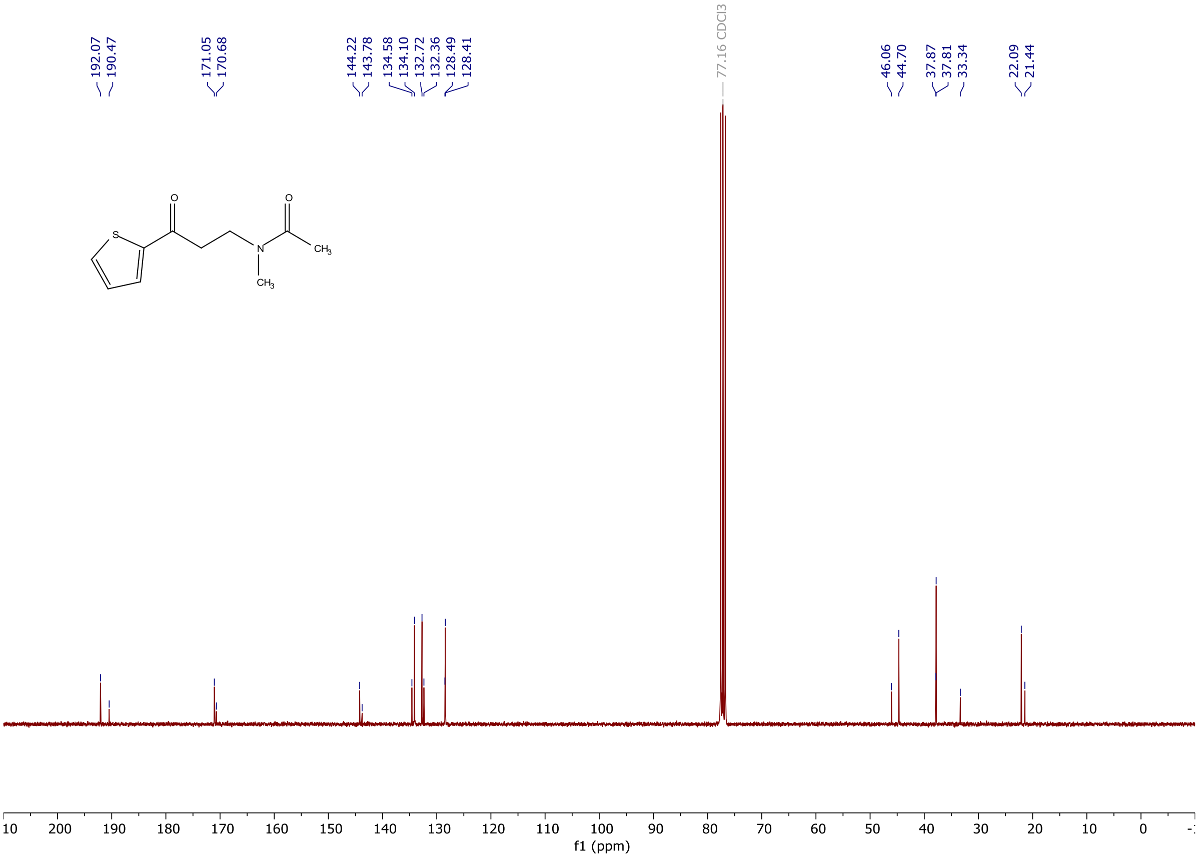

^1H NMR (300 MHz, $CDCl_3$) of **30**

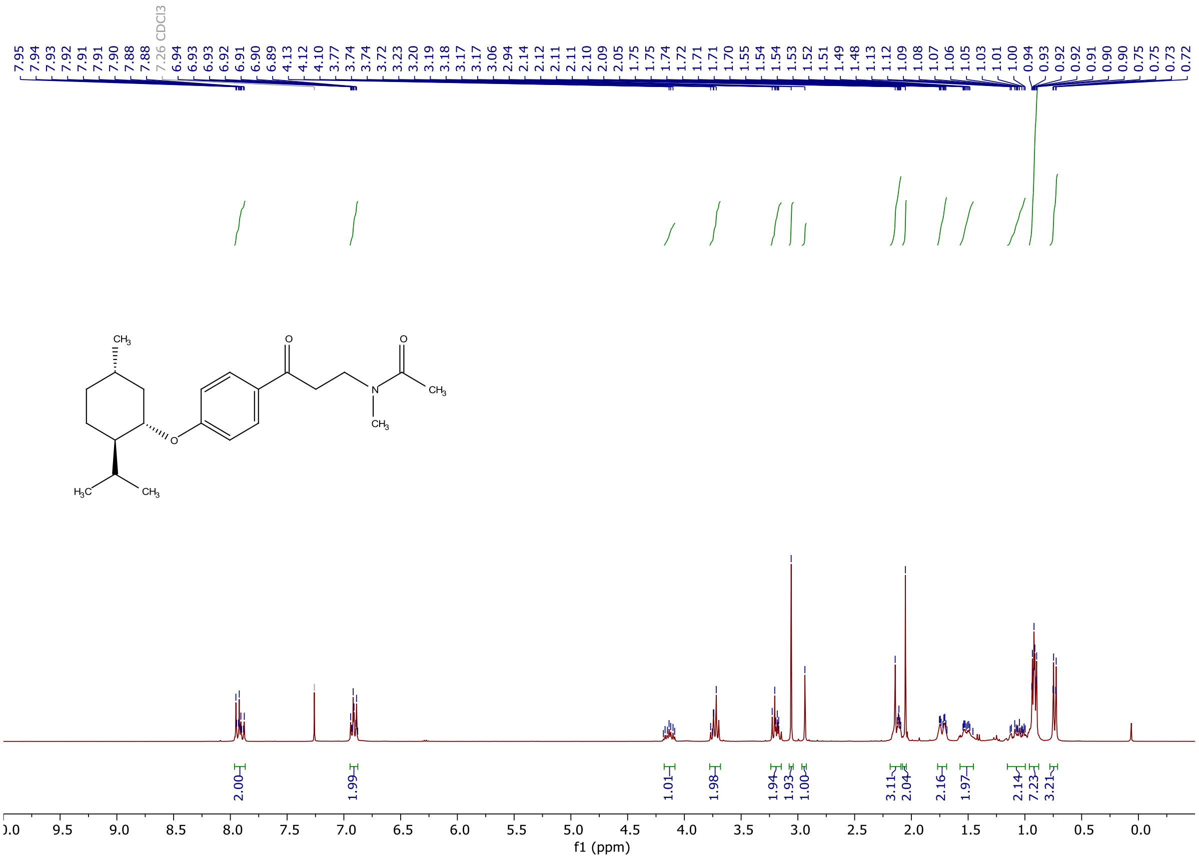

^{13}C NMR (75 MHz, $CDCl_3$) of **30**

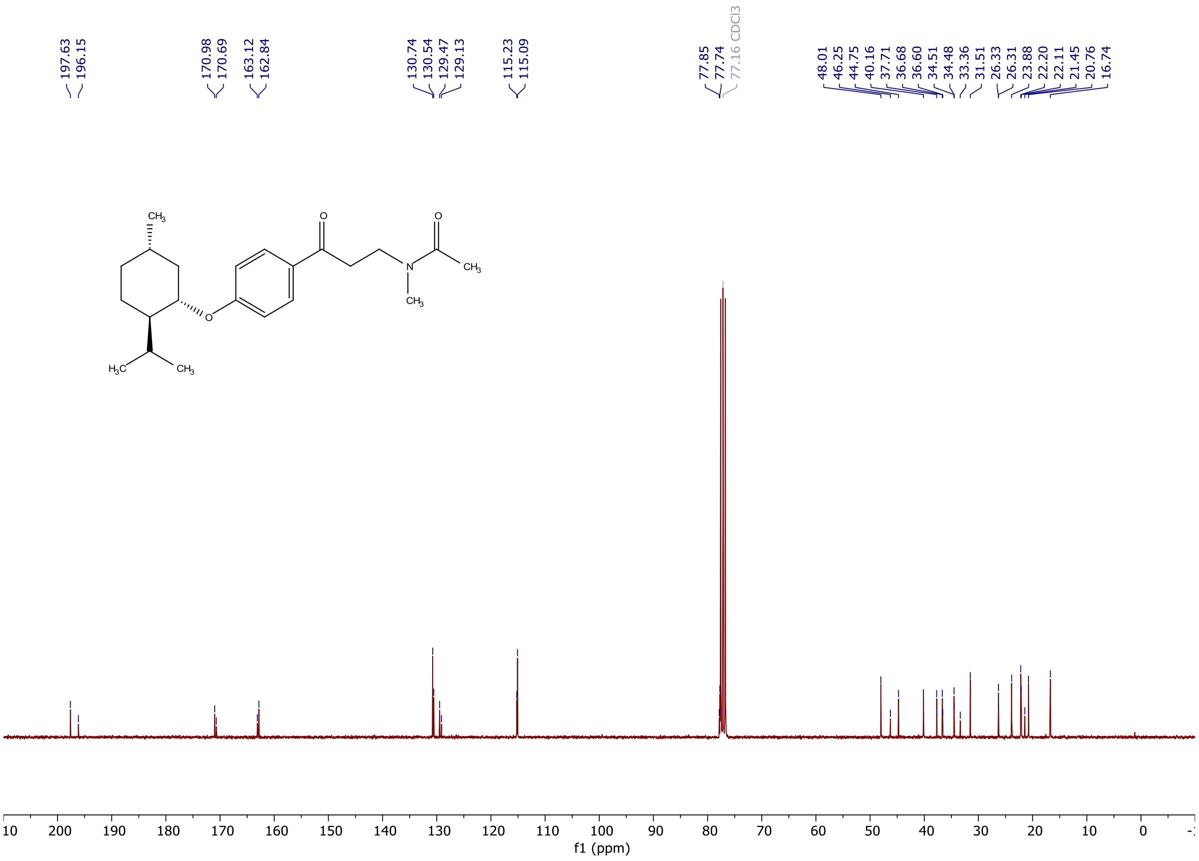

^{1}H NMR (300 MHz, $CDCl_3$) of **31**

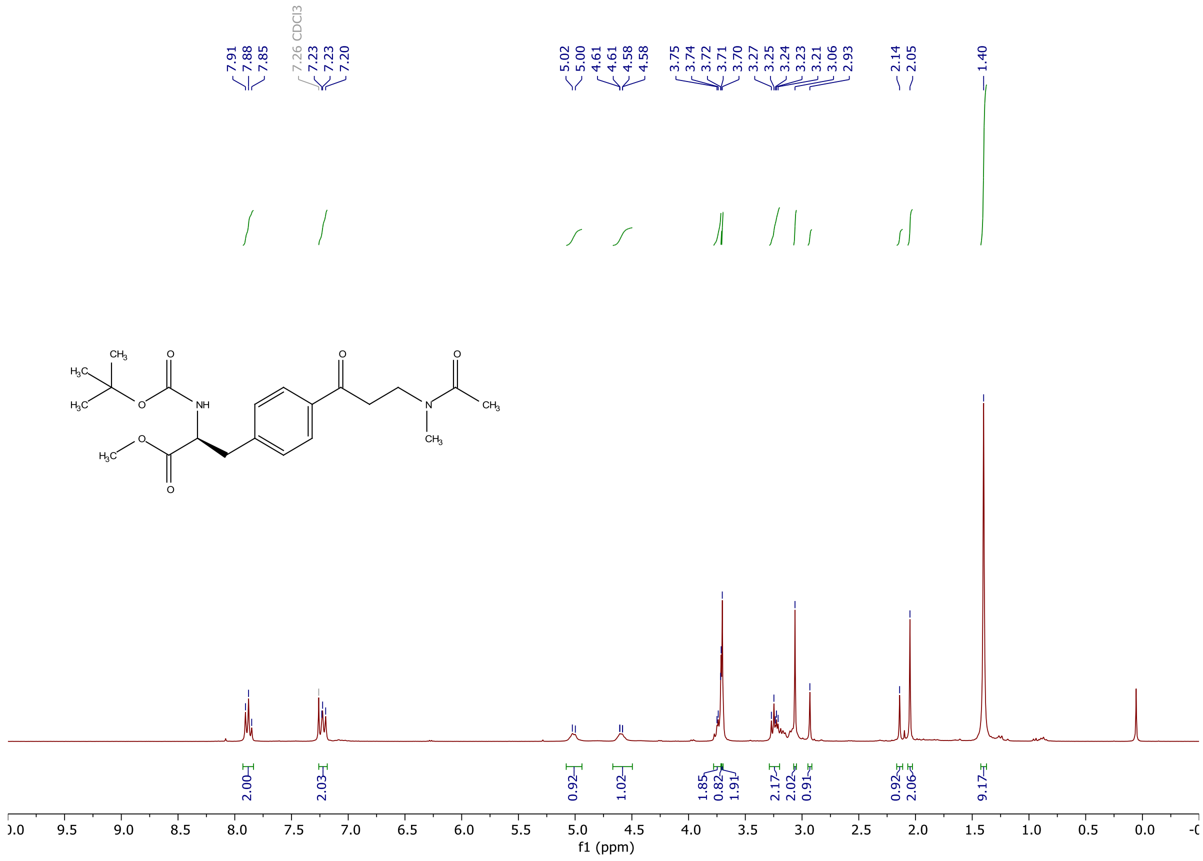

^{13}C NMR (75 MHz, $CDCl_3$) of **31**

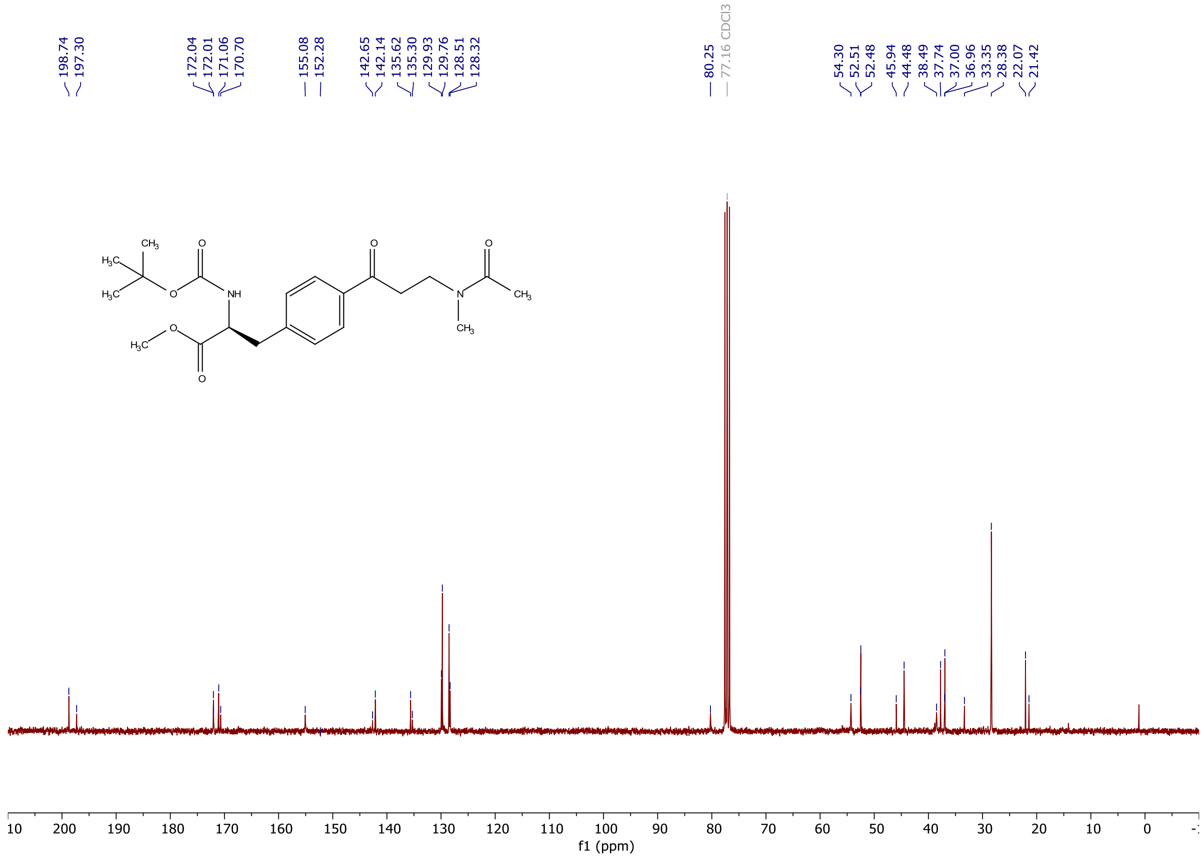

^{1}H NMR (300 MHz, $CDCl_3$) of **32**

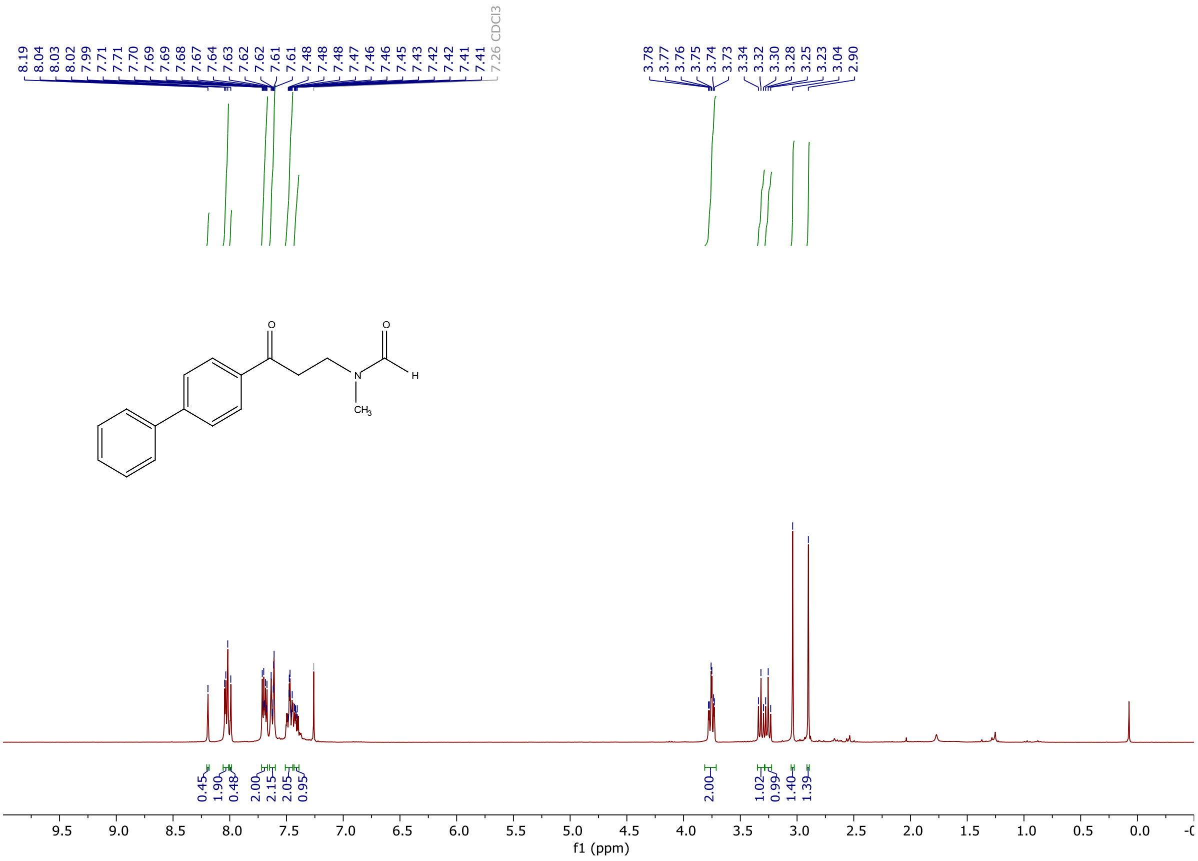

^{13}C NMR (75 MHz, $CDCl_3$) of **32**

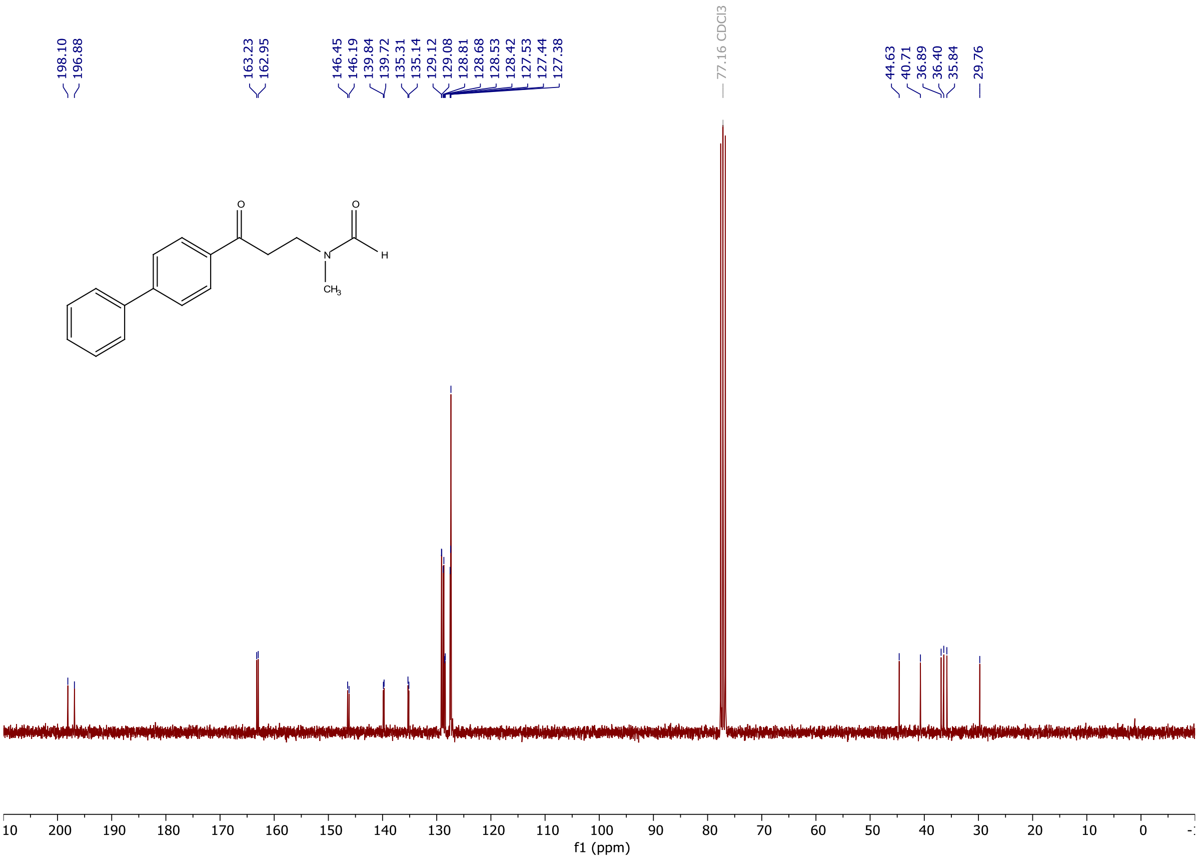

^{1}H NMR (300 MHz, $CDCl_3$) of **33**

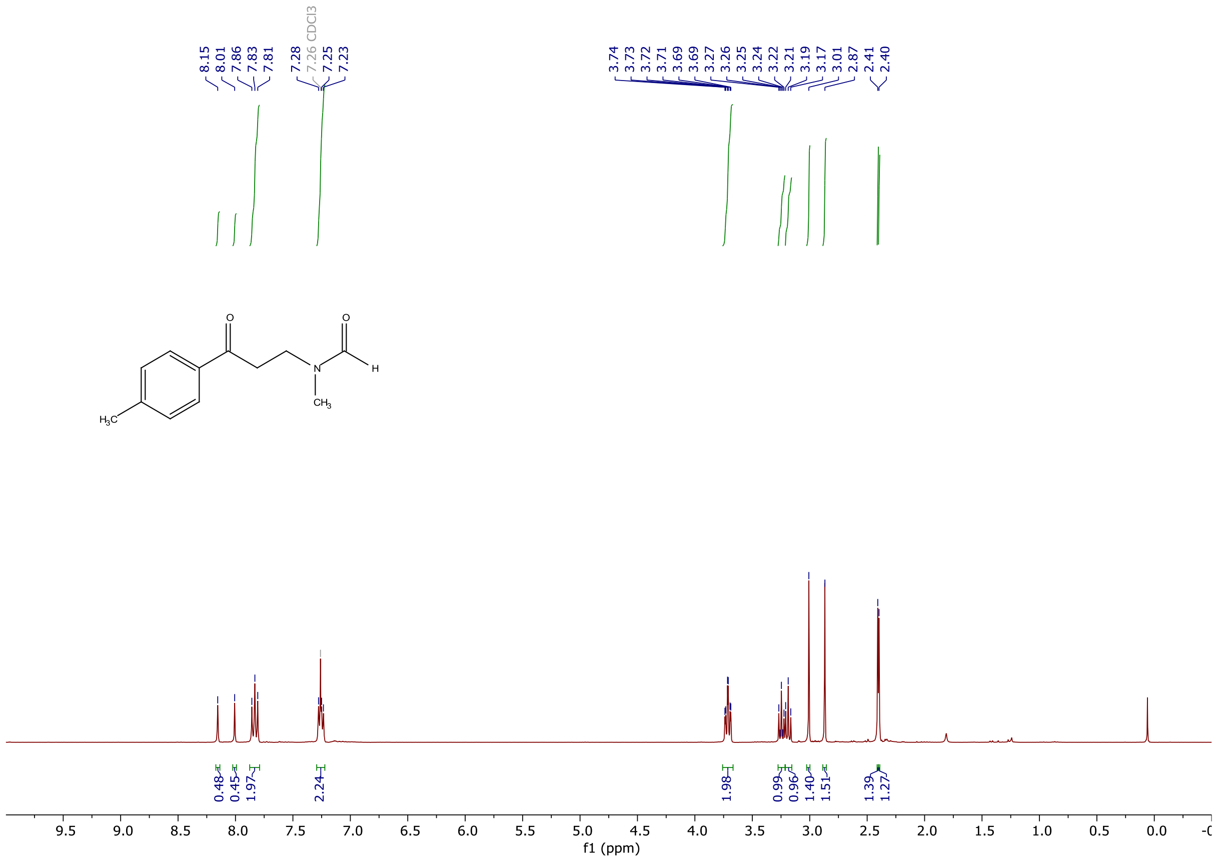

^{13}C NMR (75 MHz, $CDCl_3$) of **33**

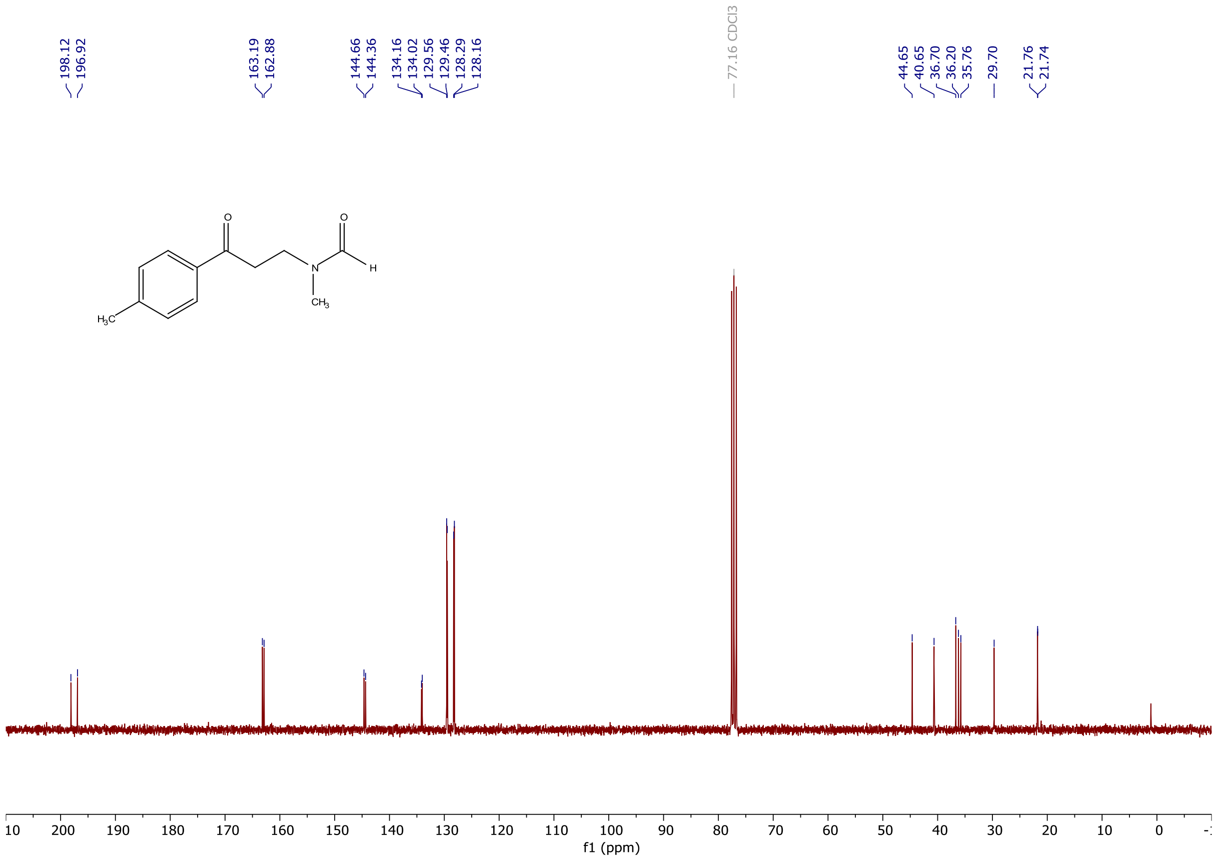

^{1}H NMR (300 MHz, $CDCl_3$) of **34**

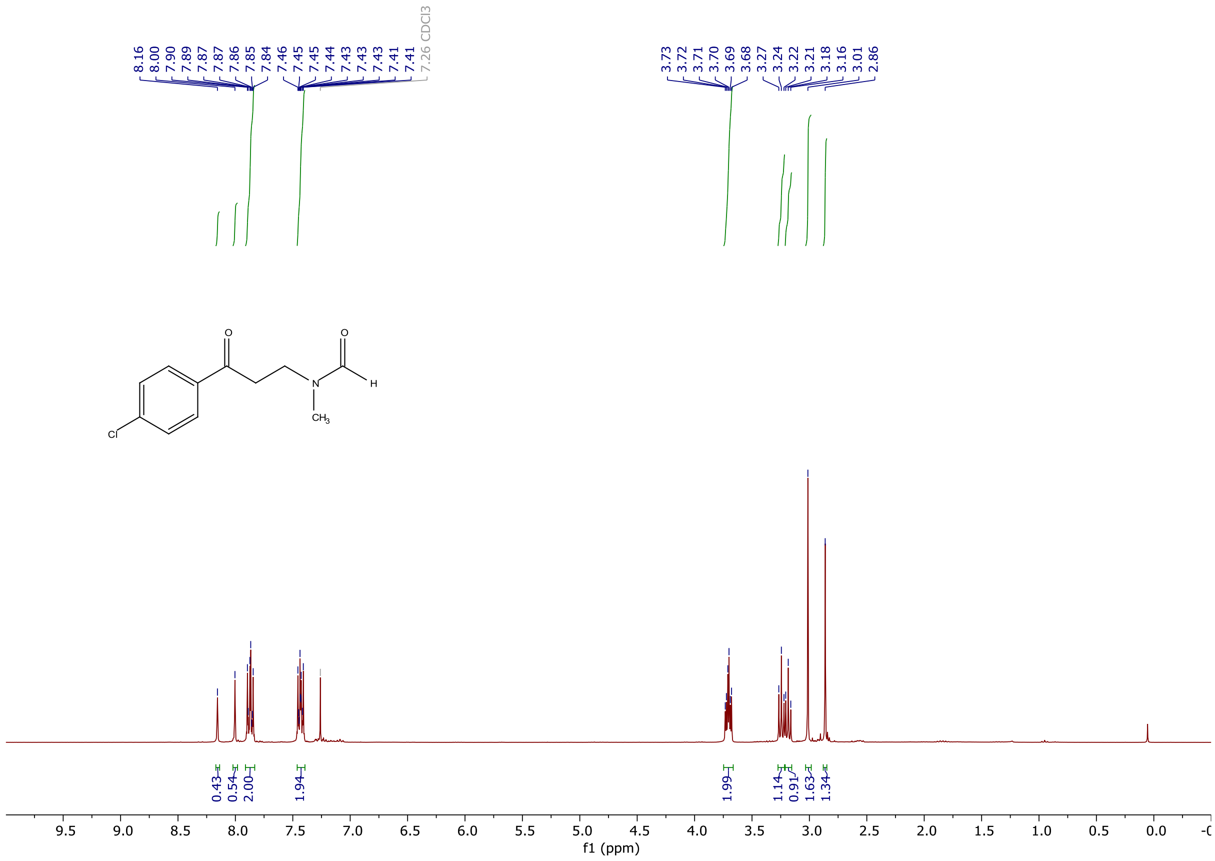

^{13}C NMR (75 MHz, $CDCl_3$) of **34**

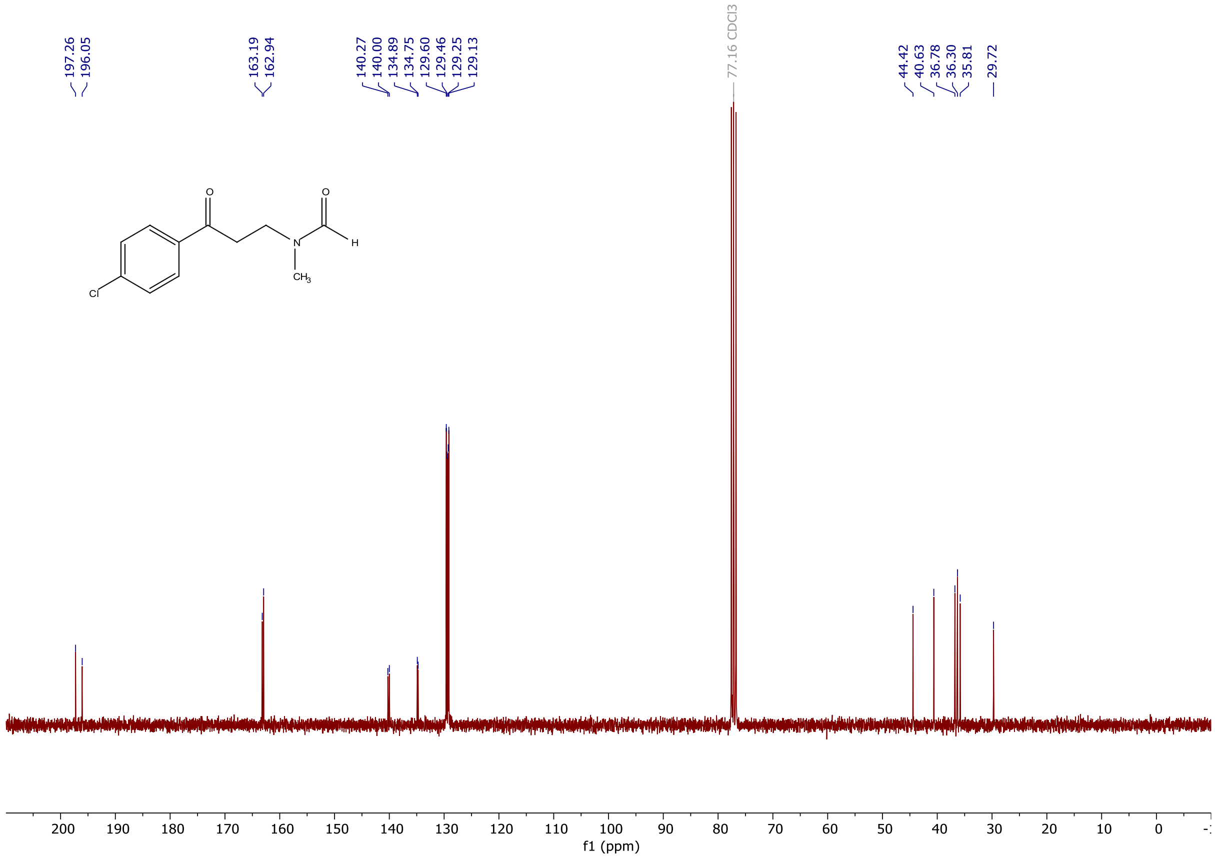

^{1}H NMR (300 MHz, $CDCl_3$) of **35**

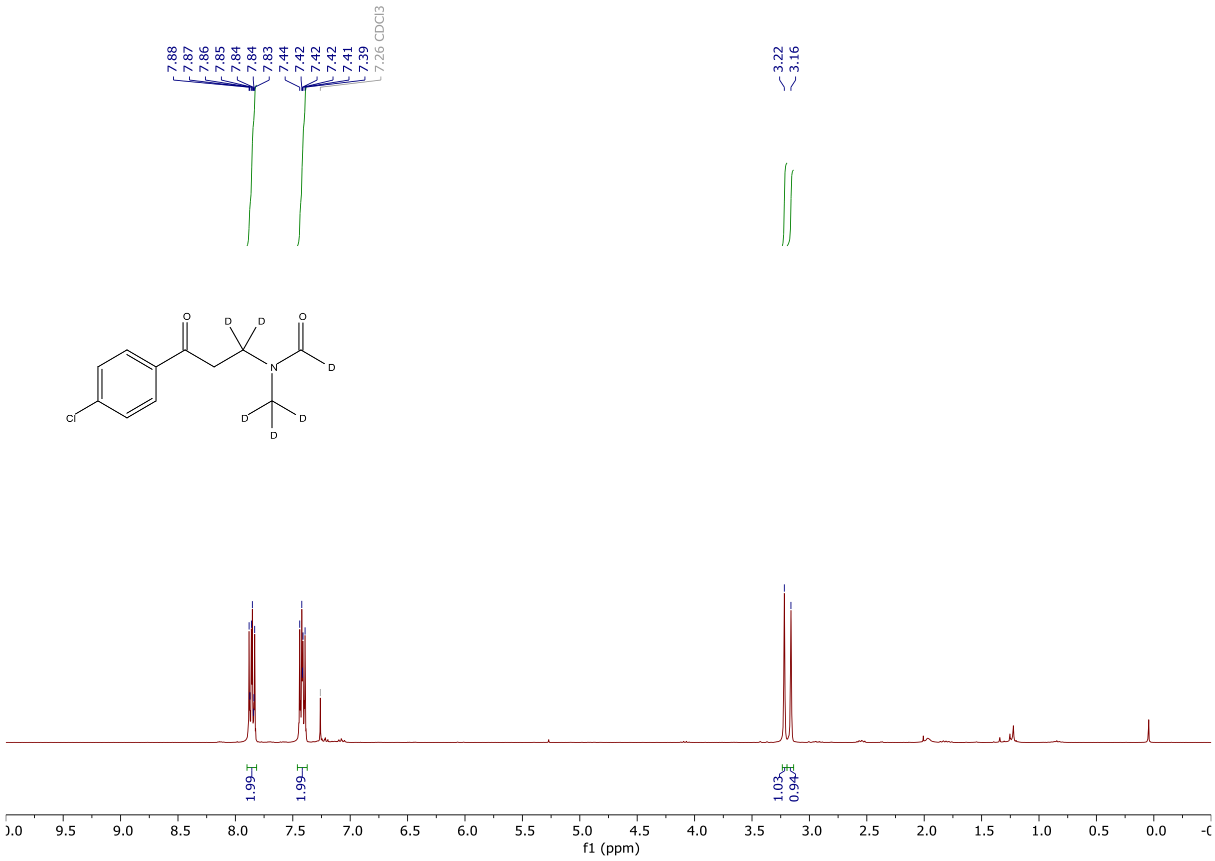

^{13}C NMR (75 MHz, $CDCl_3$) of **35**

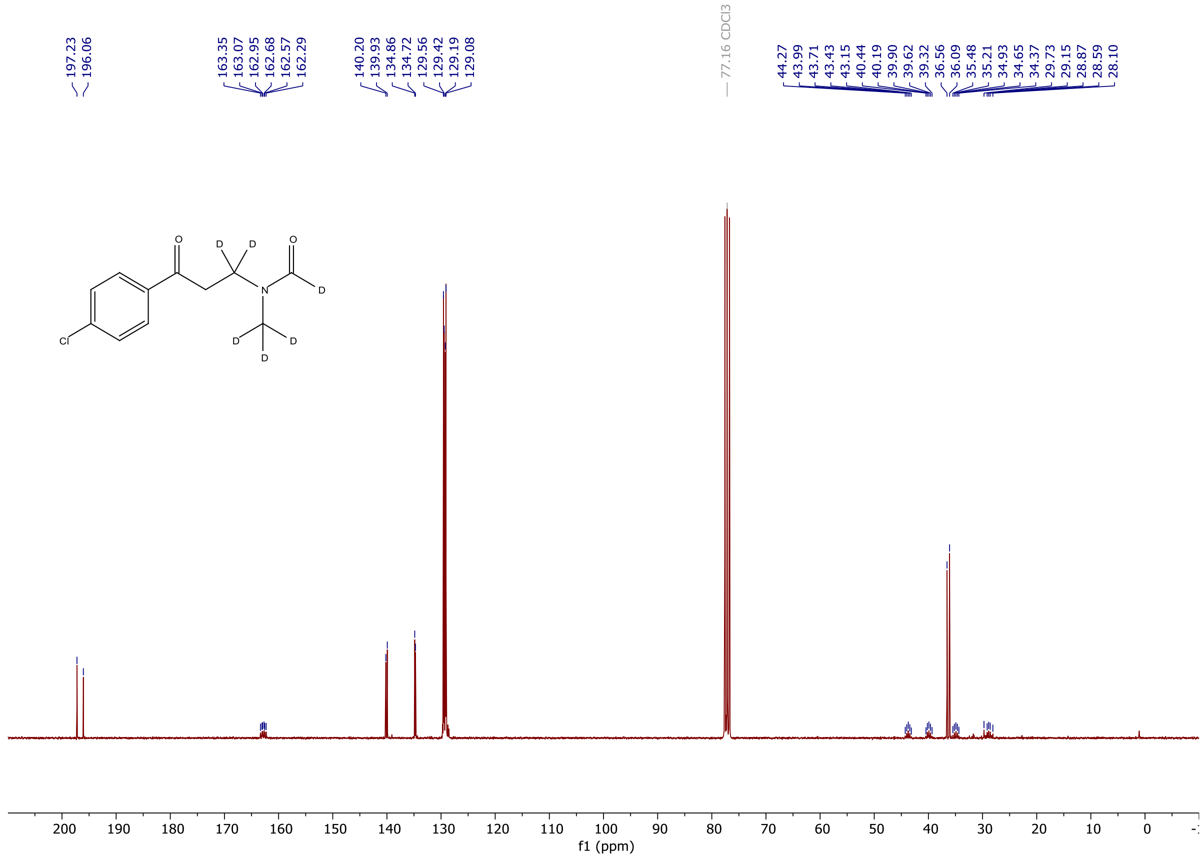

^{1}H NMR (300 MHz, $CDCl_3$) of **36**

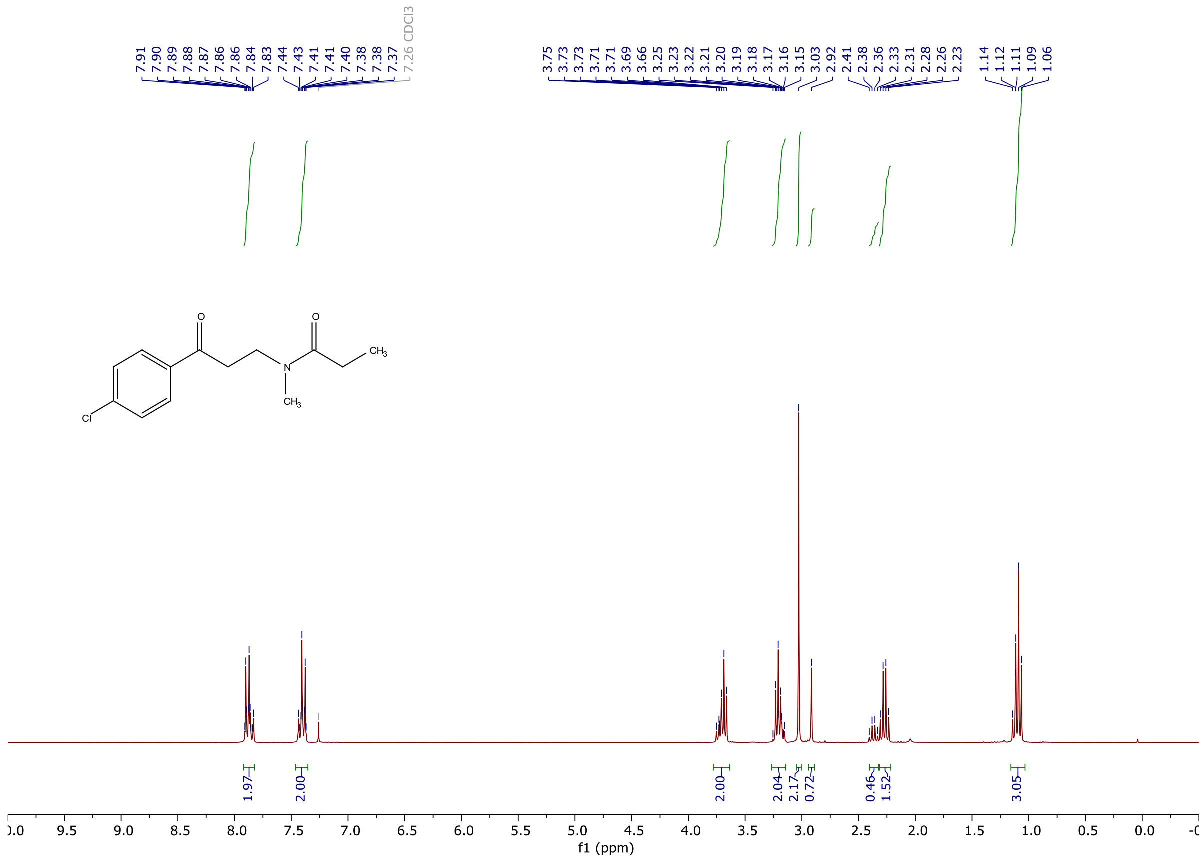

^{13}C NMR (75 MHz, $CDCl_3$) of **36**

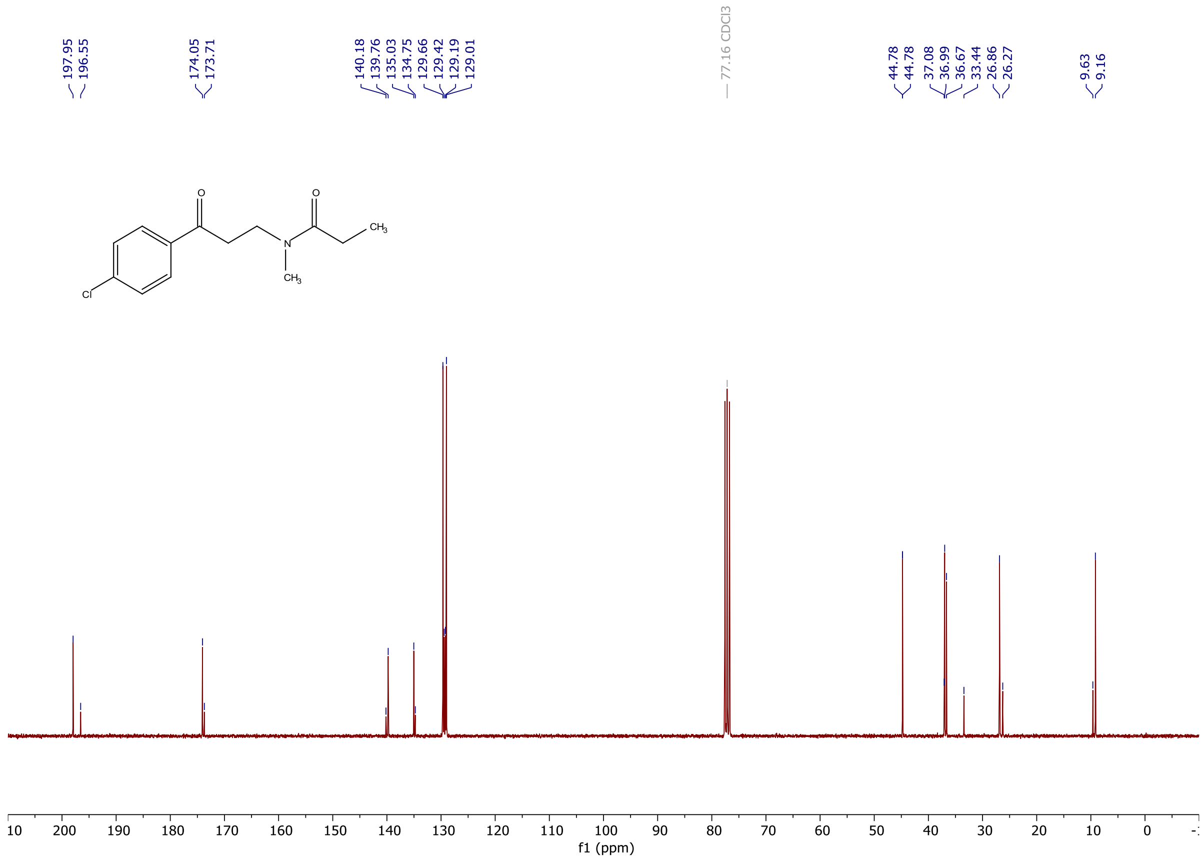

^{1}H NMR (300 MHz, $CDCl_3$) of **37**

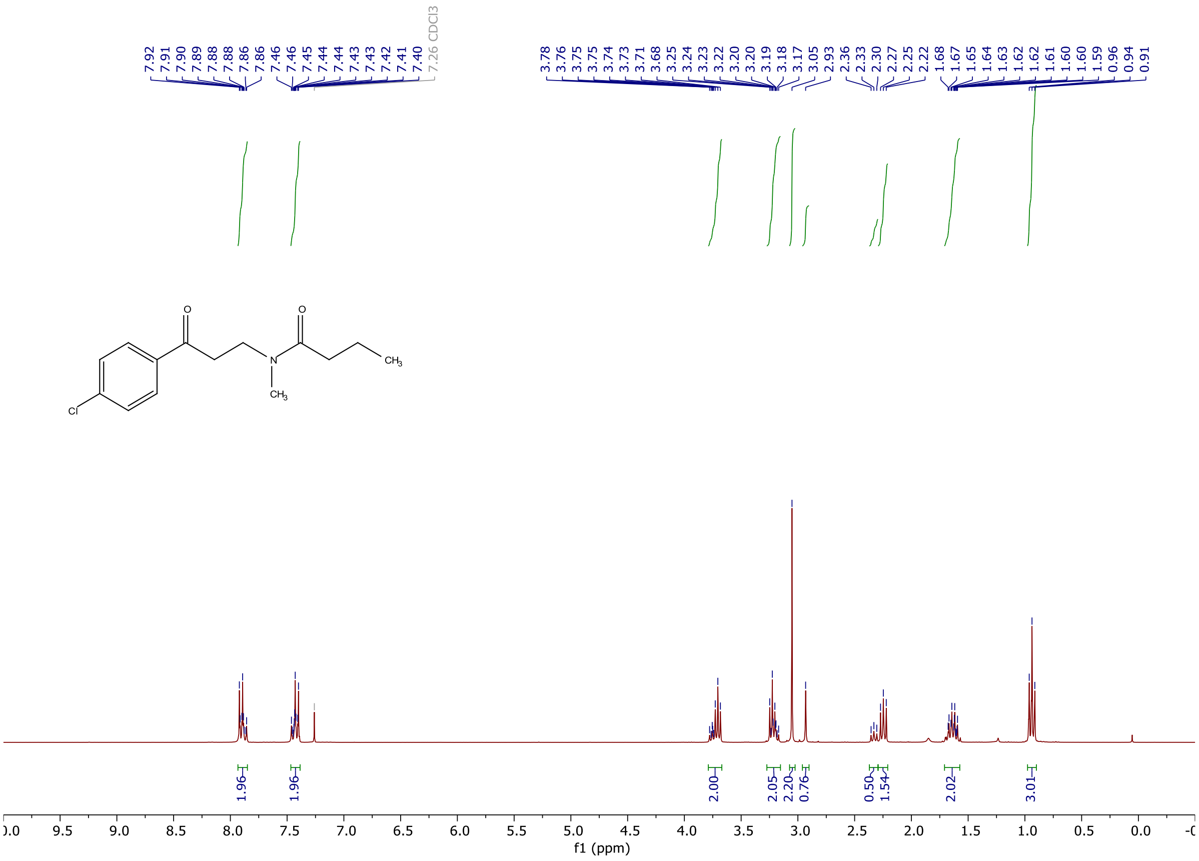

^{13}C NMR (75 MHz, $CDCl_3$) of **37**

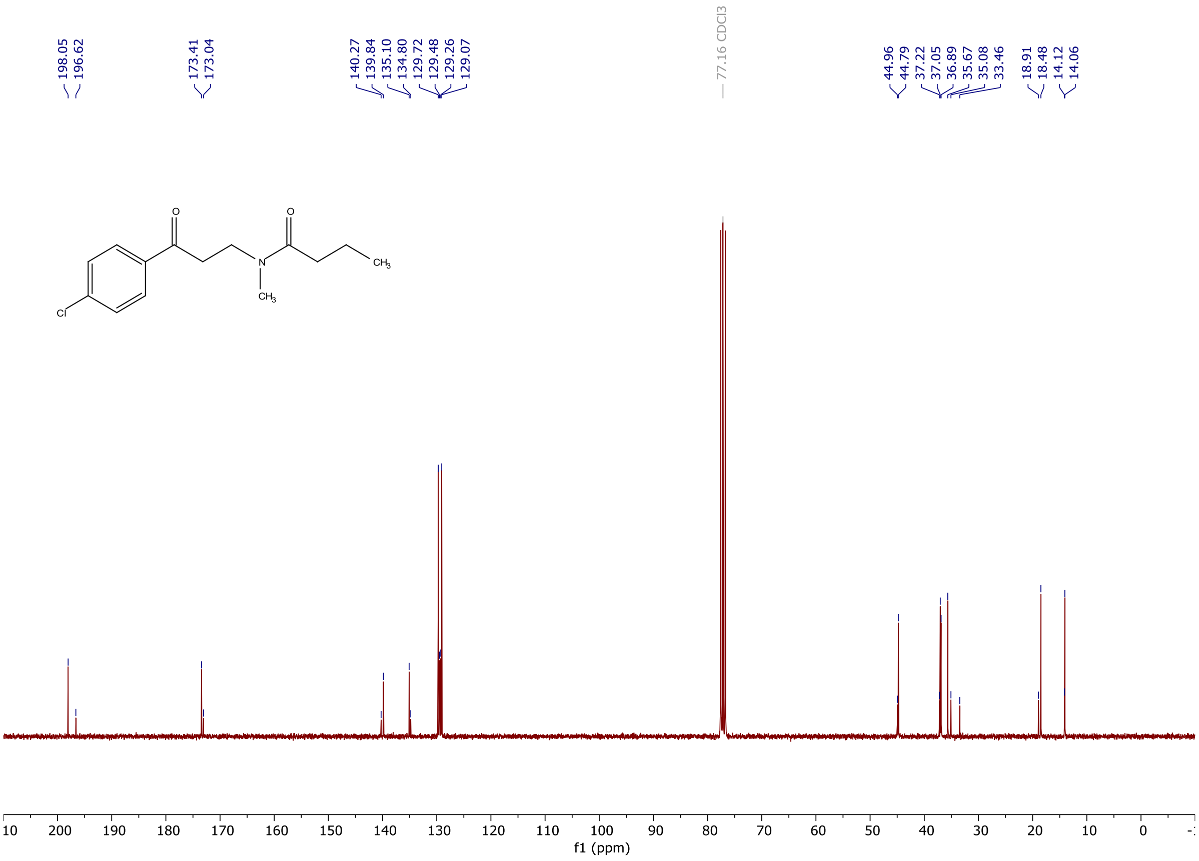

^{1}H NMR (300 MHz, $CDCl_3$) of **38**

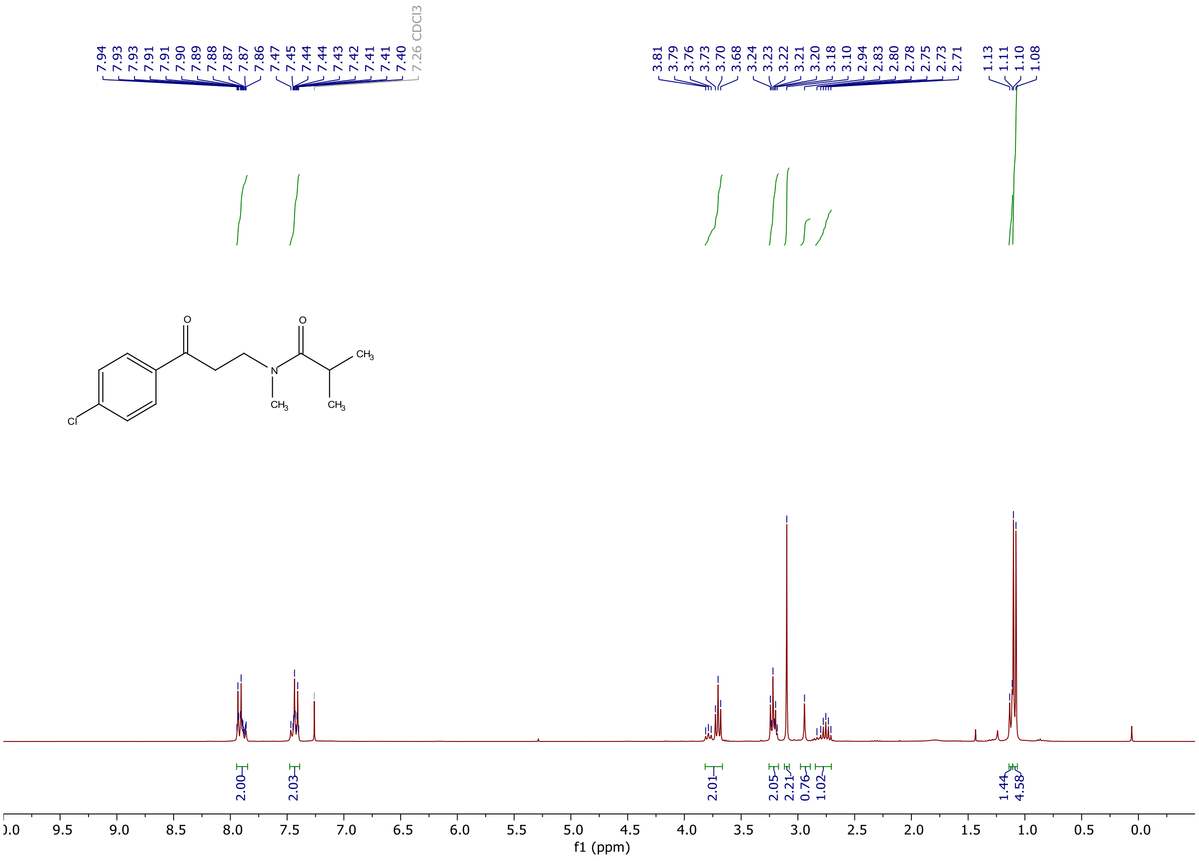

^{13}C NMR (75 MHz, $CDCl_3$) of **38**

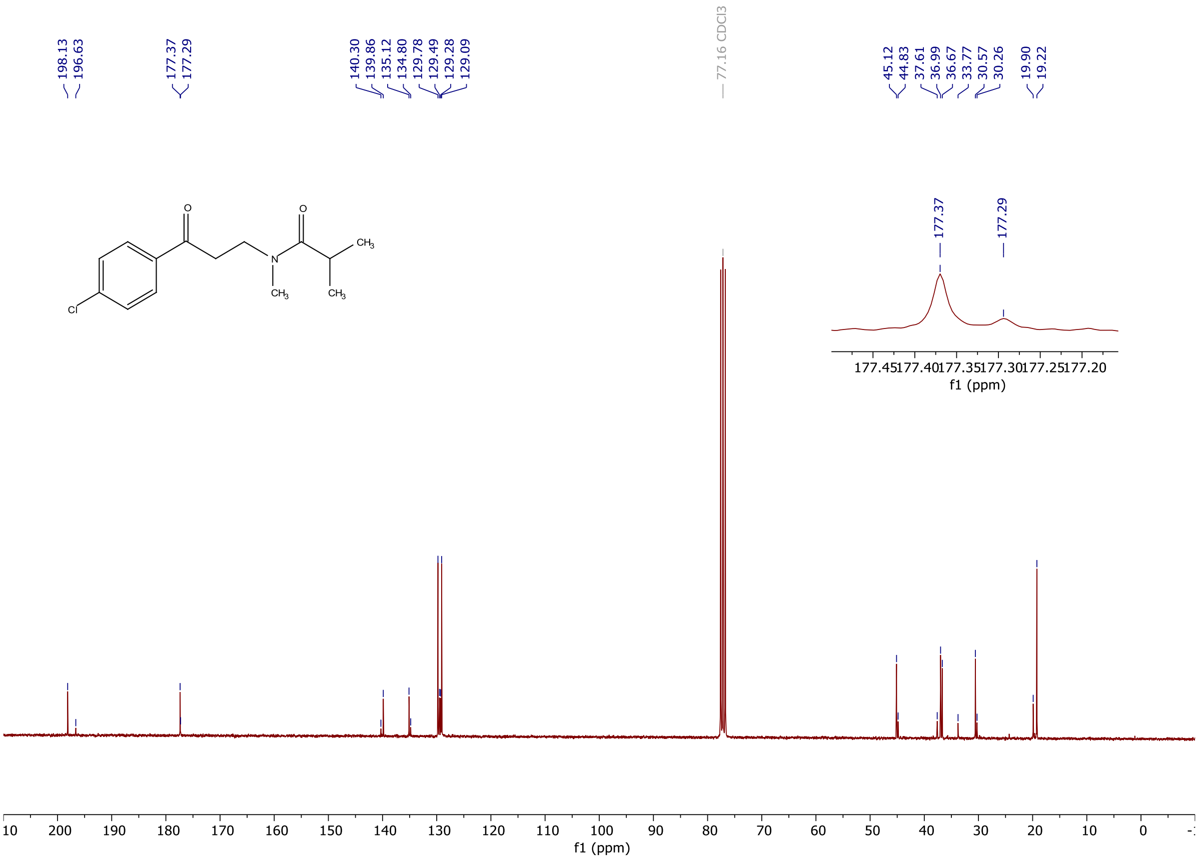

^{1}H NMR (300 MHz, $CDCl_3$) of **39**

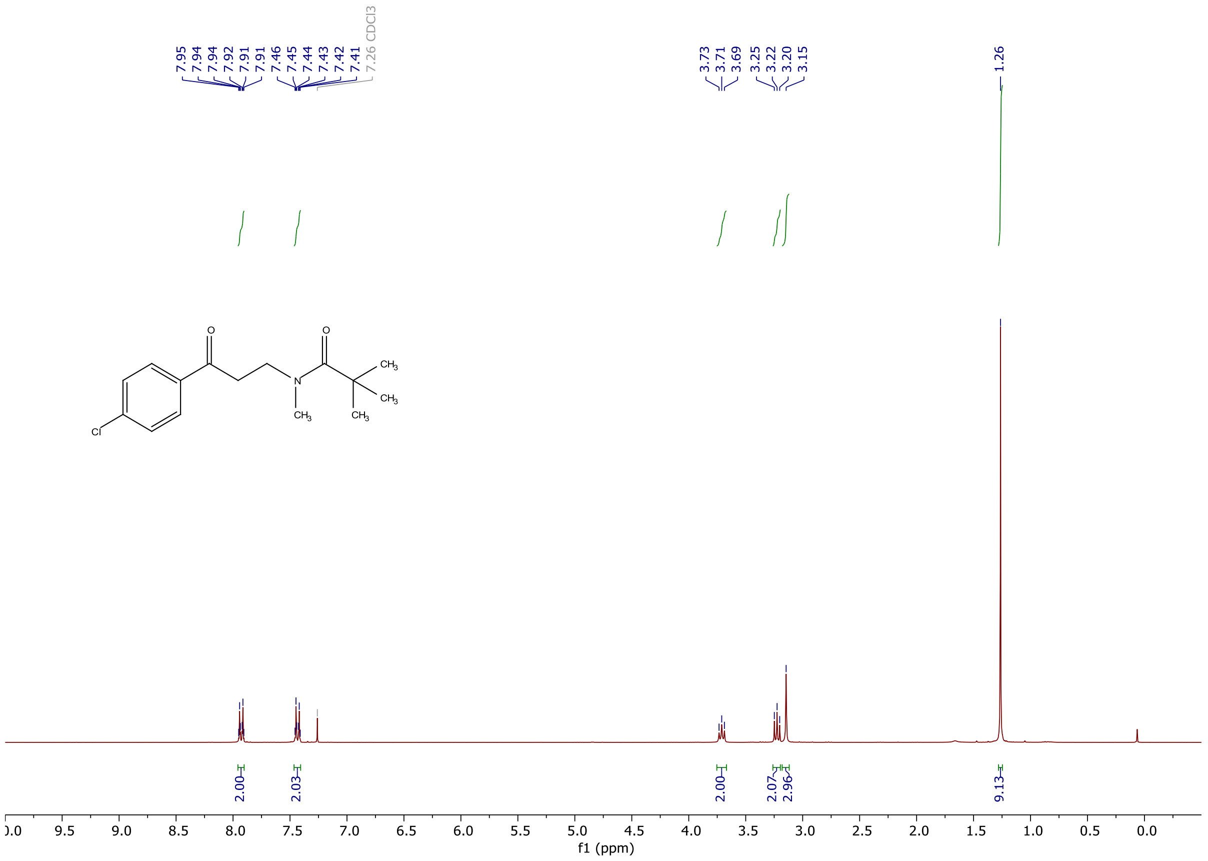

^{13}C NMR (75 MHz, $CDCl_3$) of **39**

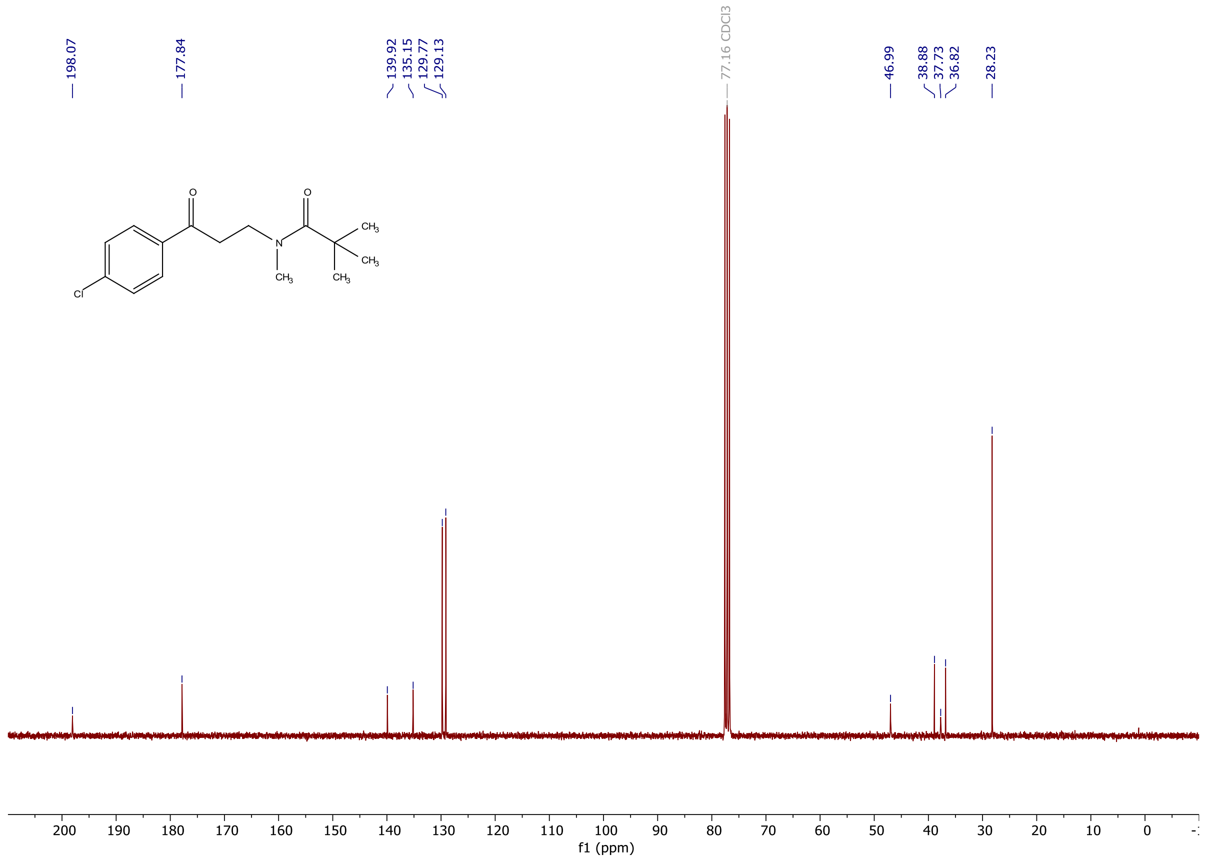

^{1}H NMR (300 MHz, $CDCl_3$) of **40**

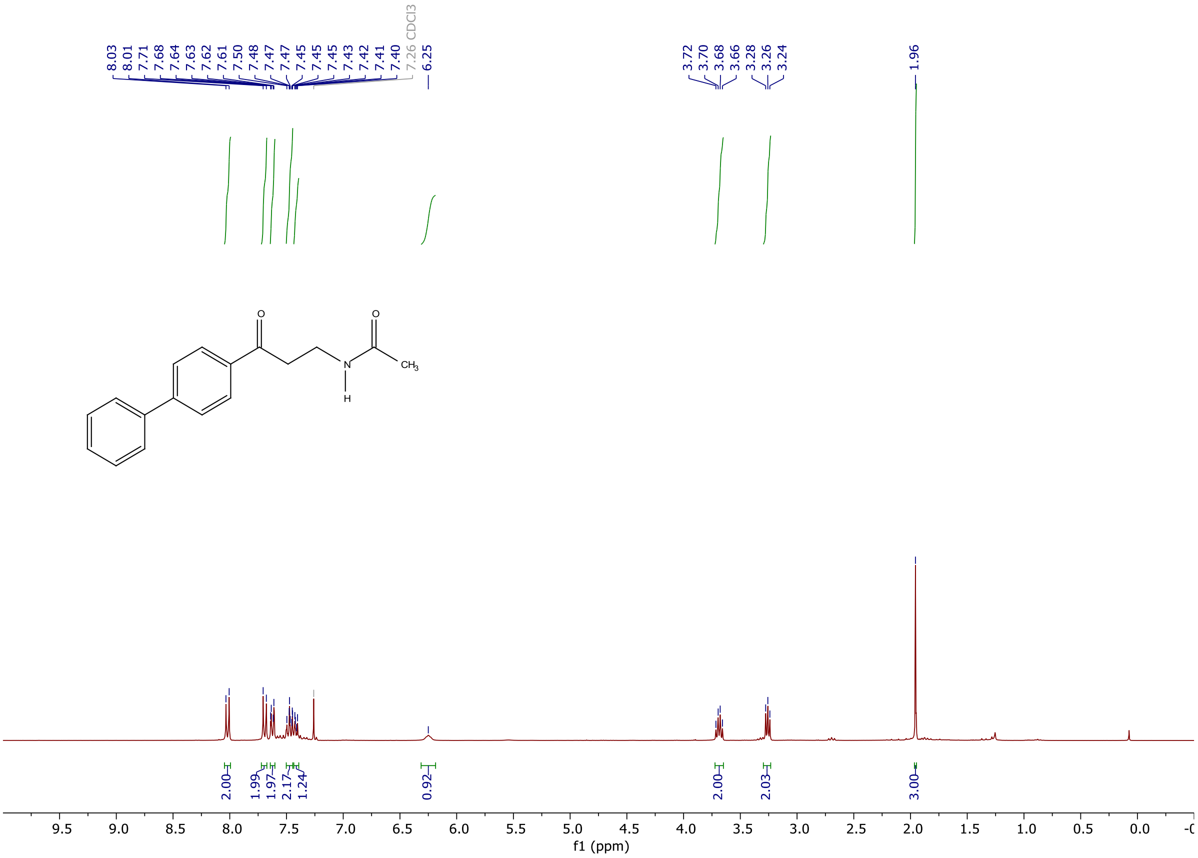

^{13}C NMR (75 MHz, $CDCl_3$) of **40**

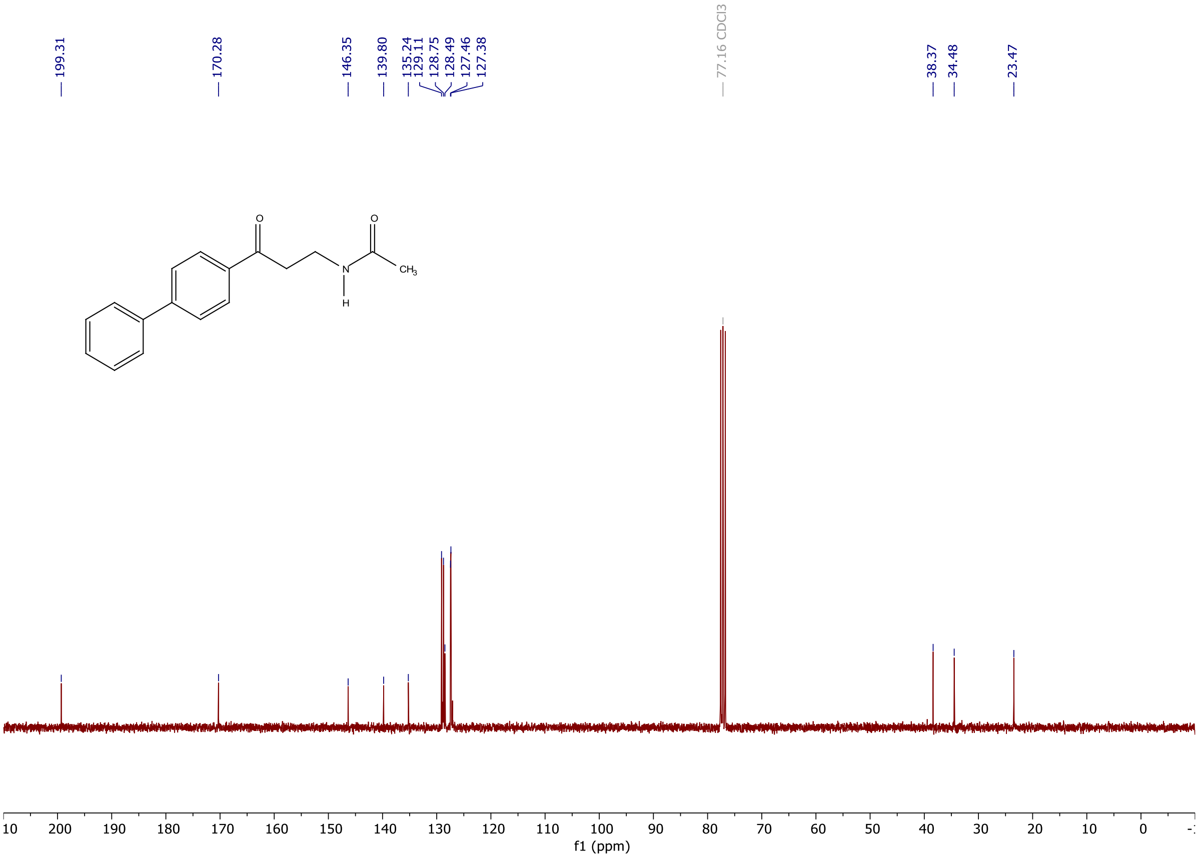

^{1}H NMR (300 MHz, $CDCl_3$) of **41**

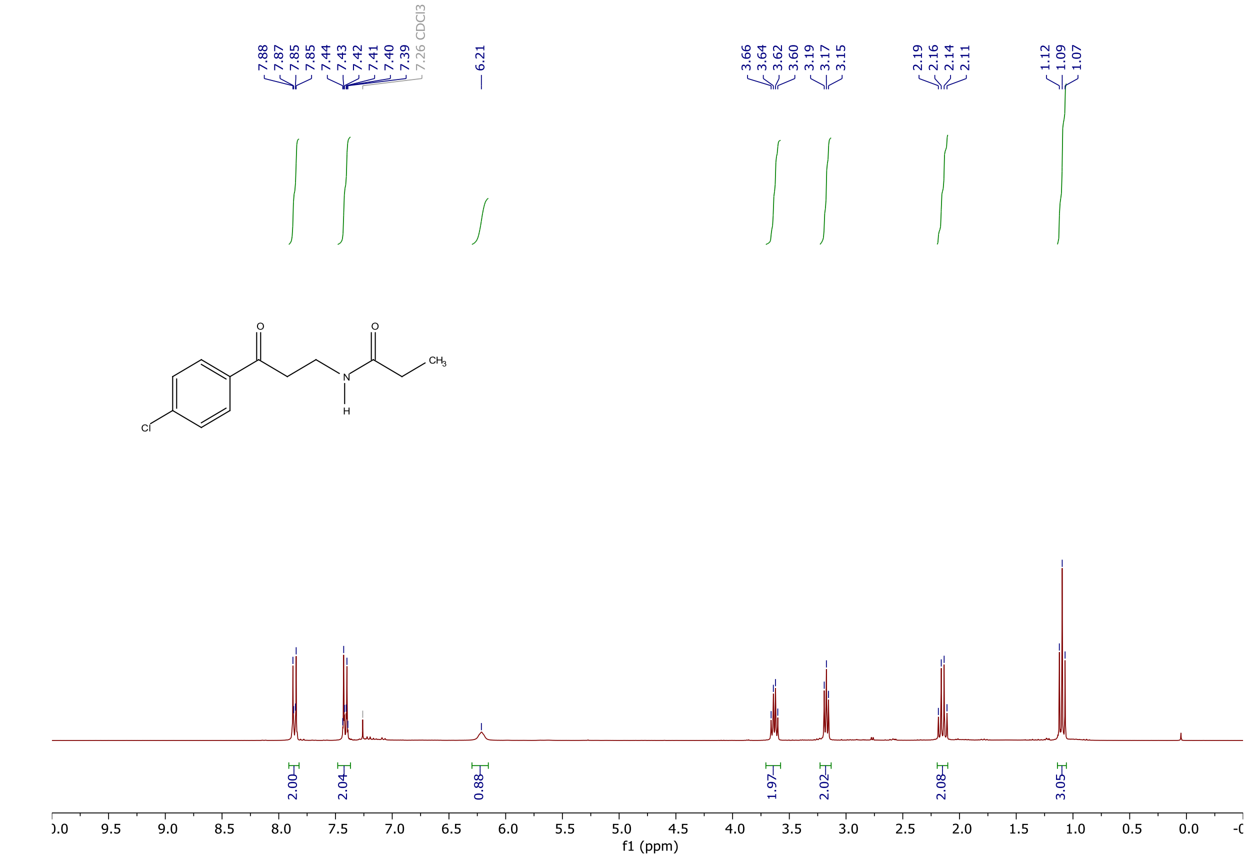

^{13}C NMR (75 MHz, $CDCl_3$) of **41**

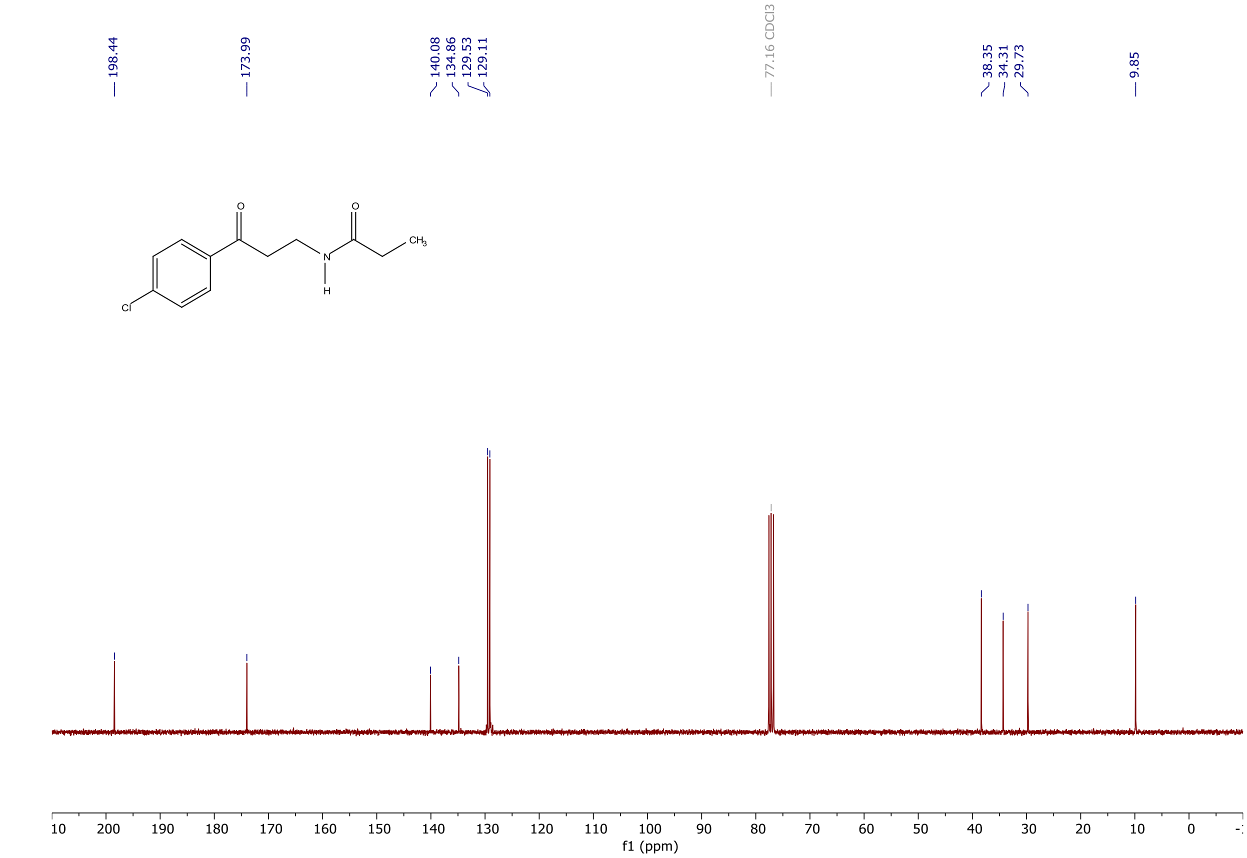

^{1}H NMR (300 MHz, $CDCl_3$) of **42a** and **42b**

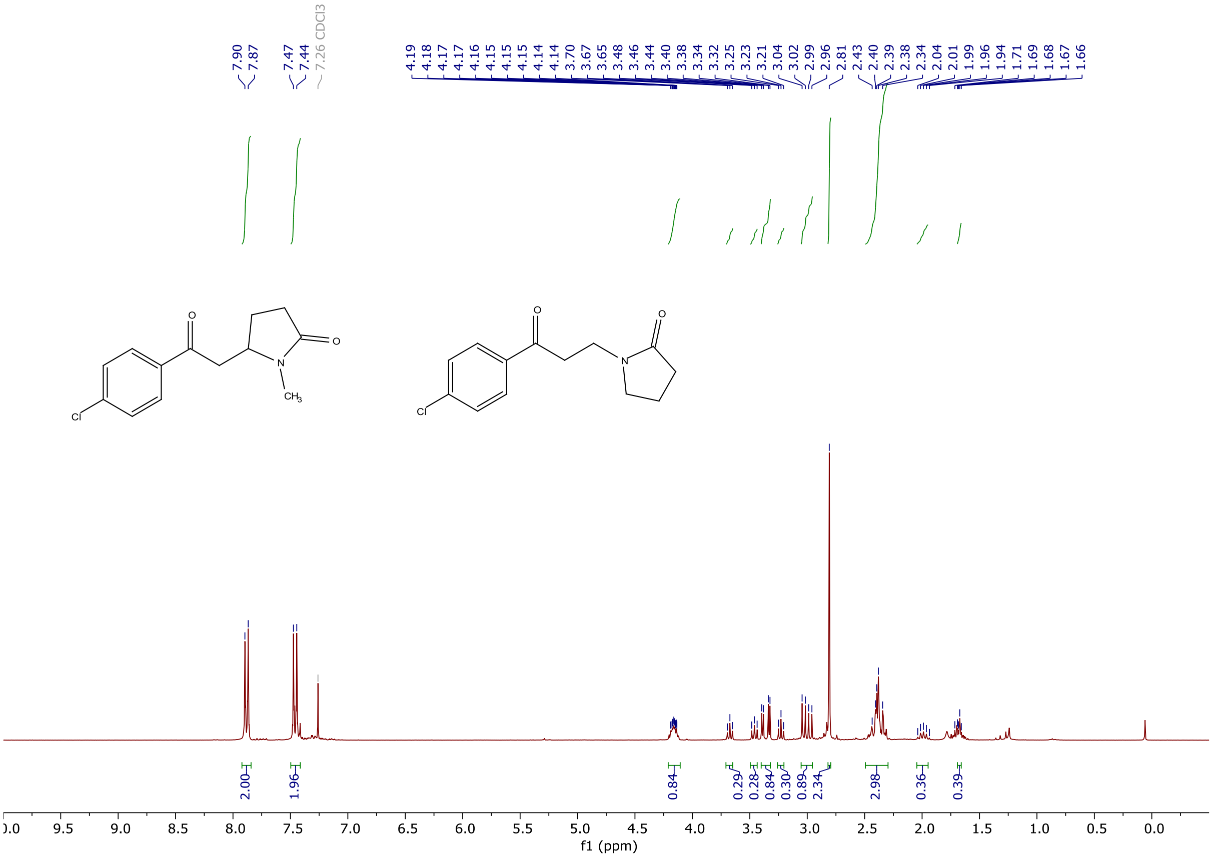

^{13}C NMR (75 MHz, $CDCl_3$) of **42a** and **42b**

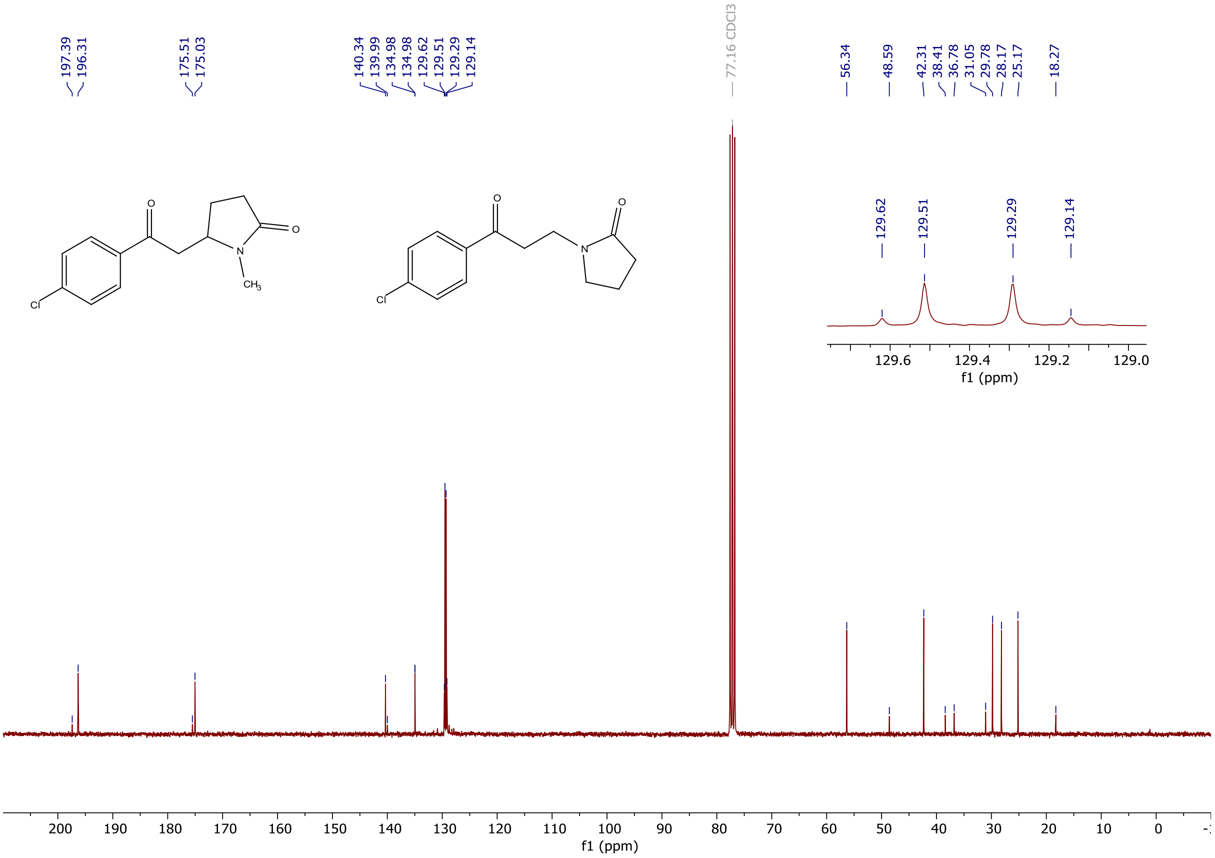

GC chromatogram and EI-mass spectra of **42a** and **42b**

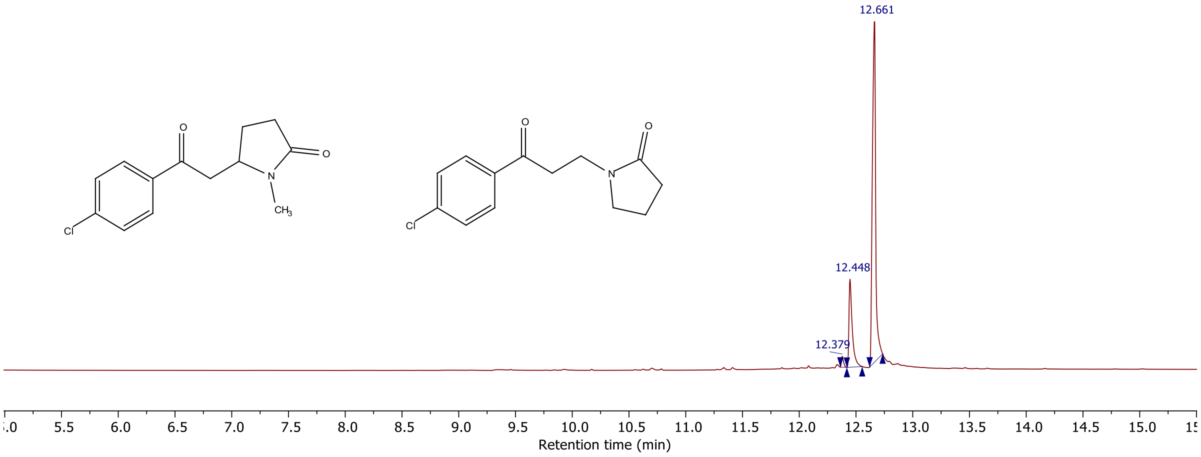

Rt = 12.4 min

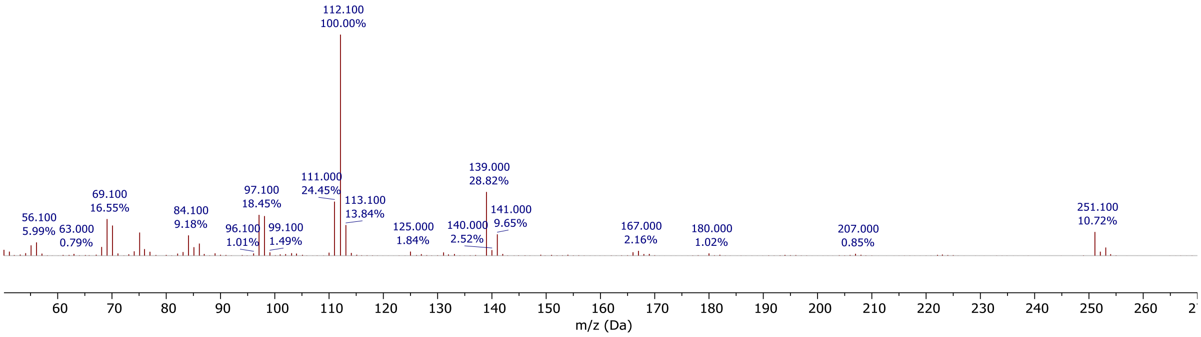

Rt = 12.6 min

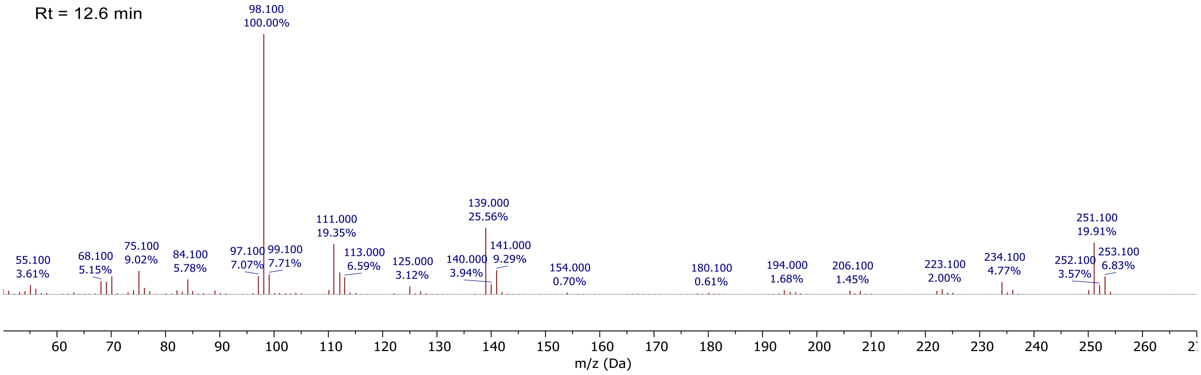

^{1}H NMR (300 MHz, $CDCl_3$) of **43**

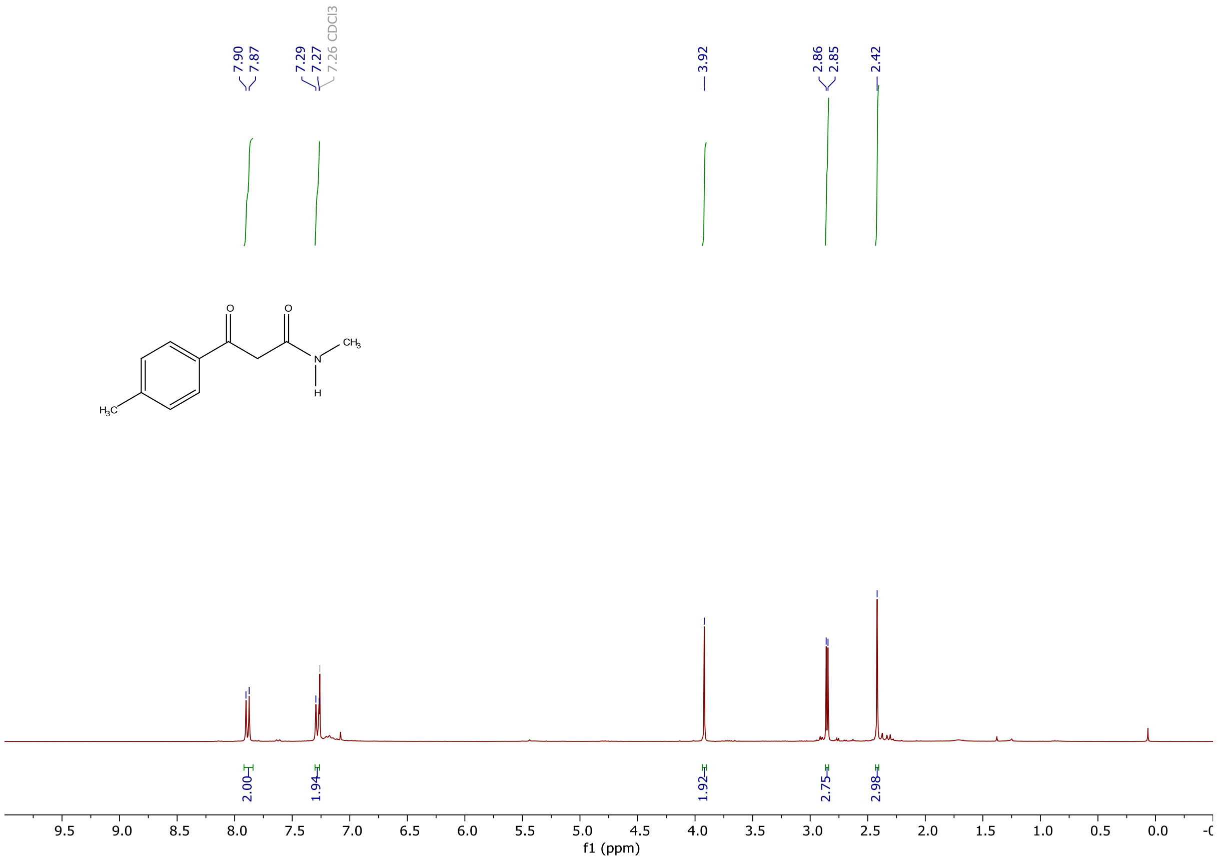

^{13}C NMR (75 MHz, $CDCl_3$) of **43**

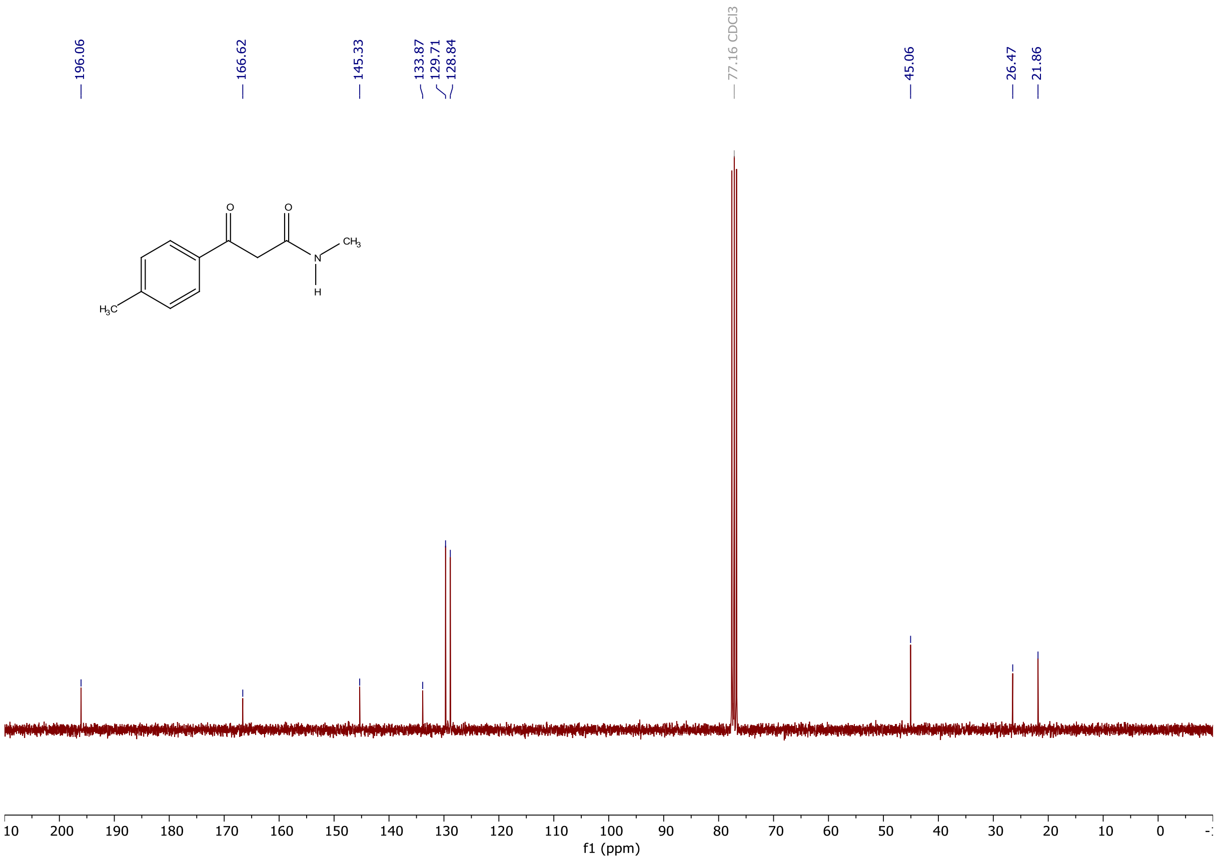

14. NMR spectra of post-functionalizations

^1H NMR (300 MHz, $CDCl_3$) of **45**

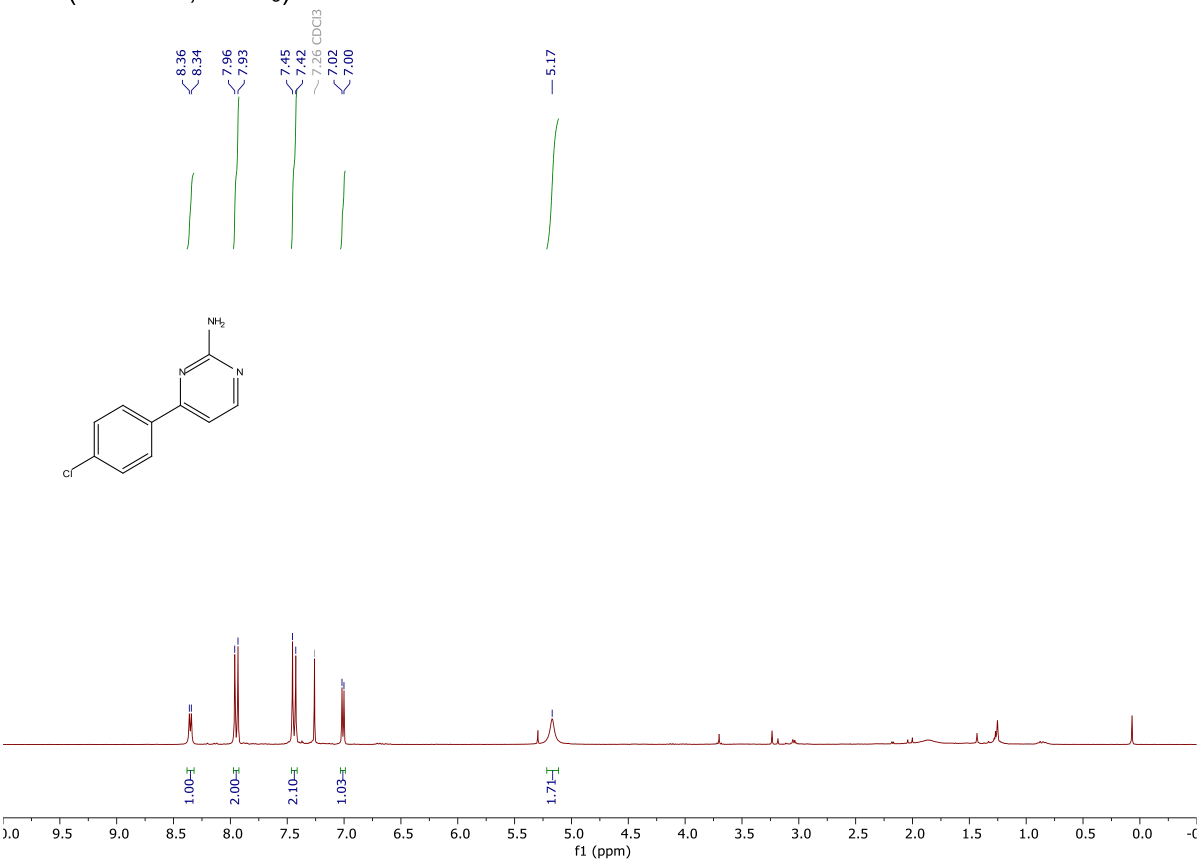

^{13}C NMR (75 MHz, $CDCl_3$) of **45**

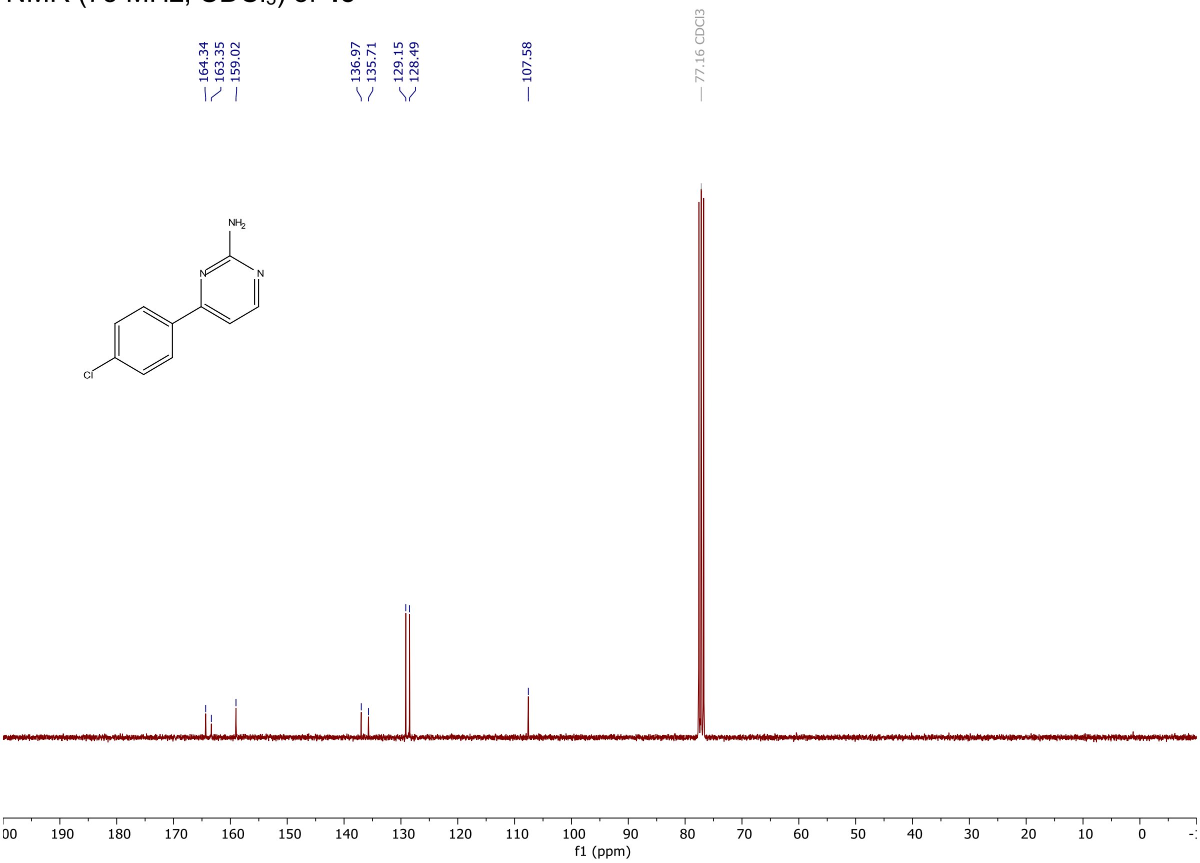

^{1}H NMR (300 MHz, $CDCl_3$) of **46**

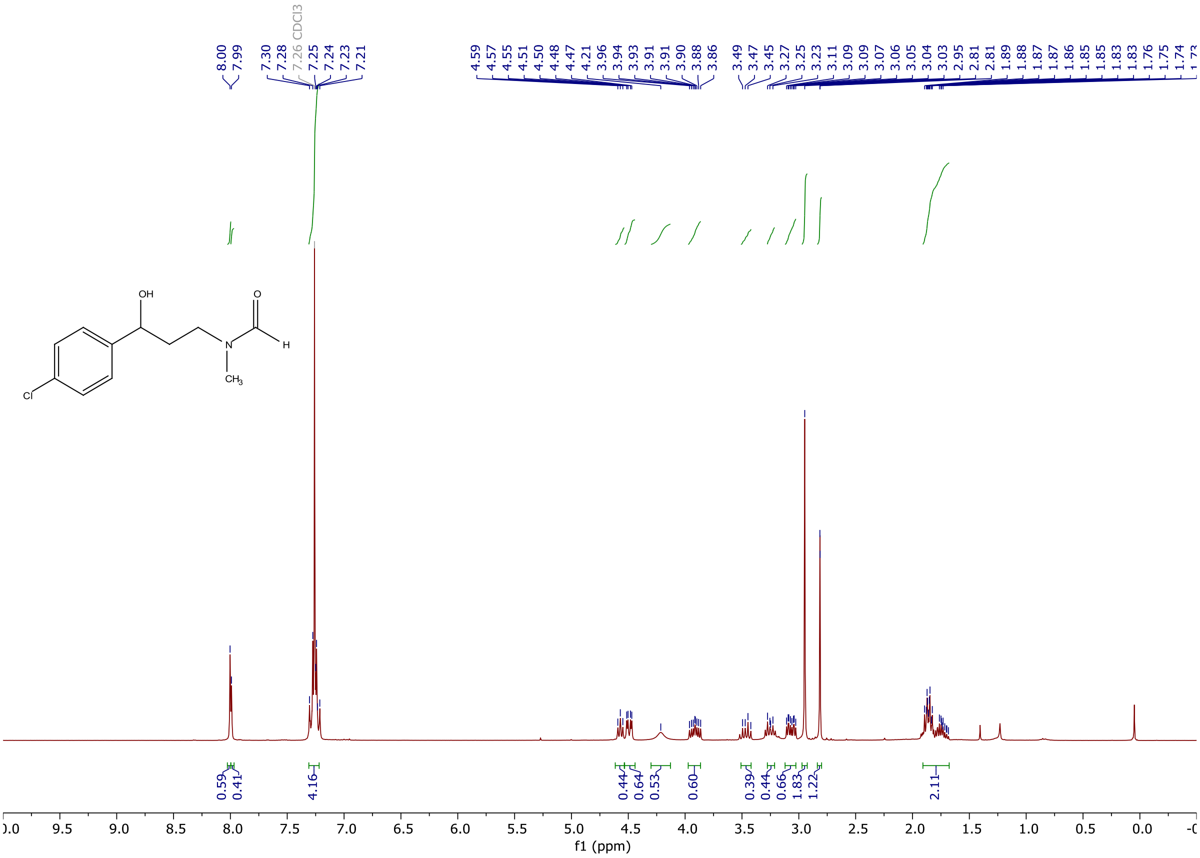

^{13}C NMR (75 MHz, $CDCl_3$) of **46**

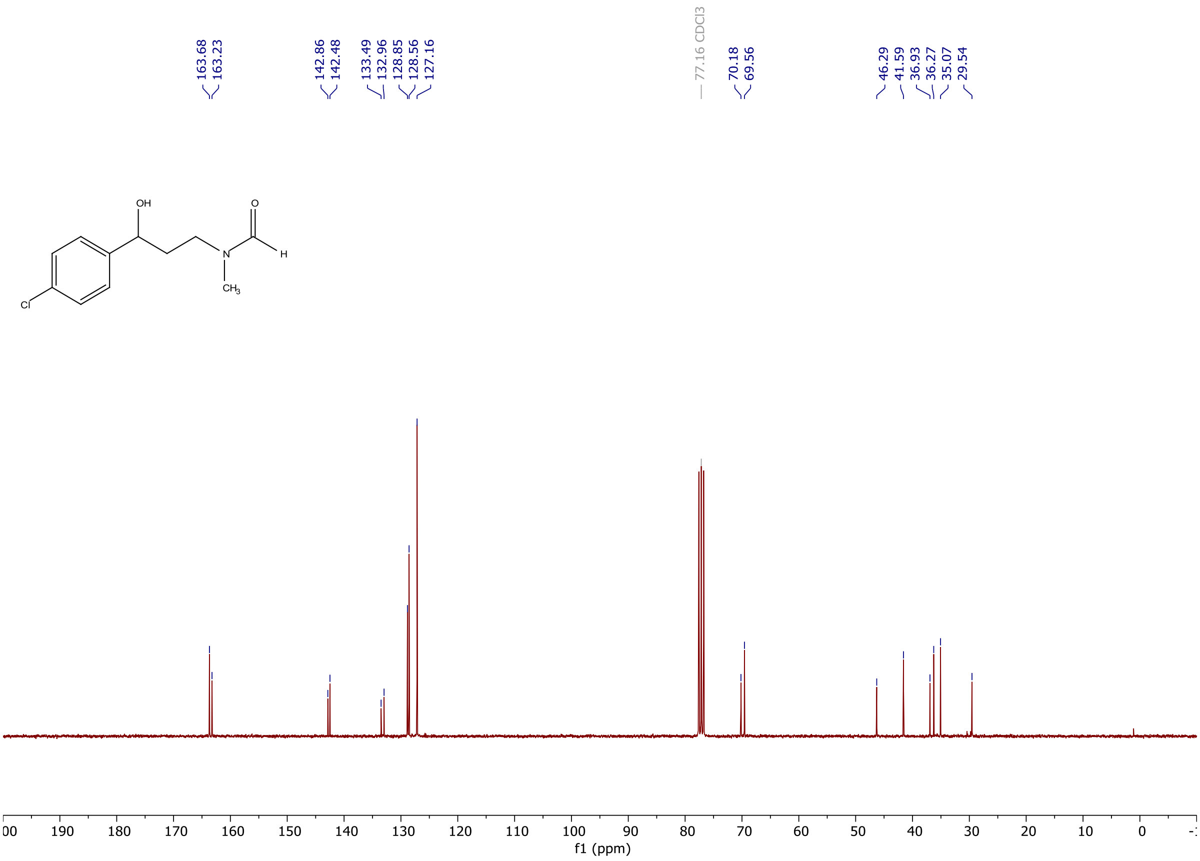

^{1}H NMR (300 MHz, $CDCl_3$) of **47**

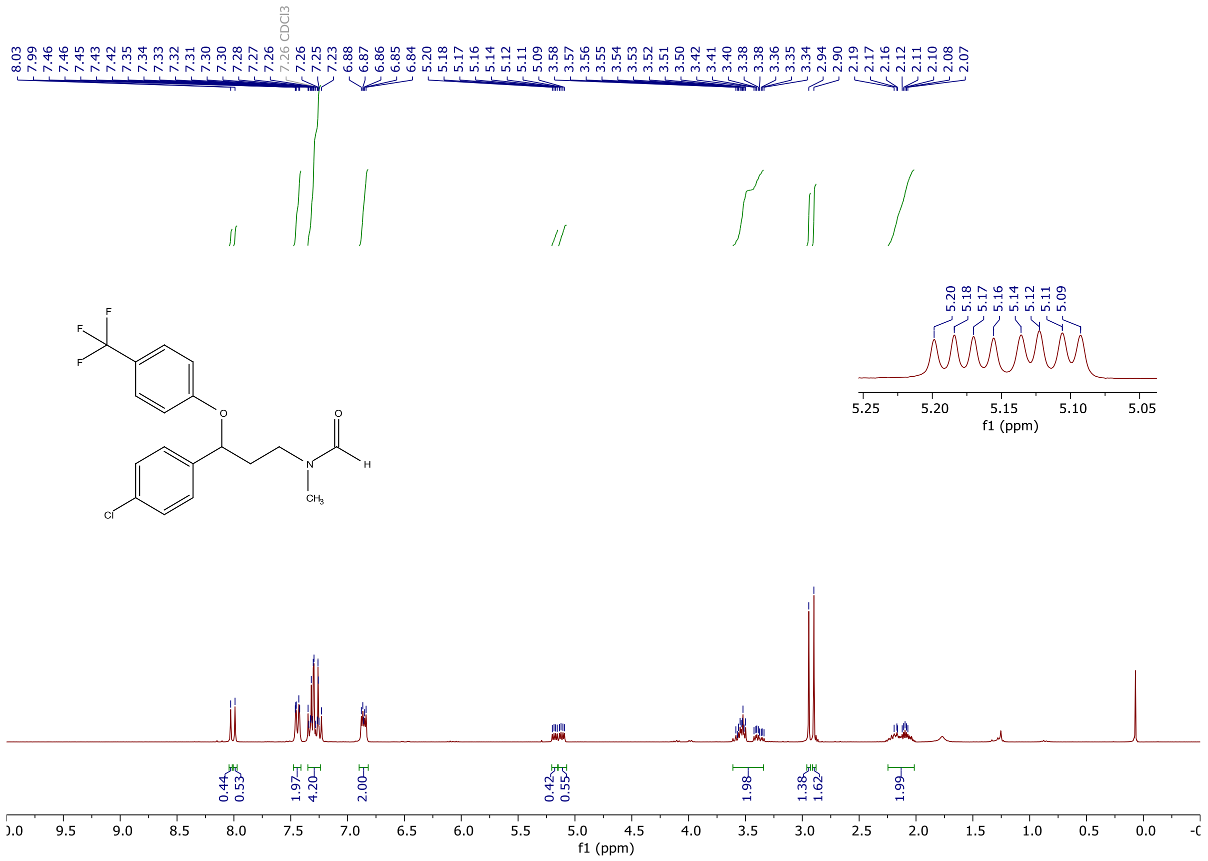

^{13}C NMR (75 MHz, $CDCl_3$) of **47**

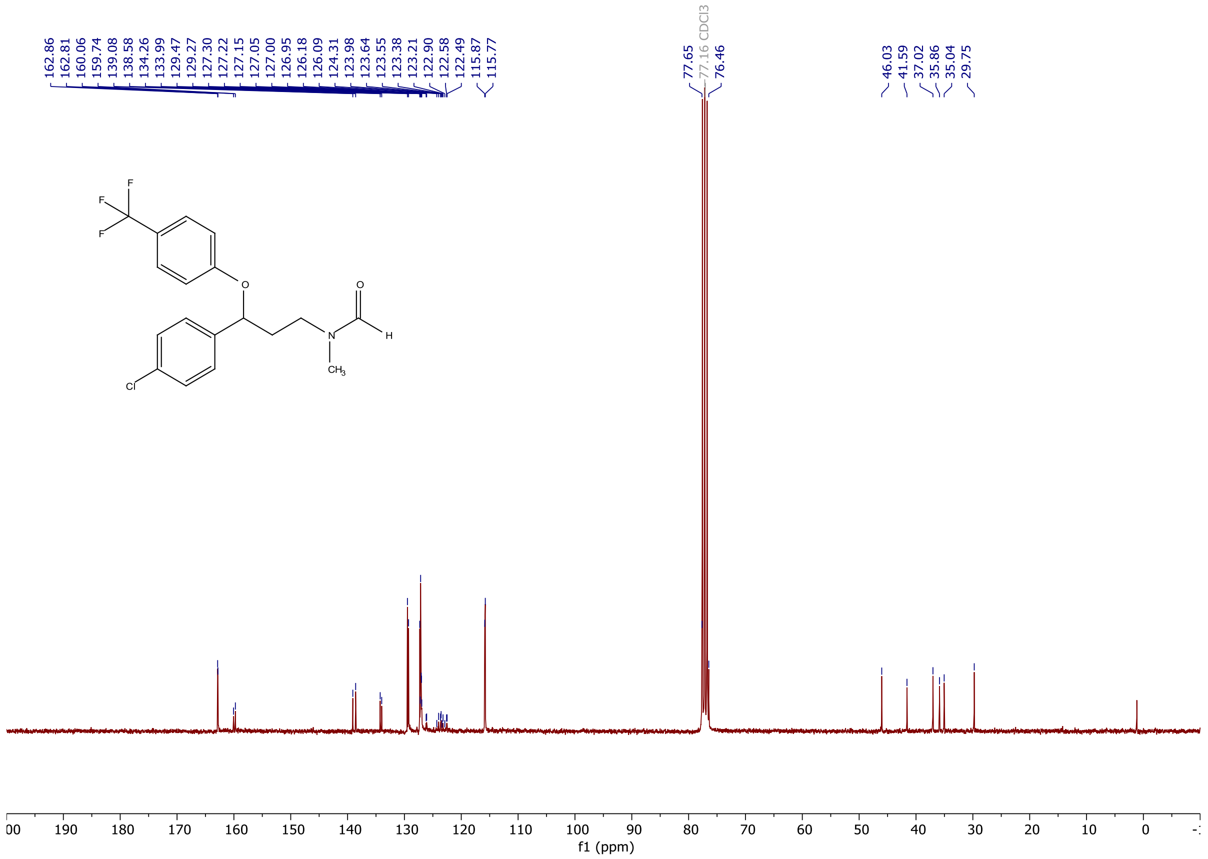

^{19}F NMR (282 MHz, $CDCl_3$) of **47**

-61.65
-61.72

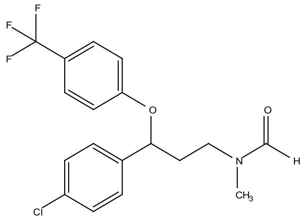

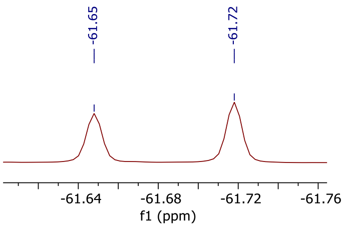

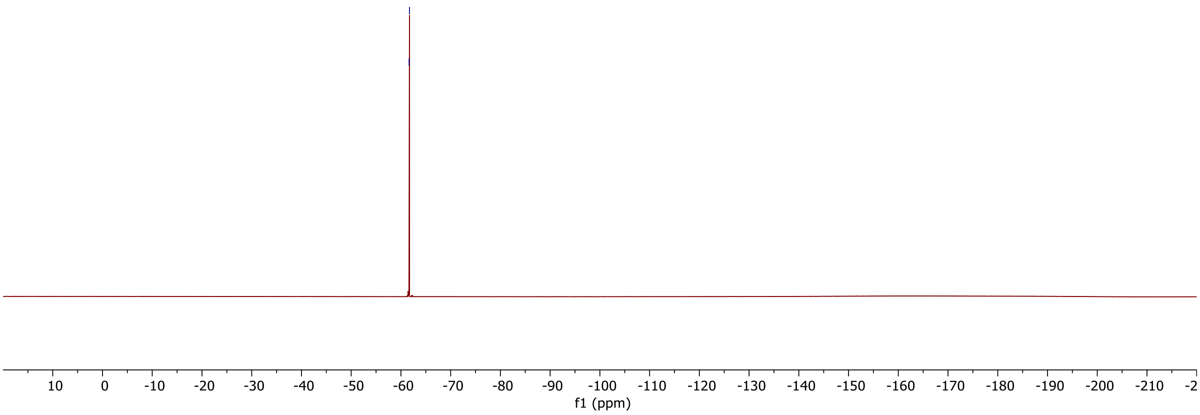

15. References

1 S. Protti, D. Ravelli, M. Fagnoni and A. Albini, *Chem. Commun.*, 2009, 7351–7353.

2 M. Lepori, C. Pratley, I. Dey, V. Butera, V. Roider and J. P. Barham, *Chem.-Eur. J.*, 2025, **31**, e202500666.

3 M. Majchrzak, G. Wilkowski and M. Kubicki, *Eur. J. Org. Chem.*, 2017, **2017**, 4291–4299.

4 G.-Z. Wang, R. Shang, W.-M. Cheng and Y. Fu, *J. Am. Chem. Soc.*, 2017, **139**, 18307–18312.

5 L. Tan, M. Boehme, L. Papworth, K. Wu, Y. Qiu and G. Li, *Eur. J. Org. Chem.*, 2025, **28**, e202401069.

6 J. Xu, W. Qiu, X. Zhang, Z. Wu, Z. Zhang, K. Yang and Q. Song, *Angew. Chem., Int. Ed.*, 2023, **62**, e202313388.

7 Á. L. Fuentes de Arriba, E. Lenci, M. Sonawane, O. Formery and D. J. Dixon, *Angew. Chem., Int. Ed.*, 2017, **129**, 3709–3713.

8 S. Kawamorita, T. Miyazaki, T. Iwai, H. Ohmiya and M. Sawamura, *J. Am. Chem. Soc.*, 2012, **134**, 12924–12927.

9 T. M. Masson, S. D. A. Zondag, J. H. A. Schuurmans and T. Noël, *React. Chem. Eng.*, 2024, **9**, 2218–2225.

10 F. Lukas, M. T. Findlay, M. Fillols, J. Templ, E. Savino, B. Martin, S. Allmendinger, M. Furegati and T. Noël, *Angew. Chem., Int. Ed.*, 2024, **63**, e202405902.

11 D. M. Schultz, F. Lévesque, D. A. DiRocco, M. Reibarkh, Y. Ji, L. A. Joyce, J. F. Dropinski, H. Sheng, B. D. Sherry and I. W. Davies, *Angew. Chem., Int. Ed.*, 2017, **56**, 15274–15278.

12 G. Laudadio, S. Govaerts, Y. Wang, D. Ravelli, H. F. Koolman, M. Fagnoni, S. W. Djuric and T. Noël, *Angew. Chem., Int. Ed.*, 2018, **130**, 4142–4146.

13 P. Xie, C. Xue, J. Luo, S. Shi and D. Du, *Green Chem.*, 2021, **23**, 5936–5943.

14 Y.-L. Shih, Y.-K. Wu, M. Hyodo and I. Ryu, *J. Org. Chem.*, 2023, **88**, 6548–6552.

15 J. P. Barham, M. P. John and J. A. Murphy, *J. Am. Chem. Soc.*, 2016, **138**, 15482–15487.

16 A. S. H. Ryder, W. B. Cunningham, G. Ballantyne, T. Mules, A. G. Kinsella, J. Turner-Dore, C. M. Alder, L. J. Edwards, B. S. J. McKay, M. N. Grayson and A. J. Cresswell, *Angew. Chem., Int. Ed.*, 2020, **132**, 15096–15101.

17 V. De Waele, O. Poizat, M. Fagnoni, A. Bagno and D. Ravelli, *ACS Catal.*, 2016, **6**, 7174–7182.

18 D. Chun, S. B. Lee, S. Chun, S. H. Choi, J. Hong, H. Lee, J. Lee and S. Hong, *Org. Biomol. Chem.*, 2024, **22**, 8617–8624.

19 R.-J. Song, Y.-Q. Tu, D.-Y. Zhu, F.-M. Zhang and S.-H. Wang, *Chem. Commun.*, 2015, 749–752.

20 Z. Yang, Z. Liu, H. Zhang, B. Yu, Y. Zhao, H. Wang, G. Ji, Y. Chen, X. Liu and Z. Liu, *Chem. Commun.*, 2017, 929–932.

21 M. C. Maust and S. B. Blakey, *ACS Catal.*, 2024, **14**, 2582–2587.

22 R. F. Renneke, M. Pasquali and C. L. Hill, *J. Am. Chem. Soc.*, 1990, **112**, 6585–6594.

23 I. B. Perry, T. F. Brewer, P. J. Sarver, D. M. Schultz, D. A. DiRocco and D. W. C. MacMillan, *Nature*, 2018, **560**, 70–75.

24 P. J. Sarver, N. B. Bissonnette and D. W. C. MacMillan, *J. Am. Chem. Soc.*, 2021, **143**, 9737–9743.

25 M. Leone, D. Arnaldi and M. Fagnoni, *Org. Chem. Front.*, 2025, **12**, 4970-4979.

26 N. Sellet, L. Clement-Comoy, M. Elhabiri, M. Cormier and J. Goddard, *Chem.-Eur. J.*, 2023, **29**, e202302353.

27 D. Kalaitzakis, A. Bosveli, K. Sfakianaki, T. Montagnon and G. Vassilikogiannakis, *Angew. Chem., Int. Ed.*, 2021, **133**, 4381–4387.

28 F. H. Westheimer, *Chem. Rev.*, 1961, **61**, 265–273.

29 O. P. Datsenko, A. Baziievskyi, I. Sadkova, B. Campos, J. T. Brewster, J. Kowalski, R. J. Hinklin and P. K. Mykhailiuk, *Org. Lett.*, 2025, **27**, 5318–5323.

30 R. Eskandrani, L. S. Al-Rasheed, S. A. Ansari, A. H. Bakheit, A. A. Almehizia, M. Almutairi and H. M. Alkahtani, *Molecules*, 2023, **28**:4271.

31 Y. Tian, X.-T. Li, J.-R. Liu, J. Cheng, A. Gao, N.-Y. Yang, Z. Li, K.-X. Guo, W. Zhang, H.-T. Wen, Z.-L. Li, Q.-S. Gu, X. Hong and X.-Y. Liu, *Nat. Chem.*, 2024, **16**, 466–475.

32 R. K. Rej, T. Das, S. Hazra and S. Nanda, *Tetrahedron: Asymmetry*, 2013, **24**, 913–918.

33 M. Su, X. Huang, C. Lei and J. Jin, *Org. Lett.*, 2022, **24**, 354–358.

34 L. Liu, Y. Guo, L. Shi, Y. Wang, X. Lei and P. Jiao, *Chem. Commun.*, 2025, 5170–5173.

35 D. Cao, S. Xia, L. Li, H. Zeng and C.-J. Li, *Org. Lett.*, 2024, **26**, 6418–6423.

36 Y. Wang, J. Zhang, F. Wang and L. Wu, *Angew. Chem., Int. Ed.*, 2025, **137**, e202420092.

37 N. Zhu, T. Wang, L. Ge, Y. Li, X. Zhang and H. Bao, *Org. Lett.*, 2017, **19**, 4718–4721.

38 Y. Gao, Y. Ou and L. J. Gooßen, *Chem.-Eur. J.*, 2019, **25**, 8709–8712.

39 H. Yan, L. Lu, G. Rong, D. Liu, Y. Zheng, J. Chen and J. Mao, *J. Org. Chem.*, 2014, **79**, 7103–7111.

40 X.-H. Yang, W.-T. Wei, H.-B. Li, R.-J. Song and J.-H. Li, *Chem. Commun.*, 2014, 12867–12869.
